# Variability in trends of opioid-related hospital utilization among U.S. Adults, 2016–2021 check

**DOI:** 10.1016/j.eclinm.2025.103355

**Published:** 2025-07-12

**Authors:** Lingxiao Chen, Zhuo Chen, Jiaming Ding, Roger Chou, Claire E. Ashton-James, Baoyi Shi, Stephanie Mathieson, Maja R. Radojčić, David B. Anderson, Ruiyuan Zheng, Runhan Fu, Yujie Chen, Lei Qi, Hengxing Zhou, Shiqing Feng, Manuela L. Ferreira

**Affiliations:** a Department of Orthopaedics, Qilu Hospital of Shandong University, Shandong University Centre for Orthopaedics, Advanced Medical Research Institute, Cheeloo College of Medicine, Shandong University, Jinan, Shandong, 250012, PR China; b Department of Biostatistics, School of Public Health, Cheeloo College of Medicine, Shandong University, Jinan, Shandong, 250012, PR China; c Division of Psychology and Mental Health, Faculty of Biology, Medicine and Health, University of Manchester, Manchester, UK; d Department of Medical Informatics and Clinical Epidemiology, Oregon Health & Science University, Portland, OR, USA; e Sydney Medical School, Faculty of Medicine and Health, Pain Management Research Institute, Kolling Institute, University of Sydney, Sydney, New South Wales, Australia; f Department of Biostatistics, Mailman School of Public Health, Columbia University, NY, USA; g Sydney School of Public Health, Faculty of Medicine and Health, The University of Sydney, Sydney, Australia; h Sydney Pharmacy School, Faculty of Medicine and Health, The University of Sydney, Sydney, Australia; i Sydney Musculoskeletal Health, School of Health Sciences, Faculty of Medicine and Health, The University of Sydney, Sydney, Australia; j Program in Child Health Evaluative Sciences, The Hospital for Sick Children, Toronto, ON, Canada; k Institute of Health Policy, Management and Evaluation, University of Toronto, Toronto, ON, Canada; l The Second Hospital of Shandong University, Cheeloo College of Medicine, Shandong University, Jinan, Shandong, 250033, PR China; m Center for Reproductive Medicine, Shandong University, Jinan, Shandong, 250012, PR China; n The George Institute for Global Health, University of New South Wales, Sydney, NSW, Australia

**Keywords:** Opioid, Variability, Hospital utilization, COVID-19 pandemic, Pain

## Abstract

**Background:**

Understanding trends in opioid-related hospital utilization is crucial for informing public health policies; however, existing research is often limited in scope and methodology. This study provides national estimates from 2016 to 2021, emphasizing the variability in trends across different opioid categories and subpopulations.

**Methods:**

This study employed a repeated cross-sectional analysis using data from the National Inpatient Sample (NIS) and Nationwide Emergency Department Sample (NEDS). Analyses were performed in two periods: 2016–2019 and 2019–2021 (during the COVID-19 pandemic). Outcomes included rates of opioid-related diagnoses and three types of opioid use disorder-related clinical events: nonfatal opioid overdose, injection drug use-related acute infection, and substance abuse treatment. Further analyses were conducted by opioid category (e.g., heroin and synthetic opioids as a proxy for fentanyl), as well as subgroup analyses based on predefined demographic characteristics, including age, sex, race/ethnicity, socioeconomic status, and geographic location.

**Findings:**

Between 2016 and 2019, in the NIS, there was a significant decrease in the rate of opioid-related diagnoses (relative change: −5.4%, 95% Cl: −9.4 to −1.3), nonfatal opioid overdose (−18.4%, −21.7 to −15.0), and substance abuse treatment (−25.1%, −45.9 to −4.3). Conversely, the rate of injection drug use-related acute infection increased significantly (14.4%, 7.3–21.4). In the NEDS, the rates of these outcomes did not change significantly. Notable variations were observed; for instance, in the NIS, the rate of nonfatal synthetic opioids as a proxy for fentanyl overdose increased by 21.1% (11.6–30.5), and heroin-related adverse event or poisoning increased by 51.8% (16.8–86.8) among adults aged 65–84. Between 2019 and 2021, in both the NIS and NEDS, the rate of nonfatal opioid overdose increased significantly (NIS: 8.1%, 3.5–12.7; NEDS: 24.8%, 11.5–38.0), in the NIS, a significant increase was found in the rate of injection drug use-related acute infection (relative increase: 8.2%, 1.2–15.1), while the rates of the other outcomes did not change significantly. Significant variations were also identified; for example, in the NIS, the rate of nonfatal opioid overdose did not show significant change among females, non-Hispanic whites, and adults with higher socioeconomic status.

**Interpretation:**

The significant variability in opioid-related hospital utilization trends among U.S. adults underscores the need for careful consideration in the design of future policies, especially during crises. Management strategies should be tailored to specific subpopulations, opioid categories, and OUD-related clinical events to maximize success rates.

**Funding:**

Taishan Scholars Program of Shandong Province-Pandeng Taishan Scholars.

**Research in context**

**Evidence before this study**

We searched PubMed for studies published from the database’s inception to August 1, 2024, using the following search term: (“analgesics opioid”[Pharmacological Action] OR “analgesics, opioid”[MeSH Terms] OR (“analgesics”[All Fields] AND “opioid”[All Fields]) OR “opioid analgesics”[All Fields] OR “opioid”[All Fields] OR “opioids”[All Fields] OR “opioid s”[All Fields]) AND (“trends”[All Fields] OR “trend”[All Fields]) AND (“America”[All Fields] OR “the United States”[All Fields]). Since 2016, we identified three relevant US studies: one utilized 2016–2017 Nationwide Emergency Department Sample (NEDS) data, another grouped all opioid categories into a single type, and the third covered a brief period during the COVID-19 pandemic (up to October 10, 2020).

**Added value of this study**

This study is the most comprehensive to date in using nationally representative inpatient and emergency department data to assess trends in opioid-related events since 2016. Opioid-related events included opioid-related diagnoses and opioid use disorder-related clinical events which were reported as nonfatal opioid overdose, injection drug use-related acute infection, and substance abuse treatment. Using six waves of data from the National Inpatient Sample (NIS) and Nationwide Emergency Department Sample (NEDS) spanning 2016–2021, we demonstrated that the rates of opioid-related events exhibited non-linear trends. Moreover, these trends varied across different opioid categories and predefined demographic characteristics. Between 2016 and 2019, in the NIS, the rate of opioid-related diagnoses, nonfatal opioid overdose, and substance abuse treatment decreased significantly, while the rate of injection drug use-related acute infection increased significantly; in the NEDS, the rate of these outcomes did not change significantly. Between 2019 and 2021 (during the COVID-19 pandemic), in both the NIS and NEDS, the rate of nonfatal opioid overdose increased significantly, in the NIS, the rate of injection drug use-related acute infection increased significantly, while the rate of the other outcomes did not change significantly. Notable variations were observed. For instance, between 2016 and 2019, in the NIS, the rate of heroin-related adverse event or poisoning increased among adults aged 65–84; between 2019 and 2021, in the NIS, the rate of nonfatal opioid overdose did not show significant change among females, non-Hispanic whites, and adults with higher socioeconomic status.

**Implications of all the available evidence**

The significant variability in opioid-related hospital utilization trends among U.S. adults underscores the need for careful consideration in the design of future policies, especially during crises.

## Introduction

The opioid crisis continues to have a significant impact on the population of the United States (US).^1^^,^^2^ In the past 25 years, there have been four waves of the opioid crisis.^3^^,^^4^ The first wave began in the late 1990s, primarily driven by the non-medical use and addiction to prescription opioid analgesics. The second wave began in the mid-2000s, characterized by a rise in heroin use and the associated increase in heroin use disorder. The third wave began in the mid-2010s, marked by a rapid increase in the use of fentanyl and other synthetic opioids.^5^, ^6^, ^7^, ^8^ The fourth wave began recently, driven by the rising co-use of psychostimulant drugs and opioids.^9^, ^10^, ^11^, ^12^ In 2021, opioids were involved in 80,411 overdose deaths in the US, accounting for 75.4% of all drug overdose deaths.^13^ For each fatal drug overdose, there are many more nonfatal drug overdoses, which can lead to substantial hospital utilization (i.e., inpatient stays or emergency department [ED] visits) and other adverse health consequences.^14^ Therefore, understanding the trend of hospital utilization for opioid-related events is important for informing relevant public health policies.^15^

Although several previous studies have evaluated opioid-related hospitalizations, overdoses, and injection-related infections in both inpatient and ED settings, these studies were limited by using regional U.S. data, being outdated, and failing to analyze specific opioid categories.^16^, ^17^, ^18^, ^19^, ^20^ Therefore, a new study using more recent, nationally representative data and performing more detailed analyses is needed.

It is unclear what impact the COVID-19 pandemic may have had on trends in hospital utilization for opioid-related events. The National Center for Health Statistics has reported that the number of national overdose deaths involving any opioid significantly increased in 2020 (n = 68,630) and 2021 (n = 80,411) compared with 2019 (n = 49,860).^21^ However, few studies have reported on hospital utilization for opioid-related events during this period.^22^ Emphasizing the variability in trends across different opioid categories and subpopulations is crucial, as it reflects potential health disparities, which are a priority to eliminate under Healthy People 2030.^23^

The reason for choosing 2016 as the starting year is that the conversion from the International Classification of Diseases, Ninth Revision, Clinical Modification (ICD-9-CM) to ICD-10-CM occurred in October 2015, resulting in several changes to the codes used to identify opioid-related events.^24^ Thus, the objective of the current research is to assess the impact of the COVID-19 pandemic (specifically, during 2020–2021) on opioid-related hospital utilization and to report changes in the national estimates of opioid-related hospital utilization since 2016. Further analyses were conducted by opioid category, as well as subgroup analyses based on predefined demographic characteristics.

## Methods

### Ethics subsection

Institutional review board approval and patient written informed consent were not required as this study is a secondary analysis of publicly available deidentified databases. This study followed the Strengthening the Reporting of Observational Studies in Epidemiology (STROBE) reporting guideline.^25^

### Data sources

Analyses were based on two data sources: the National Inpatient Sample (NIS)^26^ and the Nationwide Emergency Department Sample (NEDS).^27^ Cases from inpatient stays were gathered from the NIS. The NIS is the largest publicly available all-payer inpatient healthcare database in the US, containing data from ∼7 million hospital stays each year. The unweighted NIS data approximates a 20% stratified sample of discharges from US community hospitals and the weighted data covers more than 97% of the US population. Cases from ED visits were gathered from the NEDS. The NEDS is the largest all-payer ED database in the US, containing data from over 28 million ED visits each year. The unweighted NEDS data approximates a 20% stratified sample of US hospital-owned ED visits and the weighted data covers about 95% of ED visits. Adults older than 18 years and six annual waves (2016–2021; 2021 was the latest available wave at the time of the data analysis) were included.

### Outcomes

To comprehensively reflect opioid-related events, we summarized information from prior literature, engaged in discussions within the research group from both clinical and public health perspectives, and incorporated insights from the reviewers. Outcomes included rates of opioid-related diagnoses and opioid use disorder (OUD)-related clinical events in both the NIS (per 10,000 hospitalizations) and NEDS (per 10,000 ED visits).

Cases of opioid-related diagnoses were identified following the definition proposed by the Healthcare Cost and Utilization Project (detailed in Supplementary Tables S1 and S2).^28^ Opioid-related diagnoses were classified into two subtypes: abuse or dependence, and adverse event or poisoning. The International Classification of Diseases, Tenth Revision, Clinical Modification (ICD-10-CM) coding system only allows adverse event or poisoning to be further classified by opioid category, namely synthetic opioids as a proxy for fentanyl, prescription natural/semisynthetic opioids as a proxy for opioid pain medications, heroin, opium, methadone, and other opioids, thus only this outcome was analyzed by opioid category.^28^

Cases of OUD-related clinical events (detailed in Supplementary Tables S2 and S3) were identified following the study conducted by Barnett et al.,^29^ as well as insights from the reviewers. These events were defined as nonfatal opioid overdose, injection drug use-related acute infection, and substance abuse treatment. These three types are not mutually exclusive, meaning that an encounter could involve two or more types. For injection drug use-related acute infection and substance abuse treatment, the patient needed to have a diagnosis of OUD in addition to the codes identifying these specific conditions.^29^ Nonfatal opioid overdose was classified by opioid category, including synthetic opioids as a proxy for fentanyl, prescription natural/semisynthetic opioids as a proxy for opioid pain medications, heroin, opium, methadone, and other opioids. Injection drug use-related acute infection included phlebitis, abscess and/or cellulitis, infectious arthritis, infectious endocarditis, and sepsis or bacteremia. Substance abuse treatment included detoxification services, individual counseling, group counseling, individual psychotherapy, family counseling, medication management, and pharmacotherapy.

### Statistical analyses

**Fig. 1:**
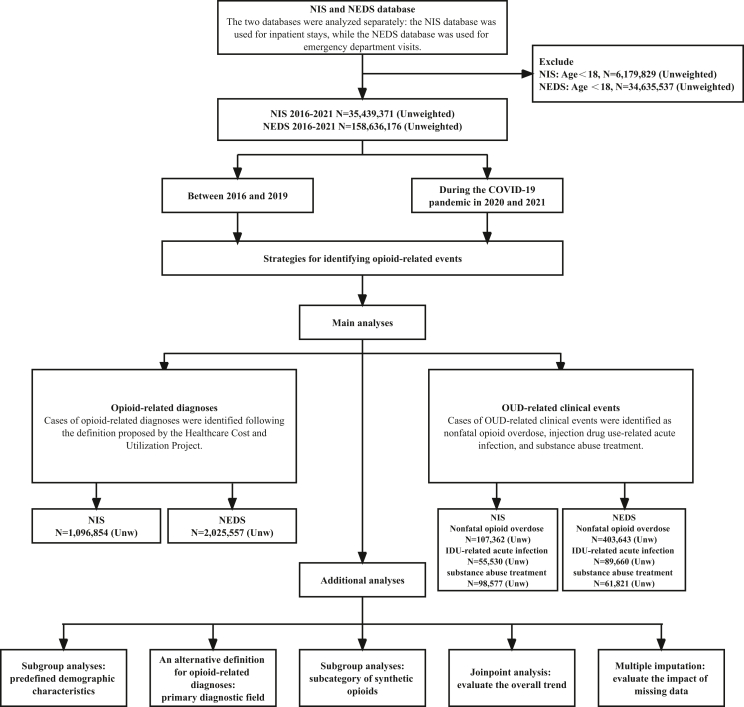
Flow chart and analysis framework.

Analyses were performed in two periods (Fig. 1): between 2016 and 2019 and during the COVID-19 pandemic (2020 and 2021). Differences in rates were compared using both absolute differences (i.e., between 2016 and 2019, estimate in 2019 minus estimate in 2016; during the COVID-19 pandemic, estimate in 2021 minus estimate in 2019) and corresponding percentage changes (e.g., [(estimate in 2019 minus estimate in 2016)/estimate in 2016] × 100%) with 95% confidence intervals (CIs).^30^ The 95% confidence intervals were calculated using the maximum pseudolikelihood method because both NIS and NEDS data are weighted and may not be independently distributed.^31^ This calculation represents a measure of overall change, as it is based on the estimate from the first and last data wave.^30^

Several additional analyses (detailed in Supplementary Methods) were performed. First, subgroup analyses were performed on predefined demographic characteristics (age, sex, race/ethnicity, socioeconomic status, and geographic location).^32^ Second, to address potential misclassification issues, an alternative definition for opioid-related diagnoses was provided, restricted to cases identified from the primary diagnostic field.^15^ Third, subgroup analyses were performed on the subcategory of synthetic opioids (i.e., fentanyl and its analogs, tramadol, and other synthetic opioids) as the ICD-10-CM coding system allowed this separation since 2020 Q4.^28^ The results from the subgroup analyses are exploratory, as no multiplicity adjustment was conducted, which increases the risk of type I error.^33^^,^^34^ Fourth, Joinpoint analysis was conducted to evaluate the overall trend.^35^ Fifth, multiple imputation was used to evaluate the impact of missing data.^36^^,^^37^

As the NIS and NEDS collected data through the complex sampling strategy, weights were used to ensure that the estimates were nationally representative, and weights and design variables were included to obtain unbiased estimates and standard errors.^38^ Complete case analysis was performed (Supplementary Table S4).^39^ In accordance with the database’s management rules to minimize the risk of identifying individuals, “NR” (Not Reportable) was recorded when the number of observations in a given cell of the tabulated data was ≤10.^40^ Data were analyzed through Stata version 17.0 (StataCorp), SPSS version 27.0 (IBM Corp), and Joinpoint program (version 5.2.0). Stata was used to estimate the rates and their 95% confidence intervals, as well as to perform multiple imputation. SPSS was used to identify, extract, and integrate data. The Joinpoint program was used to assess the overall trend between 2016 and 2021.

### Role of the funding source

The funders of this study had no involvement in study design, data collection, data analysis, data interpretation, or writing of the report.

## Results

### Characteristics of the study sample

For the NIS between 2016 and 2021, there were 35,439,371 sampled hospitalizations, among which there were 1,096,854 hospitalizations with opioid-related diagnoses, 107,362 hospitalizations with nonfatal opioid overdose, 55,530 hospitalizations with injection drug-use related acute infection, and 98,577 hospitalizations receiving substance abuse treatment (Supplementary Table S5). For the NEDS between 2016 and 2021, there were 158,636,176 sampled ED visits, among which there were 2,025,557 ED visits with opioid-related diagnoses, 403,643 ED visits with nonfatal opioid overdose, 89,660 ED visits with injection drug-use related acute infection, and 61,821 ED visits receiving substance abuse treatment (Supplementary Table S5). For the NIS in 2021, there were 5,688,355 sampled hospitalizations, among which there were 166,922 hospitalizations with opioid-related diagnoses, 16,663 hospitalizations with nonfatal opioid overdose, 9810 hospitalizations with injection drug-use related acute infection, and 12,973 hospitalizations receiving substance abuse treatment (Table 1). For the NEDS in 2021, there were 25,213,348 sampled ED visits, among which there were 324,483 ED visits with opioid-related diagnoses, 72,850 ED visits with nonfatal opioid overdose, 13,912 ED visits with injection drug-use related acute infection, and 8686 ED visits receiving substance abuse treatment (Table 1). Subgroup results can be found in Table 1 and Supplementary Table S6.

**Table 1:** Demographic distribution of hospitalizations and emergency department visits and opioid-related diagnoses^a^ and opioid use disorder-related clinical events^b^ in 2021.^c^

Characteristic	Opioid-related diagnoses	Opioid Use Disorder-related clinical events			All hospitalizations/ emergency department visits^d^
		Nonfatal opioid overdose	Injection drug-use related acute infection	Substance abuse treatment	
A. National Inpatient Sample					
Unweighted No. (weighted %)	166,922 (2.9)	16,663 (0.3)	9810 (0.2)	12,973 (0.2)	5,688,355
Age					
18–44	40.5	45.1	60.2	64.8	28.8
45–64	36.1	35.8	33.9	30.8	27.4
65–84	20.9	17.7	5.8	4.3	34.9
≥85	2.4	1.4	NRe	NR^e^	8.9
Sex					
Male	51.5	58.1	62.2	65.5	43.9
Female	48.5	41.8	37.8	34.5	56.1
Race/ethnicity^f^					
Non-Hispanic white	70.7	65.4	69.8	68.9	65.4
Non-Hispanic black	15.1	16.8	11.7	15.3	15.6
Hispanic	9.8	10.0	11.8	10.8	12.4
Non-Hispanic Asian or Pacific Islander	0.9	0.7	0.5	0.6	2.9
Native American	1.0	0.9	1.3	1.1	0.7
Other	2.6	3.1	2.5	3.3	2.9
B. Nationwide Emergency Department sample					
Unweighted No. (weighted %)	324,483 (1.3)	72,850 (0.3)	13,912 (0.06)	8686 (0.04)	25,213,348
Age					
18–44	52.2	62.5	62.2	64.4	44.5
45–64	32.4	28.4	32.0	30.8	29.0
65–84	13.9	8.4	5.7	4.7	21.5
≥85	1.5	0.7	0.1	NR^e^	4.9
Sex					
Male	58.4	66.1	63.0	67.5	44.9
Female	41.6	33.8	37.0	32.5	55.1
Race/ethnicity^f^					
Non-Hispanic White	67.4	64.6	70.0	73.0	57.6
Non-Hispanic Black	16.1	16.5	11.2	11.1	20.0
Hispanic	11.6	11.1	13.4	10.1	16.3
Non-Hispanic Asian or Pacific Islander	0.8	0.6	0.5	0.3	2.4
Native American	1.1	1.4	1.2	1.4	0.7
Other	2.9	3.1	2.5	4.1	3.1

a Cases of opioid-related diagnoses included two subtypes: abuse or dependence, and adverse event or poisoning (classified by opioid category, including: synthetic as a proxy for fentanyl, prescription natural/semisynthetic as a proxy for opioid pain meds, heroin, opium, methadone, and other opioids).

b Cases of opioid use disorder-related clinical events represented as nonfatal opioid overdose (classified by opioid category, including: synthetic as a proxy for fentanyl, prescription natural/semisynthetic as a proxy for opioid pain meds, heroin, opium, methadone, and other opioids), injection drug use-related acute infection, and substance abuse treatment.

c Weights provided by the Healthcare Cost and Utilization Project, National Inpatient Sample/Nationwide Emergency Department Sample were used to ensure that the estimates were nationally representative, and weights and design variables were included to obtain unbiased estimates and standard errors.

d Hospitalizations Source: Agency for Healthcare Research and Quality, Healthcare Cost and Utilization Project, National Inpatient Sample. Emergency department visits Source: Agency for Healthcare Research and Quality, Healthcare Cost and Utilization Project, Nationwide Emergency Department Sample.

e NR, Not Reportable. Suppressed to protect confidentiality, ≤10 cases.

f Race/ethnicity variable was obtained from the Agency for Healthcare Research and Quality, Healthcare Cost and Utilization Project; nationally weighted from all records in states with reliable race/ethnicity reporting, which is collected by self-report on admission to the hospital using fixed categories.

### Opioid-related diagnoses

**Fig. 2:**
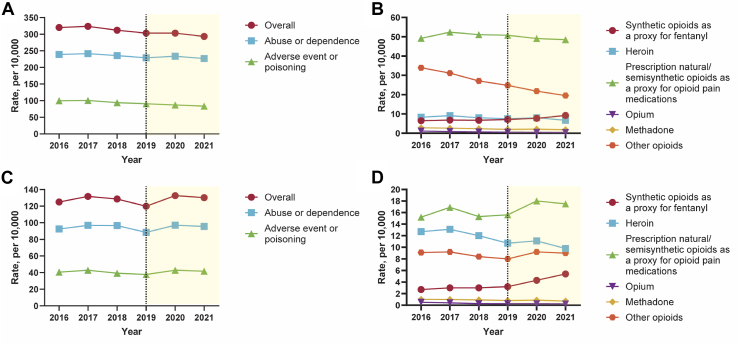
Opioid-related diagnoses (2016–2021) overall and for subtypes involving opioid categories of adverse event or poisoning in the national Inpatient Sample (A, B) and nationwide emergency department sample (C, D). Note: Panel A. Opioid-related diagnoses overall in National Inpatient Sample, Panel B. Opioid categories of adverse event or poisoning in National Inpatient Sample, Panel C. Opioid-related diagnoses overall in Nationwide Emergency Department Sample, Panel D. Opioid categories of adverse event or poisoning in Nationwide Emergency Department Sample.

Between 2016 and 2019, the rate of opioid-related diagnoses per 10,000 hospitalizations decreased significantly (relative decrease: 5.4%, 95% CI 1.3–9.4); while the rate of opioid-related diagnoses per 10,000 ED visits did not change significantly (relative change: −4.0%, −12.2 to 4.2). In both the NIS and NEDS, no significant change was found in the rate of opioid-related abuse or dependence. In the NIS, significant decreases (relative decrease: 8.9%, 5.8–12.0) were found for the rate of opioid-related adverse event or poisoning and for all opioid categories, except for prescription natural/semisynthetic opioids as a proxy for opioid pain medications, which did not change significantly, and synthetic opioids as a proxy for fentanyl, which increased significantly (relative increase: 9.1%, 3.1–15.1). In the NEDS, no significant change was found for the rate of opioid-related adverse event or poisoning, while variations were observed within opioid categories (e.g., synthetic opioids as a proxy for fentanyl, relative increase: 17.9%, 7.3–28.4) (Table 2, Table 3, Fig. 2 and Supplementary Tables S7 and S8).

**Table 2:** Opioid-related diagnoses^a^ (2016–2019 and 2020–2021) overall and for subtypes involving opioid categories of adverse event or poisoning in the national inpatient sample.^b^

Characteristics	Between 2016 and 2019			During the COVID-19 pandemic in 2020 and 2021		
	2019 Rate, per 10,000	Absolute difference from 2016 to 2019^c^	Percentage change from 2016 to 2019, %	2021 Rate, per 10,000	Absolute difference from 2019 to 2021^d^	Percentage change from 2019 to 2021, %
Overall	303.1 (294.3–311.9)	−17.1 (−30.1 to −4.2)	−5.4 (−9.4 to −1.3)	293.4 (284.7–302.2)	−9.6 (−22.0 to 2.8)	−3.2 (−7.3 to 0.9)
Abuse or dependence	229.1 (220.6–237.5)	−9.8 (−22.4 to 2.7)	−4.1 (−9.4 to 1.1)	226.8 (218.5–235.1)	−2.3 (−14.1 to 9.6)	−1.0 (−6.2 to 4.2)
Adverse event or poisoning	90.7 (88.5–92.9)	−8.9 (−12.0 to −5.7)	−8.9 (−12.0 to −5.8)	83.6 (81.3–85.9)	−7.1 (−10.3 to −3.9)	−7.9 (−11.4 to −4.3)
Synthetic opioids as a proxy for fentanyl	7.1 (6.8–7.4)	0.6 (0.2–1.0)	9.1 (3.1–15.1)	9.2 (8.7–9.6)	2.1 (1.6–2.6)	29.6 (22.0–37.2)
Prescription natural/semisynthetic opioids as a proxy for opioid pain medications	50.8 (49.0–52.6)	1.7 (−0.7 to 4.0)	3.4 (−1.4 to 8.1)	48.5 (46.6–50.4)	−2.3 (−5.0 to 0.3)	−4.6 (−9.8 to 0.6)
Heroin	7.5 (7.2–7.9)	−0.8 (−1.4 to −0.2)	−9.6 (−16.6 to −2.6)	6.7 (6.3–7.1)	−0.8 (−1.4 to −0.3)	−11.0 (−18.3 to −3.7)
Opium	0.5 (0.4–0.6)	−0.6 (−0.7 to −0.5)	−53.6 (−66.2 to −41.0)	0.4 (0.3–0.5)	−0.1 (−0.2 to −0.02)	−25.0 (−46.3 to −3.7)
Methadone	2.0 (1.9–2.2)	−0.7 (−1.0 to −0.5)	−26.3 (−34.7 to −17.8)	1.8 (1.6–1.9)	−0.3 (−0.5 to −0.1)	−14.6 (−24.8 to −4.5)
Other opioids	24.8 (24.1–25.5)	−9.1 (−10.3 to −7.9)	−26.7 (−30.3 to −23.2)	19.5 (18.9–20.1)	−5.3 (−6.3 to −4.4)	−21.4 (−25.3 to −17.5)

a Cases of opioid-related diagnoses were identified following the definition proposed by the Healthcare Cost and Utilization Project, including two subtypes: abuse or dependence, and adverse event or poisoning (classified by opioid category, including: synthetic opioids as a proxy for fentanyl, prescription natural/semisynthetic opioids as a proxy for opioid pain medications, heroin, opium, methadone, and other opioids).

b Weights provided by the Healthcare Cost and Utilization Project, National Inpatient Sample were used to ensure that the estimates were nationally representative, and weights and design variables were included to obtain unbiased estimates and standard errors.

c Reflects the overall or total difference between 2016 and 2019: absolute difference (2019–2016) and percentage change [(2019–2016)/2016 × 100].

d Reflects the overall or total difference during the COVID-19 pandemic in 2020 and 2021: absolute difference (2021–2019) and percentage change [(2021–2019)/2019 × 100].

**Table 3:** Opioid-related diagnoses^a^ (2016–2019 and 2020–2021) overall and for subtypes involving opioid categories of adverse event or poisoning in the nationwide emergency department sample.^b^

Characteristics	Between 2016 and 2019			During the COVID-19 pandemic in 2020 and 2021		
	2019 Rate, per 10,000	Absolute difference from 2016 to 2019^c^	Percentage change from 2016 to 2019, %	2021 Rate, per 10,000	Absolute difference from 2019 to 2021^d^	Percentage change from 2019 to 2021, %
Overall	119.8 (112.2–127.4)	−5.0 (−15.3 to 5.2)	−4.0 (−12.2 to 4.2)	130.2 (120.1–140.4)	10.4 (−2.3 to 23.1)	8.7 (−1.9 to 19.2)
Abuse or dependence	88.4 (82.0–94.9)	−4.0 (−12.7 to 4.8)	−4.3 (−13.8 to 5.2)	95.5 (86.1–104.8)	7.0 (−4.3 to 18.4)	8.0 (−4.9 to 20.8)
Adverse event or poisoning	37.8 (35.6–40.1)	−2.7 (−6.1 to 0.7)	−6.6 (−15.0 to 1.8)	41.7 (39.5–43.9)	3.9 (0.7–7.0)	10.2 (1.8–18.6)
Synthetic opioids as a proxy for fentanyl	3.2 (3.0–3.5)	0.5 (0.2–0.8)	17.9 (7.3–28.4)	5.4 (4.9–5.8)	2.1 (1.6–2.7)	65.9 (49.4–82.5)
Prescription natural/semisynthetic opioids as a proxy for opioid pain medications	15.6 (14.7–16.5)	0.4 (−0.7 to 1.6)	2.8 (−4.6 to 10.3)	17.5 (16.6–18.4)	1.9 (0.6–3.2)	12.2 (3.9–20.4)
Heroin	10.7 (9.5–11.8)	−2.1 (−4.3 to 0.2)	−16.1 (−33.6 to 1.3)	9.8 (8.5–11.1)	−0.8 (−2.6 to 0.9)	−7.8 (−24.2 to 8.6)
Opium	0.3 (0.2–0.3)	−0.2 (−0.3 to −0.2)	−48.0 (−62.1 to −33.9)	0.2 (0.2–0.3)	−0.04 (−0.1 to 0.03)	−15.4 (−42.6 to 11.8)
Methadone	0.8 (0.7–0.9)	−0.2 (−0.3 to −0.1)	−20.8 (−33.2 to −8.4)	0.7 (0.7–0.8)	−0.08 (−0.2 to 0.05)	−10.0 (−25.7 to 5.7)
Other opioids	8.0 (7.3–8.7)	−1.0 (−1.9 to −0.2)	−11.5 (−21.0 to −2.0)	9.0 (8.4–9.5)	0.9 (0.05–1.8)	11.7 (0.6–22.9)

a Cases of opioid-related diagnoses were identified following the definition proposed by the Healthcare Cost and Utilization Project, including two subtypes: abuse or dependence, and adverse event or poisoning (classified by opioid category, including: synthetic opioids as a proxy for fentanyl, prescription natural/semisynthetic opioids as a proxy for opioid pain medications, heroin, opium, methadone, and other opioids).

b Weights provided by the Healthcare Cost and Utilization Project, Nationwide Emergency Department Sample were used to ensure that the estimates were nationally representative, and weights and design variables were included to obtain unbiased estimates and standard errors.

c Reflects the overall or total difference between 2016 and 2019: absolute difference (2019–2016) and percentage change [(2019–2016)/2016 × 100].

d Reflects the overall or total difference during the COVID-19 pandemic in 2020 and 2021: absolute difference (2021–2019) and percentage change [(2021–2019)/2019 × 100].

During the COVID-19 pandemic in 2020 and 2021, the rate of opioid-related diagnoses and opioid-related abuse or dependence did not change significantly in either the NIS or NEDS. In the NIS, significant decreases were found for the rate of opioid-related adverse event or poisoning (relative decrease: 7.9%, 4.3–11.4) and all opioid categories except for prescription natural/semisynthetic opioids as a proxy for opioid pain medications, which did not change significantly, and synthetic opioids as a proxy for fentanyl, which increased significantly (relative increase: 29.6%, 22.0–37.2). In the NEDS, significant increases were found for the rate of opioid-related adverse event or poisoning (relative increase: 10.2%, 1.9–18.6) mainly due to the use of prescription natural/semisynthetic opioids as a proxy for opioid pain medications (relative increase: 12.2%, 3.9–20.4), synthetic opioids as a proxy for fentanyl (relative increase: 65.9%, 49.4–82.5), and other opioids (relative increase: 11.7%, 0.6–22.9) (Table 2, Table 3, Fig. 2 and Supplementary Tables S7 and S8).

### OUD-related clinical events

**Fig. 3:**
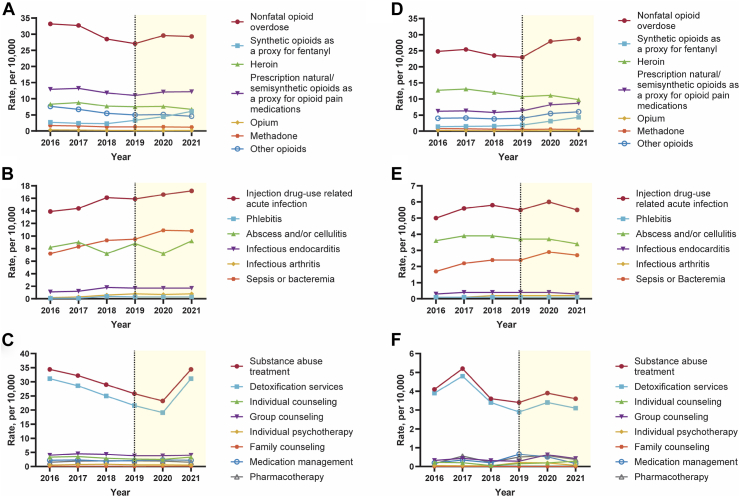
Opioid use disorder-related clinical events (2016–2021) overall and for subtypes in the National Inpatient Sample (A–C) and nationwide emergency department sample (D–F). Note: Panel A. Nonfatal opioid overdose overall and for Subtypes in National Inpatient Sample, Panel B. Injection drug-use related acute infection overall and for Subtypes in National Inpatient Sample, Panel C. Substance abuse treatment overall and for Subtypes in National Inpatient Sample, Panel D. Nonfatal opioid overdose overall and for Subtypes in Nationwide Emergency Department Sample, Panel E. Injection drug-use related acute infection overall and for Subtypes in Nationwide Emergency Department Sample, Panel F. Substance abuse treatment overall and for Subtypes in Nationwide Emergency Department Sample.

Between 2016 and 2019, significant decreases were found in the rate of nonfatal opioid overdose per 10,000 hospitalizations (relative decrease: 18.4%, 15.0–21.7) and all opioid categories except for synthetic opioids as a proxy for fentanyl, which significantly increased (relative increase: 21.1%, 11.6–30.6). The rate of nonfatal opioid overdose per 10,000 ED visits did not change significantly (relative change: −7.3%, −20.1 to 5.6), while variations were observed within opioid categories (e.g., synthetic opioids as a proxy for fentanyl, relative increase: 39.6%, 21.6–57.6). Significant increases were found in the rate of injection drug use-related acute infection per 10,000 hospitalizations (relative increase: 14.4%, 7.3–21.4) and all subcategories (e.g., phlebitis, relative increase: 303.0%, 239.0–367.1) except for abscess and/or cellulitis, which did not significantly change; the rate of injection drug use-related acute infection per 10,000 ED visits did not change significantly, while variations were observed within subcategories (e.g., sepsis or bacteremia, relative increase: 41.2%, 24.9–57.5). Significant decreases were found in the rate of substance abuse treatment per 10,000 hospitalizations (relative decrease: 25.1%, 4.3–45.9); the rate of substance abuse treatment per 10,000 ED visits did not change significantly (Table 4, Table 5, Fig. 3 and Supplementary Tables S9 and S10).

**Table 4:** Opioid use disorder-related clinical events^a^ (2016–2019 and 2020–2021) overall and for subtypes in the national inpatient sample.^b^

OUD-related clinical events	Between 2016 and 2019			During the COVID-19 pandemic in 2020 and 2021		
	2019 Rate, per 10,000	Absolute difference from 2016 to 2019^c^	Percentage change from 2016 to 2019, %	2021 Rate, per 10,000	Absolute difference from 2019 to 2021^d^	Percentage change from 2019 to 2021, %
A. Nonfatal opioid overdose	27.1 (26.3–27.9)	−6.1 (−7.2 to −5.0)	−18.4 (−21.7 to −15.0)	29.3 (28.3–30.2)	2.2 (0.9–3.5)	8.1 (3.5–12.7)
Synthetic opioids as a proxy for fentanyl	3.3 (3.1–3.5)	0.6 (0.3–0.8)	21.1 (11.6–30.5)	6.1 (5.7–6.5)	2.8 (2.4–3.3)	86.6 (72.5–100.6)
Prescription natural/semisynthetic opioids as a proxy for opioid pain medications	11.0 (10.7–11.4)	−1.9 (−2.4 to −1.4)	−14.5 (−18.5 to −10.5)	12.2 (11.8–12.6)	1.2 (0.6–1.7)	10.7 (5.8–15.6)
Heroin	7.5 (7.2–7.9)	−0.8 (−1.4 to −0.2)	−9.6 (−16.6 to −2.6)	6.7 (6.3–7.1)	−0.8 (−1.4 to −0.3)	−11.0 (−18.2 to −3.7)
Opium	0.2 (0.1–0.2)	−0.2 (−0.2 to −0.1)	−50.0 (−69.0 to −31.0)	0.1 (0.1–0.2)	−0.02 (−0.07 to 0.03)	−13.4 (−43.2 to 16.5)
Methadone	1.3 (1.2–1.4)	−0.4 (−0.6 to −0.2)	−22.6 (−32.4 to −12.8)	1.2 (1.1–1.3)	−0.1 (−0.3 to 0.03)	−9.4 (−21.0 to 2.3)
Other opioids	5.0 (4.7–5.2)	−2.6 (−2.9 to −2.3)	−34.2 (−37.9 to −30.6)	4.6 (4.4–4.9)	−0.4 (−0.7 to −0.1)	−8.0 (−13.5 to −2.5)
B. Injection drug-use related acute infection	15.9 (15.2–16.6)	2.0 (1.0–3.0)	14.4 (7.3–21.4)	17.2 (16.4–18.0)	1.3 (0.2–2.4)	8.2 (1.2–15.1)
Phlebitis	0.3 (0.3–0.4)	0.3 (0.2–0.3)	303.0 (238.9–367.1)	0.3 (0.3–0.4)	−0.04 (−0.1 to 0.04)	−10.4 (−30.9 to 10.2)
Abscess and/or cellulitis	8.8 (8.4–9.3)	0.6 (0–1.2)	7.3 (−0.2 to 14.8)	9.2 (8.7–9.7)	0.4 (−0.3 to 1.0)	4.1 (−3.4 to 11.7)
Infectious endocarditis	1.7 (1.5–1.8)	0.6 (0.4–0.8)	49.9 (33.5–66.2)	1.7 (1.5–1.8)	−0.02 (−0.2 to 0.2)	−1.0 (−13.0 to 11.0)
Infectious arthritis	0.8 (0.7–0.9)	0.5 (0.4–0.6)	231.5 (190.6–272.4)	0.8 (0.7–0.9)	−0.01 (−0.1 to 0.1)	−1.2 (−17.1 to 14.8)
Sepsis or bacteremia	9.5 (9.0–9.9)	2.3 (1.7–2.9)	31.9 (24.2–39.6)	10.8 (10.2–11.3)	1.3 (0.6–2.0)	13.7 (6.2–21.1)
C. Substance abuse treatment	25.8 (21.2–30.3)	−8.6 (−15.8 to −1.5)	−25.1 (−45.9 to −4.3)	22.8 (19.1–26.6)	−3.0 (−8.9 to 2.9)	−11.5 (−34.4 to 11.3)
Detoxification services	21.6 (17.7–25.5)	−9.5 (−15.9 to −3.1)	−30.5 (−51.2 to −9.9)	18.9 (15.6–22.1)	−2.7 (−7.7 to 2.3)	−12.5 (−35.7 to 10.7)
Individual counseling	2.6 (1.7–3.6)	−0.7 (−2.2 to 0.8)	−21.2 (−67.6 to 25.2)	2.3 (1.3–3.3)	−0.3 (−1.7 to 1.1)	−11.5 (−64.8 to 41.8)
Group counseling	3.8 (2.6–5.0)	−0.2 (−2.0 to 1.6)	−5.0 (−50.2 to 40.2)	3.9 (2.6–5.2)	0.1 (−1.6 to 1.8)	2.6 (−41.1 to 46.4)
Individual psychotherapy	0.6 (0.2–1.0)	0.1 (−0.5 to 0.7)	20.0 (−90.9 to 130.9)	0.5 (0.2–0.8)	−0.1 (−0.7 to 0.5)	−16.7 (−109.1 to 75.7)
Family counseling	0.08 (−0.01 to 0.16)	−0.03 (−0.2 to 0.1)	−27.3 (−141.4 to 86.8)	0.09 (−0.01 to 0.19)	0.01 (−0.1 to 0.1)	12.5 (−144.4 to 169.4)
Medication management	2.1 (1.0–3.2)	−0.1 (−1.6 to 1.4)	−4.5 (−74.1 to 65.0)	1.9 (0.9–2.9)	−0.2 (−1.7 to 1.3)	−9.5 (−82.4 to 63.4)
Pharmacotherapy	2.0 (1.0–2.9)	0.5 (−0.6 to 1.6)	33.3 (−42.9 to 109.5)	1.7 (1.1–2.3)	−0.3 (−1.4 to 0.8)	−15.0 (−72.1 to 42.1)

a Cases of Opioid Use Disorder-related clinical events represented as nonfatal opioid overdose, injection drug use-related acute infection, and substance abuse treatment.

b Weights provided by the Healthcare Cost and Utilization Project, National Inpatient Sample was used to ensure that the estimates were nationally representative, and weights and design variables were included to obtain unbiased estimates and standard errors.

c Reflects the overall or total difference between 2016 and 2019: absolute difference (2019–2016) and percentage change [(2019–2016)/2016 × 100].

d Reflects the overall or total difference during the COVID-19 pandemic in 2020 and 2021: absolute difference (2021–2019) and percentage change [(2021–2019)/2019 × 100].

**Table 5:** Opioid use disorder-related clinical events^a^ (2016–2019 and 2020–2021) overall and for subtypes in the nationwide emergency department sample.^b^

OUD-related clinical events	Between 2016 and 2019			During the COVID-19 pandemic in 2020 and 2021		
	2019 Rate, per 10,000	Absolute difference from 2016 to 2019^c^	Percentage change from 2016 to 2019, %	2021 Rate, per 10,000	Absolute difference from 2019 to 2021^d^	Percentage change from 2019 to 2021, %
A. Nonfatal opioid overdose	23.0 (20.8–25.1)	−1.8 (−5.0 to 1.4)	−7.3 (−20.1 to 5.6)	28.7 (26.6–30.9)	5.7 (2.7–8.7)	24.8 (11.5–38.0)
Synthetic opioids as a proxy for fentanyl	1.9 (1.7–2.1)	0.5 (0.3–0.8)	39.6 (21.6–57.6)	4.3 (3.9–4.8)	2.4 (1.9–2.9)	125.4 (99.5–151.3)
Prescription natural/semisynthetic opioids as a proxy for opioid pain medications	6.3 (5.6–6.9)	0.1 (−0.7 to 0.8)	1.0 (−10.6 to 12.5)	8.7 (8.0–9.4)	2.4 (1.5–3.4)	39.0 (24.3–53.7)
Heroin	10.7 (9.5–11.8)	−2.1 (−4.3 to 0.2)	−16.1 (−33.5 to 1.2)	9.8 (8.5–11.1)	−0.8 (−2.6 to 0.9)	−7.8 (−24.1 to 8.5)
Opium	0.1 (0.1–0.1)	−0.1 (−0.1 to −0.06)	−47.1 (−66.3 to −27.8)	0.1 (0.1–0.2)	0.02 (−0.02 to 0.06)	17.9 (−18.9 to 54.8)
Methadone	0.5 (0.5–0.6)	−0.2 (−0.3 to −0.1)	−29.8 (−42.5 to −17.2)	0.5 (0.5–0.6)	−0.03 (−0.1 to 0.05)	−4.7 (−19.3 to 10.0)
Other opioids	4.0 (3.4–4.7)	0 (−0.7 to 0.7)	0 (−17.7 to 17.7)	6.0 (5.5–6.5)	2.0 (1.2–2.8)	50.0 (29.2–70.8)
B. Injection drug-use related acute infection	5.5 (5.0–6.0)	0.5 (−0.3 to 1.3)	10.0 (−6.6 to 26.6)	5.5 (5.0–6.0)	0 (−0.8 to 0.8)	0 (−15.1 to 15.1)
Phlebitis	0.1 (0.1–0.1)	−0.01 (−0.04 to 0.01)	−9.7 (−29.4 to 10.1)	0.1 (0.1–0.1)	−0.03 (−0.05 to −0.01)	−27.7 (−47.9 to −7.5)
Abscess and/or cellulitis	3.7 (3.3–4.0)	0.1 (−0.4 to 0.6)	2.9 (−11.8 to 17.6)	3.4 (3.0–3.8)	−0.3 (−0.8 to 0.2)	−7.4 (−21.4 to 6.7)
Infectious endocarditis	0.4 (0.3–0.4)	0.07 (0–0.1)	23.4 (−1.1 to 47.9)	0.3 (0.3–0.4)	−0.05 (−0.1 to 0.03)	−12.5 (−31.6 to 6.6)
Infectious arthritis	0.2 (0.1–0.2)	0.05 (0.02–0.08)	46.7 (15.4–78.1)	0.2 (0.1–0.2)	0 (−0.03 to 0.04)	1.9 (−20.2 to 24.0)
Sepsis or bacteremia	2.4 (2.2–2.7)	0.7 (0.4–1.0)	41.2 (24.9–57.5)	2.7 (2.5–3.0)	0.3 (0.02–0.6)	12.5 (1.0–24.0)
C. Substance abuse treatment	3.4 (2.2–4.5)	−0.7 (−2.4 to 1.0)	−17.1 (−57.6 to 23.5)	3.6 (2.5–4.8)	0.2 (−1.5 to 1.9)	5.9 (−43.0 to 54.8)
Detoxification services	2.9 (1.9–4.0)	−1.0 (−2.5 to 0.5)	−25.6 (−64.9 to 13.6)	3.1 (2.1–4.1)	0.2 (−1.2 to 1.6)	6.9 (−40.9 to 54.7)
Individual counseling	0.1 (−0.1 to 0.4)	−0.1 (−0.4 to 0.3)	−31.8 (−183 to 119.4)	0.3 (0.01–0.6)	0.1 (−0.2 to 0.5)	86.7 (−154.3 to 327.6)
Group counseling	0.3 (−0.1 to 0.6)	−0.04 (−0.6 to 0.5)	−12.5 (−186.6 to 161.6)	0.4 (0.03–0.8)	0.1 (−0.4 to 0.7)	50.0 (−138.4 to 238.4)
Individual psychotherapy	0.2 (−0.2 to 0.6)	0.2 (−0.2 to 0.6)	320.0 (−472.8 to 1112.8)	0.06 (−0.01 to 0.1)	−0.2 (−0.5 to 0.2)	−71.4 (−260.2 to 117.3)
Family counseling	NR^e^	NR^e^	NR^e^	NR^e^	NR^e^	NR^e^
Medication management	0.7 (0.02–1.3)	0.5 (−0.2 to 1.1)	242.1 (−98.2 to 582.4)	0.2 (0.06–0.3)	−0.5 (−1.1 to 0.1)	−75.4 (−173.0 to 22.3)
Pharmacotherapy	0.5 (−0.1 to 1.1)	0.4 (−0.3 to 1.0)	233.3 (−189.9 to 656.5)	0.4 (−0.02 to 0.8)	−0.1 (−0.9 to 0.6)	−26.0 (−173.9 to 121.9)

a Cases of Opioid Use Disorder-related clinical events represented as nonfatal opioid overdose, injection drug use-related acute infection, and substance abuse treatment.

b Weights provided by the Healthcare Cost and Utilization Project, Nationwide Emergency Department Sample was used to ensure that the estimates were nationally representative, and weights and design variables were included to obtain unbiased estimates and standard errors.

c Reflects the overall or total difference between 2016 and 2019: absolute difference (2019–2016) and percentage change [(2019–2016)/2016 × 100].

d Reflects the overall or total difference during the COVID-19 pandemic in 2020 and 2021: absolute difference (2021–2019) and percentage change [(2021–2019)/2019 × 100].

e NR, Not Reportable. Suppressed to protect confidentiality, ≤10 cases.

During the COVID-19 pandemic in 2020 and 2021, significant increases were found in the rate of nonfatal opioid overdose in the NIS and NEDS, mainly due to the use of prescription natural/semisynthetic opioids as a proxy for opioid pain medications (in the NIS, relative increase: 10.7%, 5.8–15.6; in the NEDS, relative increase: 39.0%, 24.3–53.7) and synthetic opioids as a proxy for fentanyl (in the NIS, relative increase: 86.6%, 72.5–100.6; in the NEDS, relative increase: 125.4%, 99.5–151.3). In the NIS, a significant increase was found in the rate of injection drug use-related acute infection (relative increase: 8.2%, 1.2–15.1), primarily driven by sepsis or bacteremia (relative increase: 13.7%, 6.2–21.1); in the NEDS, the rate of injection drug use-related acute infection did not change significantly, while the rate of sepsis or bacteremia increased significantly (relative increase: 12.5%, 1.0–24.0). In both the NIS and NEDS, the rate of substance abuse treatment and all subcategories did not change significantly (Table 4, Table 5, Fig. 3 and Supplementary Tables S9 and S10).

### Additional analyses

Subgroup analyses (Supplementary Figs. S1–S10 and Tables S11–S34) based on demographic characteristics revealed substantial variations. For example, between 2016 and 2019, in the NIS, heroin-related adverse event or poisoning increased by 51.8% (16.8–86.8) among adults aged 65–84. Between 2019 and 2021, the rate of nonfatal opioid overdose did not show significant change among females (−2.2%, −7.2 to 2.7), non-Hispanic whites (1.3%, −2.9 to 5.6), and adults with higher socioeconomic status (e.g., adults in the highest income quartile, −4.9%, −12.4 to 2.5). Between 2016 and 2019, in the NIS, the rate of nonfatal opioid overdose decreased significantly in all four regions. However, significant increases were observed in the South and West hospitals between 2019 and 2021. When the analysis of opioid-related diagnoses was limited to the primary diagnosis field, the results were comparable to the main analyses (Supplementary Tables S35 and S36). Subgroup analyses based on the subcategory of synthetic opioids indicated that fentanyl and its analogs were predominant, with tramadol and other synthetic opioids comprising a minority. For example, in the 2021 NIS, synthetic opioid-related diagnoses were distributed as follows: fentanyl and its analogs (67.3%), tramadol (24.9%), and other synthetic opioids (8.3%) (Supplementary Tables S37 and S38). The increased rate of synthetic opioid-related diagnoses in the NIS from 2020 Q4 to 2021 Q4 was primarily due to the use of fentanyl and its analogs, with a relative change of 40.0% (95% CI: 25.3–54.8) (Supplementary Tables S39 and S40). Between 2016 and 2021, Joinpoint analyses showed that in the NIS, the rate of opioid-related diagnoses and two subtypes decreased significantly, while in the NEDS, no significant change was found for opioid-related diagnoses and two subtypes; in both the NIS and NEDS, notable variations were observed within subcategories of OUD-related clinical events (Supplementary Figs. S11 and S12). The results from multiple imputation are consistent with the main analyses (Supplementary Table S4).

## Discussion

Between 2016 and 2019, in the NIS, the rate of opioid-related diagnoses, nonfatal opioid overdose, and substance abuse treatment decreased significantly, while the rate of injection drug use-related acute infection increased significantly. During the same period, in the NEDS, there was no significant change in the rate of these same outcomes. This difference may reflect variations in populations or settings between these two databases, indicating a need for further research. Notable variations were observed; for instance, in the NIS, there was an increase in the rate of nonfatal synthetic opioids as a proxy for fentanyl overdose as well as a rise in heroin-related adverse event or poisoning among adults aged 65–84.

During the COVID-19 pandemic in 2020 and 2021, the rate of nonfatal opioid overdose increased significantly in both the NIS and NEDS, in the NIS, the rate of injection drug use-related acute infection increased significantly, while the rate of the other outcomes did not change significantly. Significant variations were also identified; for example, in the NIS, the rate of nonfatal opioid overdose did not show significant change among females, non-Hispanic whites, and adults with higher socioeconomic status.

Our study is the most comprehensive to date in using nationally representative inpatient and ED data to assess trends in opioid-related events since 2016. Previous studies focused only on several years after 2016 (e.g., the study by Vivolo-Kantor et al. included 2016–2017 NEDS data),^41^ did not analyze specific opioid categories (e.g., the study by Fingar et al. grouped all categories as one type),^42^ or covered only a short period during the COVID-19 pandemic (e.g., the study by Holland et al. included data until October 10, 2020).^22^

We found that the rate of nonfatal opioid overdoses in the NIS markedly increased during the COVID-19 pandemic, reversing the previous decreasing trends between 2016 and 2019, which may reflect the negative impacts of the COVID-19 pandemic. Similarly, in the NEDS, we observed an increasing trend of nonfatal opioid overdoses during the COVID-19 pandemic, contrasting with the lack of statistical change between 2016 and 2019. For different opioid categories, although the rate of nonfatal synthetic opioid overdose-related ED visits and inpatient stays significantly increased in both periods, the COVID-19 pandemic appears to have exacerbated this increase. This underscores the potential negative impacts of public health crises on opioid-related outcomes and the importance of designing policies to mitigate the impact of future crises. Although the specific pathways through which the COVID-19 pandemic has negatively affected opioid-related outcomes are not well understood, previous studies have suggested several potential drivers, including social isolation and interpersonal conflict.^43^^,^^44^

Another important finding is the significant increase in the rate of injection drug-use related acute infection in the NIS between 2016 and 2019. In particular, there was a notable rise in the rate of phlebitis and infection arthritis, with rates increasing by more than 200%. Although this study could not explore the reason for this increase, potential explanations include insufficient access to safe injection equipment and supervised injection facilities, which was recommended by the CDC in a statement in November 2016.^45^ However, these findings should be interpreted with caution, as these conditions are not necessarily related to injection drug use. We should not overlook the potential for misclassification or missed diagnoses if individuals are not always coded with OUD.

The differences observed across various demographic characteristics underscore the ongoing health disparity issue in opioid management. While this concern is not new and is acknowledged in the 2022 CDC clinical practice guideline for prescribing opioids for pain, further implementation research is necessary to understand why certain subpopulations receive better management.^46^^,^^47^ Addressing this issue is particularly crucial in future crises, where resources may be limited.

Using inpatient and emergency department records would underestimate cases of opioid-related events, and the extent of underestimation may vary over time.^48^^,^^49^ This could introduce bias in the estimation of both prevalence and temporal changes. Future studies should investigate the underlying reasons for this and consider conducting quantitative bias analysis to adjust the estimates.

According to CFR–Code of Federal Regulations Title 21, Part 1306.07, which covers the administration or dispensing of narcotic drugs,^50^ patients are not typically hospitalized for “detoxification care.” However, our results showed that, among encounters receiving substance abuse treatment, the majority underwent detoxification services. Considering the measurement of detoxification services, the ICD-10 coding system only mentions detoxification from alcohol and/or drugs, which provides insufficient detail for a comprehensive definition of this term. Future studies should consider conducting a comprehensive survey on how detoxification services are coded to explore the reasons behind this inconsistency.

Variations in the geographic trends were observed. However, the “hospital region” variable in our data only includes four categories: Northeast, Midwest, South, and West. This classification lacks sufficient granularity to provide more actionable insights. Future studies based on State Inpatient Databases could strengthen this by offering more detailed geographic breakdowns.^51^

We agree with the reviewer that our methods and models should be validated in more recent data. HCUP recently released the 2022 data. Given the proximity of the submission deadline for the revised version, we were unable to perform this analysis.

This study has several limitations. First, all estimates are limited by the reliability of ICD-10-CM coding. Although imperfect, a previous validation study based on the US population found that ICD-10-CM diagnostic codes can be used to monitor opioid overdose rates (predictive value: 81%).^52^ Second, inpatient rehabilitation or detoxification care in our study does not reflect the situation in rehabilitation and long-term acute care hospitals, as the NIS excludes these hospital types.^53^ Third, results from our study did not indicate whether encouters were: prescribed opioids, used opioids illicitly, or treated for OUD, which should be a topic for further investigation in future comprehensive data-linkage studies.^54^ Fourth, analyses using hospitalizations/ED visits as the denominator may be subject to bias during the COVID-19 pandemic because of reduced access to hospitalizations/ED visits for non-COVID-19 conditions.^55^ When new data from after 2021 are available, more research is needed to verify/assess trends following the pandemic (i.e., when ED and hospitalizations practices returned to normal or towards normal). Fifth, analyses based on opioid categories do not account for the degree of use, necessitating a cautious approach to the interpretation of results, particularly regarding the risk of specific opioids.^56^ For example, if tramadol is consumed much more frequently than another opioid, it may appear in more opioid-related events, even though it is not necessarily associated with an increased risk compared to other opioids. Sixth, a protocol for a retrospective study is important as it provides a structured, transparent, and reproducible framework for conducting research.^57^ Although our study did not include a protocol, we acknowledge its importance and recommend that it be included in future studies.

The significant variability in opioid-related hospital utilization trends among U.S. adults underscores the need for careful consideration in the design of future policies, especially during crises. Management strategies should be tailored to specific subpopulations, opioid categories, and OUD-related clinical events to maximize success rates.

## Contributors

LC, RC, HZ, SF, and MLF generated the planning and designed the study. LC, RC, and BS developed the study methods. LC, ZC, and JD did the statistical analysis. LC, ZC, and JD drafted the manuscript. LC, ZC and JD accessed and verified the underlying data. All authors discussed results, commented on the manuscript, and critically revised the manuscript. Each author contributed important intellectual content during manuscript drafting or revision. SF and HZ are the study guarantors. The corresponding author attests that all listed authors meet authorship criteria and that no others meeting the criteria have been omitted.

## Data sharing statement

Access to data can be requested via application to the Healthcare Cost and Utilization Project (https://hcup-us.ahrq.gov).

## Declaration of interests

All authors have completed the ICMJE uniform disclosure form at www.icmje.org/disclosure-of-interest/ and declare: no support from any organisation for the submitted work; MLF provided consulting advice on the scientific advisory board for Novartis, no other relationships or activities that could appear to have influenced the submitted work.

## Appendix A Supplementary data

Translated abstracts
