# Supplementary material for: Variability in trends of opioid-related hospital utilization among U.S. Adults, 2016–2021 check
Source: eClinicalMedicine. 2025 Jul 12;86:103355. doi: 10.1016/j.eclinm.2025.103355 (PMC12280345; doi:10.1016/j.eclinm.2025.103355)
Supplement: Translated abstracts [file mmc1.docx]

The following translations in Chinese were submitted by the authors and we reproduce them as supplied. They have not been peer reviewed. Our editorial processes have only been applied to the original abstract in English, which should serve as reference for this manuscript

**背景**
理解阿片类药物相关医院使用趋势对于制定公共卫生政策至关重要；然而，现有研究在范围和方法上常存在局限性。本研究提供了2016年至2021年的全国估计数据，强调了不同阿片类药物类别和亚群体之间趋势的差异性。

**方法**
本研究采用重复横断面分析方法，利用国家住院样本（National Inpatient Sample，NIS）和全国急诊科样本（Nationwide Emergency Department Sample，NEDS）数据进行研究。分析分为两个时期：2016年至2019年，以及2019年至2021年（即COVID-19大流行期间）。研究结果包括阿片类药物相关诊断的发生率，以及三种类型的阿片使用障碍相关临床事件：非致命性阿片过量、注射相关急性感染和物质滥用治疗。进一步分析根据阿片类别（例如，海洛因和作为芬太尼替代指标的合成阿片类药物）进行分类，并基于预定义的人口学特征（包括年龄、性别、种族/族裔、社会经济地位和地理位置）进行亚组分析。

**发现**
在2016至2019年期间，在NIS中，阿片类药物相关诊断率显著下降（相对变化：-5.4%，95%置信区间：-9.4%至-1.3%），非致命性阿片过量（-18.4%，-21.7%至-15.0%）和物质滥用治疗（-25.1%，-45.9%至-4.3%）也有显著下降。然而，注射相关急性感染率显著增加（14.4%，7.3%至21.4%）。相比之下，NEDS中的这些结果率变化无显著性。值得注意的是，在NIS中，65至84岁成年人中，作为芬太尼替代指标的合成阿片类药物非致命性过量率增加了21.1%（11.6%至30.5%），海洛因相关不良事件或中毒率增加了51.8%（16.8%至86.8%）。在2019年至2021年期间，NIS和NEDS均显示非致命性阿片过量率显著上升（NIS：8.1%，3.5%至12.7%；NEDS：24.8%，11.5%至38.0%）。在NIS中，注射相关急性感染率也显著增加（相对增加：8.2%，1.2%至15.1%），而其他结果率变化无显著性。​同样值得注意的是，在NIS中，女性、非西班牙裔白人和高社会经济地位成年人群体中，非致命性阿片过量率未显示出显著变化。

**解释**美国成年人中阿片类药物相关医院使用趋势的显著差异，强调了在制定未来政策，特别是在危机时期，需要仔细考虑这些差异。管理策略应针对特定亚群体、阿片类药物类别及与阿片使用障碍相关的临床事件进行量身定制，从而最大限度地提高成功率。

**资助**
山东省泰山学者计划-攀登项目

**关键词**：阿片类药物；差异性；医院使用；COVID-19大流行；疼痛
