# Supplementary material for: Variability in trends of opioid-related hospital utilization among U.S. Adults, 2016–2021 check
Source: eClinicalMedicine. 2025 Jul 12;86:103355. doi: 10.1016/j.eclinm.2025.103355 (PMC12280345; doi:10.1016/j.eclinm.2025.103355)
Supplement: Supplementary Figs S1–S12 and Tables S1–S40 [file mmc2.docx]

**Supplementary Appendix**

Supplement to: Chen L, Chen Z, Ding J, et al. Variability in Trends of Opioid-Related Hospital Utilization Among U.S. Adults, 2016-2021.

This appendix has been provided by the authors to give readers additional information about the work.

**Appendix**: Variability in Trends of Opioid-Related Hospital Utilization Among U.S. Adults, 2016-2021.

Lingxiao Chen PhD, Zhuo Chen MB, Jiaming Ding MB, Roger Chou PhD, Claire E Ashton-James PhD, Baoyi Shi MSc, Stephanie Mathieson PhD, Maja R Radojčić PhD, David B Anderson PhD, Ruiyuan Zheng MB, Runhan Fu MB, Yujie Chen MSc, Lei Qi MD, Hengxing Zhou MD, Shiqing Feng MD, Manuela L Ferreira PhD

[Supplement Methods 6](#_Toc32522)

[Supplementary Table S1. Definitions for Opioid-Related Diagnoses 7](#_Toc21018)

[Supplementary Table S2. Opioid-Related Drugs and Chemicals 8](#_Toc8175)

[Supplementary Table S3. Definitions for Opioid Use Disorder-Related Clinical Events 11](#_Toc14351)

[Supplementary Table S4.1. Missing Data 13](#_Toc9926)

[Supplementary Table S4.2. Estimates after multiple imputation for Supplementary Table S11 14](#_Toc16784)

[Supplementary Table S4.3. Estimates after multiple imputation for Supplementary Table S12 17](#_Toc22482)

[Supplementary Table S5. Distribution of Hospitalizations and Emergency Department Visits and Opioid-Related Diagnoses](#_Toc20189)^[a](#_Toc20189)^ [and Opioid Use Disorder-Related Clinical Events](#_Toc20189)^[b](#_Toc20189)^ [in 2016-2021](#_Toc20189)^[c](#_Toc20189)^ [20](#_Toc20189)

[Supplementary Table S6. Demographic Distribution of Hospitalizations and Emergency Department Visits and Opioid-Related Diagnoses](#_Toc13112)^[a](#_Toc13112)^ [and Opioid Use Disorder-Related Clinical Events](#_Toc13112)^[b](#_Toc13112)^ [in 2021](#_Toc13112)^[c](#_Toc13112)^ [21](#_Toc13112)

[Supplementary Table S7. Opioid-Related Diagnoses](#_Toc1247)^[a](#_Toc1247)^ [(2016 to 2021) Overall and for Subtypes Involving Opioid Categories of Adverse Event or Poisoning in the National Inpatient Sample 25](#_Toc1247)

[Supplementary Table S8. Opioid-Related Diagnoses](#_Toc16944)^[a](#_Toc16944)^ [(2016 to 2021) Overall and for Subtypes Involving Opioid Categories of Adverse Event or Poisoning in the Nationwide Emergency Department Sample 27](#_Toc16944)

[Supplementary Table S9. Opioid Use Disorder-Related Clinical Events](#_Toc10271)^[a](#_Toc10271)^ [(2016 to 2021) Overall and for Subtypes in the National Inpatient Sample](#_Toc10271)^[b](#_Toc10271)^ [29](#_Toc10271)

[Supplementary Table S10. Opioid Use Disorder-Related Clinical Events](#_Toc16034)^[a](#_Toc16034)^ [(2016 to 2021) Overall and for Subtypes in the Nationwide Emergency Department Sample](#_Toc16034)^[b](#_Toc16034)^ [31](#_Toc16034)

[Supplementary Figure S1. Opioid-Related Diagnoses (2016 to 2021) in National Inpatient Sample for Subtypes by Age group (A), by Sex (B), by Race/ethnicity (C), by Zip code income quartile (D), by Primary expected payer (E) and by Patient location (F). 33](#_Toc31831)

[Supplementary Figure S2. Opioid-Related Diagnoses (2016 to 2021) in Nationwide Emergency Department Sample for Subtypes by Age group (A), by Sex (B), by Race/ethnicity (C), by Zip code income quartile (D), by Primary expected payer (E) and by Patient location (F). 34](#_Toc28909)

[Supplementary Figure S3. Opioid-Related Diagnoses (2016 to 2021) for Subtypes by Hospital region in National Inpatient Sample (A) and Nationwide Emergency Department Sample (B) 35](#_Toc13750)

[Supplementary Figure S4. Nonfatal Opioid Overdose (2016 to 2021) in National Inpatient Sample for Subtypes by Age group (A), by Sex (B), by Race/ethnicity (C), by Zip code income quartile (D), by Primary expected payer (E) and by Patient location (F). 36](#_Toc15531)

[Supplementary Figure S5. Injection Drug-Use Related Acute Infection (2016 to 2021) in National Inpatient Sample for Subtypes by Age group (A), by Sex (B), by Race/ethnicity (C), by Zip code income quartile (D), by Primary expected payer (E) and by Patient location (F). 37](#_Toc12554)

[Supplementary Figure S6. Substance Abuse Treatment (2016 to 2021) in National Inpatient Sample for Subtypes by Age group (A), by Sex (B), by Race/ethnicity (C), by Zip code income quartile (D), by Primary expected payer (E) and by Patient location (F). 38](#_Toc23714)

[Supplementary Figure S7. Nonfatal Opioid Overdose (2016 to 2021) in Nationwide Emergency Department Sample for Subtypes by Age group (A), by Sex (B), by Race/ethnicity (C), by Zip code income quartile (D), by Primary expected payer (E) and by Patient location (F). 39](#_Toc4715)

[Supplementary Figure S8. Injection Drug-Use Related Acute Infection (2016 to 2021) in Nationwide Emergency Department Sample for Subtypes by Age group (A), by Sex (B), by Race/ethnicity (C), by Zip code income quartile (D), by Primary expected payer (E) and by Patient location (F). 40](#_Toc3917)

[Supplementary Figure S9. Substance Abuse Treatment (2016 to 2021) in Nationwide Emergency Department Sample for Subtypes by Age group (A), by Sex (B), by Race/ethnicity (C), by Zip code income quartile (D), by Primary expected payer (E) and by Patient location (F). 41](#_Toc9763)

[Supplementary Figure S10. OUD-related clinical events for Subtypes by Hospital Region in National Inpatient Sample (A–C) and Nationwide Emergency Department Sample (D–F) 42](#_Toc16543)

[Supplementary Table S11. Opioid-Related Diagnoses](#_Toc32225)^[a](#_Toc32225)^ [(2016 to 2019 and 2020 to 2021) in National Inpatient Sample Overall and for Subtypes by Demographic Characteristics 43](#_Toc32225)

[Supplementary Table S12. Opioid-Related Diagnoses](#_Toc25326)^[a](#_Toc25326)^ [(2016 to 2019 and 2020 to 2021) in Nationwide Emergency Department Sample Overall and for Subtypes by Demographic Characteristics 47](#_Toc25326)

[Supplementary Table S13. Opioid-Related Abuse or Dependence](#_Toc21626)^[a](#_Toc21626)^ [(2016 to 2019 and 2020 to 2021) in National Inpatient Sample Overall and for Subtypes by Demographic Characteristics 51](#_Toc21626)

[Supplementary Table S14. Opioid-Related Abuse or Dependence](#_Toc30764)^[a](#_Toc30764)^ [(2016 to 2019 and 2020 to 2021) in Nationwide Emergency Department Sample Overall and for Subtypes by Demographic Characteristics 55](#_Toc30764)

[Supplementary Table S15. Opioid-Related Adverse Event or Poisoning](#_Toc21703)^[a](#_Toc21703)^ [(2016 to 2019 and 2020 to 2021) in National Inpatient Sample Overall and for Subtypes by Demographic Characteristics 59](#_Toc21703)

[Supplementary Table S16. Opioid-Related Adverse Event or Poisoning](#_Toc5581)^[a](#_Toc5581)^ [(2016 to 2019 and 2020 to 2021) in Nationwide Emergency Department Sample Overall and for Subtypes by Demographic Characteristics 63](#_Toc5581)

[Supplementary Table S17. Synthetic opioids as a proxy for fentanyl Related Adverse Event or Poisoning](#_Toc1125)^[a](#_Toc1125)^ [(2016 to 2019 and 2020 to 2021) in National Inpatient Sample Overall and for Subtypes by Demographic Characteristics 67](#_Toc1125)

[Supplementary Table S18. Synthetic opioids as a proxy for fentanyl Related Adverse Event or Poisoning](#_Toc16661)^[a](#_Toc16661)^ [(2016 to 2019 and 2020 to 2021) in Nationwide Emergency Department Sample Overall and for Subtypes by Demographic Characteristics 71](#_Toc16661)

[Supplementary Table S19. Prescription natural/semisynthetic opioids as a proxy for opioid pain medications Related Adverse Event or Poisoning](#_Toc22866)^[a](#_Toc22866)^ [(2016 to 2019 and 2020 to 2021) in National Inpatient Sample Overall and for Subtypes by Demographic Characteristics 75](#_Toc22866)

[Supplementary Table S20. Prescription natural/semisynthetic opioids as a proxy for opioid pain medications Related Adverse Event or Poisoning](#_Toc11936)^[a](#_Toc11936)^ [(2016 to 2019 and 2020 to 2021) in Nationwide Emergency Department Sample Overall and for Subtypes by Demographic Characteristics 79](#_Toc11936)

[Supplementary Table S21. Heroin-Related Adverse Event or Poisoning](#_Toc31184)^[a](#_Toc31184)^ [(2016 to 2019 and 2020 to 2021) in National Inpatient Sample Overall and for Subtypes by Demographic Characteristics 83](#_Toc31184)

[Supplementary Table S22. Heroin-Related Adverse Event or Poisoning](#_Toc1893)^[a](#_Toc1893)^ [(2016 to 2019 and 2020 to 2021) in Nationwide Emergency Department Sample Overall and for Subtypes by Demographic Characteristics 87](#_Toc1893)

[Supplementary Table S23. Opium-Related Adverse Event or Poisoning](#_Toc24555)^[a](#_Toc24555)^ [(2016 to 2019 and 2020 to 2021) in National Inpatient Sample Overall and for Subtypes by Demographic Characteristics 91](#_Toc24555)

[Supplementary Table S24. Opium-Related Adverse Event or Poisoning](#_Toc9852)^[a](#_Toc9852)^ [(2016 to 2019 and 2020 to 2021) in Nationwide Emergency Department Sample and for Subtypes by Demographic Characteristics 95](#_Toc9852)

[Supplementary Table S25. Methadone-Related Adverse Event or Poisoning](#_Toc9872)^[a](#_Toc9872)^ [(2016 to 2019 and 2020 to 2021) in National Inpatient Sample Overall and for Subtypes by Demographic Characteristics 99](#_Toc9872)

[Supplementary Table S26. Methadone-Related Adverse Event or Poisoninga (2016 to 2019 and 2020 to 2021) in Nationwide Emergency Department Sample Overall and for Subtypes by Demographic Characteristics 103](#_Toc7793)

[Supplementary Table S27. Other Opioids-Related Adverse Event or Poisoning](#_Toc13349)^[a](#_Toc13349)^ [(2016 to 2019 and 2020 to 2021) in National Inpatient Sample Overall and for Subtypes by Demographic Characteristics 107](#_Toc13349)

[Supplementary Table S28. Other Opioids-Related Adverse Event or Poisoning](#_Toc2548)^[a](#_Toc2548)^ [(2016 to 2019 and 2020 to 2021) in Nationwide Emergency Department Sample Overall and for Subtypes by Demographic Characteristics 111](#_Toc2548)

[Supplementary Table S29. Nonfatal Opioid Overdose](#_Toc22260)^[a](#_Toc22260)^ [(2016 to 2019 and 2020 to 2021) in National Inpatient Sample Overall and for Subtypes by Demographic Characteristics 115](#_Toc22260)

[Supplementary Table S30. Injection Drug-Use Related Acute Infection](#_Toc2840)^[a](#_Toc2840)^ [(2016 to 2019 and 2020 to 2021) in National Inpatient Sample Overall and for Subtypes by Demographic Characteristics 119](#_Toc2840)

[Supplementary Table S31. Substance abuse Treatment](#_Toc18929)^[a](#_Toc18929)^ [(2016 to 2019 and 2020 to 2021) in National Inpatient Sample Overall and for Subtypes by Demographic Characteristics 123](#_Toc18929)

[Supplementary Table S32. Nonfatal Opioid Overdose](#_Toc13701)^[a](#_Toc13701)^ [(2016 to 2019 and 2020 to 2021) in Nationwide Emergency Department Sample Overall and for Subtypes by Demographic Characteristics 127](#_Toc13701)

[Supplementary Table S33. Injection Drug-Use Related Acute Infection](#_Toc3229)^[a](#_Toc3229)^ [(2016 to 2019 and 2020 to 2021) in Nationwide Emergency Department Sample Overall and for Subtypes by Demographic Characteristics 131](#_Toc3229)

[Supplementary Table S34. Substance abuse Treatment](#_Toc17226)^[a](#_Toc17226)^ [(2016 to 2019 and 2020 to 2021) in Nationwide Emergency Department Sample Overall and for Subtypes by Demographic Characteristics 135](#_Toc17226)

[Supplementary Table S35. Opioid-Related Primary Diagnoses](#_Toc3861)^[a](#_Toc3861)^ [(2016 to 2019 and 2020 to 2021) in National Inpatient Sample Overall and for Subtypes Involving Opioid Categories of Adverse Event or Poisoning](#_Toc3861)^[b](#_Toc3861)^ [139](#_Toc3861)

[Supplementary Table S36. Opioid-Related Primary Diagnoses](#_Toc12594)^[a](#_Toc12594)^ [(2016 to 2019 and 2020 to 2021) in Nationwide Emergency Department Sample Overall and for Subtypes Involving Opioid Categories of Adverse Event or Poisoning](#_Toc12594)^[b](#_Toc12594)^ [141](#_Toc12594)

[Supplementary Table S37. Percentage of Synthetic Opioids in Three Specific Classifications in 2021 143](#_Toc31632)

[Supplementary Table S38. Percentage of Nonfatal Synthetic Opioid Overdose in Three Specific Classifications in 2021 144](#_Toc1735)

[Supplementary Table S39. Synthetic Opioids-Related Diagnoses (2020 Q4 to 2021 Q4) in National Inpatient Sample and Nationwide Emergency Department Sample for Subtypes 145](#_Toc28890)

[Supplementary Table S40. Nonfatal Synthetic Opioid Overdose (2020 Q4 to 2021 Q4) in National Inpatient Sample and Nationwide Emergency Department Sample for Subtypes 146](#_Toc28523)

[Supplementary Figure S11 Temporal trend changes in annual percent change (APC) for opioid related diagnoses in NIS (A), opioid related adverse event or poisoning in NIS (B), opioid related diagnoses in NEDS (C), and opioid related adverse event or poisoning in NEDS (D) from 2016 to 2021 based on the Joinpoint regression model. *p < 0.05. NIS, Nationwide Inpatient Sample; NEDS, Nationwide Emergency Department Sample. 147](#_Toc23946)

[Supplementary Figure S12 Temporal trend changes in annual percent change (APC) for nonfatal opioid overdose and subtypes in NIS (A), injection drug-use related acute infection and subtypes in NIS (B), substance abuse treatment and subtypes in NIS (C), nonfatal opioid overdose and subtypes in NEDS (D), injection drug-use related acute infection and subtypes in NEDS (E), and substance abuse treatment and subtypes in NEDS (F) from 2016 to 2021 based on the Joinpoint regression model. *p < 0.05. NIS, Nationwide Inpatient Sample; NEDS, Nationwide Emergency Department Sample. 148](#_Toc13609)

**Supplement Methods**

*Outcomes*

The rationale behind the decision not to use the number of the general population as the denominator was to reduce the impact of the COVID-19 pandemic on health service use.

*Additional analyses*

First, these demographic characteristics were identified based on the statement of the CDC guideline and the data availability of the NIS and NEDS. We followed the statement from the 2022 CDC guideline as this version reflected the most recent understanding. In the 2022 CDC guideline, it mentioned several specific, long-standing health disparities: age, sex, race, socioeconomic status, and geographic location. Thus, we included age, sex, race/ethnicity, zip code income quartile, primary expected payer, patient location, and hospital region.

Second, previous studies mentioned that it may miscategorize the true reason for hospitalization if cases were identified by any diagnosis field rather than by the primary diagnosis field only as the secondary diagnosis field may only indicate a history of the disease (Health Aff (Millwood) 2017; 36(12): 2054-61). On the other hand, it may lose a lot of opioid-related diagnoses if cases were identified by the primary diagnosis field only considering the high burden of opioid-related complications (clinicians may code the complication in the primary diagnosis field). Thus, the main analyses were based on any diagnosis field and one additional analysis was performed on the primary diagnosis field.

Third, since 2020 Q4, synthetic opioids can be classified into three subcategories: fentanyl and its analogs, tramadol, and other synthetic opioids (previously, synthetic opioids could not be divided into subcategories). Given the focus on synthetic opioids in this area, we performed an additional analysis, as this is the first time we could explore these subcategories in nationally representative data about inpatient stays and ED visits.

Fourth, Joinpoint analysis was conducted in both the NIS and NEDS for: 1) opioid-related diagnoses and all subcategories; and 2) opioid use disorder-related clinical events and all subtypes. The number of joinpoints was 0.

Fifth, missing data were handled using multiple imputation in both the NIS and NEDS for subgroup analyses of opioid-related diagnoses by predefined demographic characteristics, assuming the data were missing at random. Ten imputed datasets were generated, and all available variables were included in the imputation process. Given that the data were weighted, analyses were conducted using the mi and svy commands in STATA.

**Supplementary Table S1. Definitions for Opioid-Related Diagnoses**

| **Classification** | | **ICD-10-CM** |
| --- | --- | --- |
| **Opioid-Related Diagnoses** | **F11 series: Abuse or dependence** | F11 (except F11.11, F11.21, and F11.91) |
|  | **T40 series: Adverse event or poisoning** |  |
|  | Opium | T400X1, T400X2, T400X3, T400X4, T400X5 |
|  | Heroin | T401X1, T401X2, T401X3, T401X4 |
|  | Prescription natural/ semisynthetic opioids ^a^ | T402X1, T402X2, T402X3, T402X4, T402X5 |
|  | Methadone | T403X1, T403X2, T403X3, T403X4, T403X5 |
|  | Synthetic opioids ^b^ (through 2020 Q3) | T404X1, T404X2, T404X3, T404X4, T404X5 |
|  | Fentanyl and its analogs (beginning 2020 Q4) | T40411, T40412, T40413, T40414, T40415 |
|  | Tramadol (beginning 2020 Q4) | T40421, T40422, T40423, T40424, T40425 |
|  | Other synthetic opioids (beginning 2020 Q4) | T40491, T40492, T40493, T40494, T40495 |
|  | Other opioids (unspecified and other narcotics) | T40601, T40602, T40603, T40604, T40605, T40691, T40692, T40693, T40694, T40695 |

Adapted from: HCUP Fast Stats. Healthcare Cost and Utilization Project (HCUP). June 2023. Agency for Healthcare Research and Qualt, Rockville, MD. <https://datatools.ahrq.gov/hcup-fast-stats>

^a^ Using Prescription Natural/Semisynthetic opioids as a proxy for opioid pain meds provides a clinical lens to observe real-world patterns of use.

^b^ Using synthetic opioids as a proxy for fentanyl provides a clinical lens to observe real-world patterns of use.

**Supplementary Table S2. Opioid-Related Drugs and Chemicals**

| **Classification** | **ICD-10-CM** | **Substance** |
| --- | --- | --- |
| **Opium** | T400X1, T400X2, T400X3, T400X4, T400X5 | Laudanum |
|  |  | Opium alkaloids (total)- standardized powdered/tincture (camphorated) |
|  |  | Pantopon |
|  |  | Papaveretum |
|  |  | Paregoric |
| **Heroin** | T401X1, T401X2, T401X3, T401X4 | Acetomorphine |
|  |  | Diacetylmorphine |
|  |  | Diamorphine |
|  |  | Heroin |
| **Prescription natural/ semisynthetic opioids** | T402X1, T402X2, T402X3, T402X4, T402X5 | 14-hydroxydihydro-morphinone |
|  |  | Acemorphan |
|  |  | Acetorphine |
|  |  | Acetyldihydrocodeine |
|  |  | Acetyldihydrocodeinone |
|  |  | Alvodine |
|  |  | Antitussive NEC codeine mixture/opiate |
|  |  | Benzomorphan |
|  |  | Benzyl morphine |
|  |  | Blue velvet |
|  |  | Cliradon |
|  |  | Codeine |
|  |  | Cough mixture (syrup) containing opiates |
|  |  | Demerol |
|  |  | Desocodeine |
|  |  | Desomorphine |
|  |  | Dextrorphan |
|  |  | Difencloxazine |
|  |  | Dihydrocodeine |
|  |  | Dihydrocodeinone |
|  |  | Dihydrohydroxycodeinone |
|  |  | Dihydrohydroxymorphinone |
|  |  | Dihydroisocodeine |
|  |  | Dihydromorphine |
|  |  | Dihydromorphinone |
|  |  | Dihydroxycodeinone |
|  |  | Dilaudid |
|  |  | Dimethyl meperidine |
|  |  | Dionin |
|  |  | Drocode |
|  |  | Dromoran |
|  |  | Ethyl morphine |
|  |  | Ethylmorphine |
|  |  | Etorphine |
|  |  | Eucodal |
|  |  | Heptalgin |
|  |  | Hycodan |
|  |  | Hydrocodone |
|  |  | Hydromorphinol |
|  |  | Hydromorphinone |
|  |  | Hydromorphone |
|  |  | Hydroxydihydrocodeinone |
|  |  | Leritine |
|  |  | Levodromoran |
|  |  | Methyl dihydromorphinone/morphine NEC |
|  |  | Methylmorphine |
|  |  | Metopon |
|  |  | Morfin |
|  |  | Morphine |
|  |  | Morpholinylethylmorphine |
|  |  | Nicomorphine |
|  |  | Nisentil |
|  |  | Normorphine |
|  |  | Numorphan |
|  |  | Opioid NEC |
|  |  | Oxycodone |
|  |  | Oxymorphone |
|  |  | Palfium |
|  |  | Paracodin |
|  |  | Parzone |
|  |  | Percodan |
|  |  | Peronine |
|  |  | Phenadoxone |
|  |  | Phenomorphan |
|  |  | Piminodine |
|  |  | Pipadone |
|  |  | Prinadol |
|  |  | Promedol |
|  |  | Racemoramide |
|  |  | Racemorphan |
|  |  | Thebaine |
| **Methadone** | T403X1, T403X2, T403X3, T403X4, T403X5 | Amidone |
|  |  | Dolophine |
|  |  | Levo-iso-methadone |
|  |  | Methadone |
|  |  | Physeptone |
| **Synthetic opioids (fentanyl and its analogs）** | T40411, T40412, T40413, T40414, T40415 | Alfentanil |
|  |  | Fentanyl (analogs) |
|  |  | Sufentanil |
| **Synthetic opioids (tramadol)** | T40421, T40422, T40423, T40424, T40425 | Tramadol |
| **Synthetic opioids (other synthetic opioids)** | T40491, T40492, T40493, T40494, T40495 | Alphaprodine |
|  |  | Anileridine |
|  |  | Bezitramide |
|  |  | Buprenorphine |
|  |  | Butorphanol |
|  |  | Dextromoramide |
|  |  | Dextropropoxyphene |
|  |  | Dipipanone |
|  |  | Eptazocine |
|  |  | Ethoheptazine |
|  |  | Isonipecaine |
|  |  | Ketobemidone |
|  |  | Levopropoxyphene |
|  |  | Levorphanol |
|  |  | Meperidine |
|  |  | Nalbuphine |
|  |  | Narcotic (drug) synthetic |
|  |  | Pentazocine |
|  |  | Pethidine |
|  |  | Phenazocine |
|  |  | Phenoperidine |
|  |  | Piritramide |
|  |  | Profadol |
|  |  | Propoxyphene |
|  |  | Tilidine |
| **Other opioids (unspecified and other narcotics)** | T40601, T40602, T40603, T40604, T40605, T40691, T40692, T40693, T40694, T40695 | Analgesic narcotic NEC/combination/obstetric |
|  |  | Narcotic (drug) analgesic NEC |
|  |  | Opiate NEC |
|  |  | Narcotic (drug) specified NEC |

Sourced from ICD–10–CM Table of Drugs and Chemicals https://www.cdc.gov/nchs/icd/icd10cm_browsertool.htm

**Supplementary Table S3. Definitions for Opioid Use Disorder-Related Clinical Events**

| **Opioid Use Disorder-related clinical events** | **ICD-10** |
| --- | --- |
| **A. Nonfatal opioid overdose** | T40.0X1A, T40.0X1D, T40.0X1S, T40.0X2A, T40.0X2D, T40.0X2S, T40.0X3A, T40.0X3D, T40.0X3S, T40.0X4A, T40.0X4D, T40.0X4S, T40.1X1A, T40.1X1D, T40.1X1S, T40.1X2A, T40.1X2D, T40.1X2S, T40.1X3A, T40.1X3D, T40.1X3S, T40.1X4A, T40.1X4D, T40.1X4S, T40.2X1A, T40.2X1D, T40.2X1S, T40.2X2A,  T40.2X2D, T40.2X2S, T40.2X3A, T40.2X3D, T40.2X3S, T40.2X4A, T40.2X4D, T40.2X4S, T40.3X1A, T40.3X1D, T40.3X1S, T40.3X2A, T40.3X2D, T40.3X2S, T40.3X3A, T40.3X3D, T40.3X3S, T40.3X4A, T40.3X4D, T40.3X4S, T40.4X1A, T40.4X1D, T40.4X1S, T40.4X2A, T40.4X2D, T40.4X2S, T40.4X3A, T40.4X3D,  T40.4X3S, T40.4X4A, T40.4X4D, T40.4X4S |
| **B. Injection drug-use related acute infection** |  |
| **Phlebitis** | I80.1-I80.9 |
| **Abscess and/or cellulitis** | G06.1, G06.2, L02.01, L02.11, L02.12, L02.13, L02.211, L02.212, L02.213, L02.214, L02.215, L02.216, L02.219, L02.221, L02.222, L02.223, L02.224, L02.225, L02.226, L02.229, L02.231, L02.232, L02.233, L02.234, L02.235, L02.236, L02.239, L02.31, L02.32, L02.33, L02.411, L02.412, L02.413, L02.414, L02.415, L02.416, L02.419, L02.421, L02.422, L02.423, L02.424, L02.425, L02.426, L02.429, L02.431, L02.432, L02.433, L02.434, L02.435, L02.436, L02.439, L02.91, L03.011, L03.012, L03.019, L03.021, L03.022, L03.029, L03.031, L03.032, L03.039, L03.041, L03.042, L03.049, L03.111, L03.112, L03.113, L03.114, L03.115, L03.116, L03.119, L03.121, L03.122, L03.123, L03.124, L03.125, L03.126, L03.129, L03.212, L03.221, L03.222, L03.311, L03.312, L03.313, L03.314, L03.315, L03.316, L03.317, L03.319, L03.321, L03.322, L03.323, L03.324, L03.325, L03.327, L03.329, L03.811, L03.818, L03.891, L03.898, L03.390, L03.391, L98.3 |
| **Infectious endocarditis** | I33.0, I33.9, I38, I39 |
| **Infectious arthritis** | M00 |
| **Sepsis or bacteremia** | A02.1, A20.0, A20.7, A21.7, A22.7, A24.1, A26.7, A28.2, A32.7, A39.1, A39.2, A39.3, A39.4, A40., A41., A42.7, A48.3, A49.9, A54.8, B00.7, B37.6, B37.7, B49, O75.3, O85, R65.0, R65.1, R57.2, R78.81 |
| **C. Substance abuse treatment** |  |
| **>1 of Following Revenue ICD-10 Procedure Codes** | HZ2: detoxification services |
|  | HZ3: individual counseling |
|  | HZ4: group counseling |
|  | HZ5: individual psychotherapy |
|  | HZ6: family counseling |
|  | HZ8: medication management |
|  | HZ9: pharmacotherapy |
| **PLUS > 1 of Following ICD-10 Diagnosis Codes** | F11 |

Adapted from one previous study. Barnett ML, Meara E, Lewinson T, Hardy B, Chyn D, Onsando M, Huskamp HA, Mehrotra A, Morden NE. Racial Inequality in Receipt of Medications for Opioid Use Disorder. N Engl J Med. 2023 May 11;388(19):1779-1789.

Note: Nonfatal opioid overdose was defined by any diagnosis in inpatient stays and ED visits for non-fatal opioid overdose.

Injection drug use-related acute infection were defined by any diagnosis of one of the infections in inpatient stays and ED visits,  in addition to any diagnosis of opioid use disorder (ICD-10-CM code used: F11.1).

**Supplementary Table S4.1. Missing Data**

| **Characteristics** | **2016** | **2017** | **2018** | **2019** | **2020** | **2021** |
| --- | --- | --- | --- | --- | --- | --- |
|  | **Unweighted No. (weighted %)** | | | | | |
| 1. **National Inpatient Sample** | | | | | | |
| **Overall** | 13238 (6.8) | 11667 (5.9) | 9350 (5.0) | 9042 (4.9) | 9872 (5.9) | 10468 (6.3) |
| **Sex** | 83 (0.04) | NR^a^ | 11 (0.006) | 15 (0.008) | 18 (0.01) | 45 (0.03) |
| **Race/ethnicity** | 8330 (4.3) | 6519 (3.3) | 3845 (2.0) | 3307 (1.8) | 3453 (2.1) | 3928 (2.4) |
| **Zip code income quartile** | 4848 (2.5) | 4890 (2.5) | 5360 (2.8) | 5601 (3.1) | 6299 (3.7) | 6379 (3.8) |
| **Primary expected payer** | 307 (0.2) | 533 (0.3) | 269 (0.1) | 238 (0.1) | 240 (0.1) | 263 (0.2) |
| **Patient Location** | 2275 (1.2) | 2370 (1.2) | 2838 (1.5) | 3258 (1.8) | 4309 (2.6) | 4480 (2.7) |
| 1. **Nationwide Emergency Department Sample** | | | | | | |
| **Overall** | 9859 (3.0) | 10927 (3.1) | 14519 (3.5) | 16144 (4.7) | 19234 (5.6) | 20552 (6.1) |
| **Sex** | 98 (0.03) | 14 (0.004) | 22 (0.005) | 33 (0.009) | 33 (0.01) | 52 (0.02) |
| **Race/ethnicity** | NA^b^ | NA^b^ | NA^b^ | 3698 (1.2) | 5448 (1.7) | 6912 (2.0) |
| **Zip code income quartile** | 9392 (2.9) | 10,142 (2.8) | 14,272 (3.5) | 12,126 (3.4) | 13,348 (3.8) | 13,380 (3.9) |
| **Primary expected payer** | 383 (0.1) | 806 (0.3) | 236 (0.07) | 581 (0.2) | 716 (0.3) | 612 (0.2) |
| **Patient Location** | 4808 (1.5) | 5483 (1.5) | 9526 (2.2) | 7760 (2.1) | 9327 (2.6) | 9526 (2.8) |

^a^ NR, Not Reportable. Suppressed to protect confidentiality, ≤10 cases.

^b^ NA, Not Available. Due to the NEDS survey design, the Race/ethnicity classification was not available until 2019 and therefore could not present estimates.

**Supplementary Table S4.2.** **Estimates after multiple imputation for Supplementary Table S11**

| **Characteristics** | **Between 2016 and 2019** | | | **During the COVID-19 pandemic in 2020 and 2021** | | |
| --- | --- | --- | --- | --- | --- | --- |
|  | **2019 Rate,**  **per 10,000** | **Absolute difference**  **from 2016-2019^b^** | **Change**  **from 2016-2019, %** | **2021 Rate,**  **per 10,000** | **Absolute difference**  **from 2019-2021^c^** | **Change**  **from 2019-2021, %** |
| **National Inpatient Sample** | | | | | | |
| **Sex** |  |  |  |  |  |  |
| **Male** | 348.0  (336.3 to 359.8) | -22.1  (-39.7 to -4.5) | -6.0  (-10.7 to -1.2) | 344.4  (333.0 to 355.9) | -3.6  (-20.0 to 12.8) | -1.0  (-5.7 to 3.7) |
| **Female** | 269.2  (261.8 to 276.6) | -15.1  (-26.1 to -4.1) | -5.3  (-9.2 to -1.5) | 253.6  (246.2 to 260.9) | -15.6  (-26.0 to -5.2) | -5.8  (-9.7 to -1.9) |
| **Race/ethnicity^d^** |  |  |  |  |  |  |
| **Non-Hispanic White** | 325.7  (316.7 to 334.7) | -21.7  (-35.2 to -8.2) | -6.2  (-10.1 to -2.4) | 316.0  (306.9 to 325.0) | -9.7  (-22.5 to 3.1) | -3.0  (-6.9 to 0.9) |
| **Non-Hispanic Black** | 286.7  (269.5 to 303.8) | -11.1  (-37.2 to 15.0) | -3.7  (-12.5 to 5.0) | 283.8  (267.8 to 299.8) | -2.9  (-26.3 to 20.5) | -1.0  (-9.2 to 7.2) |
| **Hispanic** | 243.3  (225.3 to 261.3) | 0.2  (-24.4 to 24.8) | 0.1  (-10.0 to 10.2) | 233.9  (217.6 to 250.3) | -9.4  (-33.7 to 14.9) | -3.9  (-13.8 to 6.1) |
| **Non-Hispanic Asian or Pacific Islander** | 99.4  (90.4 to 108.5) | -12.3  (-26.7 to 2.1) | -11.0  (-23.9 to 1.8) | 95.5  (87.4 to 103.5) | -3.9  (-16.0 to 8.2) | -3.9  (-16.1 to 8.2) |
| **Native American** | 431.3  (381.7 to 480.8) | 16.5  (-54.5 to 87.5) | 4.0  (-13.1 to 21.1) | 410.4  (359 to 461.9) | -20.9  (-92.3 to 50.5) | -4.8  (-21.4 to 11.7) |
| **Other** | 258.5  (231.3 to 285.7) | -5.0  (-43.0 to 33.0) | -1.9  (-16.3 to 12.5) | 261.2  (241.2 to 281.2) | 2.7  (-31.1 to 36.5) | 1.0  (-12.0 to 14.1) |
| **Zip code income quartile^e^** |  |  |  |  |  |  |
| **1 (lowest)** | 337.8  (323.7 to 351.9) | -14.8  (-35.2 to 5.6) | -4.2  (-10.0 to 1.6) | 336.4  (322.3 to 350.5) | -1.4  (-21.4 to 18.6) | -0.4  (-6.3 to 5.5) |
| **2** | 298.7  (289.5 to 307.9) | -18.6  (-32.6 to -4.6) | -5.9  (-10.3 to -1.4) | 288.2  (278.8 to 297.7) | -10.5  (-23.7 to 2.7) | -3.5  (-7.9 to 0.9) |
| **3** | 294.1  (284.7 to 303.6) | -14.4  (-28.1 to -0.7) | -4.7  (-9.1 to -0.2) | 280.3  (271.2 to 289.5) | -13.8  (-27.0 to -0.6) | -4.7  (-9.2 to -0.2) |
| **4 (highest)** | 265.3  (253.6 to 277.0) | -21.7  (-38.9 to -4.5) | -7.6  (-13.6 to -1.6) | 250.6  (239.4 to 261.8) | -14.7  (-30.9 to 1.5) | -5.5  (-11.7 to 0.6) |
| **Primary expected payer^f^** |  |  |  |  |  |  |
| **Medicare** | 232.1  (225.3 to 238.9) | -8.8  (-18.5 to 0.9) | -3.7  (-7.7 to 0.4) | 221.3  (214.4 to 228.3) | -10.8  (-20.5 to -1.1) | -4.7  (-8.8 to -0.5) |
| **Medicaid** | 618.5  (591.6 to 645.4) | -4.7  (-43.0 to 33.6) | -0.8  (-6.9 to 5.4) | 602.1  (576.9 to 627.4) | -16.4  (-53.3 to 20.5) | -2.7  (-8.6 to 3.3) |
| **Private insurance** | 190.3  (183.2 to 197.4) | -30.6  (-42.7 to -18.5) | -13.9  (-19.3 to -8.4) | 176.1  (168.8 to 183.4) | -14.2  (-24.3 to -4.1) | -7.5  (-12.8 to -2.1) |
| **Self-pay** | 441.6  (417.7 to 465.5) | -47.7  (-81.4 to -14.0) | -9.7  (-16.6 to -2.9) | 431.3  (406.2 to 456.3) | -10.3  (-45.0 to 24.4) | -2.3  (-10.2 to 5.5) |
| **No charge** | 607.6  (471.1 to 744.2) | -13.7  (-245.1 to 217.7) | -2.2  (-39.5 to 35.0) | 579.9  (462.7 to 697.1) | -27.7  (-207.7 to 152.3) | -4.6  (-34.2 to 25.1) |
| **Other** | 296.1  (273.3 to 318.9) | -43.6  (-84.6 to -2.6) | -12.8  (-24.9 to -0.8) | 259.6  (240.3 to 278.9) | -36.5  (-66.3 to -6.7) | -12.3  (-22.4 to -2.3) |
| **Patient Location^g^** |  |  |  |  |  |  |
| **"Central" counties of metro areas of >=1 million population** | 344.7  (326.5 to 362.8) | -9.5  (-35.8 to 16.8) | -2.7  (-10.1 to 4.8) | 330.7  (313.4 to 348) | -14.0  (-39.1 to 11.1) | -4.1  (-11.3 to 3.2) |
| **"Fringe" counties of metro areas of >=1 million population** | 297.9  (284.3 to 311.6) | -23.6  (-44 to -3.2) | -7.3  (-13.7 to -1.0) | 282.6  (269.2 to 296) | -15.3  (-34.6 to 4.0) | -5.1  (-11.6 to 1.3) |
| **Counties in metro areas of 250,000-999,999 population** | 308.0  (293 to 322.9) | -9.3  (-30.5 to 11.9) | -2.9  (-9.6 to 3.8) | 302.3  (287.4 to 317.2) | -5.7  (-26.8 to 15.4) | -1.9  (-8.7 to 5.0) |
| **Counties in metro areas of 50,000-249,999 population** | 282.8  (263.8 to 301.9) | -23.4  (-52.1 to 5.3) | -7.6  (-17.0 to 1.7) | 275.3  (255.3 to 295.3) | -7.5  (-35.1 to 20.1) | -2.7  (-12.4 to 7.1) |
| **Micropolitan counties** | 253.6  (241.6 to 265.5) | -30.7  (-49.8 to -11.6) | -10.8  (-17.5 to -4.1) | 250.8  (239.2 to 262.4) | -2.8  (-19.4 to 13.8) | -1.1  (-7.7 to 5.5) |
| **Not metropolitan or micropolitan counties** | 224.2  (202.7 to 245.6) | -24.0  (-54.2 to 6.2) | -9.7  (-21.8 to 2.5) | 227.3  (204.0 to 250.5) | 3.1  (-28.5 to 34.7) | 1.4  (-12.7 to 15.5) |

**Supplementary Table S4.3. Estimates after multiple imputation for Supplementary Table S12**

| **Characteristics** | **Between 2016 and 2019** | | | **During the COVID-19 pandemic in 2020 and 2021** | | |
| --- | --- | --- | --- | --- | --- | --- |
|  | **2019 Rate,**  **per 10,000** | **Absolute difference**  **from 2016-2019^b^** | **Change**  **from 2016-2019, %** | **2021 Rate,**  **per 10,000** | **Absolute difference**  **from 2019-2021^c^** | **Change**  **from 2019-2021, %** |
| **Nationwide Emergency Department Sample** | | | | | | |
| **Sex** |  |  |  |  |  |  |
| **Male** | 152.4  (141.4 to 163.3) | -3.8  (-18.1 to 10.5) | -2.4  (-11.6 to 6.7) | 169.4  (154.1 to 184.7) | 17.0  (-1.8 to 35.8) | 11.2  (-1.2 to 23.5) |
| **Female** | 94.8  (89.5 to 100.1) | -6.7  (-14.3 to 0.9) | -6.6  (-14.1 to 0.9) | 98.3  (92.0 to 104.6) | 3.5  (-4.7 to 11.7) | 3.7  (-5.0 to 12.3) |
| **Race/ethnicity^d^** |  |  |  |  |  |  |
| **Non-Hispanic White** | 141.5  (133.4 to 149.6) | NA | NA | 151.9  (141.7 to 162.2) | 10.4  (-2.6 to 23.4) | 7.3  (-1.8 to 16.5) |
| **Non-Hispanic Black** | 92.1  (74.9 to 109.3) | NA | NA | 105.8  (89.2 to 122.4) | 13.7  (-10.1 to 37.5) | 14.9  (-11.0 to 40.8) |
| **Hispanic** | 85.9  (75.1 to 96.7) | NA | NA | 94.1  (81.5 to 106.8) | 8.2  (-8.3 to 24.7) | 9.5  (-9.7 to 28.8) |
| **Non-Hispanic Asian or Pacific Islander** | 51.8  (31.5 to 72.0) | NA | NA | 45.9  (40.3 to 51.5) | -5.9  (-26.9 to 15.1) | -11.4  (-51.9 to 29.1) |
| **Native American** | 145.0  (116.4 to 173.6) | NA | NA | 210.4  (148.2 to 272.6) | 65.4  (-3.0 to 133.8) | 45.1  (-2.1 to 92.3) |
| **Other** | 95.1  (83.2 to 107.1) | NA | NA | 121.8  (105.0 to 138.5) | 26.7  (6.2 to 47.2) | 28.1  (6.5 to 49.6) |
| **Zip code income quartile^e^** |  |  |  |  |  |  |
| **1 (lowest)** | 121.4  (110.0 to 132.8) | 1.1  (-13.9 to 16.1) | 0.9  (-11.6 to 13.4) | 135.4  (124.6 to 146.2) | 14.0  (-1.7 to 29.7) | 11.5  (-1.4 to 24.4) |
| **2** | 114.6  (107.0 to 122.3) | -4.8  (-15.3 to 5.7) | -4.0  (-12.8 to 4.8) | 124.2  (115.2 to 133.2) | 9.6  (-2.2 to 21.4) | 8.4  (-1.9 to 18.7) |
| **3** | 122.0  (114.3 to 129.7) | -8.9  (-20.0 to 2.2) | -6.8  (-15.3 to 1.7) | 134.9  (114.8 to 154.9) | 12.9  (-8.5 to 34.3) | 10.6  (-7.0 to 28.1) |
| **4 (highest)** | 121.7  (113.0 to 130.4) | -14.2  (-28.8 to 0.4) | -10.4  (-21.2 to 0.3) | 123.1  (110.4 to 135.8) | 1.4  (-14.0 to 16.8) | 1.2  (-11.5 to 13.8) |
| **Primary expected payer^f^** |  |  |  |  |  |  |
| **Medicare** | 107.2  (101.5 to 112.8) | -8.2  (-16.5 to 0.1) | -7.1  (-14.3 to 0.1) | 111.2  (104.0 to 118.3) | 4.0  (-5.1 to 13.1) | 3.7  (-4.7 to 12.2) |
| **Medicaid** | 208.9  (189.8 to 227.9) | 13.6  (-10.6 to 37.8) | 7.0  (-5.4 to 19.3) | 221.7  (202.0 to 241.3) | 12.8  (-14.5 to 40.1) | 6.1  (-6.9 to 19.2) |
| **Private insurance** | 61.2  (57.2 to 65.2) | -15.3  (-22.0 to -8.6) | -20.0  (-28.8 to -11.2) | 64.6  (58.9 to 70.3) | 3.4  (-3.5 to 10.3) | 5.6  (-5.7 to 16.8) |
| **Self-pay** | 124.8  (111.8 to 137.8) | -1.3  (-18.3 to 15.7) | -1.0  (-14.5 to 12.4) | 158.8  (125.3 to 192.4) | 34.0  (-1.9 to 69.9) | 27.2  (-1.5 to 56.0) |
| **No charge** | 157.5  (122.4 to 192.5) | -26.6  (-119.7 to 66.5) | -14.4  (-65.0 to 36.1) | 210.4  (149.9 to 270.8) | 52.9  (-16.9 to 122.7) | 33.6  (-10.7 to 77.9) |
| **Other** | 88.7  (73.7 to 103.6) | -1.9  (-20.3 to 16.5) | -2.1  (-22.4 to 18.2) | 88.8  (78.3 to 99.3) | 0.1  (-18.1 to 18.3) | 0.1  (-20.4 to 20.6) |
| **Patient Location^g^** |  |  |  |  |  |  |
| **"Central" counties of metro areas of >=1 million population** | 137.2  (120.0 to 154.3) | 3.9  (-18.1 to 25.9) | 2.9  (-13.6 to 19.4) | 154.0  (129.8 to 178.2) | 16.8  (-12.7 to 46.3) | 12.2  (-9.3 to 33.8) |
| **"Fringe" counties of metro areas of >=1 million population** | 122.1  (111.1 to 133.2) | -21.2  (-39.4 to -3.0) | -14.8  (-27.5 to -2.1) | 127  (112.9 to 141.2) | 4.9  (-13.0 to 22.8) | 4.0  (-10.6 to 18.7) |
| **Counties in metro areas of 250,000-999,999 population** | 129.4  (114.4 to 144.3) | -3.6  (-24.1 to 16.9) | -2.7  (-18.1 to 12.7) | 131.6  (118.7 to 144.4) | 2.2  (-17.4 to 21.8) | 1.7  (-13.4 to 16.8) |
| **Counties in metro areas of 50,000-249,999 population** | 100.0  (90.8 to 109.2) | -2.4  (-18.3 to 13.5) | -2.3  (-17.9 to 13.2) | 134.8  (115 to 154.7) | 34.8  (13.0 to 56.6) | 34.8  (13.0 to 56.6) |
| **Micropolitan counties** | 92.8  (84.2 to 101.4) | -10.3  (-22.4 to 1.8) | -10.0  (-21.7 to 1.7) | 95.3  (85.9 to 104.6) | 2.5  (-10.1 to 15.1) | 2.7  (-10.9 to 16.3) |
| **Not metropolitan or micropolitan counties** | 75.3  (67.8 to 82.8) | -3.8  (-14.9 to 7.3) | -4.8  (-18.8 to 9.2) | 81.8  (73.6 to 90.0) | 6.5  (-4.6 to 17.6) | 8.6  (-6.1 to 23.4) |

**Supplementary Table S5. Distribution of Hospitalizations and Emergency Department Visits and Opioid-Related Diagnoses^a^ and Opioid Use Disorder-Related Clinical Events^b^ in 2016-2021^c^**

| **Characteristic** | **National Inpatient Sample** | **Nationwide Emergency Department Sample** |
| --- | --- | --- |
|  | **Unweighted No. (weighted %)** | |
| **Opioid-related diagnoses** | 1,096,854 (3.1) | 2,025,557 (1.3) |
| **Opioid Use Disorder-related clinical events** |  |  |
| **Nonfatal opioid overdose** | 107,362 (0.3) | 403,643 (0.3) |
| **Injection drug-use related acute infection** | 55,530 (0.2) | 89,660 (0.1) |
| **Substance abuse treatment** | 98,577 (0.3) | 61,821 (0.04) |
| **All hospitalizations/** **emergency department visits ^d^** | 35,439,371 | 158,636,176 |

^a^ Cases of opioid-related diagnoses included two subtypes: abuse or dependence, and adverse event or poisoning (classified by opioid category, including: synthetic opioids as a proxy for fentanyl, prescription natural/semisynthetic opioids as a proxy for opioid pain medications, heroin, opium, methadone, and other opioids).

^b^ Cases of Opioid Use Disorder-related clinical events represented as nonfatal opioid overdose (classified by opioid category, including: synthetic opioids as a proxy for fentanyl, prescription natural/semisynthetic opioids as a proxy for opioid pain medications, heroin, opium, methadone, and other opioids), injection drug use-related acute infection, and substance abuse treatment.

^c^ Weights provided by the Healthcare Cost and Utilization Project, National Inpatient Sample/Nationwide Emergency Department Sample were used to ensure that the estimates were nationally representative, and weights and design variables were included to obtain unbiased estimates and standard errors.

^d^ Hospitalizations Source: Agency for Healthcare Research and Quality, Healthcare Cost and Utilization Project, National Inpatient Sample. Emergency department visits Source: Agency for Healthcare Research and Quality, Healthcare Cost and Utilization Project, Nationwide Emergency Department Sample.

**Supplementary Table S6. Demographic Distribution of Hospitalizations and Emergency Department Visits and Opioid-Related Diagnoses^a^ and Opioid Use Disorder-Related Clinical Events^b^ in 2021^c^**

| **Characteristic** | **Opioid-related diagnoses** | **Nonfatal opioid overdose** | **Injection drug-use related acute infection** | **Substance abuse treatment** | **All hospitalizations/** **emergency department visits** |
| --- | --- | --- | --- | --- | --- |
| **A. National Inpatient Sample** | | | | | |
| **Unweighted No. (weighted %)** | 166,922 (2.9) | 16,663 (0.3) | 9810 (0.2) | 12,973 (0.2) | 5,688,355 |
| **Zip code income quartile^e^** |  |  |  |  |  |
| **1 (lowest)** | 35.1 | 35.4 | 38.6 | 34.9 | 30.5 |
| **2** | 25.0 | 24.7 | 23.7 | 22.4 | 25.5 |
| **3** | 22.7 | 21.0 | 18.2 | 20.6 | 23.8 |
| **4 (highest)** | 17.2 | 14.4 | 12.2 | 22.0 | 20.2 |
| **Primary expected payer^f^** |  |  |  |  |  |
| **Medicare** | 34.7 | 28.6 | 14.5 | 13.3 | 46.0 |
| **Medicaid** | 39.6 | 38.2 | 57.6 | 58.9 | 19.3 |
| **Private insurance** | 16.2 | 16.7 | 10.0 | 18.5 | 27.0 |
| **Self-pay** | 6.0 | 11.9 | 13.9 | 5.5 | 4.1 |
| **No charge** | 0.6 | 1.3 | 1.1 | 1.4 | 0.3 |
| **Other** | 2.9 | 3.1 | 2.5 | 2.4 | 3.3 |
| **Patient location^g^** |  |  |  |  |  |
| **"Central" counties of metro areas of >=1 million population** | 32.5 | 31.6 | 30.7 | 38.2 | 28.9 |
| **"Fringe" counties of metro areas of >=1 million population** | 24.0 | 22.8 | 20.7 | 28.0 | 24.9 |
| **Counties in metro areas of 250,000-999,999 population** | 21.8 | 22.1 | 22.7 | 16.3 | 21.1 |
| **Counties in metro areas of 50,000-249,999 population** | 8.8 | 8.3 | 8.4 | 8.2 | 9.4 |
| **Micropolitan counties** | 7.7 | 7.2 | 7.5 | 5.9 | 9.0 |
| **Not metropolitan or micropolitan counties** | 5.2 | 4.5 | 3.9 | 3.4 | 6.7 |
| **Hospital region** |  |  |  |  |  |
| **Northeast** | 23.0 | 19.1 | 20.1 | 45.2 | 18.3 |
| **Midwest** | 19.8 | 20.1 | 17.5 | 15.2 | 21.8 |
| **South** | 35.6 | 40.4 | 37.1 | 28.5 | 40.4 |
| **West** | 21.6 | 20.3 | 25.4 | 11.1 | 19.4 |
| **B. Nationwide Emergency Department Sample** | | | | | |
| **Unweighted No. (weighted %)** | 324,483(1.3) | 72850 (0.3) | 13912 (0.06) | 8686 (0.04) | 25,213,348 |
| **Zip code income quartile^e^** |  |  |  |  |  |
| **1 (lowest)** | 35.8 | 37.0 | 36.2 | 32.0 | 34.4 |
| **2** | 25.1 | 24.7 | 23.2 | 22.4 | 26.4 |
| **3** | 22.9 | 20.3 | 21.1 | 22.0 | 22.0 |
| **4 (highest)** | 16.2 | 13.9 | 12.6 | 19.7 | 17.2 |
| **Primary expected payer^f^** |  |  |  |  |  |
| **Medicare** | 25.0 | 15.4 | 13.9 | 15.0 | 29.3 |
| **Medicaid** | 43.1 | 42.7 | 54.0 | 54.4 | 25.3 |
| **Private insurance** | 14.5 | 15.1 | 9.9 | 20.5 | 29.4 |
| **Self-pay** | 13.7 | 22.3 | 18.3 | 7.2 | 11.2 |
| **No charge** | 0.7 | 1.3 | 1.1 | 0.9 | 0.4 |
| **Other** | 3.0 | 3.2 | 2.7 | 2.1 | 4.4 |
| **Patient location^g^** |  |  |  |  |  |
| **"Central" counties of metro areas of >=1 million population** | 35.1 | 31.7 | 33.0 | 36.6 | 29.7 |
| **"Fringe" counties of metro areas of >=1 million population** | 19.8 | 20.6 | 17.3 | 24.4 | 20.3 |
| **Counties in metro areas of 250,000-999,999 population** | 22.0 | 23.3 | 22.7 | 14.0 | 21.9 |
| **Counties in metro areas of 50,000-249,999 population** | 10.7 | 9.3 | 10.2 | 13.9 | 10.3 |
| **Micropolitan counties** | 7.9 | 8.2 | 7.7 | 5.0 | 10.8 |
| **Not metropolitan or micropolitan counties** | 4.5 | 3.9 | 3.6 | 2.9 | 7.1 |
| **Hospital region** |  |  |  |  |  |
| **Northeast** | 27.2 | 21.4 | 25.0 | 49.5 | 18.7 |
| **Midwest** | 18.5 | 23.4 | 14.4 | 16.2 | 22.1 |
| **South** | 33.2 | 37.9 | 34.3 | 25.6 | 39.4 |
| **West** | 21.1 | 17.3 | 26.3 | 8.8 | 19.7 |

^a^ Cases of opioid-related diagnoses included two subtypes: abuse or dependence, and adverse event or poisoning (classified by opioid category, including: synthetic opioids as a proxy for fentanyl, prescription natural/semisynthetic opioids as a proxy for opioid pain medications, heroin, opium, methadone, and other opioids).

^b^ Cases of Opioid Use Disorder-related clinical events represented as nonfatal opioid overdose (classified by opioid category, including: synthetic opioids as a proxy for fentanyl, prescription natural/semisynthetic opioids as a proxy for opioid pain medications, heroin, opium, methadone, and other opioids), injection drug use-related acute infection, and substance abuse treatment.

^c^ Weights provided by the Healthcare Cost and Utilization Project, National Inpatient Sample/Nationwide Emergency Department Sample were used to ensure that the estimates were nationally representative, and weights and design variables were included to obtain unbiased estimates and standard errors.

^d^ Hospitalizations Source: Agency for Healthcare Research and Quality, Healthcare Cost and Utilization Project, National Inpatient Sample. Emergency department visits Source: Agency for Healthcare Research and Quality, Healthcare Cost and Utilization Project, Nationwide Emergency Department Sample.

^e^ Median household income of residents in the patient’s zip code was updated annually; 2019 range for quartile 1 was less than $48,000; quartile 2, $48,000 to $60,999; quartile 3, $61,000 to $81,999; and quartile 4, at least $82,000; 2021 range for quartile 1 was less than $52,000; quartile 2, $52,000 to $65,999; quartile 3, $66,000 to $87,999; and quartile 4, at least $88,000.

^f^ “Medicare” includes both fee-for-service and managed care Medicare patients. “Medicaid” includes both fee-for-service and managed care Medicaid patients. “Private insurance” includes Blue Cross, commercial carriers, and private HMOs and PPOs. “Other” includes Worker's Compensation, CHAMPUS (Civilian Health and Medical Program of the Uniformed Services), CHAMPVA (Civilian Health and Medical Program of the Department of Veteran's Affairs), Title V, and other government programs.

^g^ “Patient location” is a six-category urban-rural classification scheme for U.S. counties developed by the National Center for Health Statistics (NCHS) especially for use in health care research. The classification emphasizes urban distinctions and is unique in differentiating between central and fringe counties of large metropolitan areas. Smaller metropolitan counties are subdivided by population. Non-metropolitan counties are divided simply into micropolitan and non-core categories.

**Supplementary Table S7. Opioid-Related Diagnoses^a^ (2016 to 2021) Overall and for Subtypes Involving Opioid Categories of Adverse Event or Poisoning in the National Inpatient Sample**

| **Characteristics** | **Opioid-related diagnoses, 2016-2021** | | | | | |
| --- | --- | --- | --- | --- | --- | --- |
|  | **2016 Rate,**  **per 10,000** | **2017 Rate,**  **per 10,000** | **2018 Rate,**  **per 10,000** | **2019 Rate,**  **per 10,000** | **2020 Rate,**  **per 10,000** | **2021 Rate,**  **per 10,000** |
| **Overall** | 320.2  (310.7 to 329.7) | 323.7  (314.0 to 333.4) | 312.0  (302.6 to 321.4) | 303.1  (294.3 to 311.9) | 303.1  (294.3 to 311.9) | 293.4  (284.7 to 302.2) |
| **Abuse or dependence** | 238.9  (229.6 to 248.2) | 241.6  (232.1 to 251.0) | 235.5  (226.3 to 244.7) | 229.1  (220.6 to 237.5) | 233.9  (225.4 to 242.4) | 226.8  (218.5 to 235.1) |
| **Adverse event or poisoning** | 99.6  (97.4 to 101.8) | 100.6  (98.4 to 102.8) | 93.7  (91.6 to 95.8) | 90.7  (88.5 to 92.9) | 86.9  (84.6 to 89.1) | 83.6  (81.3 to 85.9) |
| **Synthetic opioids as**  **a proxy for fentanyl** | 6.5  (6.2 to 6.7) | 6.8  (6.6 to 7.1) | 6.7  (6.4 to 7.0) | 7.1  (6.8 to 7.4) | 7.7  (7.4 to 8.1) | 9.2  (8.7 to 9.6) |
| **Prescription natural/semisynthetic opioids as a proxy for opioid pain medications** | 49.2  (47.7 to 50.6) | 52.4  (50.8 to 54.0) | 51.1  (49.5 to 52.7) | 50.8  (49.0 to 52.6) | 49.1  (47.2 to 51.0) | 48.5  (46.6 to 50.4) |
| **Heroin** | 8.3  (7.9 to 8.8) | 9.1  (8.6 to 9.6) | 8.0  (7.6 to 8.4) | 7.5  (7.2 to 7.9) | 8.0  (7.5 to 8.4) | 6.7  (6.3 to 7.1) |
| **Opium** | 1.1  (1.0 to 1.2) | 0.9  (0.8 to 1.0) | 0.7  (0.6 to 0.8) | 0.5  (0.4 to 0.6) | 0.4  (0.4 to 0.5) | 0.4  (0.3 to 0.5) |
| **Methadone** | 2.8  (2.6 to 3.0) | 2.6  (2.5 to 2.8) | 2.3  (2.1 to 2.4) | 2.0  (1.9 to 2.2) | 2.1  (2.0 to 2.3) | 1.8  (1.6 to 1.9) |
| **Other opioids** | 33.9  (32.9 to 34.8) | 31.1  (30.2 to 31.9) | 27.1  (26.3 to 27.9) | 24.8  (24.1 to 25.5) | 21.8  (21.1 to 22.5) | 19.5  (18.9 to 20.1) |

^a^ Cases of opioid-related diagnoses were identified following the definition proposed by the Healthcare Cost and Utilization Project, including two subtypes: abuse or dependence, and adverse event or poisoning (classified by opioid category, including: synthetic opioids as a proxy for fentanyl, prescription natural/semisynthetic opioids as a proxy for opioid pain medications, heroin, opium, methadone, and other opioids).

**Supplementary Table S8. Opioid-Related Diagnoses^a^ (2016 to 2021) Overall and for Subtypes Involving Opioid Categories of Adverse Event or Poisoning in the Nationwide Emergency Department Sample**

| **Characteristics** | **Opioid-related diagnoses, 2016-2021** | | | | | |
| --- | --- | --- | --- | --- | --- | --- |
|  | **2016 Rate,**  **per 10,000** | **2017 Rate,**  **per 10,000** | **2018 Rate,**  **per 10,000** | **2019 Rate,**  **per 10,000** | **2020 Rate,**  **per 10,000** | **2021 Rate,**  **per 10,000** |
| **Overall** | 124.9  (117.9 to 131.8) | 131.7  (124.2 to 139.3) | 128.5  (120.2 to 136.8) | 119.8  (112.2 to 127.4) | 132.6  (124.8 to 140.3) | 130.2  (120.1 to 140.4) |
| **Abuse or dependence** | 92.4  (86.4 to 98.3) | 96.8  (89.9 to 103.6) | 96.6  (89.1 to 104.2) | 88.4  (82.0 to 94.9) | 97.0  (90.1 to 103.8) | 95.5  (86.1 to 104.8) |
| **Adverse event or poisoning** | 40.5  (38.0 to 43.0) | 42.9  (40.7 to 45.1) | 39.2  (37.2 to 41.2) | 37.8  (35.6 to 40.1) | 42.9  (40.8 to 44.9) | 41.7  (39.5 to 43.9) |
| **Synthetic opioids as**  **a proxy for fentanyl** | 2.7  (2.6 to 2.9) | 3.0  (2.8 to 3.2) | 3.0  (2.8 to 3.2) | 3.2  (3.0 to 3.5) | 4.3  (3.9 to 4.7) | 5.4  (4.9 to 5.8) |
| **Prescription natural/semisynthetic opioids as a proxy for opioid pain medications** | 15.2  (14.5 to 15.9) | 16.9  (16.1 to 17.7) | 15.3  (14.6 to 16.0) | 15.6  (14.7 to 16.5) | 18.0  (17.0 to 18.9) | 17.5  (16.6 to 18.4) |
| **Heroin** | 12.7  (10.8 to 14.6) | 13.1  (11.6 to 14.6) | 12.0  (10.8 to 13.2) | 10.7  (9.5 to 11.8) | 11.1  (10.1 to 12.1) | 9.8  (8.5 to 11.1) |
| **Opium** | 0.5  (0.4 to 0.6) | 0.4  (0.4 to 0.5) | 0.3  (0.2 to 0.3) | 0.3  (0.2 to 0.3) | 0.3  (0.2 to 0.3) | 0.2  (0.2 to 0.3) |
| **Methadone** | 1.0  (0.9 to 1.1) | 1.0  (0.9 to 1.1) | 0.9  (0.8 to 1.0) | 0.8  (0.7 to 0.9) | 0.9  (0.8 to 1.0) | 0.7  (0.7 to 0.8) |
| **Other opioids** | 9.1  (8.6 to 9.6) | 9.2  (8.7 to 9.8) | 8.4  (7.9 to 8.9) | 8.0  (7.3 to 8.7) | 9.2  (8.7 to 9.8) | 9.0  (8.4 to 9.5) |

^a^ Cases of opioid-related diagnoses were identified following the definition proposed by the Healthcare Cost and Utilization Project, including two subtypes: abuse or dependence, and adverse event or poisoning (classified by opioid category, including: synthetic opioids as a proxy for fentanyl, prescription natural/semisynthetic opioids as a proxy for opioid pain medications, heroin, opium, methadone, and other opioids).

**Supplementary Table S9. Opioid Use Disorder-Related Clinical Events^a^ (2016 to 2021) Overall and for Subtypes in the National Inpatient Sample^b^**

| **OUD-related clinical events** | **2016** | **2017** | **2018** | **2019** | **2020** | **2021** |
| --- | --- | --- | --- | --- | --- | --- |
|  | **Rate, per 10,000** | **Rate, per 10,000** | **Rate, per 10,000** | **Rate, per 10,000** | **Rate, per 10,000** | **Rate, per 10,000** |
| **A. Nonfatal opioid overdose** | 33.2  (32.4 to 34.1) | 32.7  (31.8 to 33.5) | 28.5  (27.8 to 29.3) | 27.1  (26.3 to 27.9) | 29.6  (28.7 to 30.5) | 29.3  (28.3 to 30.2) |
| **Synthetic opioids as**  **a proxy for fentanyl** | 2.7  (2.5 to 2.8) | 2.4  (2.3 to 2.5) | 2.3  (2.1 to 2.4) | 3.3  (3.1 to 3.5) | 4.4  (4.1 to 4.8) | 6.1  (5.7 to 6.5) |
| **Prescription natural/semisynthetic opioids as a proxy for opioid pain medications** | 12.9  (12.5 to 13.3) | 13.2  (12.8 to 13.6) | 11.8  (11.5 to 12.2) | 11.0  (10.7 to 11.4) | 12.1  (11.7 to 12.4) | 12.2  (11.8 to 12.6) |
| **Heroin** | 8.3  (7.9 to 8.8) | 8.8  (8.4 to 9.3) | 7.7  (7.3 to 8.1) | 7.5  (7.2 to 7.9) | 7.6  (7.2 to 8.0) | 6.7  (6.3 to 7.1) |
| **Opium** | 0.3  (0.3 to 0.4) | 0.3  (0.2 to 0.3) | 0.2  (0.2 to 0.2) | 0.2  (0.1 to 0.2) | 0.1  (0.1 to 0.2) | 0.1  (0.1 to 0.2) |
| **Methadone** | 1.7  (1.6 to 1.8) | 1.6  (1.5 to 1.7) | 1.3  (1.2 to 1.4) | 1.3  (1.2 to 1.4) | 1.3  (1.2 to 1.5) | 1.2  (1.1 to 1.3) |
| **Other opioids** | 7.6  (7.3 to 7.9) | 6.7  (6.5 to 7.0) | 5.5  (5.3 to 5.8) | 5.0  (4.7 to 5.2) | 5.1  (4.8 to 5.3) | 4.6  (4.4 to 4.9) |
| **B. Injection drug-use related acute infection** | 13.9  (13.3 to 14.6) | 14.4  (13.7 to 15) | 16.1  (15.4 to 16.8) | 15.9  (15.2 to 16.6) | 16.6  (15.9 to 17.4) | 17.2  (16.4 to 18.0) |
| **Phlebitis** | 0.1  (0.1 to 0.1) | 0.1  (0.1 to 0.1) | 0.4  (0.3 to 0.4) | 0.3  (0.3 to 0.4) | 0.3  (0.3 to 0.4) | 0.3  (0.3 to 0.4) |
| **Abscess and/or cellulitis** | 8.2  (7.8 to 8.7) | 9.0  (8.5 to 9.4) | 7.2  (6.8 to 7.5) | 8.8  (8.4 to 9.3) | 7.2  (6.9 to 7.6) | 9.2  (8.7 to 9.7) |
| **Infectious endocarditis** | 1.1  (1.0 to 1.2) | 1.2  (1.1 to 1.4) | 1.8  (1.6 to 1.9) | 1.7  (1.5 to 1.8) | 1.7  (1.5 to 1.8) | 1.7  (1.5 to 1.8) |
| **Infectious arthritis** | 0.2  (0.2 to 0.3) | 0.3  (0.3 to 0.3) | 0.6  (0.5 to 0.7) | 0.8  (0.7 to 0.9) | 0.7  (0.6 to 0.8) | 0.8  (0.7 to 0.9) |
| **Sepsis or bacteremia** | 7.2  (6.8 to 7.6) | 8.3  (7.9 to 8.7) | 9.3  (8.8 to 9.7) | 9.5  (9.0 to 9.9) | 10.9  (10.3 to 11.4) | 10.8  (10.2 to 11.3) |
| **C. Substance abuse treatment** | 34.4  (28.9 to 40.0) | 32.2  (26.9 to 37.4) | 29.0  (23.9 to 34.1) | 25.8  (21.2 to 30.3) | 23.2  (19.4 to 27.0) | 22.8  (19.1 to 26.6) |
| **Detoxification services** | 31.1  (26.0 to 36.1) | 28.6  (23.9 to 33.4) | 25.0  (20.6 to 29.4) | 21.6  (17.7 to 25.5) | 19.1  (15.8 to 22.4) | 18.9  (15.6 to 22.1) |
| **Individual counseling** | 3.3  (2.1 to 4.6) | 3.5  (2.3 to 4.8) | 2.9  (1.9 to 4.0) | 2.6  (1.7 to 3.6) | 2.5  (1.5 to 3.4) | 2.3  (1.3 to 3.3) |
| **Group counseling** | 4.0  (2.7 to 5.4) | 4.5  (3.1 to 6.0) | 4.3  (2.9 to 5.6) | 3.8  (2.6 to 5.0) | 3.8  (2.7 to 4.9) | 3.9  (2.6 to 5.2) |
| **Individual psychotherapy** | 0.5  (0.2 to 0.8) | 0.7  (0.2 to 1.2) | 0.8  (0.2 to 1.3) | 0.6  (0.2 to 1.0) | 0.6  (0.2 to 0.9) | 0.5  (0.2 to 0.8) |
| **Family counseling** | 0.1  (0.01 to 0.2) | 0.1  (0.02 to 0.2) | 0.1  (0.001 to 0.2) | 0.1  (-0.01 to 0.2) | 0.1  (-0.01 to 0.3) | 0.1  (-0.01 to 0.2) |
| **Medication management** | 2.2  (1.1 to 3.2) | 2.3  (1.1 to 3.4) | 1.9  (0.9 to 3.0) | 2.1  (1.0 to 3.2) | 2.3  (1.2 to 3.4) | 1.9  (0.9 to 2.9) |
| **Pharmacotherapy** | 1.5  (0.8 to 2.2) | 1.9  (1.1 to 2.7) | 2.0  (1.0 to 3.0) | 2.0  (1.0 to 2.9) | 1.9  (1.0 to 2.7) | 1.7  (1.1 to 2.3) |

^a^ Cases of Opioid Use Disorder-related clinical events represented as nonfatal opioid overdose, injection drug use-related acute infection, and substance abuse treatment.

^b^ Weights provided by the Healthcare Cost and Utilization Project, National Inpatient Sample was used to ensure that the estimates were nationally representative, and weights and design variables were included to obtain unbiased estimates and standard errors.

**Supplementary Table S10. Opioid Use Disorder-Related Clinical Events^a^ (2016 to 2021) Overall and for Subtypes in the Nationwide Emergency Department Sample^b^**

| **OUD-related clinical events** | **2016** | **2017** | **2018** | **2019** | **2020** | **2021** |
| --- | --- | --- | --- | --- | --- | --- |
|  | **Rate, per 10,000** | **Rate, per 10,000** | **Rate, per 10,000** | **Rate, per 10,000** | **Rate, per 10,000** | **Rate, per 10,000** |
| **A. Nonfatal opioid overdose** | 24.8  (22.5 to 27.1) | 25.4  (23.4 to 27.4) | 23.5  (21.8 to 25.2) | 23.0  (20.8 to 25.1) | 27.9  (26.1 to 29.8) | 28.7  (26.6 to 30.9) |
| **Synthetic opioids as**  **a proxy for fentanyl** | 1.4  (1.3 to 1.5) | 1.5  (1.4 to 1.6) | 1.6  (1.5 to 1.8) | 1.9  (1.7 to 2.1) | 3.1  (2.7 to 3.5) | 4.3  (3.9 to 4.8) |
| **Prescription natural/semisynthetic opioids as a proxy for opioid pain medications** | 6.2  (5.9 to 6.5) | 6.3  (6.0 to 6.6) | 5.8  (5.5 to 6.1) | 6.3  (5.6 to 6.9) | 8.2  (7.7 to 8.7) | 8.7  (8.0 to 9.4) |
| **Heroin** | 12.7  (10.8 to 14.6) | 13.1  (11.6 to 14.6) | 12.0  (10.8 to 13.2) | 10.7  (9.5 to 11.8) | 11.1  (10.1 to 12.1) | 9.8  (8.5 to 11.1) |
| **Opium** | 0.2  (0.2 to 0.3) | 0.2  (0.2 to 0.2) | 0.1  (0.1 to 0.1) | 0.1  (0.1 to 0.1) | 0.1  (0.09 to 0.1) | 0.1  (0.09 to 0.2) |
| **Methadone** | 0.8  (0.7 to 0.8) | 0.7  (0.6 to 0.8) | 0.6  (0.6 to 0.7) | 0.5  (0.5 to 0.6) | 0.6  (0.6 to 0.7) | 0.5  (0.5 to 0.6) |
| **Other opioids** | 4.0  (3.7 to 4.3) | 4.1  (3.8 to 4.5) | 3.8  (3.5 to 4.1) | 4.0  (3.4 to 4.7) | 5.5  (5.0 to 6.0) | 6.0  (5.5 to 6.5) |
| **B. Injection drug-use related acute infection** | 5.0  (4.5 to 5.5) | 5.6  (5.0 to 6.1) | 5.8  (5.3 to 6.3) | 5.5  (5.0 to 6.0) | 6.0  (5.5 to 6.5) | 5.5  (5.0 to 6.0) |
| **Phlebitis** | 0.1  (0.1 to 0.1) | 0.1  (0.1 to 0.2) | 0.1  (0.1 to 0.1) | 0.1  (0.1 to 0.1) | 0.1  (0.1 to 0.1) | 0.1  (0.09 to 0.1) |
| **Abscess and/or cellulitis** | 3.6  (3.2 to 4.0) | 3.9  (3.5 to 4.3) | 3.9  (3.6 to 4.3) | 3.7  (3.3 to 4.0) | 3.7  (3.4 to 4.1) | 3.4  (3.0 to 3.8) |
| **Infectious endocarditis** | 0.3  (0.3 to 0.4) | 0.4  (0.3 to 0.4) | 0.4  (0.3 to 0.5) | 0.4  (0.3 to 0.4) | 0.4  (0.4 to 0.5) | 0.3  (0.3 to 0.4) |
| **Infectious arthritis** | 0.1  (0.09 to 0.1) | 0.1  (0.1 to 0.2) | 0.2  (0.2 to 0.2) | 0.2  (0.1 to 0.2) | 0.2  (0.2 to 0.2) | 0.2  (0.1 to 0.2) |
| **Sepsis or bacteremia** | 1.7  (1.5 to 1.9) | 2.2  (1.9 to 2.4) | 2.4  (2.2 to 2.6) | 2.4  (2.2 to 2.7) | 2.9  (2.6 to 3.1) | 2.7  (2.5 to 3.0) |
| **C. Substance abuse treatment** | 4.1  (2.9 to 5.4) | 5.2  (3.3 to 7.1) | 3.6  (2.3 to 5.0) | 3.4  (2.2 to 4.5) | 3.9  (2.6 to 5.3) | 3.6  (2.5 to 4.8) |
| **Detoxification services** | 3.9  (2.8 to 5.1) | 4.8  (3.0 to 6.6) | 3.4  (2.1 to 4.7) | 2.9  (1.9 to 4.0) | 3.4  (2.2 to 4.7) | 3.1  (2.1 to 4.1) |
| **Individual counseling** | 0.2  (-0.02 to 0.5) | 0.2  (-0.06 to 0.5) | 0.05  (0.02 to 0.08) | 0.1  (-0.1 to 0.4) | 0.2  (-0.1 to 0.5) | 0.3  (0.01 to 0.6) |
| **Group counseling** | 0.3  (-0.1 to 0.8) | 0.4  (-0.01 to 0.9) | 0.3  (-0.1 to 0.7) | 0.3  (-0.1 to 0.6) | 0.6  (0.1 to 1.2) | 0.4  (0.03 to 0.8) |
| **Individual psychotherapy** | 0.05  (-0.005 to 0.1) | 0.04  (0.003 to 0.08) | 0.04  (0.002 to 0.07) | 0.2  (-0.2 to 0.6) | 0.2  (-0.1 to 0.5) | 0.06  (-0.01 to 0.1) |
| **Family counseling** | NR^c^ | NR^c^ | NR^c^ | NR^c^ | NR^c^ | NR^c^ |
| **Medication management** | 0.2  (0.03 to 0.4) | 0.3  (0.1 to 0.5) | 0.2  (0.05 to 0.4) | 0.7  (0.02 to 1.3) | 0.5  (-0.1 to 1.1) | 0.2  (0.06 to 0.3) |
| **Pharmacotherapy** | 0.2  (0.06 to 0.2) | 0.6  (-0.08 to 1.2) | 0.2  (0.08 to 0.4) | 0.5  (-0.1 to 1.1) | 0.6  (-0.1 to 1.3) | 0.4  (-0.02 to 0.8) |

^a^ Cases of Opioid Use Disorder-related clinical events represented as nonfatal opioid overdose, injection drug use-related acute infection, and substance abuse treatment.

^b^ Weights provided by the Healthcare Cost and Utilization Project, Nationwide Emergency Department Sample were used to ensure that the estimates were nationally representative, and weights and design variables were included to obtain unbiased estimates and standard errors.

^c^ NR, Not Reportable. Suppressed to protect confidentiality, ≤10 cases.

**Supplementary Figure S1. Opioid-Related Diagnoses (2016 to 2021) in National Inpatient Sample for Subtypes by Age group (A), by Sex (B), by Race/ethnicity (C), by Zip code income quartile (D), by Primary expected payer (E) and by Patient location (F).**


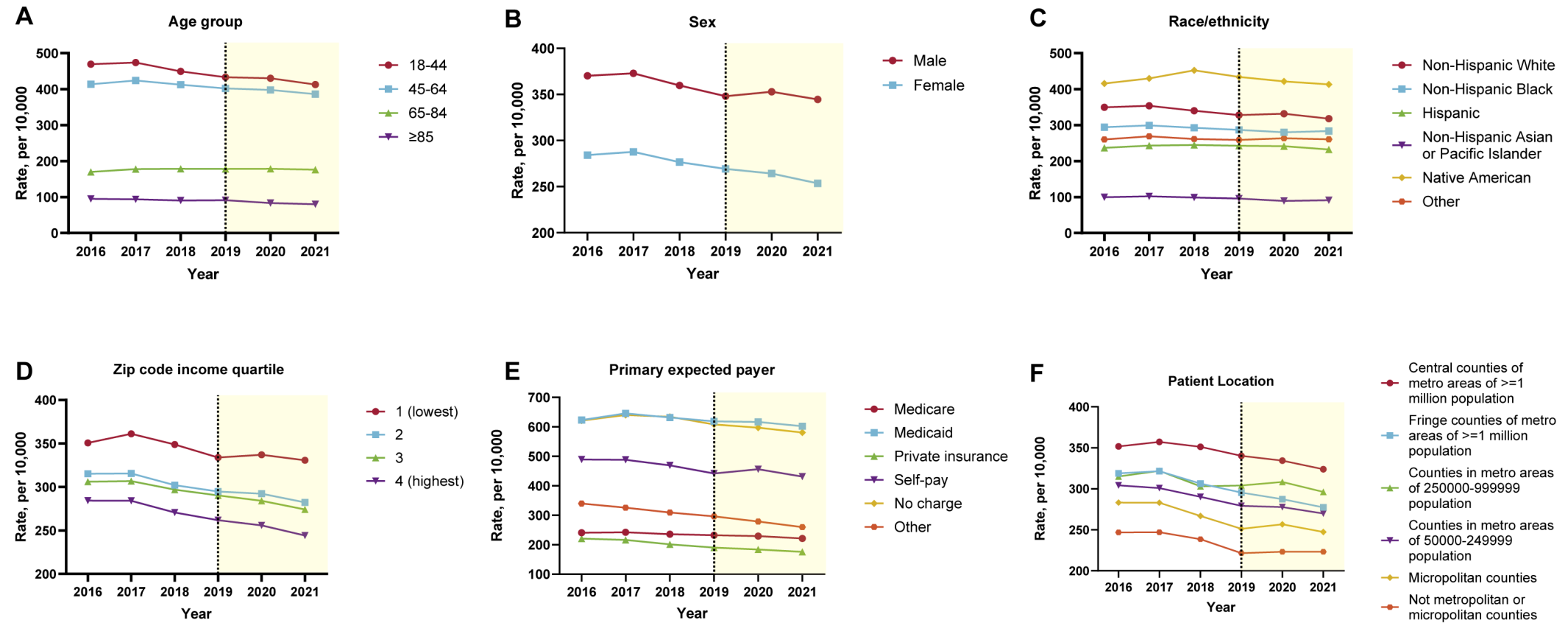


**Supplementary Figure S2. Opioid-Related Diagnoses (2016 to 2021) in Nationwide Emergency Department Sample for Subtypes by Age group (A), by Sex (B), by Race/ethnicity (C), by Zip code income quartile (D), by Primary expected payer (E) and by Patient location (F).**


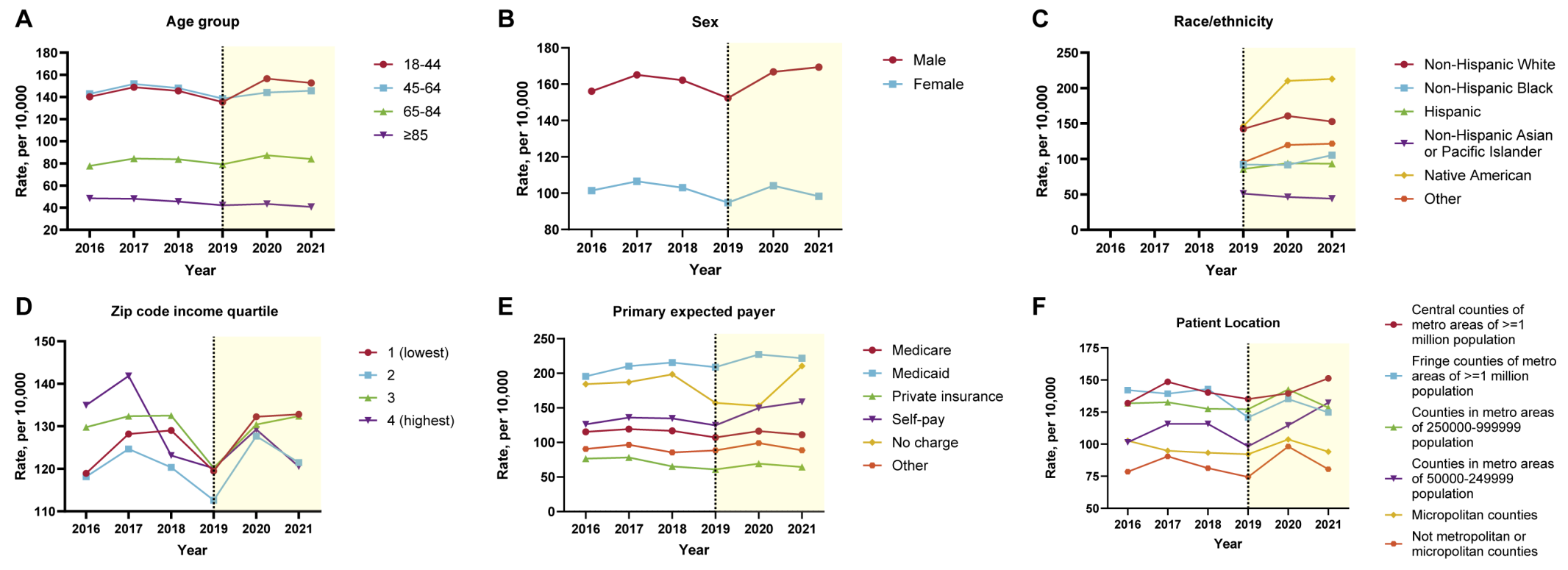


Note: Race variables provided by the Nationwide Emergency Department Sample are not reported until 2019 for the first time.

**Supplementary Figure S3. Opioid-Related Diagnoses (2016 to 2021) for Subtypes by Hospital region in National Inpatient Sample (A) and Nationwide Emergency Department Sample (B)**


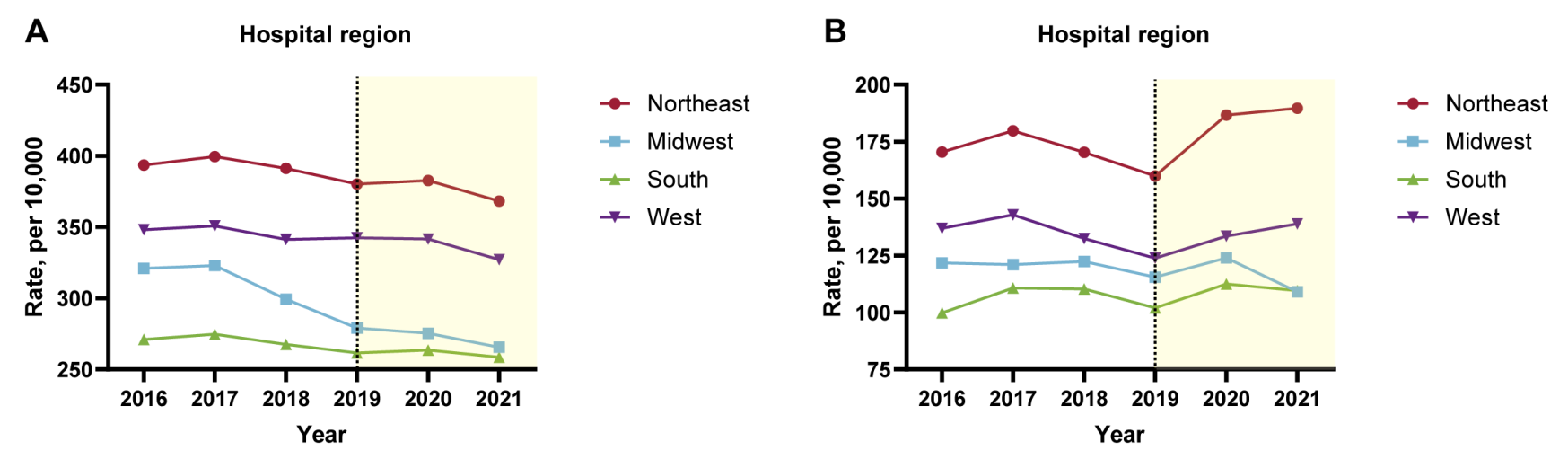


**Supplementary Figure S4. Nonfatal Opioid Overdose (2016 to 2021) in National Inpatient Sample for Subtypes by Age group (A), by Sex (B), by Race/ethnicity (C), by Zip code income quartile (D), by Primary expected payer (E) and by Patient location (F).**

**
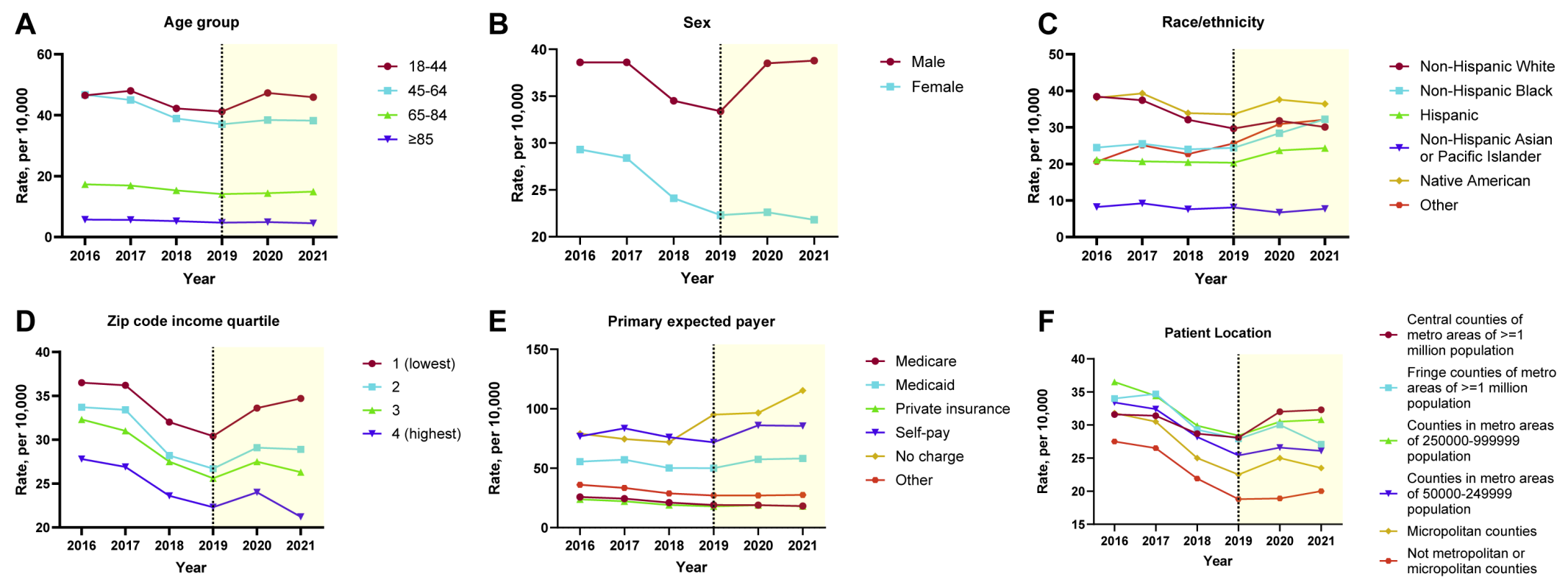
**

**Supplementary Figure S5. Injection Drug-Use Related Acute Infection (2016 to 2021) in National Inpatient Sample for Subtypes by Age group (A), by Sex (B), by Race/ethnicity (C), by Zip code income quartile (D), by Primary expected payer (E) and by Patient location (F).**

**
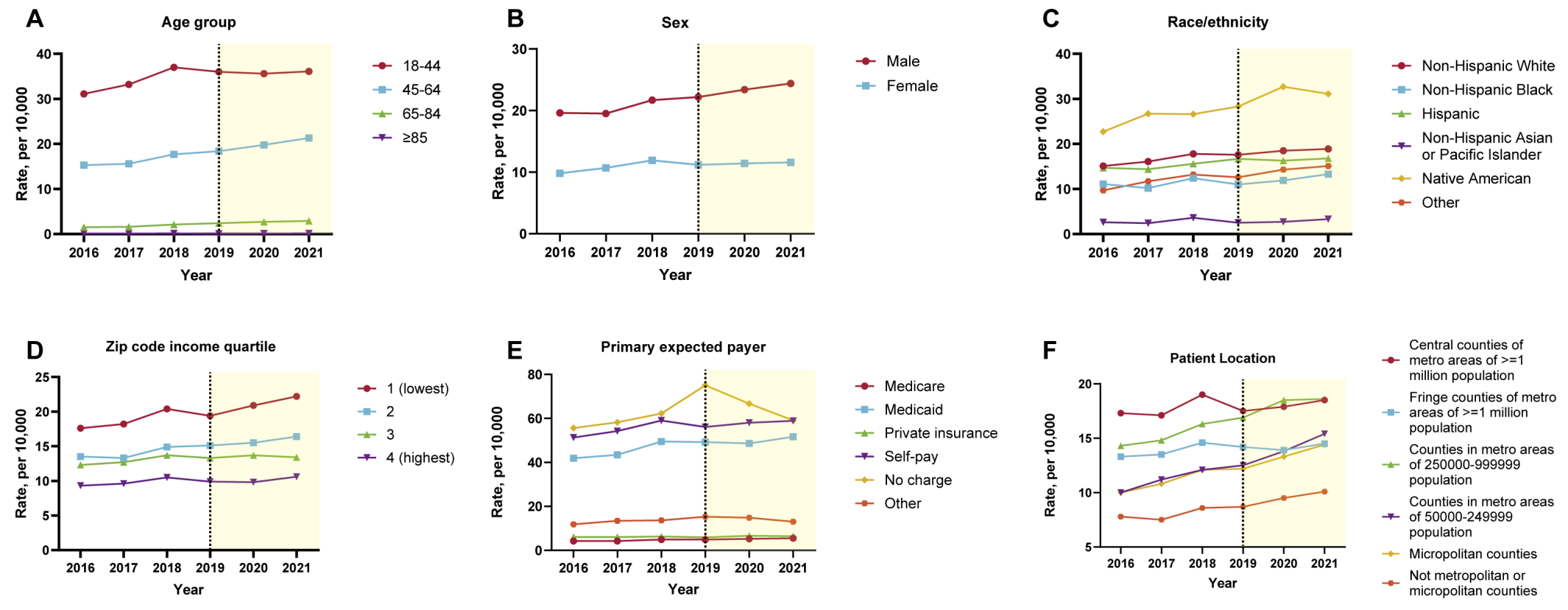
**

**Supplementary Figure S6. Substance Abuse Treatment (2016 to 2021) in National Inpatient Sample for Subtypes by Age group (A), by Sex (B), by Race/ethnicity (C), by Zip code income quartile (D), by Primary expected payer (E) and by Patient location (F).**

**
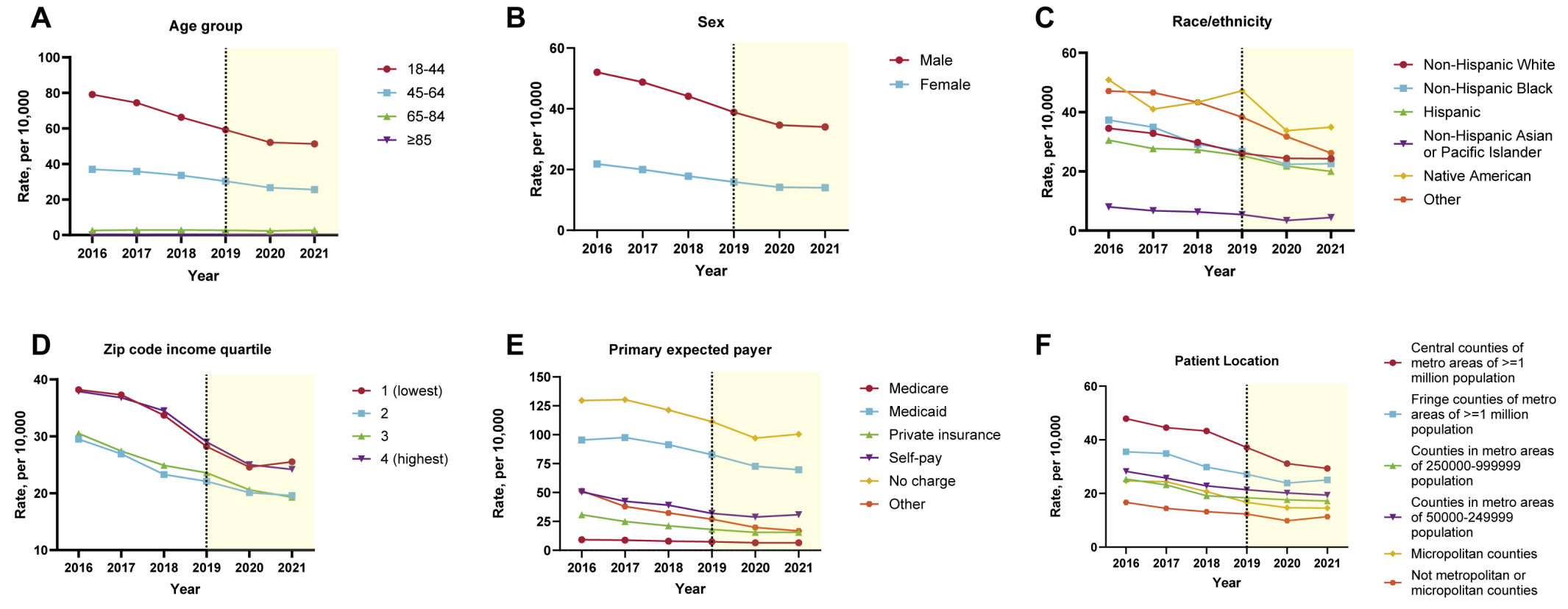
**

**Supplementary Figure S7. Nonfatal Opioid Overdose (2016 to 2021) in Nationwide Emergency Department Sample for Subtypes by Age group (A), by Sex (B), by Race/ethnicity (C), by Zip code income quartile (D), by Primary expected payer (E) and by Patient location (F).**

**
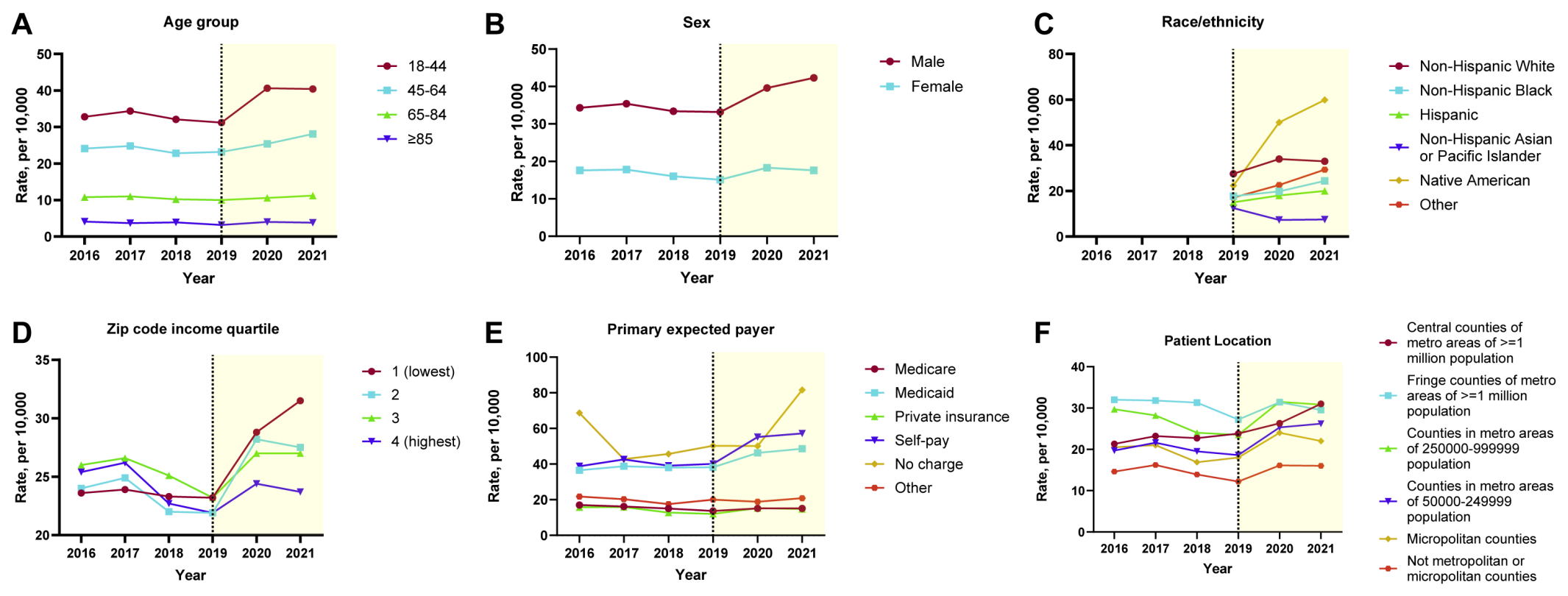
**

Note: Race variables provided by the Nationwide Emergency Department Sample are not reported until 2019 for the first time.

**Supplementary Figure S8. Injection Drug-Use Related Acute Infection (2016 to 2021) in Nationwide Emergency Department Sample for Subtypes by Age group (A), by Sex (B), by Race/ethnicity (C), by Zip code income quartile (D), by Primary expected payer (E) and by Patient location (F).**

**
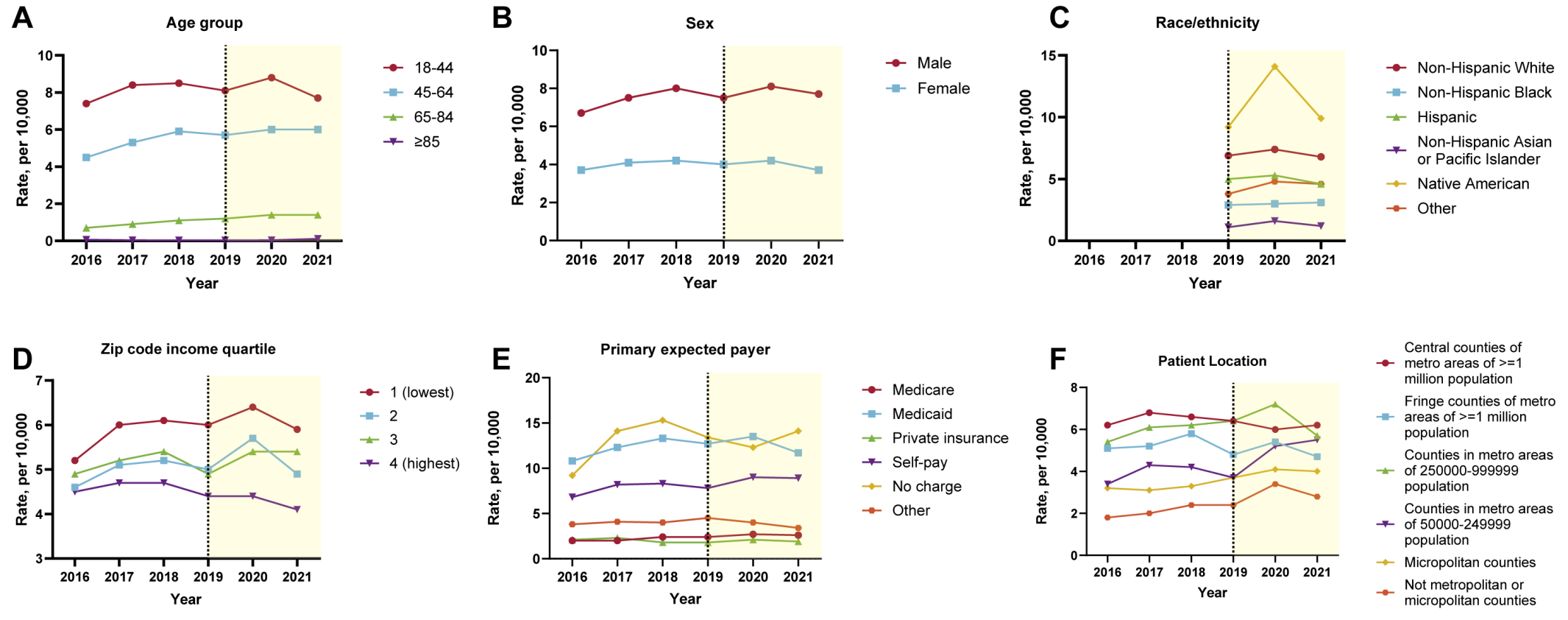
**

Note: Race variables provided by the Nationwide Emergency Department Sample are not reported until 2019 for the first time.

**Supplementary Figure S9. Substance Abuse Treatment (2016 to 2021) in Nationwide Emergency Department Sample for Subtypes by Age group (A), by Sex (B), by Race/ethnicity (C), by Zip code income quartile (D), by Primary expected payer (E) and by Patient location (F).**

**
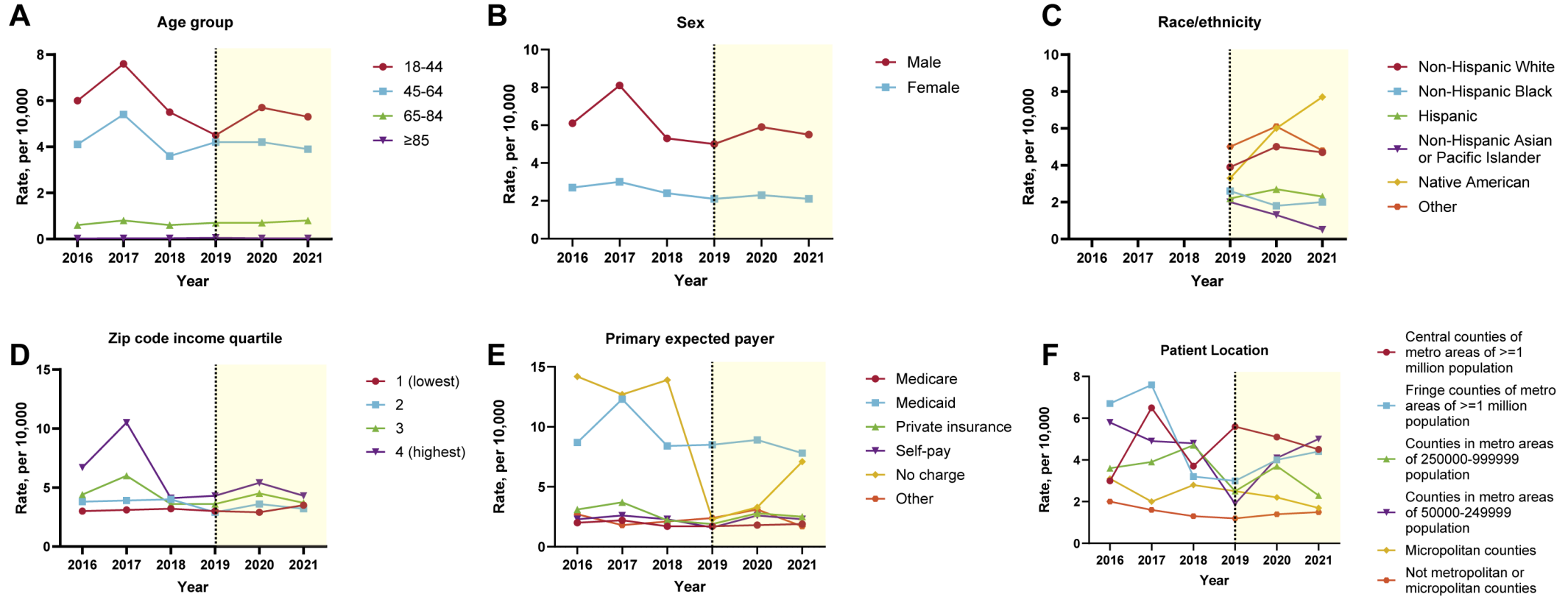
**

Note: Race variables provided by the Nationwide Emergency Department Sample are not reported until 2019 for the first time.

**Supplementary Figure S10. OUD-related clinical events for Subtypes by Hospital Region in National Inpatient Sample (A–C) and Nationwide Emergency Department Sample (D–F)**

**
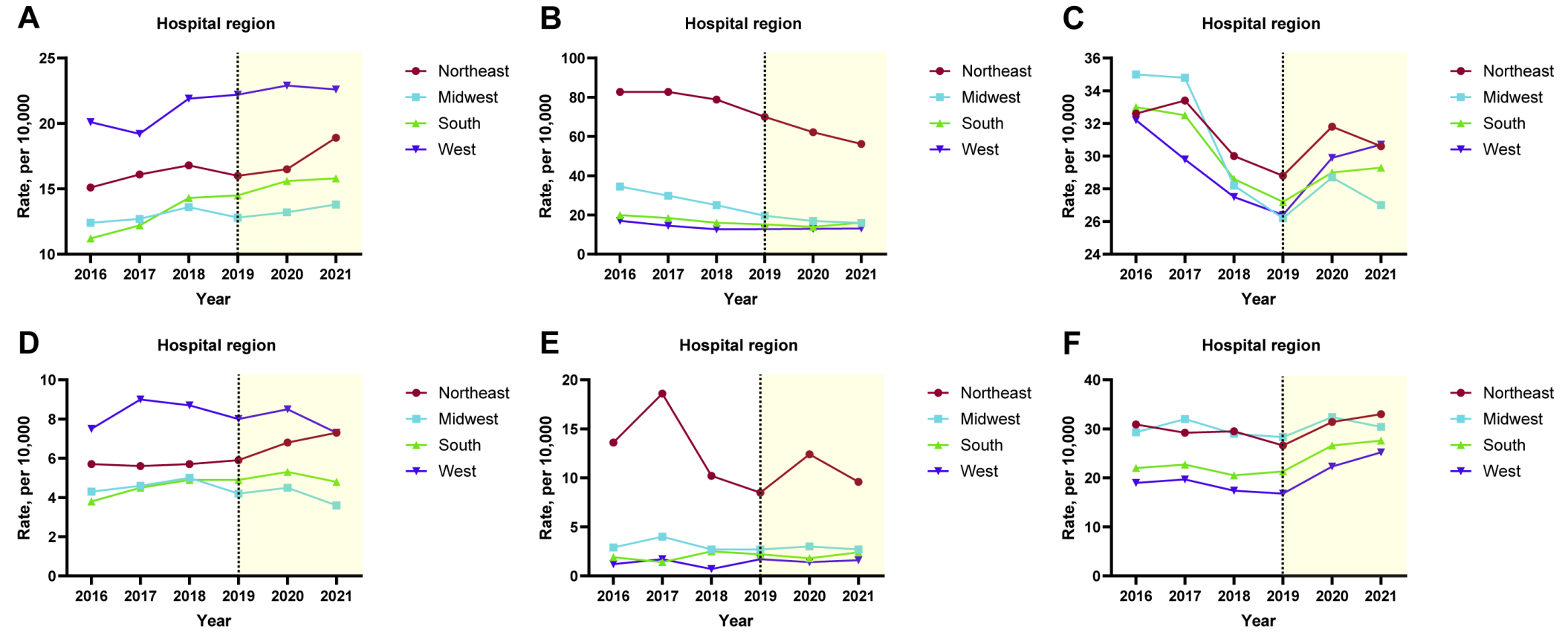
**

Note:Panel A. Nonfatal opioid overdose in National Inpatient Sample,

Panel B. Injection drug-use related acute infection in National Inpatient Sample,

Panel C. Substance abuse treatment in National Inpatient Sample,

Panel D. Nonfatal opioid overdose in Nationwide Emergency Department Sample,

Panel E. Injection drug-use related acute infection in Nationwide Emergency Department Sample,

Panel F. Substance abuse treatment in Nationwide Emergency Department Sample

**Supplementary Table S11. Opioid-Related Diagnoses^a^ (2016 to 2019 and 2020 to 2021) in National Inpatient Sample Overall and for Subtypes by Demographic Characteristics**

| **Characteristics** | **Between 2016 and 2019** | | | **During the COVID-19 pandemic in 2020 and 2021** | | |
| --- | --- | --- | --- | --- | --- | --- |
|  | **2019 Rate,**  **per 10,000** | **Absolute difference**  **from 2016-2019^b^** | **Change**  **from 2016-2019, %** | **2021 Rate,**  **per 10,000** | **Absolute difference**  **from 2019-2021^c^** | **Change**  **from 2019-2021, %** |
| **Overall** | 303.1  (294.3 to 311.9) | -17.1  (-30.1 to -4.2) | -5.4  (-9.4 to -1.3) | 293.4  (284.7 to 302.2) | -9.6  (-22.0 to 2.8) | -3.2  (-7.3 to 0.9) |
| **AGE** |  |  |  |  |  |  |
| **18-44** | 433.2  (416.4 to 450.0) | -36.3  (-61.6 to -11.0) | -7.7  (-13.1 to -2.3) | 412.7  (396.7 to 428.8) | -20.5  (-43.7 to 2.8) | -4.7  (-10.1 to 0.6) |
| **45-64** | 402.1  (388.8 to 415.3) | -11.5  (-30.7 to 7.7) | -2.8  (-7.4 to 1.9) | 386.1  (373.4 to 398.9) | -15.9  (-34.3 to 2.4) | -4.0  (-8.5 to 0.6) |
| **65-84** | 178.1  (172.5 to 183.8) | 8.3  (0.6 to 16.0) | 4.9  (0.3 to 9.4) | 176.3  (170.2 to 182.4) | -1.8  (-10.1 to 6.5) | -1.0  (-5.7 to 3.6) |
| **≥85** | 91.5  (88.0 to 95.1) | -3.7  (-8.9 to 1.6) | -3.9  (-9.4 to 1.6) | 80.1  (76.5 to 83.6) | -11.4  (-16.5 to -6.4) | -12.5  (-18.0 to -7.0) |
| **Sex** |  |  |  |  |  |  |
| **Male** | 348.0  (336.3 to 359.8) | -22.2  (-39.7 to -4.6) | -6.0  (-10.7 to -1.3) | 344.5  (333.0 to 355.9) | -3.6  (-20.0 to 12.9) | -1.0  (-5.7 to 3.7) |
| **Female** | 269.2  (261.8 to 276.6) | -15.1  (-25.9 to -4.2) | -5.3  (-9.1 to -1.5) | 253.6  (246.2 to 260.9) | -15.7  (-26.1 to -5.2) | -5.8  (-9.7 to -1.9) |
| **Race/ethnicity^d^** |  |  |  |  |  |  |
| **Non-Hispanic White** | 328.2  (319.1 to 337.3) | -21.4  (-35.1 to -7.7) | -6.1  (-10.0 to -2.2) | 317.9  (308.7 to 327.1) | -10.3  (-23.2 to 2.7) | -3.1  (-7.1 to 0.8) |
| **Non-Hispanic Black** | 286.9  (269.5 to 304.4) | -7.6  (-34.6 to 19.3) | -2.6  (-11.7 to 6.6) | 283.5  (267.1 to 299.9) | -3.4  (-27.3 to 20.5) | -1.2  (-9.5 to 7.2) |
| **Hispanic** | 242.7  (224.3 to 261.1) | 6.0  (-19.1 to 31.2) | 2.5  (-8.1 to 13.2) | 232.2  (215.5 to 248.9) | -10.5  (-35.3 to 14.4) | -4.3  (-14.6 to 5.9) |
| **Non-Hispanic Asian or Pacific Islander** | 96.0  (87.0 to 105.0) | -3.8  (-17.7 to 10.1) | -3.8  (-17.7 to 10.1) | 91.1  (83.1 to 99.1) | -4.9  (-16.9 to 7.1) | -5.1  (-17.6 to 7.4) |
| **Native American** | 434.2  (383.6 to 484.9) | 18.5  (-54.8 to 91.7) | 4.4  (-13.2 to 22.1) | 413.3  (360.5 to 466.1) | -20.9  (-94.1 to 52.2) | -4.8  (-21.7 to 12.0) |
| **Other** | 259.0  (231.1 to 286.8) | -1.1  (-40.2 to 38.0) | -0.4  (-15.5 to 14.6) | 260.6  (240.1 to 281.1) | 1.6  (-32.9 to 36.2) | 0.6  (-12.7 to 14.0) |
| **Zip code income quartile^e^** |  |  |  |  |  |  |
| **1 (lowest)** | 333.8  (319.7 to 348.0) | -16.9  (-37.4 to 3.5) | -4.8  (-10.7 to 1.0) | 330.7  (316.7 to 344.8) | -3.1  (-23.0 to 16.9) | -0.9  (-6.9 to 5.1) |
| **2** | 294.8  (285.6 to 304.0) | -20.5  (-34.5 to -6.5) | -6.5  (-10.9 to -2.1) | 282.2  (272.9 to 291.6) | -12.6  (-25.7 to 0.6) | -4.3  (-8.7 to 0.2) |
| **3** | 290.3  (280.9 to 299.7) | -15.7  (-29.3 to -2.0) | -5.1  (-9.6 to -0.7) | 274.1  (265.1 to 283.1) | -16.2  (-29.2 to -3.2) | -5.6  (-10.1 to -1.1) |
| **4 (highest)** | 261.8  (250.0 to 273.5) | -22.5  (-39.6 to -5.4) | -7.9  (-13.9 to -1.9) | 244.2  (233.1 to 255.3) | -17.6  (-33.8 to -1.4) | -6.7  (-12.9 to -0.5) |
| **Primary expected payer^f^** |  |  |  |  |  |  |
| **Medicare** | 232.1  (225.3 to 238.9) | -8.7  (-18.4 to 1.0) | -3.6  (-7.7 to 0.4) | 221.4  (214.4 to 228.3) | -10.8  (-20.5 to -1.1) | -4.6  (-8.8 to -0.5) |
| **Medicaid** | 618.8  (591.8 to 645.7) | -4.5  (-42.8 to 33.8) | -0.7  (-6.9 to 5.4) | 602.4  (577.1 to 627.7) | -16.3  (-53.3 to 20.6) | -2.6  (-8.6 to 3.3) |
| **Private insurance** | 190.1  (183.0 to 197.2) | -30.5  (-42.7 to -18.4) | -13.8  (-19.3 to -8.3) | 175.8  (168.5 to 183.1) | -14.2  (-24.4 to -4.1) | -7.5  (-12.9 to -2.1) |
| **Self-pay** | 441.6  (417.7 to 465.5) | -47.7  (-81.4 to -14.1) | -9.8  (-16.6 to -2.9) | 431.4  (406.3 to 456.5) | -10.2  (-44.9 to 24.4) | -2.3  (-10.2 to 5.5) |
| **No charge** | 608.2  (471.4 to 745.0) | -13.1  (-244.9 to 218.7) | -2.1  (-39.4 to 35.2) | 580.6  (463.2 to 697.9) | -27.7  (-207.8 to 152.5) | -4.5  (-34.2 to 25.1) |
| **Other** | 296.1  (273.3 to 318.9) | -43.6  (-84.6 to -2.6) | -12.8  (-24.9 to -0.8) | 259.6  (240.2 to 278.9) | -36.5  (-66.4 to -6.6) | -12.3  (-22.4 to -2.2) |
| **Patient Location^g^** |  |  |  |  |  |  |
| **"Central" counties of metro areas of >=1 million population** | 340.2  (322.1 to 358.3) | -11.6  (-37.8 to 14.7) | -3.3  (-10.7 to 4.2) | 323.9  (306.8 to 341.0) | -16.3  (-41.2 to 8.6) | -4.8  (-12.1 to 2.5) |
| **"Fringe" counties of metro areas of >=1 million population** | 295.4  (281.7 to 309.0) | -23.5  (-43.8 to -3.3) | -7.4  (-13.7 to -1.0) | 277.4  (264.0 to 290.7) | -18.0  (-37.2 to 1.1) | -6.1  (-12.6 to 0.4) |
| **Counties in metro areas of 250,000-999,999 population** | 304.0  (288.9 to 319.0) | -11.1  (-32.4 to 10.3) | -3.5  (-10.3 to 3.3) | 296.3  (281.4 to 311.3) | -7.6  (-28.8 to 13.6) | -2.5  (-9.5 to 4.5) |
| **Counties in metro areas of 50,000-249,999 population** | 279.2  (260.1 to 298.4) | -25.0  (-53.8 to 3.8) | -8.2  (-17.7 to 1.3) | 269.9  (249.8 to 290.0) | -9.3  (-37.1 to 18.4) | -3.3  (-13.3 to 6.6) |
| **Micropolitan counties** | 251.3  (239.4 to 263.3) | -31.9  (-51.0 to -12.7) | -11.3  (-18.0 to -4.5) | 247.4  (235.8 to 259.1) | -3.9  (-20.6 to 12.8) | -1.6  (-8.2 to 5.1) |
| **Not metropolitan or micropolitan counties** | 221.6  (200.1 to 243.1) | -25.3  (-55.8 to 5.1) | -10.3  (-22.6 to 2.1) | 223.3  (199.9 to 246.7) | 1.7  (-30.1 to 33.5) | 0.8  (-13.6 to 15.1) |
| **Hospital region** |  |  |  |  |  |  |
| **Northeast** | 380.2  (352.4 to 408.1) | -13.3  (-52.8 to 26.2) | -3.4  (-13.4 to 6.7) | 368.2  (340.9 to 395.5) | -12.0  (-50.9 to 26.9) | -3.2  (-13.4 to 7.1) |
| **Midwest** | 279.0  (263.2 to 294.8) | -42.0  (-67.3 to -16.7) | -13.1  (-21.0 to -5.2) | 265.5  (250.9 to 280.2) | -13.5  (-35.0 to 8.0) | -4.8  (-12.5 to 2.9) |
| **South** | 261.5  (248.4 to 274.6) | -9.5  (-28.8 to 9.8) | -3.5  (-10.6 to 3.6) | 258.5  (245.1 to 271.8) | -3.0  (-21.7 to 15.7) | -1.1  (-8.3 to 6.0) |
| **West** | 342.4  (324.6 to 360.3) | -5.7  (-31.2 to 19.8) | -1.6  (-9.0 to 5.7) | 327.1  (308.9 to 345.3) | -15.3  (-40.8 to 10.2) | -4.5  (-11.9 to 3.0) |

^a^ Cases of opioid-related diagnoses were identified following the definition proposed by the Healthcare Cost and Utilization Project, including two subtypes: abuse or dependence, and adverse event or poisoning (classified by opioid category, including: synthetic opioids as a proxy for fentanyl, prescription natural/semisynthetic opioids as a proxy for opioid pain medications, heroin, opium, methadone, and other opioids).

^b^ Reflects the overall or total difference between 2016 and 2019: absolute difference (2019 – 2016) and percentage change [(2019 – 2016)/2016 × 100].

^c^ Reflects the overall or total difference during the COVID-19 pandemic in 2020 and 2021: absolute difference (2021 – 2019) and percentage change [(2021 – 2019)/2019 × 100].

^d^ Race/ethnicity variable was obtained from the Agency for Healthcare Research and Quality, Healthcare Cost and Utilization Project; nationally weighted from all records in states with reliable race/ethnicity reporting, which is collected by self-report on admission to the hospital using fixed categories.

^e^ Median household income of residents in the patient’s zip code was updated annually; 2019 range for quartile 1 was less than $48,000; quartile 2, $48,000 to $60,999; quartile 3, $61,000 to $81,999; and quartile 4, at least $82,000; 2021 range for quartile 1 was less than $52,000; quartile 2, $52,000 to $65,999; quartile 3, $66,000 to $87,999; and quartile 4, at least $88,000.

^f^ “Medicare” includes both fee-for-service and managed care Medicare patients. “Medicaid” includes both fee-for-service and managed care Medicaid patients. “Private insurance” includes Blue Cross, commercial carriers, and private HMOs and PPOs. “Other” includes Worker's Compensation, CHAMPUS (Civilian Health and Medical Program of the Uniformed Services), CHAMPVA (Civilian Health and Medical Program of the Department of Veteran's Affairs), Title V, and other government programs.

^g^ “Patient location” is a six-category urban-rural classification scheme for U.S. counties developed by the National Center for Health Statistics (NCHS) especially for use in health care research. The classification emphasizes urban distinctions and is unique in differentiating between central and fringe counties of large metropolitan areas. Smaller metropolitan counties are subdivided by population. Non-metropolitan counties are divided simply into micropolitan and non-core categories.

**Supplementary Table S12. Opioid-Related Diagnoses^a^ (2016 to 2019 and 2020 to 2021) in Nationwide Emergency Department Sample Overall and for Subtypes by Demographic Characteristics**

| **Characteristics** | **Between 2016 and 2019** | | | **During the COVID-19 pandemic in 2020 and 2021** | | |
| --- | --- | --- | --- | --- | --- | --- |
|  | **2019 Rate,**  **per 10,000** | **Absolute difference**  **from 2016-2019^b^** | **Change**  **from 2016-2019, %** | **2021 Rate,**  **per 10,000** | **Absolute difference**  **from 2019-2021^c^** | **Change**  **from 2019-2021, %** |
| **Overall** | 119.8  (112.2 to 127.4) | -5.0  (-15.3 to 5.2) | -4.0  (-12.2 to 4.2) | 130.2  (120.1 to 140.4) | 10.4  (-2.3 to 23.1) | 8.7  (-1.9 to 19.2) |
| **AGE** |  |  |  |  |  |  |
| **18-44** | 135.4  (125.8 to 145) | -4.7  (-18.3 to 8.9) | -3.4  (-13.1 to 6.4) | 152.6  (139.5 to 165.6) | 17.2  (1.0 to 33.3) | 12.7  (0.8 to 24.6) |
| **45-64** | 138.6  (126.5 to 150.7) | -4.3  (-19.6 to 11.1) | -3.0  (-13.7 to 7.7) | 145.5  (131.8 to 159.2) | 7.0  (-11.3 to 25.2) | 5.0  (-8.2 to 18.2) |
| **65-84** | 79.2  (74.7 to 83.7) | 1.5  (-4.9 to 7.8) | 1.9  (-6.2 to 10.1) | 83.9  (78.4 to 89.4) | 4.7  (-2.4 to 11.8) | 5.9  (-3.0 to 14.9) |
| **≥85** | 42.1  (39.7 to 44.6) | -6.2  (-10.4 to -2.0) | -12.9  (-21.6 to -4.1) | 40.6  (37.9 to 43.4) | -1.5  (-5.2 to 2.2) | -3.5  (-12.2 to 5.2) |
| **Sex** |  |  |  |  |  |  |
| **Male** | 152.4  (141.4 to 163.3) | -3.8  (-18.1 to 10.5) | -2.4  (-11.6 to 6.7) | 169.4  (154.1 to 184.7) | 17.0  (-1.8 to 35.8) | 11.2  (-1.2 to 23.5) |
| **Female** | 94.8  (89.5 to 100.1) | -6.7  (-14.2 to 0.8) | -6.6  (-14.0 to 0.8) | 98.3  (92.0 to 104.6) | 3.5  (-4.7 to 11.7) | 3.7  (-4.9 to 12.4) |
| **Race/ethnicity^e^** |  |  |  |  |  |  |
| **Non-Hispanic White** | 142.3  (134.2 to 150.5) | NA^d^ | NA^d^ | 152.8  (142.5 to 163.2) | 10.5  (-2.7 to 23.7) | 7.4  (-1.9 to 16.6) |
| **Non-Hispanic Black** | 92.1  (74.6 to 109.6) | NA^d^ | NA^d^ | 105.4  (88.5 to 122.4) | 13.3  (-11.0 to 37.6) | 14.4  (-11.9 to 40.8) |
| **Hispanic** | 85.9  (74.9 to 96.9) | NA^d^ | NA^d^ | 93.3  (80.5 to 106.1) | 7.4  (-9.4 to 24.3) | 8.7  (-11.0 to 28.3) |
| **Non-Hispanic Asian or Pacific Islander** | 51.1  (30.5 to 71.7) | NA^d^ | NA^d^ | 44.1  (38.4 to 49.7) | -7.1  (-28.4 to 14.3) | -13.8  (-55.6 to 28.0) |
| **Native American** | 146.4  (117.3 to 175.6) | NA^d^ | NA^d^ | 212.9  (149.4 to 276.4) | 66.5  (-3.3 to 136.3) | 45.4  (-2.2 to 93.1) |
| **Other** | 95.1  (82.9 to 107.2) | NA^d^ | NA^d^ | 121.7  (104.6 to 138.7) | 26.6  (5.6 to 47.5) | 27.9  (5.9 to 49.9) |
| **Zip code income quartile^f^** |  |  |  |  |  |  |
| **1 (lowest)** | 119.5  (108.0 to 130.9) | 0.5  (-14.5 to 15.6) | 0.4  (-12.2 to 13.1) | 132.8  (121.9 to 143.7) | 13.4  (-2.4 to 29.1) | 11.2  (-2.0 to 24.4) |
| **2** | 112.6  (105.0 to 120.2) | -5.5  (-16.1 to 5.0) | -4.7  (-13.6 to 4.3) | 121.4  (112.5 to 130.4) | 8.8  (-2.9 to 20.6) | 7.8  (-2.6 to 18.3) |
| **3** | 120.2  (112.6 to 127.8) | -9.6  (-20.7 to 1.5) | -7.4  (-15.9 to 1.2) | 132.4  (112.0 to 152.8) | 12.2  (-9.6 to 33.9) | 10.1  (-8.0 to 28.2) |
| **4 (highest)** | 120.1  (111.5 to 128.8) | -14.8  (-29.6 to -0.02) | -11.0  (-21.9 to -0.02) | 120.5  (107.8 to 133.3) | 0.4  (-15.0 to 15.8) | 0.3  (-12.5 to 13.2) |
| **Primary expected payer^g^** |  |  |  |  |  |  |
| **Medicare** | 107.2  (101.5 to 112.8) | -8.2  (-16.6 to 0.1) | -7.1  (-14.4 to 0.1) | 111.1  (104 to 118.3) | 3.9  (-5.2 to 13.1) | 3.7  (-4.8 to 12.2) |
| **Medicaid** | 208.9  (189.8 to 227.9) | 13.4  (-10.7 to 37.6) | 6.9  (-5.5 to 19.2) | 221.7  (202.1 to 241.4) | 12.8  (-14.5 to 40.1) | 6.1  (-7.0 to 19.2) |
| **Private insurance** | 61.0  (57.0 to 65.0) | -15.5  (-22.2 to -8.8) | -20.2  (-29.0 to -11.5) | 64.4  (58.7 to 70.0) | 3.4  (-3.5 to 10.3) | 5.5  (-5.8 to 16.8) |
| **Self-pay** | 124.7  (111.7 to 137.7) | -1.4  (-18.5 to 15.6) | -1.1  (-14.6 to 12.4) | 158.7  (125.1 to 192.4) | 34.0  (-2.0 to 70.0) | 27.3  (-1.6 to 56.2) |
| **No charge** | 157.3  (122.2 to 192.4) | -26.9  (-120.1 to 66.2) | -14.6  (-65.2 to 35.9) | 210.5  (149.9 to 271.0) | 53.2  (-16.7 to 123.1) | 33.8  (-10.6 to 78.2) |
| **Other** | 88.5  (73.5 to 103.5) | -2.1  (-20.5 to 16.3) | -2.3  (-22.6 to 18.0) | 88.7  (78.2 to 99.2) | 0.2  (-18.1 to 18.4) | 0.2  (-20.5 to 20.8) |
| **Patient Location^h^** |  |  |  |  |  |  |
| **"Central" counties of metro areas of >=1 million population** | 135.1  (118.0 to 152.2) | 3.2  (-18.8 to 25.2) | 2.4  (-14.3 to 19.1) | 151.3  (126.9 to 175.6) | 16.2  (-13.6 to 45.9) | 12.0  (-10.0 to 34.0) |
| **"Fringe" counties of metro areas of >=1 million population** | 120.7  (109.8 to 131.6) | -21.4  (-39.6 to -3.2) | -15.1  (-27.9 to -2.3) | 124.8  (110.6 to 139.0) | 4.1  (-13.8 to 22.0) | 3.4  (-11.5 to 18.2) |
| **Counties in metro areas of 250,000-999,999 population** | 127.2  (112.1 to 142.3) | -4.6  (-25.4 to 16.1) | -3.5  (-19.2 to 12.2) | 128.4  (115.6 to 141.3) | 1.2  (-18.6 to 21.0) | 0.9  (-14.6 to 16.5) |
| **Counties in metro areas of 50,000-249,999 population** | 98.3  (89.0 to 107.5) | -3.1  (-19.1 to 12.8) | -3.1  (-18.8 to 12.6) | 132.3  (112.3 to 152.4) | 34.1  (12.0 to 56.1) | 34.7  (12.2 to 57.1) |
| **Micropolitan counties** | 92.2  (83.5 to 100.8) | -10.5  (-22.6 to 1.6) | -10.2  (-22.0 to 1.5) | 94.2  (84.8 to 103.5) | 2.0  (-10.7 to 14.7) | 2.2  (-11.6 to 15.9) |
| **Not metropolitan or micropolitan counties** | 74.4  (66.9 to 81.9) | -4.1  (-15.3 to 7.1) | -5.2  (-19.4 to 9.0) | 80.4  (72.2 to 88.6) | 6.0  (-5.1 to 17.1) | 8.1  (-6.9 to 23.0) |
| **Hospital region** |  |  |  |  |  |  |
| **Northeast** | 159.9  (145 to 174.8) | -10.5  (-36.5 to 15.5) | -6.2  (-21.4 to 9.1) | 189.7  (148.1 to 231.2) | 29.8  (-14.3 to 73.9) | 18.6  (-9.0 to 46.2) |
| **Midwest** | 115.5  (98.4 to 132.5) | -6.2  (-26.9 to 14.5) | -5.1  (-22.1 to 11.9) | 109.1  (95.9 to 122.2) | -6.4  (-27.9 to 15.1) | -5.5  (-24.2 to 13.1) |
| **South** | 102.0  (88.9 to 115.1) | 2.2  (-14.4 to 18.8) | 2.2  (-14.5 to 18.9) | 109.6  (97.6 to 121.6) | 7.6  (-10.2 to 25.4) | 7.5  (-10.0 to 24.9) |
| **West** | 123.8  (111.1 to 136.5) | -13.1  (-34.1 to 7.9) | -9.6  (-24.9 to 5.8) | 138.9  (123.1 to 154.7) | 15.1  (-5.1 to 35.3) | 12.2  (-4.1 to 28.5) |

^a^ Cases of opioid-related diagnoses were identified following the definition proposed by the Healthcare Cost and Utilization Project, including two subtypes: abuse or dependence, and adverse event or poisoning (classified by opioid category, including: synthetic opioids as a proxy for fentanyl, prescription natural/semisynthetic opioids as a proxy for opioid pain medications, heroin, opium, methadone, and other opioids).

^b^ Reflects the overall or total difference between 2016 and 2019: absolute difference (2019 – 2016) and percentage change [(2019 – 2016)/2016 × 100].

^c^ Reflects the overall or total difference during the COVID-19 pandemic in 2020 and 2021: absolute difference (2021 – 2019) and percentage change [(2021 – 2019)/2019 × 100].

^d^ NA, Not Available. Due to the NEDS survey design, the Race/ethnicity classification was not available until 2019 and therefore could not present estimates.

^e^ Race/ethnicity variable was obtained from the Agency for Healthcare Research and Quality, Healthcare Cost and Utilization Project; nationally weighted from all records in states with reliable race/ethnicity reporting, which is collected by self-report on admission to the hospital using fixed categories.

^f^ Median household income of residents in the patient’s zip code was updated annually; 2019 range for quartile 1 was less than $48,000; quartile 2, $48,000 to $60,999; quartile 3, $61,000 to $81,999; and quartile 4, at least $82,000; 2021 range for quartile 1 was less than $52,000; quartile 2, $52,000 to $65,999; quartile 3, $66,000 to $87,999; and quartile 4, at least $88,000.

^g^ “Medicare” includes both fee-for-service and managed care Medicare patients. “Medicaid” includes both fee-for-service and managed care Medicaid patients. “Private insurance” includes Blue Cross, commercial carriers, and private HMOs and PPOs. “Other” includes Worker's Compensation, CHAMPUS (Civilian Health and Medical Program of the Uniformed Services), CHAMPVA (Civilian Health and Medical Program of the Department of Veteran's Affairs), Title V, and other government programs.

^h^ “Patient location” is a six-category urban-rural classification scheme for U.S. counties developed by the National Center for Health Statistics (NCHS) especially for use in health care research. The classification emphasizes urban distinctions and is unique in differentiating between central and fringe counties of large metropolitan areas. Smaller metropolitan counties are subdivided by population. Non-metropolitan counties are divided simply into micropolitan and non-core categories.

**Supplementary Table S13. Opioid-Related Abuse or Dependence^a^ (2016 to 2019 and 2020 to 2021) in National Inpatient Sample Overall and for Subtypes by Demographic Characteristics**

| **Characteristics** | **Between 2016 and 2019** | | | **During the COVID-19 pandemic in 2020 and 2021** | | |
| --- | --- | --- | --- | --- | --- | --- |
|  | **2019 Rate,**  **per 10,000** | **Absolute difference**  **from 2016-2019^b^** | **Change**  **from 2016-2019, %** | **2021 Rate,**  **per 10,000** | **Absolute difference**  **from 2019-2021^c^** | **Change**  **from 2019-2021, %** |
| **Overall** | 229.1  (220.6 to 237.5) | -9.8  (-22.4 to 2.7) | -4.1  (-9.4 to 1.1) | 226.8  (218.5 to 235.1) | -2.3  (-14.1 to 9.6) | -1.0  (-6.2 to 4.2) |
| **AGE** |  |  |  |  |  |  |
| **18-44** | 380.2  (363.9 to 396.4) | -32.8  (-57.4 to -8.1) | -7.9  (-13.9 to -2.0) | 359.0  (343.6 to 374.5) | -21.1  (-43.5 to 1.3) | -5.6  (-11.5 to 0.3) |
| **45-64** | 310.3  (297.3 to 323.3) | -2.8  (-21.7 to 16.0) | -0.9  (-6.9 to 5.1) | 304.1  (291.8 to 316.4) | -6.2  (-24.0 to 11.7) | -2.0  (-7.7 to 3.8) |
| **65-84** | 98.7  (93.6 to 103.8) | 18.0  (11.1 to 24.9) | 22.3  (13.8 to 30.8) | 107.4  (101.9 to 112.9) | 8.7  (1.2 to 16.2) | 8.8  (1.2 to 16.4) |
| **≥85** | 28.9  (26.5 to 31.3) | 4.0  (0.5 to 7.4) | 16.0  (2.2 to 29.8) | 27.9  (25.5 to 30.4) | -0.9  (-4.3 to 2.4) | -3.3  (-15.0 to 8.5) |
| **Sex** |  |  |  |  |  |  |
| **Male** | 274.7  (263.1 to 286.2) | -16.6  (-33.9 to 0.8) | -5.7  (-11.6 to 0.3) | 275.7  (264.6 to 286.8) | 1.1  (-15.0 to 17.1) | 0.4  (-5.4 to 6.2) |
| **Female** | 194.7  (187.8 to 201.6) | -6.6  (-16.8 to 3.6) | -3.3  (-8.4 to 1.8) | 188.6  (181.7 to 195.4) | -6.1  (-15.9 to 3.6) | -3.2  (-8.1 to 1.8) |
| **Race/ethnicity^d^** |  |  |  |  |  |  |
| **Non-Hispanic White** | 247.0  (238.1 to 255.8) | -11.7  (-25.1 to 1.7) | -4.5  (-9.7 to 0.6) | 246.8  (237.9 to 255.6) | -0.2  (-12.7 to 12.3) | -0.1  (-5.2 to 5.0) |
| **Non-Hispanic Black** | 221.5  (205.0 to 238.1) | -8.2  (-34.3 to 17.8) | -3.6  (-14.9 to 7.8) | 217.7  (202.6 to 232.8) | -3.9  (-26.2 to 18.5) | -1.7  (-11.8 to 8.4) |
| **Hispanic** | 190.4  (172.6 to 208.2) | 4.7  (-19.6 to 28.9) | 2.5  (-10.6 to 15.6) | 182.4  (166.2 to 198.6) | -8.1  (-32.1 to 16.0) | -4.2  (-16.9 to 8.4) |
| **Non-Hispanic Asian or Pacific Islander** | 48.2  (41.6 to 54.8) | -3.3  (-14.2 to 7.6) | -6.4  (-27.5 to 14.7) | 49.6  (43.5 to 55.6) | 1.3  (-7.6 to 10.3) | 2.8  (-15.8 to 21.4) |
| **Native American** | 362.4  (312.6 to 412.3) | 25.8  (-44.2 to 95.8) | 7.7  (-13.1 to 28.5) | 346.8  (297.7 to 395.9) | -15.6  (-85.6 to 54.3) | -4.3  (-23.6 to 15.0) |
| **Other** | 199.6  (171.8 to 227.4) | -1.2  (-40.4 to 38.0) | -0.6  (-20.1 to 19.0) | 200.4  (180.5 to 220.3) | 0.8  (-33.4 to 34.9) | 0.4  (-16.7 to 17.5) |
| **Zip code income quartile^e^** |  |  |  |  |  |  |
| **1 (lowest)** | 266.4  (252.8 to 280.1) | -10.9  (-30.7 to 9.0) | -3.9  (-11.1 to 3.3) | 267.4  (254.0 to 280.8) | 1.0  (-18.1 to 20.1) | 0.4  (-6.8 to 7.5) |
| **2** | 222.3  (213.6 to 231.1) | -11.2  (-24.6 to 2.3) | -4.8  (-10.5 to 1.0) | 217.8  (208.9 to 226.6) | -4.5  (-17.0 to 7.9) | -2.0  (-7.6 to 3.6) |
| **3** | 212.8  (203.8 to 221.8) | -7.3  (-20.4 to 5.7) | -3.3  (-9.3 to 2.6) | 206  (197.6 to 214.5) | -6.8  (-19.1 to 5.6) | -3.2  (-9.0 to 2.6) |
| **4 (highest)** | 179.6  (168.4 to 190.9) | -16.6  (-33.1 to -0.1) | -8.5  (-16.9 to -0.04) | 172.5  (161.9 to 183.1) | -7.1  (-22.6 to 8.3) | -4.0  (-12.6 to 4.6) |
| **Primary expected payer^f^** |  |  |  |  |  |  |
| **Medicare** | 150.4  (144.0 to 156.7) | 2.8  (-6.2 to 11.8) | 1.9  (-4.2 to 8.0) | 150.5  (144.1 to 156.9) | 0.1  (-8.8 to 9.1) | 0.1  (-5.9 to 6.1) |
| **Medicaid** | 551.2  (524.7 to 577.7) | -2.1  (-39.7 to 35.5) | -0.4  (-7.2 to 6.4) | 532.6  (508.0 to 557.3) | -18.6  (-54.8 to 17.6) | -3.4  (-9.9 to 3.2) |
| **Private insurance** | 125.0  (118.4 to 131.6) | -25.8  (-37.4 to -14.2) | -17.1  (-24.8 to -9.4) | 120.4  (113.8 to 126.9) | -4.7  (-14.0 to 4.6) | -3.7  (-11.2 to 3.7) |
| **Self-pay** | 371.6  (349.1 to 394.0) | -41.4  (-73.5 to -9.3) | -10.0  (-17.8 to -2.2) | 350.9  (327.2 to 374.7) | -20.7  (-53.3 to 12.0) | -5.6  (-14.4 to 3.2) |
| **No charge** | 517.0  (382.5 to 651.4) | -28.5  (-259.5 to 202.5) | -5.2  (-47.6 to 37.1) | 490.6  (375.7 to 605.6) | -26.3  (-203.2 to 150.5) | -5.1  (-39.3 to 29.1) |
| **Other** | 225.8  (203.1 to 248.5) | -38.1  (-78.9 to 2.8) | -14.4  (-29.9 to 1.1) | 198.0  (179.3 to 216.8) | -27.8  (-57.2 to 1.6) | -12.3  (-25.3 to 0.7) |
| **Patient Location^g^** |  |  |  |  |  |  |
| **"Central" counties of metro areas of >=1 million population** | 265.3  (247.6 to 282.9) | -9.4  (-35.1 to 16.2) | -3.4  (-12.8 to 5.9) | 254.0  (237.6 to 270.3) | -11.3  (-35.4 to 12.7) | -4.3  (-13.3 to 4.8) |
| **"Fringe" counties of metro areas of >=1 million population** | 217.5  (204.4 to 230.7) | -17.5  (-37.0 to 1.9) | -7.5  (-15.7 to 0.8) | 209.1  (196.2 to 222.0) | -8.5  (-26.9 to 9.9) | -3.9  (-12.4 to 4.6) |
| **Counties in metro areas of 250,000-999,999 population** | 231.2  (216.9 to 245.5) | -0.5  (-20.8 to 19.7) | -0.2  (-9.0 to 8.5) | 231.4  (217.2 to 245.6) | 0.2  (-19.9 to 20.3) | 0.1  (-8.6 to 8.8) |
| **Counties in metro areas of 50,000-249,999 population** | 206.5  (187.7 to 225.3) | -15.5  (-43.4 to 12.4) | -7.0  (-19.5 to 5.6) | 206.7  (187.6 to 225.7) | 0.1  (-26.6 to 26.9) | 0.1  (-12.9 to 13.0) |
| **Micropolitan counties** | 178.8  (168.0 to 189.5) | -19.1  (-37.2 to -1.0) | -9.7  (-18.8 to -0.5) | 183.5  (173.1 to 193.8) | 4.7  (-10.3 to 19.6) | 2.6  (-5.7 to 11.0) |
| **Not metropolitan or micropolitan counties** | 156.9  (135.7 to 178.1) | -12.0  (-41.8 to 17.9) | -7.1  (-24.8 to 10.6) | 165.9  (142.9 to 189.0) | 9.0  (-22.3 to 40.3) | 5.7  (-14.2 to 25.7) |

^a^ Cases of opioid-related abuse or dependence were identified following the definition proposed by the Healthcare Cost and Utilization Project (ICD-10-CM code used: found in Table S 1).

^b^ Reflects the overall or total difference between 2016 and 2019: absolute difference (2019 – 2016) and percentage change [(2019 – 2016)/2016 × 100].

^c^ Reflects the overall or total difference during the COVID-19 pandemic in 2020 and 2021: absolute difference (2021 – 2019) and percentage change [(2021 – 2019)/2019 × 100].

^d^ Race/ethnicity variable was obtained from the Agency for Healthcare Research and Quality, Healthcare Cost and Utilization Project; nationally weighted from all records in states with reliable race/ethnicity reporting, which is collected by self-report on admission to the hospital using fixed categories.

^e^ Median household income of residents in the patient’s zip code was updated annually; 2019 range for quartile 1 was less than $48,000; quartile 2, $48,000 to $60,999; quartile 3, $61,000 to $81,999; and quartile 4, at least $82,000; 2021 range for quartile 1 was less than $52,000; quartile 2, $52,000 to $65,999; quartile 3, $66,000 to $87,999; and quartile 4, at least $88,000.

^f^ “Medicare” includes both fee-for-service and managed care Medicare patients. “Medicaid” includes both fee-for-service and managed care Medicaid patients. “Private insurance” includes Blue Cross, commercial carriers, and private HMOs and PPOs. “Other” includes Worker's Compensation, CHAMPUS (Civilian Health and Medical Program of the Uniformed Services), CHAMPVA (Civilian Health and Medical Program of the Department of Veteran's Affairs), Title V, and other government programs.

^g^ “Patient location” is a six-category urban-rural classification scheme for U.S. counties developed by the National Center for Health Statistics (NCHS) especially for use in health care research. The classification emphasizes urban distinctions and is unique in differentiating between central and fringe counties of large metropolitan areas. Smaller metropolitan counties are subdivided by population. Non-metropolitan counties are divided simply into micropolitan and non-core categories.

**Supplementary Table S14. Opioid-Related Abuse or Dependence^a^ (2016 to 2019 and 2020 to 2021) in Nationwide Emergency Department Sample Overall and for Subtypes by Demographic Characteristics**

| **Characteristics** | **Between 2016 and 2019** | | | **During the COVID-19 pandemic in 2020 and 2021** | | |
| --- | --- | --- | --- | --- | --- | --- |
|  | **2019 Rate,**  **per 10,000** | **Absolute difference**  **from 2016-2019^b^** | **Change**  **from 2016-2019, %** | **2021 Rate,**  **per 10,000** | **Absolute difference**  **from 2019-2021^c^** | **Change**  **from 2019-2021, %** |
| **Overall** | 88.4  (82.0 to 94.9) | -4.0  (-12.7 to 4.8) | -4.3  (-13.8 to 5.2) | 95.5  (86.1 to 104.8) | 7.0  (-4.3 to 18.4) | 8.0  (-4.9 to 20.8) |
| **AGE** |  |  |  |  |  |  |
| **18-44** | 105.4  (97.3 to 113.5) | -4.3  (-15.6 to 7.0.) | -3.9  (-14.3 to 6.4) | 114.9  (102.7 to 127.0) | 9.4  (-5.2 to 24.1) | 9.0  (-4.9 to 22.8) |
| **45-64** | 105.8  (95.3 to 116.2) | -3.3  (-16.9 to 10.3) | -3.1  (-15.5 to 9.4) | 110.4  (98.1 to 122.6) | 4.6  (-11.5 to 20.7) | 4.3  (-10.9 to 19.5) |
| **65-84** | 46.3  (42.5 to 50.1) | 3.8  (-1.7 to 9.3) | 8.9  (-4.1 to 21.9) | 53.8  (49.0 to 58.7) | 7.6  (1.4 to 13.7) | 16.3  (3.0 to 29.6) |
| **≥85** | 12.5  (11.0 to 14.0) | -1.0  (-3.8 to 1.9) | -7.2  (-28.4 to 14) | 14.5  (12.5 to 16.4) | 1.9  (-0.5 to 4.4) | 15.5  (-4.1 to 35.1) |
| **Sex** |  |  |  |  |  |  |
| **Male** | 113.9  (104.9 to 123.0) | -4.2  (-16.2 to 7.9) | -3.5  (-13.7 to 6.7) | 124.6  (110.4 to 138.8) | 10.7  (-6.2 to 27.5) | 9.4  (-5.4 to 24.2) |
| **Female** | 68.8  (64.1 to 73.4) | -4.4  (-10.9 to 2.2) | -6.0  (-14.9 to 3.0) | 71.7  (66.0 to 77.4) | 2.9  (-4.5 to 10.2) | 4.2  (-6.5 to 14.9) |
| **Race/ethnicity^e^** |  |  |  |  |  |  |
| **Non-Hispanic White** | 104.4  (97.4 to 111.4) | NA^d^ | NA^d^ | 112.5  (102.9 to 122.0) | 8.1  (-3.8 to 19.9) | 7.7  (-3.6 to 19.1) |
| **Non-Hispanic Black** | 69.2  (54.2 to 84.2) | NA^d^ | NA^d^ | 77.0  (62.8 to 91.2) | 7.8  (-12.8 to 28.5) | 11.3  (-18.5 to 41.1) |
| **Hispanic** | 65.4  (55.5 to 75.2) | NA^d^ | NA^d^ | 69.5  (57.7 to 81.2) | 4.1  (-11.3 to 19.4) | 6.3  (-17.2 to 29.7) |
| **Non-Hispanic Asian or Pacific Islander** | 28.1  (19.0 to 37.2) | NA^d^ | NA^d^ | 26.9  (22.4 to 31.4) | -1.2  (-11.4 to 8.9) | -4.4  (-40.4 to 31.6) |
| **Native American** | 120.2  (94.2 to 146.2) | NA^d^ | NA^d^ | 154.1  (111.3 to 197.0) | 33.9  (-16.1 to 84.0) | 28.2  (-13.4 to 69.9) |
| **Other** | 71.7  (59.4 to 83.9) | NA^d^ | NA^d^ | 88.8  (72.8 to 104.7) | 17.1  (-3.0 to 37.2) | 23.9  (-4.1 to 51.9) |
| **Zip code income quartile^f^** |  |  |  |  |  |  |
| **1 (lowest)** | 90.7  (80.8 to 100.7) | 0.5  (-12.6 to 13.6) | 0.6  (-14.0 to 15.1) | 97.6  (88.6 to 106.7) | 6.9  (-6.5 to 20.3) | 7.6  (-7.2 to 22.4) |
| **2** | 82.6  (76.1 to 89.1) | -2.9  (-11.9 to 6.0) | -3.4  (-13.9 to 7.0) | 87.9  (80.0 to 95.8) | 5.3  (-4.9 to 15.5) | 6.4  (-5.9 to 18.8) |
| **3** | 86.8  (80.4 to 93.3) | -8.0  (-17.4 to 1.5) | -8.4  (-18.3 to 1.6) | 98.1  (78.5 to 117.7) | 11.3  (-9.4 to 31.9) | 13.0  (-10.8 to 36.7) |
| **4 (highest)** | 85.1  (77.7 to 92.4) | -13.8  (-27.0 to -0.6) | -14.0  (-27.3 to -0.6) | 86.5  (74.7 to 98.3) | 1.5  (-12.4 to 15.4) | 1.7  (-14.6 to 18.1) |
| **Primary expected payer^g^** |  |  |  |  |  |  |
| **Medicare** | 71.5  (66.5 to 76.5) | -4.5  (-12.0 to 3.1) | -5.9  (-15.8 to 4.1) | 78.0  (71.4 to 84.6) | 6.5  (-1.8 to 14.7) | 9.0  (-2.5 to 20.6) |
| **Medicaid** | 171.3  (154.5 to 188.1) | 10.7  (-10.6 to 32.1) | 6.7  (-6.6 to 20.0) | 175.5  (157.8 to 193.3) | 4.2  (-20.2 to 28.6) | 2.4  (-11.8 to 16.7) |
| **Private insurance** | 41.0  (37.5 to 44.4) | -12.4  (-18.2 to -6.6) | -23.3  (-34.2 to -12.3) | 43.1  (38.5 to 47.8) | 2.2  (-3.6 to 8.0) | 5.4  (-8.7 to 19.4) |
| **Self-pay** | 88.2  (78.4 to 98.1) | -3.8  (-16.3 to 8.7) | -4.1  (-17.8 to 9.5) | 107.3  (75.1 to 139.6) | 19.1  (-14.6 to 52.8) | 21.7  (-16.5 to 59.8) |
| **No charge** | 108.4  (82.9 to 133.9) | -10.1  (-49.9 to 29.7) | -8.5  (-42.1 to 25.1) | 136.6  (96.6 to 176.7) | 28.2  (-19.2 to 75.7) | 26.0  (-17.7 to 69.8) |
| **Other** | 63.7  (54.9 to 72.6) | -0.1  (-11.5 to 11.3) | -0.2  (-18.0 to 17.7) | 64.4  (55.9 to 72.9) | 0.7  (-11.6 to 12.9) | 1.0  (-18.2 to 20.3) |
| **Patient Location^h^** |  |  |  |  |  |  |
| **"Central" counties of metro areas of >=1 million population** | 103.1  (88.4 to 117.8) | 0.1  (-19.2 to 19.5) | 0.1  (-18.7 to 18.9) | 115.1  (92.4 to 137.7) | 11.9  (-15.0 to 38.9) | 11.6  (-14.6 to 37.7) |
| **"Fringe" counties of metro areas of >=1 million population** | 85.5  (76.9 to 94.2) | -18.9  (-34.1 to -3.7) | -18.1  (-32.7 to -3.6) | 87.8  (75.6 to 100.0) | 2.3  (-12.7 to 17.2) | 2.7  (-14.8 to 20.2) |
| **Counties in metro areas of 250,000-999,999 population** | 95.8  (82.9 to 108.8) | 1.0  (-16.1 to 18.1) | 1.1  (-16.9 to 19.1) | 92.0  (81.0 to 103.0) | -3.8  (-20.9 to 13.2) | -4.0  (-21.8 to 13.7) |
| **Counties in metro areas of 50,000-249,999 population** | 69.1  (61.1 to 77.1) | -3.7  (-17.5 to 10.1) | -5.1  (-24.0 to 13.8) | 99.7  (82.1 to 117.3) | 30.6  (11.3 to 49.9) | 44.4  (16.4 to 72.3) |
| **Micropolitan counties** | 64.5  (57.8 to 71.3) | -7.4  (-17.2 to 2.5) | -10.2  (-24.0 to 3.5) | 65.1  (57.8 to 72.4) | 0.6  (-9.3 to 10.5) | 0.9  (-14.5 to 16.3) |
| **Not metropolitan or micropolitan counties** | 52.0  (45.6 to 58.4) | -1.3  (-11.1 to 8.6) | -2.4  (-20.9 to 16.1) | 57.4  (50.1 to 64.8) | 5.4  (-4.3 to 15.2) | 10.4  (-8.3 to 29.2) |

^a^ Cases of opioid-related abuse or dependence were identified following the definition proposed by the Healthcare Cost and Utilization Project (ICD-10-CM code used: found in Table S 1).

^b^ Reflects the overall or total difference between 2016 and 2019: absolute difference (2019 – 2016) and percentage change [(2019 – 2016)/2016 × 100].

^c^ Reflects the overall or total difference during the COVID-19 pandemic in 2020 and 2021: absolute difference (2021 – 2019) and percentage change [(2021 – 2019)/2019 × 100].

^d^ NA, Not Available. Due to the NEDS survey design, the Race/ethnicity classification was not available until 2019 and therefore could not present estimates.

^e^ Race/ethnicity variable was obtained from the Agency for Healthcare Research and Quality, Healthcare Cost and Utilization Project; nationally weighted from all records in states with reliable race/ethnicity reporting, which is collected by self-report on admission to the hospital using fixed categories.

^f^ Median household income of residents in the patient’s zip code was updated annually; 2019 range for quartile 1 was less than $48,000; quartile 2, $48,000 to $60,999; quartile 3, $61,000 to $81,999; and quartile 4, at least $82,000; 2021 range for quartile 1 was less than $52,000; quartile 2, $52,000 to $65,999; quartile 3, $66,000 to $87,999; and quartile 4, at least $88,000.

^g^ “Medicare” includes both fee-for-service and managed care Medicare patients. “Medicaid” includes both fee-for-service and managed care Medicaid patients. “Private insurance” includes Blue Cross, commercial carriers, and private HMOs and PPOs. “Other” includes Worker's Compensation, CHAMPUS (Civilian Health and Medical Program of the Uniformed Services), CHAMPVA (Civilian Health and Medical Program of the Department of Veteran's Affairs), Title V, and other government programs.

^h^ “Patient location” is a six-category urban-rural classification scheme for U.S. counties developed by the National Center for Health Statistics (NCHS) especially for use in health care research. The classification emphasizes urban distinctions and is unique in differentiating between central and fringe counties of large metropolitan areas. Smaller metropolitan counties are subdivided by population. Non-metropolitan counties are divided simply into micropolitan and non-core categories.

**Supplementary Table S15. Opioid-Related Adverse Event or Poisoning^a^ (2016 to 2019 and 2020 to 2021) in National Inpatient Sample Overall and for Subtypes by Demographic Characteristics**

| **Characteristics** | **Between 2016 and 2019** | | | **During the COVID-19 pandemic in 2020 and 2021** | | |
| --- | --- | --- | --- | --- | --- | --- |
|  | **2019 Rate,**  **per 10,000** | **Absolute difference**  **from 2016-2019^b^** | **Change**  **from 2016-2019, %** | **2021 Rate,**  **per 10,000** | **Absolute difference**  **from 2019-2021^c^** | **Change**  **from 2019-2021, %** |
| **Overall** | 90.7  (88.5 to 92.9) | -8.9  (-12.0 to -5.7) | -8.9  (-12.0 to -5.8) | 83.6  (81.3 to 85.9) | -7.1  (-10.3 to -3.9) | -7.9  (-11.4 to -4.3) |
| **AGE** |  |  |  |  |  |  |
| **18-44** | 77.5  (74.9 to 80.2) | -6.5  (-10.2 to -2.7) | -7.7  (-12.2 to -3.2) | 78.8  (75.8 to 81.8) | 1.3  (-2.7 to 5.3) | 1.7  (-3.5 to 6.9) |
| **45-64** | 115.5  (112.0 to 118.9) | -10.3  (-15.1 to -5.5) | -8.2  (-12.0 to -4.4) | 105.1  (101.5 to 108.6) | -10.4  (-15.4 to -5.4) | -9.0  (-13.3 to -4.7) |
| **65-84** | 88.2  (85.8 to 90.6) | -9.4  (-12.9 to -5.9) | -9.7  (-13.3 to -6.1) | 78.0  (75.6 to 80.4) | -10.2  (-13.6 to -6.8) | -11.6  (-15.4 to -7.7) |
| **≥85** | 65.2  (62.7 to 67.7) | -7.6  (-11.4 to -3.8) | -10.4  (-15.6 to -5.2) | 54.3  (51.8 to 56.7) | -10.9  (-14.4 to -7.4) | -16.7  (-22.2 to -11.3) |
| **Sex** |  |  |  |  |  |  |
| **Male** | 93.8  (91.3 to 96.4) | -7.3  (-10.9 to -3.8) | -7.3  (-10.8 to -3.8) | 90.6  (87.9 to 93.4) | -3.2  (-6.9 to 0.6) | -3.4  (-7.4 to 0.6) |
| **Female** | 88.4  (86.1 to 90.6) | -10.1  (-13.3 to -6.8) | -10.2  (-13.6 to -6.9) | 78.0  (75.7 to 80.3) | -10.3  (-13.6 to -7.1) | -11.7  (-15.4 to -8.0) |
| **Race/ethnicity^d^** |  |  |  |  |  |  |
| **Non-Hispanic White** | 99.3  (97.1 to 101.6) | -12.4  (-15.7 to -9.1) | -11.1  (-14.0 to -8.2) | 88.7  (86.3 to 91.1) | -10.7  (-14.0 to -7.3) | -10.7  (-14.1 to -7.4) |
| **Non-Hispanic Black** | 82.2  (78.1 to 86.2) | 2.6  (-3.0 to 8.1) | 3.2  (-3.8 to 10.2) | 85.4  (80.8 to 90.1) | 3.2  (-2.9 to 9.4) | 3.9  (-3.6 to 11.4) |
| **Hispanic** | 64.8  (61.2 to 68.4) | 2.0  (-2.8 to 6.9) | 3.2  (-4.5 to 11.0) | 62.8  (59.1 to 66.4) | -2.0  (-7.1 to 3.1) | -3.1  (-11.0 to 4.8) |
| **Non-Hispanic Asian or Pacific Islander** | 51.8  (46.6 to 57.0) | -0.3  (-7.9 to 7.2) | -0.6  (-15.1 to 13.9) | 45.8  (41.0 to 50.6) | -6.0  (-13.1 to 1.1) | -11.5  (-25.2 to 2.2) |
| **Native American** | 93.0  (81.2 to 104.8) | -6.9  (-24.2 to 10.3) | -6.9  (-24.2 to 10.3) | 88.8  (75.0 to 102.6) | -4.2  (-22.3 to 14.0) | -4.5  (-24.0 to 15.0) |
| **Other** | 74.4  (69.0 to 79.7) | 2.7  (-6.0 to 11.4) | 3.7  (-8.4 to 15.9) | 77.4  (71.5 to 83.3) | 3.0  (-4.9 to 11.0) | 4.0  (-6.6 to 14.7) |
| **Zip code income quartile^e^** |  |  |  |  |  |  |
| **1 (lowest)** | 85.6  (82.8 to 88.4) | -7.2  (-11.2 to -3.2) | -7.8  (-12.1 to -3.5) | 82.9  (79.7 to 86.0) | -2.8  (-7.0 to 1.5) | -3.2  (-8.2 to 1.7) |
| **2** | 88.3  (85.9 to 90.8) | -11.6  (-15.2 to -7.9) | -11.6  (-15.2 to -7.9) | 80.3  (77.7 to 82.8) | -8.1  (-11.6 to -4.5) | -9.1  (-13.2 to -5.1) |
| **3** | 93.8  (91.1 to 96.6) | -9.6  (-13.6 to -5.7) | -9.3  (-13.1 to -5.5) | 83.9  (81.1 to 86.7) | -9.9  (-13.9 to -6.0) | -10.6  (-14.8 to -6.4) |
| **4 (highest)** | 96.5  (92.5 to 100.5) | -8.3  (-14.0 to -2.6) | -7.9  (-13.4 to -2.5) | 84.8  (81.2 to 88.4) | -11.7  (-17.1 to -6.3) | -12.1  (-17.7 to -6.5) |
| **Primary expected payer^f^** |  |  |  |  |  |  |
| **Medicare** | 94.1  (91.8 to 96.4) | -12.9  (-16.3 to -9.5) | -12.0  (-15.2 to -8.9) | 82.7  (80.3 to 85) | -11.5  (-14.7 to -8.2) | -12.2  (-15.7 to -8.7) |
| **Medicaid** | 101.3  (97.6 to 104.9) | -2.5  (-7.7 to 2.7) | -2.4  (-7.4 to 2.6) | 105.3  (101.2 to 109.4) | 4.1  (-1.4 to 9.6) | 4.0  (-1.4 to 9.4) |
| **Private insurance** | 74.6  (71.6 to 77.7) | -7.7  (-11.8 to -3.6) | -9.3  (-14.3 to -4.3) | 64.7  (61.7 to 67.8) | -9.9  (-14.2 to -5.6) | -13.3  (-19.0 to -7.5) |
| **Self-pay** | 106.1  (100.6 to 111.7) | -7.4  (-15.2 to 0.4) | -6.5  (-13.3 to 0.3) | 117.4  (111.2 to 123.6) | 11.3  (2.9 to 19.6) | 10.6  (2.8 to 18.4) |
| **No charge** | 136.6  (117.1 to 156.2) | 20.9  (-6.6 to 48.3) | 18.0  (-5.7 to 41.7) | 146.8  (123.2 to 170.4) | 10.2  (-20.5 to 40.8) | 7.4  (-15.0 to 29.8) |
| **Other** | 85.6  (80.6 to 90.7) | -10.4  (-18.7 to -2.1) | -10.8  (-19.5 to -2.2) | 75.7  (69.9 to 81.5) | -9.9  (-17.6 to -2.2) | -11.6  (-20.6 to -2.6) |
| **Patient Location^g^** |  |  |  |  |  |  |
| **"Central" counties of metro areas of >=1 million population** | 94.5  (90.3 to 98.6) | -2.3  (-8.1 to 3.6) | -2.3  (-8.4 to 3.7) | 90.9  (86.7 to 95.2) | -3.5  (-9.4 to 2.4) | -3.7  (-10.0 to 2.5) |
| **"Fringe" counties of metro areas of >=1 million population** | 95.2  (91.8 to 98.6) | -8.2  (-13.2 to -3.2) | -7.9  (-12.7 to -3.1) | 84.5  (80.9 to 88.2) | -10.7  (-15.7 to -5.7) | -11.2  (-16.5 to -6.0) |
| **Counties in metro areas of 250,000-999,999 population** | 89.6  (86.1 to 93.2) | -12.3  (-17.5 to -7.1) | -12.1  (-17.1 to -7.0) | 81.5  (78.0 to 85.0) | -8.1  (-13.1 to -3.1) | -9.1  (-14.6 to -3.5) |
| **Counties in metro areas of 50,000-249,999 population** | 87.0  (83.0 to 91.1) | -12.6  (-18.8 to -6.4) | -12.7  (-18.9 to -6.5) | 76.1  (72.1 to 80.1) | -11.0  (-16.7 to -5.3) | -12.6  (-19.1 to -6.0) |
| **Micropolitan counties** | 83.6  (79.8 to 87.4) | -16.5  (-22.3 to -10.7) | -16.5  (-22.3 to -10.7) | 75.4  (71.0 to 79.9) | -8.2  (-14.0 to -2.3) | -9.8  (-16.8 to -2.8) |
| **Not metropolitan or micropolitan counties** | 73.6  (69.7 to 77.4) | -16.1  (-21.5 to -10.6) | -17.9  (-24.0 to -11.9) | 66.4  (62.7 to 70.1) | -7.2  (-12.5 to -1.9) | -9.8  (-17.0 to -2.5) |

^a^ Cases of opioid-related adverse event or poisoning were identified following the definition proposed by the Healthcare Cost and Utilization Project (ICD-10-CM code used: found in Table S 1).

^b^ Reflects the overall or total difference between 2016 and 2019: absolute difference (2019 – 2016) and percentage change [(2019 – 2016)/2016 × 100].

^c^ Reflects the overall or total difference during the COVID-19 pandemic in 2020 and 2021: absolute difference (2021 – 2019) and percentage change [(2021 – 2019)/2019 × 100].

^d^ Race/ethnicity variable was obtained from the Agency for Healthcare Research and Quality, Healthcare Cost and Utilization Project; nationally weighted from all records in states with reliable race/ethnicity reporting, which is collected by self-report on admission to the hospital using fixed categories.

^e^ Median household income of residents in the patient’s zip code was updated annually; 2019 range for quartile 1 was less than $48,000; quartile 2, $48,000 to $60,999; quartile 3, $61,000 to $81,999; and quartile 4, at least $82,000; 2021 range for quartile 1 was less than $52,000; quartile 2, $52,000 to $65,999; quartile 3, $66,000 to $87,999; and quartile 4, at least $88,000.

^f^ “Medicare” includes both fee-for-service and managed care Medicare patients. “Medicaid” includes both fee-for-service and managed care Medicaid patients. “Private insurance” includes Blue Cross, commercial carriers, and private HMOs and PPOs. “Other” includes Worker's Compensation, CHAMPUS (Civilian Health and Medical Program of the Uniformed Services), CHAMPVA (Civilian Health and Medical Program of the Department of Veteran's Affairs), Title V, and other government programs.

^g^ “Patient location” is a six-category urban-rural classification scheme for U.S. counties developed by the National Center for Health Statistics (NCHS) especially for use in health care research. The classification emphasizes urban distinctions and is unique in differentiating between central and fringe counties of large metropolitan areas. Smaller metropolitan counties are subdivided by population. Non-metropolitan counties are divided simply into micropolitan and non-core categories.

**Supplementary Table S16. Opioid-Related Adverse Event or Poisoning^a^ (2016 to 2019 and 2020 to 2021) in Nationwide Emergency Department Sample Overall and for Subtypes by Demographic Characteristics**

| **Characteristics** | **Between 2016 and 2019** | | | **During the COVID-19 pandemic in 2020 and 2021** | | |
| --- | --- | --- | --- | --- | --- | --- |
|  | **2019 Rate,**  **per 10,000** | **Absolute difference**  **from 2016-2019^b^** | **Change**  **from 2016-2019, %** | **2021 Rate,**  **per 10,000** | **Absolute difference**  **from 2019-2021^c^** | **Change**  **from 2019-2021, %** |
| **Overall** | 37.8  (35.6 to 40.1) | -2.7  (-6.1 to 0.7) | -6.6  (-15.0 to 1.8) | 41.7  (39.5 to 43.9) | 3.9  (0.7 to 7.0) | 10.2  (1.8 to 18.6) |
| **AGE** |  |  |  |  |  |  |
| **18-44** | 37.4  (34.6 to 40.2) | -2.7  (-7.5 to 2.2) | -6.7  (-18.7 to 5.4) | 46.1  (43.4 to 48.9) | 8.7  (4.8 to 12.6) | 23.2  (12.7 to 33.7) |
| **45-64** | 40.4  (36.8 to 43.9) | -2.1  (-6.3 to 2.0) | -5.0  (-14.8 to 4.8) | 42.9  (39.6 to 46.2) | 2.5  (-2.3 to 7.3) | 6.2  (-5.8 to 18.2) |
| **65-84** | 36.9  (35.2 to 38.7) | -2.6  (-5.0 to -0.2) | -6.6  (-12.6 to -0.5) | 34.3  (32.6 to 36.0) | -2.7  (-5.1 to -0.2) | -7.2  (-13.8 to -0.6) |
| **≥85** | 30.7  (29.0 to 32.4) | -5.5  (-8.3 to -2.7) | -15.2  (-23.0 to -7.4) | 27.2  (25.4 to 29.0) | -3.5  (-6.0 to -1.0) | -11.4  (-19.4 to -3.4) |
| **Sex** |  |  |  |  |  |  |
| **Male** | 47.0  (43.3 to 50.6) | -1.9  (-7.1 to 3.3) | -3.8  (-14.5 to 6.8) | 54.4  (51.1 to 57.7) | 7.4  (2.5 to 12.3) | 15.7  (5.2 to 26.2) |
| **Female** | 30.8  (29.4 to 32.3) | -3.5  (-5.8 to -1.2) | -10.1  (-16.8 to -3.5) | 31.4  (30.0 to 32.8) | 0.6  (-1.5 to 2.6) | 1.8  (-4.7 to 8.3) |
| **Race/ethnicity^e^** |  |  |  |  |  |  |
| **Non-Hispanic White** | 45.7  (43.4 to 47.9) | NA^d^ | NA^d^ | 48.4  (46.1 to 50.7) | 2.7  (-0.5 to 5.9) | 6.0  (-1.0 to 12.9) |
| **Non-Hispanic Black** | 28.2  (23.1 to 33.2) | NA^d^ | NA^d^ | 34.1  (29.0 to 39.2) | 5.9  (-1.3 to 13.1) | 21.0  (-4.6 to 46.5) |
| **Hispanic** | 24.5  (21.7 to 27.4) | NA^d^ | NA^d^ | 28.8  (26.0 to 31.6) | 4.2  (0.2 to 8.2) | 17.1  (0.9 to 33.4) |
| **Non-Hispanic Asian or Pacific Islander** | 24.6  (12.5 to 36.8) | NA^d^ | NA^d^ | 19.1  (16.8 to 21.5) | -5.5  (-17.8 to 6.9) | -22.2  (-72.4 to 28.0) |
| **Native American** | 33.7  (26.3 to 41.2) | NA^d^ | NA^d^ | 72.7  (45.5 to 100.0) | 39.0  (10.8 to 67.2) | 115.6  (32.0 to 199.2) |
| **Other** | 28.0  (25.4 to 30.5) | NA^d^ | NA^d^ | 39.2  (35.3 to 43.0) | 11.2  (6.6 to 15.8) | 40.2  (23.7 to 56.7) |
| **Zip code income quartile^f^** |  |  |  |  |  |  |
| **1 (lowest)** | 35.0  (31.8 to 38.3) | -0.9  (-5.6 to 3.7) | -2.6  (-15.5 to 10.4) | 42.3  (38.8 to 45.8) | 7.3  (2.5 to 12.0) | 20.7  (7.1 to 34.4) |
| **2** | 36.0  (33.8 to 38.2) | -4.2  (-7.7 to -0.6) | -10.3  (-19.3 to -1.4) | 39.9  (37.6 to 42.3) | 4.0  (0.7 to 7.2) | 11.0  (2.1 to 19.9) |
| **3** | 39.9  (37.5 to 42.3) | -3.9  (-7.5 to -0.3) | -8.9  (-17.1 to -0.7) | 41.2  (38.7 to 43.8) | 1.4  (-2.1 to 4.8) | 3.4  (-5.3 to 12.1) |
| **4 (highest)** | 41.7  (38.7 to 44.6) | -3.6  (-8.0 to 0.9) | -7.9  (-17.8 to 2.0) | 40.2  (37.6 to 42.9) | -1.4  (-5.4 to 2.5) | -3.4  (-12.9 to 6.1) |
| **Primary expected payer^g^** |  |  |  |  |  |  |
| **Medicare** | 41.2  (39.4 to 43.0) | -5.1  (-7.7 to -2.6) | -11.1  (-16.6 to -5.5) | 38.7  (37.0 to 40.5) | -2.5  (-5.0 to 0) | -6.0  (-12.0 to 0.1) |
| **Medicaid** | 48.5  (43.4 to 53.5) | 1.2  (-5.6 to 8.0) | 2.6  (-11.8 to 17.0) | 57.8  (53.2 to 62.4) | 9.4  (2.6 to 16.1) | 19.3  (5.3 to 33.3) |
| **Private insurance** | 23.2  (22.0 to 24.5) | -4.9  (-7.0 to -2.8) | -17.5  (-24.9 to -10.1) | 24.5  (22.7 to 26.4) | 1.3  (-1.0 to 3.6) | 5.6  (-4.2 to 15.4) |
| **Self-pay** | 44.5  (39.4 to 49.5) | 0.9  (-6.8 to 8.6) | 2.1  (-15.6 to 19.8) | 61.2  (55.6 to 66.8) | 16.8  (9.3 to 24.3) | 37.8  (20.9 to 54.7) |
| **No charge** | 57.6  (42.5 to 72.8) | -18.6  (-93.3 to 56.2) | -24.4  (-122.6 to 73.8) | 89.1  (55.5 to 122.8) | 31.5  (-5.4 to 68.4) | 54.7  (-9.3 to 118.7) |
| **Other** | 29.2  (20.2 to 38.1) | -3.3  (-13.9 to 7.3) | -10.1  (-42.8 to 22.6) | 28.8  (24.8 to 32.8) | -0.3  (-10.1 to 9.5) | -1.1  (-34.6 to 32.5) |
| **Patient Location^h^** |  |  |  |  |  |  |
| **"Central" counties of metro areas of >=1 million population** | 39.0  (33.4 to 44.5) | 3.0  (-3.5 to 9.6) | 8.5  (-9.8 to 26.7) | 44.6  (39.9 to 49.4) | 5.7  (-1.7 to 13.0) | 14.5  (-4.2 to 33.3) |
| **"Fringe" counties of metro areas of >=1 million population** | 42.6  (38.8 to 46.3) | -5.7  (-12.3 to 0.9) | -11.8  (-25.4 to 1.9) | 43.7  (39.9 to 47.5) | 1.2  (-4.2 to 6.5) | 2.7  (-9.9 to 15.3) |
| **Counties in metro areas of 250,000-999,999 population** | 38.1  (34.6 to 41.6) | -8.5  (-16.2 to -0.8) | -18.2  (-34.7 to -1.6) | 43.5  (39.3 to 47.6) | 5.3  (-0.1 to 10.8) | 14.0  (-0.3 to 28.2) |
| **Counties in metro areas of 50,000-249,999 population** | 34.1  (31.1 to 37.1) | -1.2  (-6.2 to 3.9) | -3.3  (-17.5 to 10.9) | 39.3  (34.8 to 43.7) | 5.2  (-0.2 to 10.5) | 15.1  (-0.6 to 30.8) |
| **Micropolitan counties** | 32.3  (29.2 to 35.4) | -4.7  (-9.3 to -0.2) | -12.8  (-25.1 to -0.5) | 33.4  (30.2 to 36.6) | 1.0  (-3.4 to 5.5) | 3.2  (-10.6 to 17.0) |
| **Not metropolitan or micropolitan counties** | 25.7  (23.7 to 27.6) | -3.9  (-6.8 to -1.0) | -13.3  (-23.1 to -3.5) | 26.2  (24.3 to 28.1) | 0.5  (-2.2 to 3.2) | 2.0  (-8.6 to 12.6) |

^a^ Cases of opioid-related adverse event or poisoning were identified following the definition proposed by the Healthcare Cost and Utilization Project (ICD-10-CM code used: found in Table S 1).

^b^ Reflects the overall or total difference between 2016 and 2019: absolute difference (2019 – 2016) and percentage change [(2019 – 2016)/2016 × 100].

^c^ Reflects the overall or total difference during the COVID-19 pandemic in 2020 and 2021: absolute difference (2021 – 2019) and percentage change [(2021 – 2019)/2019 × 100].

^d^ NA, Not Available. Due to the NEDS survey design, the Race/ethnicity classification was not available until 2019 and therefore could not present estimates.

^e^ Race/ethnicity variable was obtained from the Agency for Healthcare Research and Quality, Healthcare Cost and Utilization Project; nationally weighted from all records in states with reliable race/ethnicity reporting, which is collected by self-report on admission to the hospital using fixed categories.

^f^ Median household income of residents in the patient’s zip code was updated annually; 2019 range for quartile 1 was less than $48,000; quartile 2, $48,000 to $60,999; quartile 3, $61,000 to $81,999; and quartile 4, at least $82,000; 2021 range for quartile 1 was less than $52,000; quartile 2, $52,000 to $65,999; quartile 3, $66,000 to $87,999; and quartile 4, at least $88,000.

^g^ “Medicare” includes both fee-for-service and managed care Medicare patients. “Medicaid” includes both fee-for-service and managed care Medicaid patients. “Private insurance” includes Blue Cross, commercial carriers, and private HMOs and PPOs. “Other” includes Worker's Compensation, CHAMPUS (Civilian Health and Medical Program of the Uniformed Services), CHAMPVA (Civilian Health and Medical Program of the Department of Veteran's Affairs), Title V, and other government programs.

^h^ “Patient location” is a six-category urban-rural classification scheme for U.S. counties developed by the National Center for Health Statistics (NCHS) especially for use in health care research. The classification emphasizes urban distinctions and is unique in differentiating between central and fringe counties of large metropolitan areas. Smaller metropolitan counties are subdivided by population. Non-metropolitan counties are divided simply into micropolitan and non-core categories.

**Supplementary Table S17. Synthetic opioids as a proxy for fentanyl Related Adverse Event or Poisoning^a^ (2016 to 2019 and 2020 to 2021) in National Inpatient Sample Overall and for Subtypes by Demographic Characteristics**

| **Characteristics** | **Between 2016 and 2019** | | | **During the COVID-19 pandemic in 2020 and 2021** | | |
| --- | --- | --- | --- | --- | --- | --- |
|  | **2019 Rate,**  **per 10,000** | **Absolute difference**  **from 2016-2019^b^** | **Change**  **from 2016-2019, %** | **2021 Rate,**  **per 10,000** | **Absolute difference**  **from 2019-2021^c^** | **Change**  **from 2019-2021, %** |
| **Overall** | 7.1  (6.8 to 7.4) | 0.6  (0.2 to 1.0) | 9.1  (3.1 to 15.1) | 9.2  (8.7 to 9.6) | 2.1  (1.6 to 2.6) | 29.6  (22.0 to 37.2) |
| **AGE** |  |  |  |  |  |  |
| **18-44** | 7.5  (6.9 to 8.1) | 2.2  (1.5 to 2.9) | 41.0  (28.2 to 53.8) | 13.8  (12.8 to 14.8) | 6.3  (5.2 to 7.5) | 84.4  (69.3 to 99.5) |
| **45-64** | 7.0  (6.6 to 7.5) | 0.7  (0.1 to 1.3) | 11.3  (1.6 to 20.9) | 9.4  (8.7 to 10.0) | 2.4  (1.6 to 3.2) | 33.6  (22.2 to 45.1) |
| **65-84** | 6.5  (6.1 to 6.8) | -0.4  (-0.9 to 0.1) | -5.7  (-13.4 to 2.0) | 5.7  (5.4 to 6.1) | -0.7  (-1.2 to -0.2) | -11.4  (-19.1 to -3.7) |
| **≥85** | 8.1  (7.3 to 8.9) | -1.1  (-2.2 to 0.1) | -11.8  (-24.1 to 0.6) | 6.7  (6.0 to 7.4) | -1.4  (-2.5 to -0.4) | -17.5  (-30.7 to -4.3) |
| **Sex** |  |  |  |  |  |  |
| **Male** | 7.2  (6.7 to 7.6) | 1.4  (0.9 to 1.9) | 24.8  (15.8 to 33.8) | 11.6  (10.8 to 12.3) | 4.4  (3.6 to 5.2) | 61.5  (49.9 to 73.2) |
| **Female** | 7.0  (6.7 to 7.3) | 0  (-0.5 to 0.5) | 0  (-6.5 to 6.4) | 7.3  (6.9 to 7.7) | 0.3  (-0.2 to 0.8) | 3.9  (-3.3 to 11.0) |
| **Race/ethnicity^d^** |  |  |  |  |  |  |
| **Non-Hispanic White** | 8.0  (7.6 to 8.3) | 0.5  (0.1 to 1.0) | 6.9  (0.7 to 13.0) | 9.8  (9.3 to 10.2) | 1.8  (1.2 to 2.4) | 22.9  (15.6 to 30.1) |
| **Non-Hispanic Black** | 5.3  (4.7 to 5.9) | 0.9  (0.2 to 1.7) | 21.6  (4.3 to 38.9) | 8.4  (7.4 to 9.4) | 3.1  (1.9 to 4.2) | 58.2  (36.3 to 80.1) |
| **Hispanic** | 5.1  (4.4 to 5.7) | 1.2  (0.4 to 2.0) | 31.0  (9.5 to 52.5) | 8.1  (7.2 to 8.9) | 3.0  (1.9 to 4.1) | 59.2  (37.8 to 80.5) |
| **Non-Hispanic Asian or Pacific Islander** | 4.3  (3.3 to 5.3) | 1.2  (-0.2 to 2.5) | 36.5  (-5.8 to 78.8) | 4.2  (3.1 to 5.2) | -0.1  (-1.6 to 1.3) | -2.8  (-36.6 to 31.1) |
| **Native American** | 8.2  (5.7 to 10.7) | -1.3  (-5.5 to 2.9) | -13.8  (-57.6 to 30.0) | 11.4  (7.6 to 15.1) | 3.2  (-1.3 to 7.7) | 38.8  (-16.1 to 93.7) |
| **Other** | 4.8  (3.6 to 6.0) | 0.8  (-0.8 to 2.3) | 18.4  (-18.9 to 55.7) | 7.5  (5.8 to 9.2) | 2.7  (0.6 to 4.7) | 54.9  (12.1 to 97.7) |
| **Zip code income quartile^e^** |  |  |  |  |  |  |
| **1 (lowest)** | 6.5  (6.1 to 7.0) | 0.4  (-0.2 to 1.0) | 7.1  (-2.7 to 16.9) | 9.8  (9.1 to 10.5) | 3.3  (2.4 to 4.1) | 50.2  (37.1 to 63.2) |
| **2** | 7.5  (7.0 to 8.0) | 0.7  (0 to 1.4) | 10.0  (0 to 20.0) | 8.7  (8.1 to 9.3) | 1.3  (0.5 to 2.0) | 16.7  (6.3 to 27.1) |
| **3** | 7.2  (6.7 to 7.7) | 0.4  (-0.2 to 1.1) | 6.5  (-3.5 to 16.6) | 8.7  (8.1 to 9.3) | 1.5  (0.7 to 2.3) | 20.6  (9.7 to 31.4) |
| **4 (highest)** | 6.9  (6.3 to 7.4) | 0.6  (-0.1 to 1.3) | 9.4  (-2.3 to 21.1) | 7.7  (7.1 to 8.3) | 0.8  (0 to 1.6) | 12.2  (0.5 to 24.0) |
| **Primary expected payer^f^** |  |  |  |  |  |  |
| **Medicare** | 7.3  (6.9 to 7.6) | -0.6  (-1.2 to -0.1) | -8.1  (-14.6 to -1.6) | 6.7  (6.4 to 7.1) | -0.5  (-1.0 to 0) | -7.2  (-14.0 to -0.3) |
| **Medicaid** | 8.8  (8.0 to 9.6) | 2.9  (2.0 to 3.8) | 49.7  (33.9 to 65.4) | 16.3  (15.1 to 17.6) | 7.6  (6.0 to 9.1) | 85.8  (68.6 to 103.0) |
| **Private insurance** | 4.8  (4.4 to 5.2) | 0.2  (-0.3 to 0.7) | 5.0  (-6.2 to 16.3) | 5.9  (5.5 to 6.4) | 1.2  (0.6 to 1.7) | 24.0  (11.8 to 36.1) |
| **Self-pay** | 11.2  (9.7 to 12.7) | 5.0  (3.2 to 6.9) | 80.9  (50.9 to 111.0) | 22.8  (20.0 to 25.5) | 11.6  (8.4 to 14.7) | 103.2  (74.9 to 131.6) |
| **No charge** | 11.5  (6.4 to 16.5) | 6.4  (0.5 to 12.3) | 126.7  (9.5 to 243.8) | 30.4  (20.0 to 40.7) | 18.9  (7.3 to 30.4) | 164.6  (64.0 to 265.2) |
| **Other** | 6.8  (5.4 to 8.1) | 0.8  (-1.0 to 2.6) | 12.7  (-17.2 to 42.6) | 8.1  (6.7 to 9.6) | 1.4  (-0.6 to 3.3) | 20.4  (-8.5 to 49.4) |
| **Patient Location^g^** |  |  |  |  |  |  |
| **"Central" counties of metro areas of >=1 million population** | 6.3  (5.7 to 6.8) | 1.1  (0.4 to 1.8) | 21.8  (8.7 to 34.9) | 9.6  (8.7 to 10.6) | 3.4  (2.2 to 4.5) | 53.8  (35.9 to 71.8) |
| **"Fringe" counties of metro areas of >=1 million population** | 7.4  (6.8 to 7.9) | 1.4  (0.7 to 2.1) | 24.0  (12.1 to 35.9) | 8.9  (8.2 to 9.7) | 1.6  (0.6 to 2.5) | 21.0  (8.5 to 33.5) |
| **Counties in metro areas of 250,000-999,999 population** | 7.1  (6.5 to 7.8) | -0.6  (-1.4 to 0.3) | -7.3  (-18.1 to 3.5) | 8.9  (8.2 to 9.6) | 1.8  (0.9 to 2.7) | 24.8  (12.0 to 37.6) |
| **Counties in metro areas of 50,000-249,999 population** | 7.7  (6.9 to 8.5) | 0.4  (-0.7 to 1.5) | 5.5  (-9.6 to 20.6) | 8.2  (7.3 to 9.1) | 0.5  (-0.7 to 1.7) | 6.8  (-8.6 to 22.2) |
| **Micropolitan counties** | 7.6  (6.8 to 8.4) | -0.4  (-1.5 to 0.7) | -5.3  (-19.1 to 8.6) | 8.4  (7.5 to 9.3) | 0.8  (-0.4 to 2.0) | 11.1  (-4.7 to 26.8) |
| **Not metropolitan or micropolitan counties** | 6.8  (5.9 to 7.7) | -0.4  (-1.6 to 0.8) | -5.4  (-22.3 to 11.5) | 6.4  (5.6 to 7.2) | -0.5  (-1.7 to 0.8) | -6.6  (-24.3 to 11.1) |

^a^ Cases of synthetic opioids as a proxy for fentanyl Related adverse event or poisoning were identified following the definition proposed by the Healthcare Cost and Utilization Project (ICD-10-CM code used: found in Table S 1).

^b^ Reflects the overall or total difference between 2016 and 2019: absolute difference (2019 – 2016) and percentage change [(2019 – 2016)/2016 × 100].

^c^ Reflects the overall or total difference during the COVID-19 pandemic in 2020 and 2021: absolute difference (2021 – 2019) and percentage change [(2021 – 2019)/2019 × 100].

^d^ Race/ethnicity variable was obtained from the Agency for Healthcare Research and Quality, Healthcare Cost and Utilization Project; nationally weighted from all records in states with reliable race/ethnicity reporting, which is collected by self-report on admission to the hospital using fixed categories.

^e^ Median household income of residents in the patient’s zip code was updated annually; 2019 range for quartile 1 was less than $48,000; quartile 2, $48,000 to $60,999; quartile 3, $61,000 to $81,999; and quartile 4, at least $82,000; 2021 range for quartile 1 was less than $52,000; quartile 2, $52,000 to $65,999; quartile 3, $66,000 to $87,999; and quartile 4, at least $88,000.

^f^ “Medicare” includes both fee-for-service and managed care Medicare patients. “Medicaid” includes both fee-for-service and managed care Medicaid patients. “Private insurance” includes Blue Cross, commercial carriers, and private HMOs and PPOs. “Other” includes Worker's Compensation, CHAMPUS (Civilian Health and Medical Program of the Uniformed Services), CHAMPVA (Civilian Health and Medical Program of the Department of Veteran's Affairs), Title V, and other government programs.

^g^ “Patient location” is a six-category urban-rural classification scheme for U.S. counties developed by the National Center for Health Statistics (NCHS) especially for use in health care research. The classification emphasizes urban distinctions and is unique in differentiating between central and fringe counties of large metropolitan areas. Smaller metropolitan counties are subdivided by population. Non-metropolitan counties are divided simply into micropolitan and non-core categories.

**Supplementary Table S18. Synthetic opioids as a proxy for fentanyl Related Adverse Event or Poisoning^a^ (2016 to 2019 and 2020 to 2021) in Nationwide Emergency Department Sample Overall and for Subtypes by Demographic Characteristics**

| **Characteristics** | **Between 2016 and 2019** | | | **During the COVID-19 pandemic in 2020 and 2021** | | |
| --- | --- | --- | --- | --- | --- | --- |
|  | **2019 Rate,**  **per 10,000** | **Absolute difference**  **from 2016-2019^b^** | **Change**  **from 2016-2019, %** | **2021 Rate,**  **per 10,000** | **Absolute difference**  **from 2019-2021^c^** | **Change**  **from 2019-2021, %** |
| **Overall** | 3.2  (3.0 to 3.5) | 0.5  (0.2 to 0.8) | 17.9  (7.3 to 28.4) | 5.4  (4.9 to 5.8) | 2.1  (1.6 to 2.7) | 65.9  (49.4 to 82.5) |
| **AGE** |  |  |  |  |  |  |
| **18-44** | 3.2  (2.8 to 3.6) | 1.1  (0.6 to 1.5) | 48.6  (29.4 to 67.8) | 7.5  (6.7 to 8.2) | 4.3  (3.4 to 5.1) | 132.4  (106.2 to 158.6) |
| **45-64** | 2.8  (2.6 to 3.1) | 0.1  (-0.2 to 0.4) | 2.2  (-9.0 to 13.4) | 4.1  (3.7 to 4.6) | 1.3  (0.8 to 1.8) | 45.9  (27.6 to 64.2) |
| **65-84** | 3.4  (3.2 to 3.6) | -0.1  (-0.5 to 0.2) | -3.6  (-13.0 to 5.7) | 3.1  (2.9 to 3.3) | -0.4  (-0.7 to 0) | -10.2  (-19.0 to -1.3) |
| **≥85** | 4.7  (4.3 to 5.2) | -0.4  (-1.1 to 0.3) | -7.5  (-20.8 to 5.9) | 3.5  (3.1 to 3.9) | -1.2  (-1.9 to -0.6) | -26.1  (-39.3 to -12.8) |
| **Sex** |  |  |  |  |  |  |
| **Male** | 3.6  (3.2 to 4.0) | 1.1  (0.6 to 1.5) | 42.4  (24.8 to 59.9) | 7.4  (6.6 to 8.1) | 3.7  (2.9 to 4.6) | 102.8  (79.3 to 126.2) |
| **Female** | 2.9  (2.7 to 3.1) | 0.03  (-0.2 to 0.3) | 1.0  (-7.1 to 9.2) | 3.7  (3.5 to 4.0) | 0.8  (0.5 to 1.1) | 27.7  (17.1 to 38.4) |
| **Race/ethnicity^e^** |  |  |  |  |  |  |
| **Non-Hispanic White** | 4.1  (3.7 to 4.4) | NA^d^ | NA^d^ | 6.5  (6.0 to 7.1) | 2.5  (1.8 to 3.1) | 60.2  (44.4 to 76.0) |
| **Non-Hispanic Black** | 1.9  (1.5 to 2.3) | NA^d^ | NA^d^ | 3.1  (2.5 to 3.7) | 1.2  (0.4 to 2.0) | 63.2  (23.7 to 102.6) |
| **Hispanic** | 2.2  (1.9 to 2.5) | NA^d^ | NA^d^ | 4.2  (3.5 to 4.9) | 2.0  (1.2 to 2.8) | 90.5  (55.6 to 125.5) |
| **Non-Hispanic Asian or Pacific Islander** | 1.5  (1.2 to 1.9) | NA^d^ | NA^d^ | 2.0  (1.5 to 2.5) | 0.5  (-0.1 to 1.1) | 30.1  (-9.1 to 69.3) |
| **Native American** | 2.6  (1.8 to 3.5) | NA^d^ | NA^d^ | 14.2  (7.3 to 21.1) | 11.6  (4.6 to 18.5) | 440.3  (176.1 to 704.5) |
| **Other** | 2.0  (1.7 to 2.4) | NA^d^ | NA^d^ | 3.9  (3.2 to 4.6) | 1.8  (1.0 to 2.6) | 88.8  (50.3 to 127.3) |
| **Zip code income quartile^f^** |  |  |  |  |  |  |
| **1 (lowest)** | 2.9  (2.6 to 3.2) | 0.5  (0.2 to 0.9) | 21.5  (6.3 to 36.7) | 5.2  (4.6 to 5.8) | 2.3  (1.7 to 2.9) | 79.9  (57.3 to 102.4) |
| **2** | 3.3  (2.9 to 3.6) | 0.3  (-0.1 to 0.7) | 10.9  (-2.9 to 24.7) | 5.2  (4.7 to 5.8) | 2.0  (1.4 to 2.6) | 61.2  (41.7 to 80.8) |
| **3** | 3.5  (3.2 to 3.8) | 0.4  (0 to 0.8) | 14.0  (0.7 to 27.3) | 5.3  (4.7 to 5.9) | 1.8  (1.1 to 2.5) | 52.0  (32.7 to 71.3) |
| **4 (highest)** | 3.4  (3.0 to 3.8) | 0.6  (0.1 to 1.0) | 20.6  (4.0 to 37.1) | 4.8  (4.2 to 5.5) | 1.4  (0.7 to 2.2) | 41.8  (19.5 to 64.0) |
| **Primary expected payer^g^** |  |  |  |  |  |  |
| **Medicare** | 3.9  (3.7 to 4.1) | -0.2  (-0.6 to 0.1) | -5.8  (-13.9 to 2.3) | 3.8  (3.5 to 4.0) | -0.1  (-0.5 to 0.2) | -3.4  (-12.0 to 5.3) |
| **Medicaid** | 3.8  (3.3 to 4.3) | 1.4  (0.8 to 2.0) | 57.7  (33.2 to 82.1) | 8.3  (7.4 to 9.3) | 4.5  (3.4 to 5.6) | 119.5  (90.4 to 148.6) |
| **Private insurance** | 2.1  (1.9 to 2.3) | -0.1  (-0.3 to 0.2) | -2.3  (-13.5 to 8.8) | 3.1  (2.8 to 3.5) | 1.1  (0.6 to 1.5) | 50.2  (30.1 to 70.4) |
| **Self-pay** | 3.4  (2.7 to 4.0) | 1.4  (0.7 to 2.1) | 75.4  (39.0 to 111.8) | 8.8  (7.4 to 10.3) | 5.5  (3.9 to 7.1) | 163.9  (117.3 to 210.5) |
| **No charge** | 3.6  (2.3 to 4.8) | 2.4  (1.0 to 3.8) | 195.9  (80.7 to 311.1) | 16.0  (7.7 to 24.2) | 12.4  (4.0 to 20.7) | 345.5  (112.4 to 578.7) |
| **Other** | 2.6  (2.1 to 3.2) | 0.5  (-0.1 to 1.1) | 21.8  (-6.7 to 50.2) | 3.9  (3.2 to 4.5) | 1.2  (0.4 to 2.0) | 46.4  (15.2 to 77.6) |
| **Patient Location^h^** |  |  |  |  |  |  |
| **"Central" counties of metro areas of >=1 million population** | 2.9  (2.5 to 3.2) | 0.7  (0.2 to 1.1) | 30.1  (10.0 to 50.2) | 5.2  (4.4 to 6.1) | 2.4  (1.4 to 3.3) | 83.2  (50.8 to 115.5) |
| **"Fringe" counties of metro areas of >=1 million population** | 3.7  (3.1 to 4.3) | 1.0  (0.3 to 1.7) | 35.9  (10.9 to 60.9) | 5.6  (4.7 to 6.6) | 1.9  (0.8 to 3.1) | 52.0  (21.4 to 82.7) |
| **Counties in metro areas of 250,000-999,999 population** | 3.4  (2.8 to 3.9) | 0.3  (-0.3 to 1.0) | 11.2  (-9.3 to 31.8) | 5.6  (4.8 to 6.5) | 2.3  (1.2 to 3.3) | 67.1  (36.7 to 97.4) |
| **Counties in metro areas of 50,000-249,999 population** | 3.2  (2.8 to 3.6) | 0.2  (-0.3 to 0.7) | 7.3  (-9.8 to 24.5) | 4.9  (4.2 to 5.5) | 1.6  (0.9 to 2.4) | 50.9  (26.9 to 74.9) |
| **Micropolitan counties** | 2.9  (2.6 to 3.3) | -0.2  (-0.7 to 0.3) | -6.4  (-21.2 to 8.3) | 4.6  (3.8 to 5.3) | 1.7  (0.8 to 2.5) | 57.4  (29.1 to 85.7) |
| **Not metropolitan or micropolitan counties** | 2.9  (2.6 to 3.1) | -0.5  (-1 to 0) | -13.9  (-28.6 to 0.9) | 3.6  (3.1 to 4.0) | 0.7  (0.2 to 1.2) | 24.8  (6.0 to 43.6) |

^a^ Cases of synthetic opioids as a proxy for fentanyl related adverse event or poisoning were identified following the definition proposed by the Healthcare Cost and Utilization Project (ICD-10-CM code used: found in Table S 1).

^b^ Reflects the overall or total difference between 2016 and 2019: absolute difference (2019 – 2016) and percentage change [(2019 – 2016)/2016 × 100].

^c^ Reflects the overall or total difference during the COVID-19 pandemic in 2020 and 2021: absolute difference (2021 – 2019) and percentage change [(2021 – 2019)/2019 × 100].

^d^ NA, Not Available. Due to the NEDS survey design, the Race/ethnicity classification was not available until 2019 and therefore could not present estimates.

^e^ Race/ethnicity variable was obtained from the Agency for Healthcare Research and Quality, Healthcare Cost and Utilization Project; nationally weighted from all records in states with reliable race/ethnicity reporting, which is collected by self-report on admission to the hospital using fixed categories.

^f^ Median household income of residents in the patient’s zip code was updated annually; 2019 range for quartile 1 was less than $48,000; quartile 2, $48,000 to $60,999; quartile 3, $61,000 to $81,999; and quartile 4, at least $82,000; 2021 range for quartile 1 was less than $52,000; quartile 2, $52,000 to $65,999; quartile 3, $66,000 to $87,999; and quartile 4, at least $88,000.

^g^ “Medicare” includes both fee-for-service and managed care Medicare patients. “Medicaid” includes both fee-for-service and managed care Medicaid patients. “Private insurance” includes Blue Cross, commercial carriers, and private HMOs and PPOs. “Other” includes Worker's Compensation, CHAMPUS (Civilian Health and Medical Program of the Uniformed Services), CHAMPVA (Civilian Health and Medical Program of the Department of Veteran's Affairs), Title V, and other government programs.

^h^ “Patient location” is a six-category urban-rural classification scheme for U.S. counties developed by the National Center for Health Statistics (NCHS) especially for use in health care research. The classification emphasizes urban distinctions and is unique in differentiating between central and fringe counties of large metropolitan areas. Smaller metropolitan counties are subdivided by population. Non-metropolitan counties are divided simply into micropolitan and non-core categories.

**Supplementary Table S19.** **Prescription natural/semisynthetic opioids as a proxy for opioid pain medications Related Adverse Event or Poisoning^a^ (2016 to 2019 and 2020 to 2021) in National Inpatient Sample Overall and for Subtypes by Demographic Characteristics**

| **Characteristics** | **Between 2016 and 2019** | | | | **During the COVID-19 pandemic in 2020 and 2021** | | |
| --- | --- | --- | --- | --- | --- | --- | --- |
|  | **2019 Rate,**  **per 10,000** | **Absolute difference**  **from 2016-2019^b^** | **Change**  **from 2016-2019, %** | **2021 Rate,**  **per 10,000** | | **Absolute difference**  **from 2019-2021^c^** | **Change**  **from 2019-2021, %** |
| **Overall** | 50.8  (49.0 to 52.6) | 1.7  (-0.7 to 4.0) | 3.4  (-1.4 to 8.1) | 48.5  (46.6 to 50.4) | | -2.3  (-5.0 to 0.3) | -4.6  (-9.8 to 0.6) |
| **AGE** |  |  |  |  | |  |  |
| **18-44** | 36.9  (35.0 to 38.7) | 2.3  (-0.1 to 4.6) | 6.5  (-0.3 to 13.4) | 39.3  (37.2 to 41.5) | | 2.5  (-0.3 to 5.3) | 6.8  (-0.9 to 14.4) |
| **45-64** | 67.0  (64.1 to 70.0) | 0.9  (-2.8 to 4.7) | 1.4  (-4.3 to 7.0) | 63.1  (60.0 to 66.1) | | -4.0  (-8.2 to 0.2) | -6.0  (-12.3 to 0.4) |
| **65-84** | 52.6  (50.7 to 54.5) | 1.6  (-0.9 to 4.1) | 3.1  (-1.8 to 8.0) | 48.5  (46.5 to 50.5) | | -4.1  (-6.8 to -1.3) | -7.7  (-12.9 to -2.5) |
| **≥85** | 37.3  (35.4 to 39.1) | 2.3  (-0.3 to 4.9) | 6.5  (-0.9 to 13.9) | 33.1  (31.2 to 35.0) | | -4.2  (-6.8 to -1.6) | -11.2  (-18.3 to -4.2) |
| **Sex** |  |  |  |  | |  |  |
| **Male** | 50.1  (48.0 to 52.2) | 3.2  (0.6 to 5.9) | 6.9  (1.2 to 12.6) | 49.5  (47.2 to 51.7) | | -0.7  (-3.7 to 2.4) | -1.3  (-7.4 to 4.8) |
| **Female** | 51.4  (49.6 to 53.1) | 0.5  (-1.8 to 2.9) | 1.1  (-3.6 to 5.7) | 47.7  (45.8 to 49.7) | | -3.6  (-6.3 to -1.0) | -7.1  (-12.2 to -2.0) |
| **Race/ethnicity^d^** |  |  |  |  | |  |  |
| **Non-Hispanic White** | 55.0  (53.2 to 56.9) | 0.4  (-2.1 to 2.8) | 0.7  (-3.8 to 5.1) | 51.0  (48.9 to 53.0) | | -4.1  (-6.8 to -1.3) | -7.4  (-12.4 to -2.3) |
| **Non-Hispanic Black** | 48.0  (45.0 to 51.1) | 7.4  (3.5 to 11.3) | 18.2  (8.6 to 27.9) | 51.2  (47.8 to 54.6) | | 3.2  (-1.4 to 7.7) | 6.6  (-3.0 to 16.1) |
| **Hispanic** | 35.9  (33.2 to 38.6) | 5.1  (1.7 to 8.4) | 16.5  (5.6 to 27.4) | 36.7  (33.7 to 39.6) | | 0.8  (-3.2 to 4.8) | 2.1  (-8.9 to 13.2) |
| **Non-Hispanic Asian or Pacific Islander** | 33.6  (29.4 to 37.8) | 4.1  (-1.8 to 9.9) | 13.9  (-6.0 to 33.7) | 29.9  (26.1 to 33.7) | | -3.7  (-9.3 to 2.0) | -11.0  (-27.8 to 5.9) |
| **Native American** | 53.4  (44.7 to 62.2) | 3.9  (-7.6 to 15.5) | 7.9  (-15.4 to 31.2) | 50.1  (40.6 to 59.6) | | -3.4  (-16.3 to 9.6) | -6.3  (-30.5 to 17.9) |
| **Other** | 42.1  (38.1 to 46.0) | 6.1  (-0.3 to 12.5) | 17.0  (-0.8 to 34.8) | 44.8  (40.4 to 49.3) | | 2.8  (-3.2 to 8.7) | 6.6  (-7.5 to 20.7) |
| **Zip code income quartile^e^** |  |  |  |  | |  |  |
| **1 (lowest)** | 47.0  (44.9 to 49.1) | 2.3  (-0.5 to 5.0) | 5.1  (-1.1 to 11.3) | 46.5  (44.1 to 48.9) | | -0.5  (-3.7 to 2.7) | -1.1  (-7.9 to 5.7) |
| **2** | 48.4  (46.5 to 50.3) | -1.2  (-3.8 to 1.4) | -2.5  (-7.7 to 2.7) | 46.2  (44.2 to 48.2) | | -2.2  (-5.0 to 0.6) | -4.5  (-10.2 to 1.2) |
| **3** | 53.7  (51.5 to 55.9) | 2.1  (-0.7 to 4.9) | 4.1  (-1.4 to 9.6) | 49.8  (47.4 to 52.1) | | -4.0  (-7.1 to -0.8) | -7.4  (-13.3 to -1.4) |
| **4 (highest)** | 56.6  (53.2 to 59.9) | 3.8  (-0.4 to 8.0) | 7.2  (-0.8 to 15.2) | 52.3  (49.2 to 55.4) | | -4.3  (-8.9 to 0.3) | -7.6  (-15.7 to 0.4) |
| **Primary expected payer^f^** |  |  |  |  | |  |  |
| **Medicare** | 55.6  (53.8 to 57.4) | 0.2  (-2.2 to 2.7) | 0.4  (-4.0 to 4.8) | 50.9  (49.0 to 52.8) | | -4.7  (-7.3 to -2.1) | -8.5  (-13.1 to -3.8) |
| **Medicaid** | 49.2  (46.7 to 51.7) | 4.5  (1.1 to 7.8) | 9.9  (2.5 to 17.4) | 53.5  (50.6 to 56.5) | | 4.3  (0.4 to 8.2) | 8.8  (0.9 to 16.7) |
| **Private insurance** | 45.1  (42.5 to 47.6) | 1.9  (-1.3 to 5.1) | 4.4  (-3.1 to 11.9) | 41.2  (38.5 to 43.8) | | -3.9  (-7.6 to -0.2) | -8.7  (-16.9 to -0.5) |
| **Self-pay** | 40.9  (37.9 to 43.9) | 0.1  (-4.0 to 4.2) | 0.2  (-9.8 to 10.3) | 48.2  (44.6 to 51.7) | | 7.3  (2.6 to 11.9) | 17.7  (6.4 to 29.1) |
| **No charge** | 50.6  (39.4 to 61.9) | 9.8  (-5.5 to 25.0) | 23.8  (-13.3 to 61.0) | 55.2  (43.1 to 67.3) | | 4.5  (-12.0 to 21.1) | 9.0  (-23.7 to 41.7) |
| **Other** | 48.8  (44.9 to 52.8) | 1.9  (-4.0 to 7.7) | 4.0  (-8.4 to 16.4) | 45.9  (41.1 to 50.6) | | -3.0  (-9.1 to 3.2) | -6.1  (-18.7 to 6.5) |
| **Patient Location^g^** |  |  |  |  | |  |  |
| **"Central" counties of metro areas of >=1 million population** | 54.5  (51.2 to 57.9) | 5.8  (1.5 to 10.1) | 11.9  (3.0 to 20.7) | 53.7  (50.4 to 57.0) | | -0.8  (-5.5 to 3.9) | -1.5  (-10.2 to 7.2) |
| **"Fringe" counties of metro areas of >=1 million population** | 51.9  (49.4 to 54.5) | 2.7  (-0.6 to 6.1) | 5.5  (-1.2 to 12.3) | 49.4  (46.3 to 52.6) | | -2.5  (-6.6 to 1.5) | -4.8  (-12.6 to 3.0) |
| **Counties in metro areas of 250,000-999,999 population** | 49.9  (47.4 to 52.5) | 0  (-3.5 to 3.5) | 0  (-7.0 to 7.0) | 46.7  (44.2 to 49.2) | | -3.2  (-6.8 to 0.3) | -6.5  (-13.6 to 0.6) |
| **Counties in metro areas of 50,000-249,999 population** | 48.4  (45.4 to 51.3) | -0.4  (-4.4 to 3.6) | -0.8  (-9.0 to 7.5) | 43.6  (40.7 to 46.5) | | -4.8  (-8.9 to -0.6) | -9.9  (-18.5 to -1.3) |
| **Micropolitan counties** | 47.8  (45.0 to 50.6) | -3.1  (-7.2 to 1.0) | -6.0  (-14.1 to 2.1) | 43.7  (39.9 to 47.5) | | -4.1  (-8.8 to 0.7) | -8.5  (-18.5 to 1.4) |
| **Not metropolitan or micropolitan counties** | 42.2  (39.2 to 45.1) | -4.8  (-8.7 to -0.9) | -10.2  (-18.6 to -1.9) | 39.4  (36.5 to 42.4) | | -2.7  (-6.9 to 1.5) | -6.5  (-16.4 to 3.5) |

^a^ Cases of prescription natural/semisynthetic opioids as a proxy for opioid pain medications related adverse event or poisoning were identified following the definition proposed by the Healthcare Cost and Utilization Project (ICD-10-CM code used: found in Table S 1).

^b^ Reflects the overall or total difference between 2016 and 2019: absolute difference (2019 – 2016) and percentage change [(2019 – 2016)/2016 × 100].

^c^ Reflects the overall or total difference during the COVID-19 pandemic in 2020 and 2021: absolute difference (2021 – 2019) and percentage change [(2021 – 2019)/2019 × 100].

^d^ Race/ethnicity variable was obtained from the Agency for Healthcare Research and Quality, Healthcare Cost and Utilization Project; nationally weighted from all records in states with reliable race/ethnicity reporting, which is collected by self-report on admission to the hospital using fixed categories.

^e^ Median household income of residents in the patient’s zip code was updated annually; 2019 range for quartile 1 was less than $48,000; quartile 2, $48,000 to $60,999; quartile 3, $61,000 to $81,999; and quartile 4, at least $82,000; 2021 range for quartile 1 was less than $52,000; quartile 2, $52,000 to $65,999; quartile 3, $66,000 to $87,999; and quartile 4, at least $88,000.

^f^ “Medicare” includes both fee-for-service and managed care Medicare patients. “Medicaid” includes both fee-for-service and managed care Medicaid patients. “Private insurance” includes Blue Cross, commercial carriers, and private HMOs and PPOs. “Other” includes Worker's Compensation, CHAMPUS (Civilian Health and Medical Program of the Uniformed Services), CHAMPVA (Civilian Health and Medical Program of the Department of Veteran's Affairs), Title V, and other government programs.

^g^ “Patient location” is a six-category urban-rural classification scheme for U.S. counties developed by the National Center for Health Statistics (NCHS) especially for use in health care research. The classification emphasizes urban distinctions and is unique in differentiating between central and fringe counties of large metropolitan areas. Smaller metropolitan counties are subdivided by population. Non-metropolitan counties are divided simply into micropolitan and non-core categories.

**Supplementary Table S20. Prescription natural/semisynthetic opioids as a proxy for opioid pain medications Related Adverse Event or Poisoning^a^ (2016 to 2019 and 2020 to 2021) in Nationwide Emergency Department Sample Overall and for Subtypes by Demographic Characteristics**

| **Characteristics** | **Between 2016 and 2019** | | | **During the COVID-19 pandemic in 2020 and 2021** | | |
| --- | --- | --- | --- | --- | --- | --- |
|  | **2019 Rate,**  **per 10,000** | **Absolute difference**  **from 2016-2019^b^** | **Change**  **from 2016-2019, %** | **2021 Rate,**  **per 10,000** | **Absolute difference**  **from 2019-2021^c^** | **Change**  **from 2019-2021, %** |
| **Overall** | 15.6  (14.7 to 16.5) | 0.4  (-0.7 to 1.6) | 2.8  (-4.6 to 10.3) | 17.5  (16.6 to 18.4) | 1.9  (0.6 to 3.2) | 12.2  (3.9 to 20.4) |
| **AGE** |  |  |  |  |  |  |
| **18-44** | 10.5  (9.8 to 11.2) | 0.5  (-0.4 to 1.4) | 5.1  (-4.0 to 14.2) | 14.8  (13.8 to 15.8) | 4.3  (3.1 to 5.5) | 40.7  (29.4 to 52.1) |
| **45-64** | 18.9  (17.5 to 20.4) | -0.3  (-2.0 to 1.4) | -1.6  (-10.4 to 7.3) | 19.5  (18.3 to 20.7) | 0.6  (-1.3 to 2.5) | 3.2  (-6.8 to 13.2) |
| **65-84** | 21.6  (20.5 to 22.7) | 0.2  (-1.3 to 1.7) | 1.1  (-6.0 to 8.2) | 20.7  (19.5 to 21.8) | -1.0  (-2.6 to 0.7) | -4.4  (-12.0 to 3.2) |
| **≥85** | 17.1  (15.9 to 18.2) | -1.0  (-2.8 to 0.8) | -5.6  (-15.6 to 4.4) | 16.4  (15.0 to 17.8) | -0.7  (-2.5 to 1.1) | -4.0  (-14.6 to 6.6) |
| **Sex** |  |  |  |  |  |  |
| **Male** | 16.5  (15.2 to 17.8) | 1.4  (-0.1 to 2.9) | 9.0  (-0.9 to 18.9) | 20.0  (18.8 to 21.2) | 3.5  (1.8 to 5.3) | 21.5  (10.8 to 32.2) |
| **Female** | 14.9  (14.2 to 15.7) | -0.3  (-1.3 to 0.8) | -1.8  (-8.8 to 5.1) | 15.5  (14.6 to 16.3) | 0.5  (-0.6 to 1.6) | 3.4  (-4.1 to 10.9) |
| **Race/ethnicity^e^** |  |  |  |  |  |  |
| **Non-Hispanic White** | 18.5  (17.7 to 19.3) | NA^d^ | NA^d^ | 19.9  (19.0 to 20.9) | 1.4  (0.2 to 2.7) | 7.7  (0.8 to 14.6) |
| **Non-Hispanic Black** | 11.7  (9.9 to 13.6) | NA^d^ | NA^d^ | 14.5  (12.9 to 16.1) | 2.8  (0.4 to 5.2) | 23.7  (3.1 to 44.3) |
| **Hispanic** | 10.8  (9.4 to 12.2) | NA^d^ | NA^d^ | 12.7  (11.4 to 14.1) | 2.0  (0 to 4.0) | 18.3  (-0.1 to 36.7) |
| **Non-Hispanic Asian or Pacific Islander** | 13.4  (8.2 to 18.7) | NA^d^ | NA^d^ | 11.5  (9.6 to 13.4) | -1.9  (-7.5 to 3.7) | -14.4  (-56.0 to 27.3) |
| **Native American** | 13.1  (10.6 to 15.6) | NA^d^ | NA^d^ | 26.4  (16.6 to 36.2) | 13.3  (3.2 to 23.4) | 101.8  (24.7 to 178.8) |
| **Other** | 11.7  (10.5 to 12.9) | NA^d^ | NA^d^ | 15.9  (14.2 to 17.6) | 4.2  (2.1 to 6.3) | 36.1  (18.1 to 54.1) |
| **Zip code income quartile^f^** |  |  |  |  |  |  |
| **1 (lowest)** | 13.6  (12.4 to 14.8) | 0.8  (-0.7 to 2.3) | 6.3  (-5.1 to 17.8) | 16.3  (15.1 to 17.4) | 2.7  (1.0 to 4.3) | 19.6  (7.3 to 31.9) |
| **2** | 14.6  (13.8 to 15.5) | -0.6  (-1.8 to 0.6) | -4.2  (-12.0 to 3.6) | 16.6  (15.5 to 17.7) | 2.0  (0.6 to 3.3) | 13.3  (4.0 to 22.7) |
| **3** | 17.1  (16.0 to 18.1) | 0.3  (-1.1 to 1.7) | 1.6  (-6.7 to 9.9) | 18.2  (16.9 to 19.5) | 1.2  (-0.5 to 2.8) | 6.9  (-2.8 to 16.7) |
| **4 (highest)** | 19.0  (17.5 to 20.6) | 1.1  (-0.9 to 3.0) | 6.0  (-4.9 to 17) | 19.8  (18.3 to 21.3) | 0.7  (-1.4 to 2.9) | 3.9  (-7.4 to 15.2) |
| **Primary expected payer^g^** |  |  |  |  |  |  |
| **Medicare** | 23.1  (21.9 to 24.3) | -0.6  (-2.2 to 1.0) | -2.4  (-9.1 to 4.4) | 22.4  (21.2 to 23.7) | -0.6  (-2.4 to 1.1) | -2.8  (-10.3 to 4.7) |
| **Medicaid** | 15.1  (13.4 to 16.8) | 1.9  (0.1 to 3.8) | 14.6  (0.4 to 28.9) | 18.7  (17.4 to 20.0) | 3.6  (1.5 to 5.7) | 23.9  (10.0 to 37.8) |
| **Private insurance** | 11.4  (10.7 to 12.1) | -0.7  (-1.7 to 0.3) | -5.6  (-13.7 to 2.4) | 12.3  (11.3 to 13.3) | 0.9  (-0.3 to 2.2) | 8.2  (-2.7 to 19.0) |
| **Self-pay** | 10.2  (9.1 to 11.3) | 1.5  (0.2 to 2.8) | 17.4  (2.5 to 32.3) | 17.4  (15.1 to 19.8) | 7.2  (4.7 to 9.8) | 70.9  (45.6 to 96.2) |
| **No charge** | 14.1  (11.0 to 17.2) | 3.2  (-1.6 to 8.0) | 29.5  (-14.4 to 73.4) | 25.9  (17.5 to 34.3) | 11.8  (2.9 to 20.8) | 84.2  (20.5 to 147.9) |
| **Other** | 10.6  (9.5 to 11.8) | -0.4  (-1.9 to 1.1) | -3.5  (-17.1 to 10.0) | 11.8  (10.6 to 13.0) | 1.1  (-0.5 to 2.8) | 10.7  (-4.9 to 26.4) |
| **Patient Location^h^** |  |  |  |  |  |  |
| **"Central" counties of metro areas of >=1 million population** | 16.7  (14.4 to 18.9) | 2.8  (0.2 to 5.5) | 20.5  (1.5 to 39.6) | 19.6  (17.8 to 21.4) | 2.9  (0 to 5.8) | 17.5  (0.1 to 34.8) |
| **"Fringe" counties of metro areas of >=1 million population** | 16.1  (14.7 to 17.5) | 0.1  (-1.8 to 1.9) | 0.4  (-11.2 to 12.1) | 18.0  (16.5 to 19.6) | 2.0  (-0.1 to 4.0) | 12.1  (-0.9 to 25.2) |
| **Counties in metro areas of 250,000-999,999 population** | 15.6  (14.3 to 17.0) | -1.1  (-3.1 to 1.0) | -6.3  (-18.3 to 5.8) | 17.5  (15.7 to 19.3) | 1.9  (-0.3 to 4.1) | 12.0  (-2.2 to 26.2) |
| **Counties in metro areas of 50,000-249,999 population** | 14.5  (13.2 to 15.8) | 0  (-1.8 to 1.9) | 0.3  (-12.7 to 13.3) | 16.4  (14.6 to 18.2) | 1.9  (-0.3 to 4.0) | 12.8  (-2.3 to 27.8) |
| **Micropolitan counties** | 14.3  (13.1 to 15.5) | -1.5  (-3.3 to 0.3) | -9.3  (-20.9 to 2.2) | 14.6  (13.3 to 16.0) | 0.4  (-1.5 to 2.2) | 2.5  (-10.3 to 15.2) |
| **Not metropolitan or micropolitan counties** | 12.8  (11.6 to 14.0) | -1.3  (-2.9 to 0.3) | -9.2  (-20.7 to 2.2) | 11.7  (10.7 to 12.7) | -1.1  (-2.6 to 0.5) | -8.5  (-20.6 to 3.5) |

^a^ Cases of prescription natural/semisynthetic opioids as a proxy for opioid pain medications related adverse event or poisoning were identified following the definition proposed by the Healthcare Cost and Utilization Project (ICD-10-CM code used: found in Table S 1).

^b^ Reflects the overall or total difference between 2016 and 2019: absolute difference (2019 – 2016) and percentage change [(2019 – 2016)/2016 × 100].

^c^ Reflects the overall or total difference during the COVID-19 pandemic in 2020 and 2021: absolute difference (2021 – 2019) and percentage change [(2021 – 2019)/2019 × 100].

^d^ NA, Not Available. Due to the NEDS survey design, the Race/ethnicity classification was not available until 2019 and therefore could not present estimates.

^e^ Race/ethnicity variable was obtained from the Agency for Healthcare Research and Quality, Healthcare Cost and Utilization Project; nationally weighted from all records in states with reliable race/ethnicity reporting, which is collected by self-report on admission to the hospital using fixed categories.

^f^ Median household income of residents in the patient’s zip code was updated annually; 2019 range for quartile 1 was less than $48,000; quartile 2, $48,000 to $60,999; quartile 3, $61,000 to $81,999; and quartile 4, at least $82,000; 2021 range for quartile 1 was less than $52,000; quartile 2, $52,000 to $65,999; quartile 3, $66,000 to $87,999; and quartile 4, at least $88,000.

^g^ “Medicare” includes both fee-for-service and managed care Medicare patients. “Medicaid” includes both fee-for-service and managed care Medicaid patients. “Private insurance” includes Blue Cross, commercial carriers, and private HMOs and PPOs. “Other” includes Worker's Compensation, CHAMPUS (Civilian Health and Medical Program of the Uniformed Services), CHAMPVA (Civilian Health and Medical Program of the Department of Veteran's Affairs), Title V, and other government programs.

^h^ “Patient location” is a six-category urban-rural classification scheme for U.S. counties developed by the National Center for Health Statistics (NCHS) especially for use in health care research. The classification emphasizes urban distinctions and is unique in differentiating between central and fringe counties of large metropolitan areas. Smaller metropolitan counties are subdivided by population. Non-metropolitan counties are divided simply into micropolitan and non-core categories.

**Supplementary Table S21. Heroin-Related Adverse Event or Poisoning^a^ (2016 to 2019 and 2020 to 2021) in National Inpatient Sample Overall and for Subtypes by Demographic Characteristics**

| **Characteristics** | **Between 2016 and 2019** | | | | **During the COVID-19 pandemic in 2020 and 2021** | | |
| --- | --- | --- | --- | --- | --- | --- | --- |
|  | **2019 Rate,**  **per 10,000** | **Absolute difference**  **from 2016-2019^b^** | **Change**  **from 2016-2019, %** | **2021 Rate,**  **per 10,000** | | **Absolute difference**  **from 2019-2021^c^** | **Change**  **from 2019-2021, %** |
| **Overall** | 7.5  (7.2 to 7.9) | -0.8  (-1.4 to -0.2) | -9.6  (-16.6 to -2.6) | 6.7  (6.3 to 7.1) | | -0.8  (-1.4 to -0.3) | -11.0  (-18.3 to -3.7) |
| **AGE** |  |  |  |  | |  |  |
| **18-44** | 18.0  (17.0 to 19.0) | -2.9  (-4.4 to -1.4) | -14.0  (-21.1 to -6.9) | 14.1  (13.3 to 14.9) | | -3.9  (-5.2 to -2.6) | -21.7  (-28.8 to -14.6) |
| **45-64** | 8.1  (7.4 to 8.8) | 0.9  (-0.1 to 1.8) | 11.8  (-1.7 to 25.2) | 7.9  (7.1 to 8.7) | | -0.2  (-1.2 to 0.9) | -2.1  (-15.2 to 11.0) |
| **65-84** | 0.8  (0.7 to 10.0) | 0.3  (0.1 to 0.5) | 51.8  (16.8 to 86.8) | 1.3  (1.1 to 1.6) | | 0.5  (0.2 to 0.8) | 57.6  (26.3 to 89.0) |
| **≥85** | 0.07  (0 to 0.1) | -0.1  (-0.2 to 0.1) | -41.7  (-146.3 to 62.9) | 0.1  (0 to 0.3) | | 0.1  (-0.1 to 0.2) | 100.0  (-101.9 to 301.9) |
| **Sex** |  |  |  |  | |  |  |
| **Male** | 12.1  (11.4 to 12.7) | -1.7  (-2.7 to -0.7) | -12.5  (-19.8 to -5.3) | 10.9  (10.2 to 11.5) | | -1.2  (-2.1 to -0.3) | -9.9  (-17.6 to -2.2) |
| **Female** | 4.1  (3.8 to 4.4) | -0.3  (-0.7 to 0.1) | -6.3  (-16.1 to 3.4) | 3.5  (3.2 to 3.7) | | -0.7  (-1.1 to -0.3) | -16.2  (-25.9 to -6.5) |
| **Race/ethnicity^d^** |  |  |  |  | |  |  |
| **Non-Hispanic White** | 7.6  (7.2 to 8.0) | -1.7  (-2.3 to -1.0) | -17.9  (-24.8 to -11.0) | 6.3  (5.9 to 6.6) | | -1.4  (-1.9 to -0.8) | -17.8  (-25.0 to -10.7) |
| **Non-Hispanic Black** | 8.6  (7.3 to 9.8) | 1.7  (-0.1 to 3.4) | 24.3  (-0.9 to 49.5) | 9.3  (8.0 to 10.7) | | 0.8  (-1.0 to 2.6) | 9.2  (-12.0 to 30.5) |
| **Hispanic** | 7.0  (6.1 to 7.8) | 0.3  (-0.9 to 1.5) | 4.5  (-13.8 to 22.8) | 6.1  (5.3 to 6.9) | | -0.8  (-2.0 to 0.3) | -12.1  (-28.8 to 4.7) |
| **Non-Hispanic Asian or Pacific Islander** | 1.7  (1.0 to 2.4) | 0.6  (-0.3 to 1.5) | 55.0  (-25.9 to 136.0) | 1.1  (0.6 to 1.6) | | -0.6  (-1.4 to 0.3) | -33.7  (-85.2 to 17.8) |
| **Native American** | 9.2  (5.5 to 12.9) | 2.2  (-2.6 to 7.0) | 31.4  (-36.9 to 99.8) | 7.8  (4.8 to 10.9) | | -1.4  (-6.1 to 3.4) | -14.8  (-66.8 to 37.2) |
| **Other** | 9.6  (7.8 to 11.4) | 2.8  (0.4 to 5.1) | 40.1  (6.1 to 74.0) | 9.7  (7.5 to 11.9) | | 0.1  (-2.7 to 2.9) | 0.9  (-28.4 to 30.3) |
| **Zip code income quartile^e^** |  |  |  |  | |  |  |
| **1 (lowest)** | 8.9  (8.1 to 9.7) | -0.4  (-1.5 to 0.7) | -4.3  (-16.5 to 7.9) | 8.8  (7.9 to 9.6) | | -0.2  (-1.3 to 1.0) | -1.8  (-14.5 to 11.0) |
| **2** | 6.9  (6.4 to 7.4) | -0.6  (-1.3 to 0.2) | -7.4  (-17.6 to 2.9) | 6.4  (5.9 to 6.9) | | -0.5  (-1.2 to 0.3) | -6.7  (-17.3 to 4.0) |
| **3** | 6.9  (6.3 to 7.4) | -0.9  (-1.7 to -0.1) | -11.3  (-21.5 to -1.0) | 5.8  (5.4 to 6.3) | | -1.0  (-1.7 to -0.3) | -14.6  (-24.9 to -4.3) |
| **4 (highest)** | 5.8  (5.2 to 6.4) | -2.5  (-3.4 to -1.7) | -30.4  (-40.9 to -19.9) | 4.0  (3.6 to 4.4) | | -1.8  (-2.5 to -1.1) | -30.5  (-42.6 to -18.4) |
| **Primary expected payer^f^** |  |  |  |  | |  |  |
| **Medicare** | 1.8  (1.6 to 2.0) | 0  (-0.3 to 0.3) | -0.5  (-15.0 to 13.9) | 2.0  (1.8 to 2.3) | | 0.2  (-0.1 to 0.5) | 12.7  (-3.4 to 28.8) |
| **Medicaid** | 20.9  (19.5 to 22.3) | -0.8  (-2.8 to 1.2) | -3.8  (-13.0 to 5.5) | 17.8  (16.6 to 19.1) | | -3.1  (-5.0 to -1.2) | -14.7  (-23.8 to -5.7) |
| **Private insurance** | 4.2  (3.8 to 4.6) | -2.1  (-2.8 to -1.5) | -33.9  (-44.7 to -23.0) | 3.3  (2.8 to 3.7) | | -0.9  (-1.5 to -0.3) | -22.0  (-35.9 to -8.1) |
| **Self-pay** | 33.3  (30.3 to 36.3) | -0.1  (-4.2 to 4.0) | -0.3  (-12.6 to 12.1) | 29.4  (26.7 to 32.0) | | -3.9  (-7.9 to 0.1) | -11.7  (-23.8 to 0.3) |
| **No charge** | 44.9  (33.5 to 56.3) | 1.3  (-14.6 to 17.1) | 2.9  (-33.5 to 39.2) | 38.1  (28.5 to 47.7) | | -6.8  (-21.8 to 8.1) | -15.2  (-48.4 to 18.0) |
| **Other** | 7.4  (6.0 to 8.9) | -1.8  (-4.0 to 0.3) | -19.8  (-43.0 to 3.4) | 4.3  (3.3 to 5.2) | | -3.2  (-4.9 to -1.4) | -42.8  (-66.4 to -19.2) |
| **Patient Location^g^** |  |  |  |  | |  |  |
| **"Central" counties of metro areas of >=1 million population** | 8.9  (8.0 to 9.8) | -0.4  (-1.7 to 0.9) | -4.3  (-18.4 to 9.9) | 8.2  (7.3 to 9.1) | | -0.7  (-2.0 to 0.6) | -7.9  (-22.5 to 6.8) |
| **"Fringe" counties of metro areas of >=1 million population** | 8.4  (7.6 to 9.1) | -2.2  (-3.2 to -1.1) | -20.5  (-30.9 to -10.0) | 6.2  (5.7 to 6.8) | | -2.1  (-3.0 to -1.2) | -25.6  (-36.5 to -14.7) |
| **Counties in metro areas of 250,000-999,999 population** | 7.6  (6.8 to 8.3) | -0.9  (-2.0 to 0.3) | -10.1  (-23.3 to 3.1) | 7.0  (6.2 to 7.7) | | -0.6  (-1.6 to 0.4) | -7.8  (-21.4 to 5.8) |
| **Counties in metro areas of 50,000-249,999 population** | 6.0  (5.1 to 6.8) | -0.6  (-1.8 to 0.7) | -8.5  (-27.9 to 11.0) | 5.8  (4.9 to 6.6) | | -0.2  (-1.4 to 1.0) | -3.2  (-23.2 to 16.8) |
| **Micropolitan counties** | 3.9  (3.3 to 4.5) | -1.2  (-2.1 to -0.3) | -24.1  (-41.5 to -6.7) | 4.3  (3.7 to 5.0) | | 0.5  (-0.4 to 1.4) | 12.4  (-10.2 to 35.0) |
| **Not metropolitan or micropolitan counties** | 2.4  (1.9 to 2.9) | 0.05  (-0.7 to 0.8) | 2.1  (-28.1 to 32.4) | 3.0  (2.4 to 3.7) | | 0.7  (-0.1 to 1.4) | 27.2  (-5.6 to 60.0) |

^a^ Cases of heroin-related adverse event or poisoning were identified following the definition proposed by the Healthcare Cost and Utilization Project (ICD-10-CM code used: found in Table S 1).

^b^ Reflects the overall or total difference between 2016 and 2019: absolute difference (2019 – 2016) and percentage change [(2019 – 2016)/2016 × 100].

^c^ Reflects the overall or total difference during the COVID-19 pandemic in 2020 and 2021: absolute difference (2021 – 2019) and percentage change [(2021 – 2019)/2019 × 100].

^d^ Race/ethnicity variable was obtained from the Agency for Healthcare Research and Quality, Healthcare Cost and Utilization Project; nationally weighted from all records in states with reliable race/ethnicity reporting, which is collected by self-report on admission to the hospital using fixed categories.

^e^ Median household income of residents in the patient’s zip code was updated annually; 2019 range for quartile 1 was less than $48,000; quartile 2, $48,000 to $60,999; quartile 3, $61,000 to $81,999; and quartile 4, at least $82,000; 2021 range for quartile 1 was less than $52,000; quartile 2, $52,000 to $65,999; quartile 3, $66,000 to $87,999; and quartile 4, at least $88,000.

^f^ “Medicare” includes both fee-for-service and managed care Medicare patients. “Medicaid” includes both fee-for-service and managed care Medicaid patients. “Private insurance” includes Blue Cross, commercial carriers, and private HMOs and PPOs. “Other” includes Worker's Compensation, CHAMPUS (Civilian Health and Medical Program of the Uniformed Services), CHAMPVA (Civilian Health and Medical Program of the Department of Veteran's Affairs), Title V, and other government programs.

^g^ “Patient location” is a six-category urban-rural classification scheme for U.S. counties developed by the National Center for Health Statistics (NCHS) especially for use in health care research. The classification emphasizes urban distinctions and is unique in differentiating between central and fringe counties of large metropolitan areas. Smaller metropolitan counties are subdivided by population. Non-metropolitan counties are divided simply into micropolitan and non-core categories.

**Supplementary Table S22. Heroin-Related Adverse Event or Poisoning^a^ (2016 to 2019 and 2020 to 2021) in** **Nationwide Emergency Department Sample Overall and for Subtypes by Demographic Characteristics**

| **Characteristics** | **Between 2016 and 2019** | | | | **During the COVID-19 pandemic in 2020 and 2021** | | |
| --- | --- | --- | --- | --- | --- | --- | --- |
|  | **2019 Rate,**  **per 10,000** | **Absolute difference**  **from 2016-2019^b^** | **Change**  **from 2016-2019, %** | **2021 Rate,**  **per 10,000** | | **Absolute difference**  **from 2019-2021^c^** | **Change**  **from 2019-2021, %** |
| **Overall** | 10.7  (9.5 to 11.8) | -2.1  (-4.3 to 0.2) | -16.1  (-33.6 to 1.3) | 9.8  (8.5 to 11.1) | | -0.8  (-2.6 to 0.9) | -7.8  (-24.2 to 8.6) |
| **AGE** |  |  |  |  | |  |  |
| **18-44** | 17.5  (15.7 to 19.3) | -4.2  (-7.9 to -0.4) | -19.2  (-36.3 to -2.0) | 14.7  (13.3 to 16.0) | | -2.8  (-5.0 to -0.6) | -16.1  (-28.9 to -3.3) |
| **45-64** | 8.7  (7.2 to 10.2) | 0.7  (-1.4 to 2.8) | 9.2  (-17.1 to 35.4) | 9.8  (7.3 to 12.3) | | 1.1  (-1.8 to 4.0) | 12.4  (-21.0 to 45.8) |
| **65-84** | 1.3  (1.0 to 1.7) | 0.6  (0.2 to 1.0) | 90.0  (32.3 to 147.7) | 2.1  (1.4 to 2.9) | | 0.8  (-0.1 to 1.6) | 58.6  (-4.0 to 121.3) |
| **≥85** | 0.2  (0.1 to 0.4) | 0.1  (-0.1 to 0.3) | 76.9  (-44.6 to 198.5) | 0.3  (0.2 to 0.5) | | 0.1  (-0.1 to 0.3) | 47.8  (-42.8 to 138.4) |
| **Sex** |  |  |  |  | |  |  |
| **Male** | 17.0  (15.1 to 18.8) | -3.5  (-7.0 to -0.1) | -17.2  (-34.0 to -0.4) | 15.5  (13.4 to 17.6) | | -1.4  (-4.2 to 1.4) | -8.4  (-24.9 to 8.0) |
| **Female** | 5.8  (5.1 to 6.5) | -1.1  (-2.4 to 0.2) | -16.0  (-34.5 to 2.5) | 5.2  (4.6 to 5.8) | | -0.6  (-1.5 to 0.3) | -10.7  (-26.6 to 5.3) |
| **Race/ethnicity^e^** |  |  |  |  | |  |  |
| **Non-Hispanic White** | 13.0  (11.6 to 14.4) | NA^d^ | NA^d^ | 11.2  (10.2 to 12.2) | | -1.8  (-3.5 to -0.1) | -14.0  (-27.2 to -0.8) |
| **Non-Hispanic Black** | 8.3  (5.8 to 10.8) | NA^d^ | NA^d^ | 9.6  (5.5 to 13.8) | | 1.3  (-3.5 to 6.2) | 16.1  (-42.1 to 74.4) |
| **Hispanic** | 6.3  (5.4 to 7.2) | NA^d^ | NA^d^ | 5.9  (4.7 to 7.1) | | -0.4  (-1.9 to 1.1) | -6.2  (-30.2 to 17.7) |
| **Non-Hispanic Asian or Pacific Islander** | 3.5  (0.9 to 6.1) | NA^d^ | NA^d^ | 1.5  (1.1 to 1.9) | | -2.0  (-4.6 to 0.6) | -57.1  (-132.6 to 18.5) |
| **Native American** | 11.1  (5.2 to 16.9) | NA^d^ | NA^d^ | 17.5  (9.5 to 25.5) | | 6.4  (-3.5 to 16.3) | 57.9  (-31.3 to 147.0) |
| **Other** | 8.0  (6.4 to 9.6) | NA^d^ | NA^d^ | 11.4  (8.5 to 14.2) | | 3.4  (0.1 to 6.6) | 42.0  (1.2 to 82.8) |
| **Zip code income quartile^f^** |  |  |  |  | |  |  |
| **1 (lowest)** | 10.8  (9.2 to 12.5) | -1.5  (-4.5 to 1.6) | -12.0  (-36.6 to 12.7) | 12.0  (9.3 to 14.6) | | 1.2  (-2.0 to 4.3) | 10.7  (-18.3 to 39.8) |
| **2** | 10.1  (9.0 to 11.3) | -1.7  (-4.0 to 0.7) | -14.1  (-34.0 to 5.8) | 9.2  (8.2 to 10.2) | | -0.9  (-2.4 to 0.6) | -9.2  (-24.0 to 5.7) |
| **3** | 10.6  (9.4 to 11.8) | -2.6  (-4.9 to -0.4) | -19.7  (-36.7 to -2.8) | 8.8  (7.8 to 9.7) | | -1.9  (-3.4 to -0.3) | -17.4  (-31.9 to -3.0) |
| **4 (highest)** | 9.9  (8.7 to 11.2) | -3.5  (-5.9 to -1.1) | -26.1  (-44.1 to -8.1) | 6.9  (6.0 to 7.7) | | -3.1  (-4.6 to -1.6) | -30.9  (-46.2 to -15.6) |
| **Primary expected payer^g^** |  |  |  |  | |  |  |
| **Medicare** | 2.8  (2.4 to 3.3) | -0.1  (-0.7 to 0.6) | -2.8  (-24.7 to 19.2) | 3.2  (2.6 to 3.8) | | 0.4  (-0.4 to 1.1) | 13.8  (-12.4 to 40.1) |
| **Medicaid** | 20.6  (18.0 to 23.2) | -2.0  (-6.6 to 2.5) | -9.0  (-29.0 to 11.0) | 19.4  (16.0 to 22.7) | | -1.2  (-5.4 to 3.0) | -6.0  (-26.5 to 14.4) |
| **Private insurance** | 4.6  (4.1 to 5.1) | -2.8  (-3.9 to -1.6) | -37.9  (-53.6 to -22.1) | 3.9  (3.3 to 4.6) | | -0.6  (-1.4 to 0.2) | -13.6  (-31.3 to 4.1) |
| **Self-pay** | 23.2  (20.1 to 26.3) | -2.9  (-8.5 to 2.7) | -11.1  (-32.7 to 10.4) | 21.4  (19.1 to 23.8) | | -1.8  (-5.6 to 2.1) | -7.6  (-24.1 to 8.9) |
| **No charge** | 30.1  (20.0 to 40.3) | -23.1  (-87.2 to 40.9) | -43.4  (-163.6 to 76.8) | 28.0  (13.5 to 42.5) | | -2.1  (-19.8 to 15.5) | -7.1  (-65.7 to 51.5) |
| **Other** | 10.2  (2.9 to 17.6) | -2.1  (-10.8 to 6.7) | -16.7  (-87.9 to 54.5) | 7.3  (4.2 to 10.3) | | -3.0  (-10.9 to 5.0) | -29.0  (-106.8 to 48.9) |
| **Patient Location^h^** |  |  |  |  | |  |  |
| **"Central" counties of metro areas of >=1 million population** | 11.1  (8.6 to 13.6) | 0.4  (-3.0 to 3.8) | 3.6  (-28.1 to 35.4) | 11.2  (7.6 to 14.8) | | 0.1  (-4.3 to 4.5) | 0.6  (-39.0 to 40.3) |
| **"Fringe" counties of metro areas of >=1 million population** | 13.2  (11.1 to 15.2) | -5.6  (-10.2 to -1.0) | -29.9  (-54.5 to -5.2) | 9.4  (8.1 to 10.6) | | -3.8  (-6.2 to -1.4) | -28.9  (-47.0 to -10.8) |
| **Counties in metro areas of 250,000-999,999 population** | 10.9  (9.2 to 12.6) | -5.2  (-10.8 to 0.5) | -32.2  (-67.3 to 2.9) | 10.6  (8.6 to 12.5) | | -0.3  (-2.9 to 2.3) | -3.1  (-26.9 to 20.7) |
| **Counties in metro areas of 50,000-249,999 population** | 8.5  (6.7 to 10.3) | 0.1  (-3.0 to 3.3) | 1.5  (-36.2 to 39.3) | 9.6  (7.5 to 11.8) | | 1.1  (-1.7 to 3.9) | 13.0  (-19.8 to 45.8) |
| **Micropolitan counties** | 7.8  (6.1 to 9.4) | -0.8  (-3.5 to 1.9) | -9.3  (-40.3 to 21.6) | 7.2  (6.1 to 8.2) | | -0.6  (-2.6 to 1.3) | -7.8  (-32.8 to 17.1) |
| **Not metropolitan or micropolitan counties** | 3.9  (3.2 to 4.6) | -0.1  (-1.2 to 1.1) | -2.0  (-31.5 to 27.4) | 5.2  (4.4 to 6.0) | | 1.3  (0.3 to 2.3) | 33.9  (7.5 to 60.4) |

^a^ Cases of heroin-related adverse event or poisoning were identified following the definition proposed by the Healthcare Cost and Utilization Project (ICD-10-CM code used: found in Table S 1).

^b^ Reflects the overall or total difference between 2016 and 2019: absolute difference (2019 – 2016) and percentage change [(2019 – 2016)/2016 × 100].

^c^ Reflects the overall or total difference during the COVID-19 pandemic in 2020 and 2021: absolute difference (2021 – 2019) and percentage change [(2021 – 2019)/2019 × 100].

^d^ NA, Not Available. Due to the NEDS survey design, the Race/ethnicity classification was not available until 2019 and therefore could not present estimates.

^e^ Race/ethnicity variable was obtained from the Agency for Healthcare Research and Quality, Healthcare Cost and Utilization Project; nationally weighted from all records in states with reliable race/ethnicity reporting, which is collected by self-report on admission to the hospital using fixed categories.

^f^ Median household income of residents in the patient’s zip code was updated annually; 2019 range for quartile 1 was less than $48,000; quartile 2, $48,000 to $60,999; quartile 3, $61,000 to $81,999; and quartile 4, at least $82,000; 2021 range for quartile 1 was less than $52,000; quartile 2, $52,000 to $65,999; quartile 3, $66,000 to $87,999; and quartile 4, at least $88,000.

^g^ “Medicare” includes both fee-for-service and managed care Medicare patients. “Medicaid” includes both fee-for-service and managed care Medicaid patients. “Private insurance” includes Blue Cross, commercial carriers, and private HMOs and PPOs. “Other” includes Worker's Compensation, CHAMPUS (Civilian Health and Medical Program of the Uniformed Services), CHAMPVA (Civilian Health and Medical Program of the Department of Veteran's Affairs), Title V, and other government programs.

^h^ “Patient location” is a six-category urban-rural classification scheme for U.S. counties developed by the National Center for Health Statistics (NCHS) especially for use in health care research. The classification emphasizes urban distinctions and is unique in differentiating between central and fringe counties of large metropolitan areas. Smaller metropolitan counties are subdivided by population. Non-metropolitan counties are divided simply into micropolitan and non-core categories.

**Supplementary Table S23. Opium-Related Adverse Event or Poisoning^a^ (2016 to 2019 and 2020 to 2021) in National Inpatient Sample Overall and for Subtypes by Demographic Characteristics**

| **Characteristics** | **Between 2016 and 2019** | | | | **During the COVID-19 pandemic in 2020 and 2021** | | |
| --- | --- | --- | --- | --- | --- | --- | --- |
|  | **2019 Rate,**  **per 10,000** | **Absolute difference**  **from 2016-2019^b^** | **Change**  **from 2016-2019, %** | **2021 Rate,**  **per 10,000** | | **Absolute difference**  **from 2019-2021^c^** | **Change**  **from 2019-2021, %** |
| **Overall** | 0.5  (0.4 to 0.6) | -0.6  (-0.7 to -0.5) | -53.6  (-66.2 to -41.0) | 0.4  (0.3 to 0.5) | | -0.1  (-0.2 to -0.02) | -25.0  (-46.3 to -3.7) |
| **AGE** |  |  |  |  | |  |  |
| **18-44** | 0.5  (0.4 to 0.6) | -0.6  (-0.8 to -0.4) | -53.8  (-73.8 to -33.8) | 0.4  (0.3 to 0.5) | | -0.1  (-0.3 to 0.07) | -20.4  (-54.4 to 13.5) |
| **45-64** | 0.7  (0.6 to 0.9) | -0.9  (-1.2 to -0.7) | -56.6  (-73.0 to -40.2) | 0.5  (0.4 to 0.7) | | -0.2  (-0.4 to 0) | -26.4  (-53.3 to 0.6) |
| **65-84** | 0.4  (0.3 to 0.5) | -0.5  (-0.6 to -0.3) | -50.5  (-70.9 to -30.2) | 0.3  (0.2 to 0.4) | | -0.1  (-0.3 to 0) | -28.9  (-56.8 to -1.0) |
| **≥85** | 0.3  (0.1 to 0.4) | -0.07  (-0.3 to 0.1) | -20.0  (-79.5 to 39.5) | 0.2  (0.1 to 0.3) | | -0.06  (-0.3 to 0.1) | -21.4  (-90.7 to 47.9) |
| **Sex** |  |  |  |  | |  |  |
| **Male** | 0.6  (0.5 to 0.7) | -0.5  (-0.7 to -0.3) | -47.7  (-65.4 to -30.1) | 0.4  (0.3 to 0.5) | | -0.2  (-0.3 to -0.06) | -34.5  (-58.9 to -10.1) |
| **Female** | 0.5  (0.4 to 0.6) | -0.6  (-0.8 to -0.5) | -57.1  (-71.3 to -43.0) | 0.4  (0.3 to 0.5) | | -0.08  (-0.2 to 0.05) | -16.7  (-42.8 to 9.5) |
| **Race/ethnicity^d^** |  |  |  |  | |  |  |
| **Non-Hispanic White** | 0.5  (0.5 to 0.6) | -0.7  (-0.8 to -0.5) | -55.7  (-68.7 to -42.8) | 0.4  (0.3 to 0.4) | | -0.2  (-0.3 to -0.06) | -31.5  (-52.0 to -11.0) |
| **Non-Hispanic Black** | 0.5  (0.3 to 0.6) | -0.5  (-0.8 to -0.2) | -52.0  (-79.2 to -24.8) | 0.5  (0.3 to 0.6) | | -0.02  (-0.2 to 0.2) | -4.2  (-50.7 to 42.4) |
| **Hispanic** | 0.5  (0.2 to 0.8) | -0.2  (-0.6 to 0.2) | -31.1  (-83.7 to 21.5) | 0.5  (0.2 to 0.7) | | -0.06  (-0.5 to 0.3) | -11.8  (-90.6 to 67.1) |
| **Non-Hispanic Asian or Pacific Islander** | NR^e^ | -0.3  (-0.8 to 0.2) | -58.6  (-145.8 to 28.6) | NR^e^ | | 0.01  (-0.4 to 0.4) | 4.2  (-157.9 to 166.3) |
| **Native American** | NR^e^ | -1.5  (-3.1 to 0.1) | -74.5  (-155.9 to 6.9) | NR^e^ | | -0.3  (-1.1 to 0.6) | -50.0  (-215.4 to 115.4) |
| **Other** | NR^e^ | -0.5  (-1.1 to 0.1) | -50.5  (-115.8 to 14.7) | NR^e^ | | -0.3  (-0.7 to 0.1) | -59.6  (-147.6 to 28.4) |
| **Zip code income quartile^f^** |  |  |  |  | |  |  |
| **1 (lowest)** | 0.5  (0.4 to 0.7) | -0.6  (-0.8 to -0.3) | -51.4  (-72.3 to -30.5) | 0.5  (0.3 to 0.6) | | -0.07  (-0.3 to 0.1) | -13.5  (-48.2 to 21.3) |
| **2** | 0.6  (0.4 to 0.7) | -0.5  (-0.8 to -0.2) | -45.8  (-70.7 to -20.9) | 0.3  (0.2 to 0.5) | | -0.2  (-0.4 to -0.04) | -41.4  (-75.2 to -7.6) |
| **3** | 0.4  (0.3 to 0.5) | -0.9  (-1.1 to -0.6) | -68.0  (-86.3 to -49.7) | 0.4  (0.3 to 0.5) | | -0.02  (-0.2 to 0.2) | -5.0  (-46.6 to 36.6) |
| **4 (highest)** | 0.6  (0.4 to 0.7) | -0.5  (-0.7 to -0.2) | -44.8  (-69.1 to -20.4) | 0.4  (0.2 to 0.5) | | -0.2  (-0.4 to -0.05) | -39.7  (-70.8 to -8.5) |
| **Primary expected payer^g^** |  |  |  |  | |  |  |
| **Medicare** | 0.5  (0.4 to 0.6) | -0.7  (-0.9 to -0.5) | -58.1  (-73.1 to -43.1) | 0.3  (0.3 to 0.4) | | -0.2  (-0.3 to -0.04) | -30.6  (-53.2 to -8.0) |
| **Medicaid** | 0.7  (0.5 to 0.9) | -0.7  (-1.0 to -0.3) | -48.6  (-71.8 to -25.3) | 0.5  (0.3 to 0.7) | | -0.2  (-0.5 to 0.06) | -28.2  (-65.3 to 9.0) |
| **Private insurance** | 0.4  (0.3 to 0.5) | -0.4  (-0.5 to -0.2) | -46.7  (-72.8 to -20.5) | 0.3  (0.2 to 0.4) | | -0.07  (-0.2 to 0.1) | -17.5  (-59.1 to 24.1) |
| **Self-pay** | 0.7  (0.4 to 1.1) | -0.8  (-1.4 to -0.2) | -52.3  (-90.5 to -14.0) | 0.7  (0.4 to 1.0) | | -0.05  (-0.5 to 0.4) | -6.8  (-70.4 to 56.9) |
| **No charge** | NR^e^ | -0.4  (-2.5 to 1.6) | -30.4  (-178.6 to 117.8) | NR^e^ | | NR^e^ | NR^e^ |
| **Other** | NR^e^ | -1.1  (-1.8 to -0.4) | -71.5  (-114.8 to -28.2) | NR^e^ | | 0.09  (-0.4 to 0.6) | 20.0  (-81.7 to 121.7) |
| **Patient Location^h^** |  |  |  |  | |  |  |
| **"Central" counties of metro areas of >=1 million population** | 0.7  (0.5 to 0.9) | -0.5  (-0.7 to -0.2) | -39.1  (-61.0 to -17.3) | 0.5  (0.3 to 0.6) | | -0.2  (-0.4 to 0) | -31.4  (-63.1 to 0.3) |
| **"Fringe" counties of metro areas of >=1 million population** | 0.6  (0.4 to 0.7) | -0.4  (-0.7 to -0.2) | -43.1  (-65.0 to -21.2) | 0.4  (0.2 to 0.5) | | -0.2  (-0.4 to -0.01) | -34.5  (-67.9 to -1.0) |
| **Counties in metro areas of 250,000-999,999 population** | 0.5  (0.3 to 0.6) | -0.8  (-1.0 to -0.5) | -62.8  (-84.5 to -41.1) | 0.2  (0.1 to 0.3) | | -0.2  (-0.4 to -0.06) | -46.7  (-80.7 to -12.7) |
| **Counties in metro areas of 50,000-249,999 population** | 0.3  (0.2 to 0.4) | -0.9  (-1.2 to -0.6) | -75.0  (-103.5 to -46.5) | 0.4  (0.2 to 0.6) | | 0.1  (-0.2 to 0.4) | 36.7  (-48.5 to 121.9) |
| **Micropolitan counties** | 0.4  (0.2 to 0.5) | -0.8  (-1.3 to -0.3) | -69.0  (-108.5 to -29.4) | 0.5  (0.3 to 0.6) | | 0.09  (-0.2 to 0.3) | 25.0  (-40.6 to 90.6) |
| **Not metropolitan or micropolitan counties** | NR^e^ | -0.6  (-0.9 to -0.3) | -70.7  (-109.3 to -32.2) | 0.3  (0.1 to 0.5) | | 0.08  (-0.2 to 0.3) | 33.3  (-65.0 to 131.7) |

^a^ Cases of opium-related adverse event or poisoning were identified following the definition proposed by the Healthcare Cost and Utilization Project (ICD-10-CM code used: found in Table S 1).

^b^ Reflects the overall or total difference between 2016 and 2019: absolute difference (2019 – 2016) and percentage change [(2019 – 2016)/2016 × 100].

^c^ Reflects the overall or total difference during the COVID-19 pandemic in 2020 and 2021: absolute difference (2021 – 2019) and percentage change [(2021 – 2019)/2019 × 100].

^d^ Race/ethnicity variable was obtained from the Agency for Healthcare Research and Quality, Healthcare Cost and Utilization Project; nationally weighted from all records in states with reliable race/ethnicity reporting, which is collected by self-report on admission to the hospital using fixed categories.

^e^ NR, Not Reportable. Suppressed to protect confidentiality, ≤10 cases.

^f^ Median household income of residents in the patient’s zip code was updated annually; 2019 range for quartile 1 was less than $48,000; quartile 2, $48,000 to $60,999; quartile 3, $61,000 to $81,999; and quartile 4, at least $82,000; 2021 range for quartile 1 was less than $52,000; quartile 2, $52,000 to $65,999; quartile 3, $66,000 to $87,999; and quartile 4, at least $88,000.

^g^ “Medicare” includes both fee-for-service and managed care Medicare patients. “Medicaid” includes both fee-for-service and managed care Medicaid patients. “Private insurance” includes Blue Cross, commercial carriers, and private HMOs and PPOs. “Other” includes Worker's Compensation, CHAMPUS (Civilian Health and Medical Program of the Uniformed Services), CHAMPVA (Civilian Health and Medical Program of the Department of Veteran's Affairs), Title V, and other government programs.

^h^ “Patient location” is a six-category urban-rural classification scheme for U.S. counties developed by the National Center for Health Statistics (NCHS) especially for use in health care research. The classification emphasizes urban distinctions and is unique in differentiating between central and fringe counties of large metropolitan areas. Smaller metropolitan counties are subdivided by population. Non-metropolitan counties are divided simply into micropolitan and non-core categories.

**Supplementary Table S24. Opium-Related Adverse Event or Poisoning^a^ (2016 to 2019 and 2020 to 2021) in Nationwide Emergency Department Sample and for Subtypes by Demographic Characteristics**

| **Characteristics** | **Between 2016 and 2019** | | | | **During the COVID-19 pandemic in 2020 and 2021** | | |
| --- | --- | --- | --- | --- | --- | --- | --- |
|  | **2019 Rate,**  **per 10,000** | **Absolute difference**  **from 2016-2019^b^** | **Change**  **from 2016-2019, %** | **2021 Rate,**  **per 10,000** | | **Absolute difference**  **from 2019-2021^c^** | **Change**  **from 2019-2021, %** |
| **Overall** | 0.3  (0.2 to 0.3) | -0.2  (-0.3 to -0.2) | -48.0  (-62.1 to -33.9) | 0.2  (0.2 to 0.3) | | -0.04  (-0.1 to 0.03) | -15.4  (-42.6 to 11.8) |
| **AGE** |  |  |  |  | |  |  |
| **18-44** | 0.2  (0.2 to 0.3) | -0.2  (-0.3 to -0.1) | -46.5  (-65.9 to -27.2) | 0.3  (0.2 to 0.3) | | 0.02  (-0.06 to 0.1) | 8.7  (-27.5 to 44.9) |
| **45-64** | 0.3  (0.3 to 0.4) | -0.4  (-0.5 to -0.2) | -51.5  (-69.9 to -33.0) | 0.2  (0.1 to 0.3) | | -0.1  (-0.2 to 0.03) | -30.3  (-68.3 to 7.7) |
| **65-84** | 0.2  (0.2 to 0.3) | -0.2  (-0.3 to -0.1) | -46.7  (-68.4 to -24.9) | 0.2  (0.1 to 0.2) | | -0.06  (-0.1 to 0.02) | -25.0  (-59.7 to 9.7) |
| **≥85** | 0.1  (0.1 to 0.2) | -0.2  (-0.3 to -0.03) | -53.1  (-97.3 to -9.0) | 0.1  (0.1 to 0.2) | | -0.04  (-0.1 to 0.06) | -26.7  (-92.0 to 38.7) |
| **Sex** |  |  |  |  | |  |  |
| **Male** | 0.3  (0.3 to 0.4) | -0.2  (-0.3 to -0.1) | -42.9  (-60.4 to -25.4) | 0.3  (0.2 to 0.3) | | -0.04  (-0.1 to 0.06) | -12.5  (-43.1 to 18.1) |
| **Female** | 0.2  (0.2 to 0.3) | -0.2  (-0.3 to -0.2) | -52.2  (-70.3 to -34.1) | 0.2  (0.1 to 0.2) | | -0.04  (-0.1 to 0.04) | -18.2  (-56.0 to 19.6) |
| **Race/ethnicity^d^** |  |  |  |  | |  |  |
| **Non-Hispanic White** | 0.3  (0.2 to 0.3) | NA^e^ | NA^e^ | 0.2  (0.2 to 0.3) | | -0.06  (-0.1 to 0.01) | -20.7  (-45.1 to 3.7) |
| **Non-Hispanic Black** | 0.2  (0.1 to 0.3) | NA^e^ | NA^e^ | 0.2  (0.1 to 0.3) | | -0.02  (-0.1 to 0.09) | -9.5  (-62.3 to 43.3) |
| **Hispanic** | 0.3  (0.1 to 0.4) | NA^e^ | NA^e^ | 0.2  (0.1 to 0.4) | | -0.02  (-0.2 to 0.2) | -7.4  (-89.5 to 74.7) |
| **Non-Hispanic Asian or Pacific Islander** | NR^f^ | NA^e^ | NA^e^ | NR^f^ | | 0.06  (-0.07 to 0.2) | 75.0  (-81.9 to 231.9) |
| **Native American** | NR^f^ | NA^e^ | NA^e^ | NR^f^ | | 0.07  (-0.2 to 0.4) | 50.0  (-171.4 to 271.4) |
| **Other** | 0.1  (0.03 to 0.2) | NA^e^ | NA^e^ | 0.3  (0.2 to 0.4) | | 0.2  (0.01 to 0.3) | 141.7  (10.0 to 273.4) |
| **Zip code income quartile^g^** |  |  |  |  | |  |  |
| **1 (lowest)** | 0.3  (0.2 to 0.3) | -0.2  (-0.3 to -0.08) | -44.7  (-71.4 to -18.0) | 0.2  (0.2 to 0.3) | | -0.03  (-0.1 to 0.07) | -11.5  (-49.2 to 26.2) |
| **2** | 0.2  (0.2 to 0.3) | -0.2  (-0.3 to -0.2) | -50.0  (-68.1 to -31.9) | 0.2  (0.1 to 0.3) | | -0.02  (-0.1 to 0.08) | -8.7  (-51.3 to 33.9) |
| **3** | 0.2  (0.2 to 0.3) | -0.4  (-0.5 to -0.3) | -61.4  (-78.6 to -44.2) | 0.2  (0.2 to 0.3) | | 0  (-0.08 to 0.08) | 0  (-37.8 to 37.8) |
| **4 (highest)** | 0.3  (0.2 to 0.4) | -0.2  (-0.4 to -0.1) | -44.4  (-67.7 to -21.2) | 0.2  (0.1 to 0.3) | | -0.09  (-0.2 to 0.02) | -30.0  (-67.0 to 7.0) |
| **Primary expected payer^h^** |  |  |  |  | |  |  |
| **Medicare** | 0.3  (0.2 to 0.3) | -0.4  (-0.5 to -0.3) | -56.5  (-72.3 to -40.7) | 0.2  (0.2 to 0.2) | | -0.07  (-0.1 to 0) | -25.9  (-52.1 to 0.3) |
| **Medicaid** | 0.3  (0.2 to 0.4) | -0.3  (-0.4 to -0.2) | -48.3  (-69.3 to -27.4) | 0.3  (0.2 to 0.4) | | -0.02  (-0.1 to 0.09) | -6.5  (-42.2 to 29.3) |
| **Private insurance** | 0.2  (0.1 to 0.3) | -0.2  (-0.2 to -0.07) | -42.9  (-66.6 to -19.1) | 0.1  (0.1 to 0.2) | | -0.05  (-0.2 to 0.05) | -25.0  (-74.0 to 24.0) |
| **Self-pay** | 0.3  (0.2 to 0.4) | -0.1  (-0.3 to 0.01) | -30.2  (-62.5 to 2.0) | 0.4  (0.2 to 0.5) | | 0.06  (-0.1 to 0.3) | 20.0  (-47.3 to 87.3) |
| **No charge** | NR^f^ | -0.5  (-1.2 to 0.3) | -78.7  (-197.9 to 40.5) | NR^f^ | | 0.06  (-0.3 to 0.4) | 46.2  (-192.2 to 284.5) |
| **Other** | 0.2  (0.08 to 0.3) | -0.2  (-0.3 to 0) | -50.0  (-99.6 to -0.4) | 0.2  (0.1 to 0.2) | | -0.01  (-0.1 to 0.1) | -5.9  (-79.7 to 67.9) |
| **Patient Location^i^** |  |  |  |  | |  |  |
| **"Central" counties of metro areas of >=1 million population** | 0.3  (0.2 to 0.4) | -0.1  (-0.3 to -0.01) | -31.1  (-59.0 to -3.2) | 0.3  (0.2 to 0.4) | | -0.04  (-0.2 to 0.1) | -12.9  (-57.6 to 31.8) |
| **"Fringe" counties of metro areas of >=1 million population** | 0.3  (0.2 to 0.4) | -0.2  (-0.4 to -0.1) | -44.4  (-67.7 to -21.2) | 0.3  (0.1 to 0.4) | | -0.02  (-0.2 to 0.2) | -6.7  (-65.1 to 51.8) |
| **Counties in metro areas of 250,000-999,999 population** | 0.2  (0.2 to 0.3) | -0.4  (-0.5 to -0.2) | -63.8  (-86.5 to -41.1) | 0.2  (0.1 to 0.2) | | -0.06  (-0.1 to 0.01) | -28.6  (-62.2 to 5.1) |
| **Counties in metro areas of 50,000-249,999 population** | 0.2  (0.1 to 0.2) | -0.2  (-0.3 to -0.1) | -56.4  (-85.7 to -27.1) | 0.3  (0.2 to 0.4) | | 0.09  (-0.02 to 0.2) | 52.9  (-14.3 to 120.2) |
| **Micropolitan counties** | 0.2  (0.2 to 0.3) | -0.4  (-0.6 to -0.2) | -60.0  (-95.2 to -24.8) | 0.2  (0.1 to 0.2) | | -0.09  (-0.2 to 0.01) | -37.5  (-78.3 to 3.3) |
| **Not metropolitan or micropolitan counties** | 0.2  (0.1 to 0.2) | -0.2  (-0.4 to -0.07) | -57.5  (-97.0 to -18.0) | 0.09  (0.04 to 0.1) | | -0.08  (-0.2 to 0.01) | -47.1  (-98.6 to 4.5) |

^a^ Cases of opium-related adverse event or poisoning were identified following the definition proposed by the Healthcare Cost and Utilization Project (ICD-10-CM code used: found in Table S 1).

^b^ Reflects the overall or total difference between 2016 and 2019: absolute difference (2019 – 2016) and percentage change [(2019 – 2016)/2016 × 100].

^c^ Reflects the overall or total difference during the COVID-19 pandemic in 2020 and 2021: absolute difference (2021 – 2019) and percentage change [(2021 – 2019)/2019 × 100].

^d^ Race/ethnicity variable was obtained from the Agency for Healthcare Research and Quality, Healthcare Cost and Utilization Project; nationally weighted from all records in states with reliable race/ethnicity reporting, which is collected by self-report on admission to the hospital using fixed categories.

^e^ NA, Not Available. Due to the NEDS survey design, the Race/ethnicity classification was not available until 2019 and therefore could not present estimates.

^f^ NR, Not Reportable. Suppressed to protect confidentiality, ≤10 cases.

^g^ Median household income of residents in the patient’s zip code was updated annually; 2019 range for quartile 1 was less than $48,000; quartile 2, $48,000 to $60,999; quartile 3, $61,000 to $81,999; and quartile 4, at least $82,000; 2021 range for quartile 1 was less than $52,000; quartile 2, $52,000 to $65,999; quartile 3, $66,000 to $87,999; and quartile 4, at least $88,000.

^h^ “Medicare” includes both fee-for-service and managed care Medicare patients. “Medicaid” includes both fee-for-service and managed care Medicaid patients. “Private insurance” includes Blue Cross, commercial carriers, and private HMOs and PPOs. “Other” includes Worker's Compensation, CHAMPUS (Civilian Health and Medical Program of the Uniformed Services), CHAMPVA (Civilian Health and Medical Program of the Department of Veteran's Affairs), Title V, and other government programs.

^i^ “Patient location” is a six-category urban-rural classification scheme for U.S. counties developed by the National Center for Health Statistics (NCHS) especially for use in health care research. The classification emphasizes urban distinctions and is unique in differentiating between central and fringe counties of large metropolitan areas. Smaller metropolitan counties are subdivided by population. Non-metropolitan counties are divided simply into micropolitan and non-core categories.

**Supplementary Table S25. Methadone-Related Adverse Event or Poisoning^a^ (2016 to 2019 and 2020 to 2021) in National Inpatient Sample Overall and for Subtypes by Demographic Characteristics**

| **Characteristics** | **Between 2016 and 2019** | | | | **During the COVID-19 pandemic in 2020 and 2021** | | |
| --- | --- | --- | --- | --- | --- | --- | --- |
|  | **2019 Rate,**  **per 10,000** | **Absolute difference**  **from 2016-2019^b^** | **Change**  **from 2016-2019, %** | **2021 Rate,**  **per 10,000** | | **Absolute difference**  **from 2019-2021^c^** | **Change**  **from 2019-2021, %** |
| **Overall** | 2.0  (1.9 to 2.2) | -0.7  (-1.0 to -0.5) | -26.3  (-34.7 to -17.8) | 1.8  (1.6 to 1.9) | | -0.3  (-0.5 to -0.09) | -14.6  (-24.8 to -4.5) |
| **AGE** |  |  |  |  | |  |  |
| **18-44** | 2.2  (2.0 to 2.5) | -1.3  (-1.7 to -0.9) | -36.6  (-48.1 to -25.2) | 1.7  (1.5 to 2.0) | | -0.5  (-0.8 to -0.2) | -22.4  (-37.4 to -7.5) |
| **45-64** | 3.6  (3.2 to 3.9) | -1.1  (-1.6 to -0.6) | -23.4  (-34.7 to -12.1) | 2.9  (2.6 to 3.2) | | -0.7  (-1.1 to -0.2) | -18.5  (-31.7 to -5.3) |
| **65-84** | 1.2  (1.0 to 1.3) | 0  (-0.2 to 0.2) | -0.8  (-19.6 to 17.9) | 1.3  (1.1 to 1.4) | | 0.08  (-0.1 to 0.3) | 6.8  (-12.1 to 25.8) |
| **≥85** | 0.3  (0.1 to 0.4) | 0  (-0.2 to 0.2) | 12.0  (-65.6 to 89.6) | 0.2  (0.1 to 0.3) | | -0.08  (-0.3 to 0.1) | -28.6  (-93.1 to 36.0) |
| **Sex** |  |  |  |  | |  |  |
| **Male** | 2.4  (2.2 to 2.6) | -0.9  (-1.2 to -0.5) | -27.1  (-37.8 to -16.5) | 2.1  (1.9 to 2.3) | | -0.3  (-0.6 to -0.01) | -13.0  (-25.7 to -0.2) |
| **Female** | 1.8  (1.6 to 2.0) | -0.6  (-0.9 to -0.4) | -26.0  (-35.8 to -16.3) | 1.5  (1.3 to 1.7) | | -0.3  (-0.5 to -0.07) | -16.2  (-28.6 to -3.8) |
| **Race/ethnicity^e^** |  |  |  |  | |  |  |
| **Non-Hispanic White** | 2.2  (2.1 to 2.4) | -0.9  (-1.1 to -0.6) | -27.6  (-36.2 to -19.0) | 1.9  (1.8 to 2.1) | | -0.3  (-0.5 to -0.06) | -13.5  (-24.0 to -2.9) |
| **Non-Hispanic Black** | 1.9  (1.5 to 2.3) | -0.5  (-1.1 to 0.1) | -21.3  (-45.1 to 2.4) | 1.7  (1.3 to 2.0) | | -0.2  (-0.8 to 0.3) | -11.7  (-39.8 to 16.4) |
| **Hispanic** | 1.8  (1.4 to 2.2) | -0.4  (-0.9 to 0.2) | -16.8  (-44.1 to 10.4) | 1.3  (1.0 to 1.6) | | -0.5  (-1.0 to -0.04) | -29.8  (-57.3 to -2.3) |
| **Non-Hispanic Asian or Pacific Islander** | 0.5  (0.1 to 0.8) | -0.1  (-0.6 to 0.4) | -17.2  (-108.5 to 74.1) | 0.3  (0 to 0.6) | | -0.2  (-0.6 to 0.3) | -35.4  (-125.3 to 54.5) |
| **Native American** | 3.0  (1.2 to 4.7) | -0.4  (-3.0 to 2.2) | -11.3  (-89.0 to 66.3) | 3.5  (1.6 to 5.5) | | 0.6  (-2.1 to 3.2) | 18.8  (-70.2 to 107.8) |
| **Other** | 1.5  (0.8 to 2.2) | -0.7  (-1.7 to 0.3) | -31.1  (-76.7 to 14.6) | 1.5  (1.0 to 2.1) | | 0.04  (-0.9 to 0.9) | 2.7  (-56.2 to 61.5) |
| **Zip code income quartile^f^** |  |  |  |  | |  |  |
| **1 (lowest)** | 2.4  (2.1 to 2.7) | -0.9  (-1.4 to -0.5) | -28.1  (-41.1 to -15.1) | 2.1  (1.8 to 2.4) | | -0.3  (-0.7 to 0.1) | -11.8  (-28.7 to 5.1) |
| **2** | 2.0  (1.8 to 2.3) | -0.7  (-1.1 to -0.3) | -26.1  (-40.4 to -11.8) | 1.6  (1.4 to 1.8) | | -0.4  (-0.7 to -0.06) | -19.4  (-36.0 to -2.8) |
| **3** | 1.8  (1.6 to 2.1) | -0.7  (-1.1 to -0.4) | -28.5  (-43.2 to -13.8) | 1.6  (1.4 to 1.8) | | -0.2  (-0.6 to 0.1) | -12.0  (-30.2 to 6.2) |
| **4 (highest)** | 1.7  (1.5 to 2.0) | -0.3  (-0.7 to 0) | -16.6  (-34.9 to 1.7) | 1.4  (1.2 to 1.6) | | -0.3  (-0.7 to 0.03) | -18.7  (-39.0 to 1.6) |
| **Primary expected payer^g^** |  |  |  |  | |  |  |
| **Medicare** | 1.6  (1.5 to 1.8) | -0.4  (-0.6 to -0.2) | -19.3  (-310 to -7.6) | 1.4  (1.2 to 1.5) | | -0.3  (-0.5 to -0.04) | -16.0  (-29.6 to -2.4) |
| **Medicaid** | 4.6  (4.1 to 5.1) | -1.4  (-2.1 to -0.6) | -23.0  (-35.9 to -10.2) | 3.8  (3.4 to 4.2) | | -0.8  (-1.5 to -0.1) | -17.3  (-32.1 to -2.4) |
| **Private insurance** | 1.0  (0.8 to 1.2) | -0.5  (-0.8 to -0.3) | -34.6  (-51.9 to -17.4) | 0.9  (0.7 to 1.0) | | -0.1  (-0.4 to 0.1) | -13.0  (-36.6 to 10.6) |
| **Self-pay** | 3.0  (2.4 to 3.7) | -2.9  (-4.1 to -1.6) | -48.5  (-69.4 to -27.6) | 2.2  (1.6 to 2.8) | | -0.8  (-1.8 to 0.1) | -27.4  (-58.1 to 3.3) |
| **No charge** | 4.3  (0.8 to 7.8) | 0.2  (-4.4 to 4.7) | 3.9  (-106.7 to 114.4) | 2.8  (0.5 to 5.0) | | -1.5  (-5.7 to 2.7) | -35.8  (-133.3 to 61.7) |
| **Other** | 1.1  (0.6 to 1.6) | -1.2  (-2.2 to -0.2) | -50.9  (-92.9 to -8.8) | 1.7  (1.1 to 2.3) | | 0.6  (-0.2 to 1.4) | 50.9  (-16.3 to 118.0) |
| **Patient Location^h^** |  |  |  |  | |  |  |
| **"Central" counties of metro areas of >=1 million population** | 2.5  (2.2 to 2.9) | -0.8  (-1.3 to -0.3) | -23.7  (-38.6 to -8.7) | 2.2  (1.9 to 2.5) | | -0.4  (-0.8 to 0.1) | -13.7  (-32.2 to 4.8) |
| **"Fringe" counties of metro areas of >=1 million population** | 1.8  (1.6 to 2.1) | -0.5  (-0.8 to -0.1) | -20.5  (-37.0 to -4.1) | 1.5  (1.3 to 1.8) | | -0.3  (-0.6 to 0.04) | -15.9  (-34.2 to 2.3) |
| **Counties in metro areas of 250,000-999,999 population** | 2.1  (1.7 to 2.4) | -0.7  (-1.1 to -0.2) | -24.2  (-42.0 to -6.3) | 1.9  (1.6 to 2.2) | | -0.2  (-0.6 to 0.3) | -8.2  (-28.3 to 11.9) |
| **Counties in metro areas of 50,000-249,999 population** | 2.1  (1.6 to 2.5) | -0.6  (-1.2 to 0) | -22.6  (-45.7 to 0.4) | 1.5  (1.2 to 1.9) | | -0.5  (-1.1 to 0.02) | -25.4  (-51.8 to 1.1) |
| **Micropolitan counties** | 1.2  (0.9 to 1.5) | -1.0  (-1.5 to -0.5) | -45.2  (-67.6 to -22.8) | 1.0  (0.7 to 1.3) | | -0.2  (-0.6 to 0.2) | -15.0  (-48.5 to 18.5) |
| **Not metropolitan or micropolitan counties** | 1.5  (1.1 to 1.9) | -1.1  (-1.8 to -0.5) | -42.7  (-67.8 to -17.7) | 0.9  (0.6 to 1.2) | | -0.6  (-1.1 to -0.05) | -38.0  (-72.5 to -3.5) |

^a^ Cases of methadone-related adverse event or poisoning were identified following the definition proposed by the Healthcare Cost and Utilization Project (ICD-10-CM code used: found in Table S 1).

^b^ Reflects the overall or total difference between 2016 and 2019: absolute difference (2019 – 2016) and percentage change [(2019 – 2016)/2016 × 100].

^c^ Reflects the overall or total difference during the COVID-19 pandemic in 2020 and 2021: absolute difference (2021 – 2019) and percentage change [(2021 – 2019)/2019 × 100].

^d^ NR, Not Reportable. Suppressed to protect confidentiality, ≤10 cases.

^e^ Race/ethnicity variable was obtained from the Agency for Healthcare Research and Quality, Healthcare Cost and Utilization Project; nationally weighted from all records in states with reliable race/ethnicity reporting, which is collected by self-report on admission to the hospital using fixed categories.

^f^ Median household income of residents in the patient’s zip code was updated annually; 2019 range for quartile 1 was less than $48,000; quartile 2, $48,000 to $60,999; quartile 3, $61,000 to $81,999; and quartile 4, at least $82,000; 2021 range for quartile 1 was less than $52,000; quartile 2, $52,000 to $65,999; quartile 3, $66,000 to $87,999; and quartile 4, at least $88,000.

^g^ “Medicare” includes both fee-for-service and managed care Medicare patients. “Medicaid” includes both fee-for-service and managed care Medicaid patients. “Private insurance” includes Blue Cross, commercial carriers, and private HMOs and PPOs. “Other” includes Worker's Compensation, CHAMPUS (Civilian Health and Medical Program of the Uniformed Services), CHAMPVA (Civilian Health and Medical Program of the Department of Veteran's Affairs), Title V, and other government programs.

^h^ “Patient location” is a six-category urban-rural classification scheme for U.S. counties developed by the National Center for Health Statistics (NCHS) especially for use in health care research. The classification emphasizes urban distinctions and is unique in differentiating between central and fringe counties of large metropolitan areas. Smaller metropolitan counties are subdivided by population. Non-metropolitan counties are divided simply into micropolitan and non-core categories.

**Supplementary Table S26. Methadone****-Related Adverse Event or Poisoninga (2016 to 2019 and 2020 to 2021) in Nationwide Emergency Department Sample Overall and for Subtypes by Demographic Characteristics**

| **Characteristics** | **Between 2016 and 2019** | | | | **During the COVID-19 pandemic in 2020 and 2021** | | |
| --- | --- | --- | --- | --- | --- | --- | --- |
|  | **2019 Rate,**  **per 10,000** | **Absolute difference**  **from 2016-2019^b^** | **Change**  **from 2016-2019, %** | **2021 Rate,**  **per 10,000** | | **Absolute difference**  **from 2019-2021^c^** | **Change**  **from 2019-2021, %** |
| **Overall** | 0.8  (0.7 to 0.9) | -0.2  (-0.3 to -0.08) | -20.8  (-33.2 to -8.4) | 0.7  (0.7 to 0.8) | | -0.08  (-0.2 to 0.05) | -10.0  (-25.7 to 5.7) |
| **AGE** |  |  |  |  | |  |  |
| **18-44** | 0.6  (0.6 to 0.7) | -0.2  (-0.4 to -0.1) | -27.6  (-40.3 to -14.8) | 0.6  (0.6 to 0.7) | | 0.02  (-0.09 to 0.1) | 3.2  (-14.4 to 20.8) |
| **45-64** | 1.3  (1.1 to 1.5) | -0.3  (-0.6 to -0.04) | -19.1  (-35.9 to -2.3) | 1.0  (0.9 to 1.1) | | -0.3  (-0.5 to -0.04) | -21.3  (-39.3 to -3.3) |
| **65-84** | 0.7  (0.6 to 0.8) | -0.03  (-0.2 to 0.1) | -4.2  (-23.8 to 15.3) | 0.7  (0.6 to 0.8) | | -0.02  (-0.2 to 0.1) | -2.9  (-23.3 to 17.4) |
| **≥85** | 0.2  (0.1 to 0.2) | 0  (-0.1 to 0.1) | 0  (-61.3 to 61.3) | 0.1  (0 to 0.2) | | -0.04  (-0.1 to 0.06) | -25.0  (-86.3 to 36.3) |
| **Sex** |  |  |  |  | |  |  |
| **Male** | 1.0  (0.8 to 1.1) | -0.3  (-0.5 to -0.1) | -22.6  (-37.2 to -8.0) | 0.8  (0.7 to 0.9) | | -0.1  (-0.3 to 0.03) | -14.6  (-32.2 to 3.0) |
| **Female** | 0.7  (0.6 to 0.8) | -0.2  (-0.3 to -0.05) | -19.1  (-32.3 to -5.9) | 0.6  (0.6 to 0.7) | | -0.04  (-0.1 to 0.06) | -5.9  (-20.3 to 8.5) |
| **Race/ethnicity^e^** |  |  |  |  | |  |  |
| **Non-Hispanic White** | 0.9  (0.9 to 1.0) | NA^d^ | NA^d^ | 0.9  (0.8 to 1.0) | | -0.03  (-0.2 to 0.1) | -3.2  (-16.5 to 10.2) |
| **Non-Hispanic Black** | 0.6  (0.4 to 0.8) | NA^d^ | NA^d^ | 0.4  (0.3 to 0.5) | | -0.2  (-0.4 to 0.09) | -26.7  (-69.1 to 15.8) |
| **Hispanic** | 0.6  (0.4 to 0.8) | NA^d^ | NA^d^ | 0.4  (0.3 to 0.5) | | -0.2  (-0.4 to 0.02) | -29.0  (-61.6 to 3.5) |
| **Non-Hispanic Asian or Pacific Islander** | 0.2  (0.1 to 0.3) | NA^d^ | NA^d^ | 0.3  (0.1 to 0.4) | | 0.08  (-0.1 to 0.3) | 38.1  (-55.2 to 131.4) |
| **Native American** | 0.8  (0.2 to 1.4) | NA^d^ | NA^d^ | 1.2  (0.5 to 1.9) | | 0.4  (-0.5 to 1.3) | 48.1  (-68.2 to 164.4) |
| **Other** | 0.8  (0.5 to 1.1) | NA^d^ | NA^d^ | 1.0  (0.7 to 1.3) | | 0.2  (-0.3 to 0.6) | 21.0  (-30.3 to 72.3) |
| **Zip code income quartile^f^** |  |  |  |  | |  |  |
| **1 (lowest)** | 0.8  (0.7 to 1.0) | -0.1  (-0.3 to 0.06) | -12.8  (-32.0 to 6.5) | 0.7  (0.6 to 0.8) | | -0.08  (-0.3 to 0.09) | -9.8  (-30.3 to 10.8) |
| **2** | 0.7  (0.6 to 0.8) | -0.3  (-0.5 to -0.2) | -31.7  (-46.5 to -17.0) | 0.6  (0.5 to 0.7) | | -0.1  (-0.3 to -0.01) | -19.7  (-37.4 to -2.0) |
| **3** | 0.8  (0.7 to 0.9) | -0.2  (-0.4 to -0.05) | -22.1  (-39.5 to -4.7) | 0.7  (0.6 to 0.8) | | -0.08  (-0.3 to 0.09) | -9.9  (-30.4 to 10.7) |
| **4 (highest)** | 0.9  (0.7 to 1.0) | -0.1  (-0.3 to 0.08) | -11.3  (-31.3 to 8.7) | 0.8  (0.7 to 1.0) | | -0.04  (-0.2 to 0.2) | -4.7  (-27.2 to 17.9) |
| **Primary expected payer^g^** |  |  |  |  | |  |  |
| **Medicare** | 0.8  (0.7 to 0.9) | -0.2  (-0.4 to -0.09) | -22.2  (-36.4 to -8.1) | 0.7  (0.7 to 0.8) | | -0.1  (-0.2 to 0.03) | -11.9  (-26.9 to 3.0) |
| **Medicaid** | 1.5  (1.2 to 1.7) | -0.2  (-0.5 to 0.2) | -9.8  (-29.2 to 9.7) | 1.3  (1.1 to 1.5) | | -0.2  (-0.5 to 0.1) | -12.2  (-31.3 to 6.9) |
| **Private insurance** | 0.4  (0.3 to 0.4) | -0.1  (-0.2 to 0) | -20.8  (-41.3 to -0.4) | 0.3  (0.2 to 0.4) | | -0.06  (-0.2 to 0.05) | -15.8  (-45.0 to 13.4) |
| **Self-pay** | 0.6  (0.5 to 0.7) | -0.3  (-0.5 to -0.08) | -32.6  (-56.0 to -9.1) | 0.5  (0.4 to 0.6) | | -0.06  (-0.2 to 0.09) | -10.3  (-36.7 to 16.1) |
| **No charge** | 0.9  (0.2 to 1.5) | -0.3  (-1.2 to 0.6) | -25.2  (-104.4 to 54.0) | 1.0  (0.2 to 1.8) | | 0.1  (-0.9 to 1.2) | 15.7  (-98.5 to 129.9) |
| **Other** | 0.4  (0.3 to 0.6) | -0.4  (-0.6 to -0.2) | -46.3  (-74.2 to -18.5) | 0.4  (0.3 to 0.6) | | -0.01  (-0.2 to 0.2) | -2.3  (-43.3 to 38.8) |
| **Patient Location^h^** |  |  |  |  | |  |  |
| **"Central" counties of metro areas of >=1 million population** | 1.0  (0.8 to 1.2) | -0.1  (-0.5 to 0.2) | -12.1  (-38.5 to 14.3) | 0.8  (0.7 to 1.0) | | -0.2  (-0.5 to 0.09) | -18.6  (-46.3 to 9.1) |
| **"Fringe" counties of metro areas of >=1 million population** | 0.8  (0.6 to 0.9) | -0.07  (-0.3 to 0.1) | -8.2  (-29.5 to 13.0) | 0.8  (0.6 to 0.9) | | -0.01  (-0.2 to 0.2) | -1.3  (-29.9 to 27.4) |
| **Counties in metro areas of 250,000-999,999 population** | 0.8  (0.6 to 0.9) | -0.4  (-0.6 to -0.2) | -32.1  (-49.6 to -14.6) | 0.7  (0.6 to 0.8) | | -0.04  (-0.2 to 0.1) | -5.3  (-27.2 to 16.6) |
| **Counties in metro areas of 50,000-249,999 population** | 0.8  (0.6 to 0.9) | -0.2  (-0.4 to 0.02) | -21.1  (-44.6 to 2.5) | 0.7  (0.6 to 0.9) | | -0.02  (-0.2 to 0.2) | -2.7  (-28.5 to 23.2) |
| **Micropolitan counties** | 0.5  (0.4 to 0.6) | -0.2  (-0.4 to -0.07) | -31.6  (-53.5 to -9.7) | 0.4  (0.3 to 0.5) | | -0.09  (-0.2 to 0.06) | -17.3  (-46.8 to 12.1) |
| **Not metropolitan or micropolitan counties** | 0.6  (0.4 to 0.7) | -0.2  (-0.5 to 0) | -29.5  (-58.8 to -0.2) | 0.4  (0.3 to 0.5) | | -0.2  (-0.4 to -0.02) | -34.6  (-64.8 to -4.3) |

^a^ Cases of methadone-related adverse event or poisoning were identified following the definition proposed by the Healthcare Cost and Utilization Project (ICD-10-CM code used: found in Table S 1).

^b^ Reflects the overall or total difference between 2016 and 2019: absolute difference (2019 – 2016) and percentage change [(2019 – 2016)/2016 × 100].

^c^ Reflects the overall or total difference during the COVID-19 pandemic in 2020 and 2021: absolute difference (2021 – 2019) and percentage change [(2021 – 2019)/2019 × 100].

^d^ NA, Not Available. Due to the NEDS survey design, the Race/ethnicity classification was not available until 2019 and therefore could not present estimates.

^e^ Race/ethnicity variable was obtained from the Agency for Healthcare Research and Quality, Healthcare Cost and Utilization Project; nationally weighted from all records in states with reliable race/ethnicity reporting, which is collected by self-report on admission to the hospital using fixed categories.

^f^ Median household income of residents in the patient’s zip code was updated annually; 2019 range for quartile 1 was less than $48,000; quartile 2, $48,000 to $60,999; quartile 3, $61,000 to $81,999; and quartile 4, at least $82,000; 2021 range for quartile 1 was less than $52,000; quartile 2, $52,000 to $65,999; quartile 3, $66,000 to $87,999; and quartile 4, at least $88,000.

^g^ “Medicare” includes both fee-for-service and managed care Medicare patients. “Medicaid” includes both fee-for-service and managed care Medicaid patients. “Private insurance” includes Blue Cross, commercial carriers, and private HMOs and PPOs. “Other” includes Worker's Compensation, CHAMPUS (Civilian Health and Medical Program of the Uniformed Services), CHAMPVA (Civilian Health and Medical Program of the Department of Veteran's Affairs), Title V, and other government programs.

^h^ “Patient location” is a six-category urban-rural classification scheme for U.S. counties developed by the National Center for Health Statistics (NCHS) especially for use in health care research. The classification emphasizes urban distinctions and is unique in differentiating between central and fringe counties of large metropolitan areas. Smaller metropolitan counties are subdivided by population. Non-metropolitan counties are divided simply into micropolitan and non-core categories.

**Supplementary Table S27. Other Opioids-Related Adverse Event or Poisoning^a^ (2016 to 2019 and 2020 to 2021) in National Inpatient Sample Overall and for Subtypes by Demographic Characteristics**

| **Characteristics** | **Between 2016 and 2019** | | | **During the COVID-19 pandemic in 2020 and 2021** | | |
| --- | --- | --- | --- | --- | --- | --- |
|  | **2019 Rate,**  **per 10,000** | **Absolute difference**  **from 2016-2019^b^** | **Change**  **from 2016-2019, %** | **2021 Rate,**  **per 10,000** | **Absolute difference**  **from 2019-2021^c^** | **Change**  **from 2019-2021, %** |
| **Overall** | 24.8  (24.1 to 25.5) | -9.1  (-10.3 to -7.9) | -26.7  (-30.3 to -23.2) | 19.5  (18.9 to 20.1) | -5.3  (-6.3 to -4.4) | -21.4  (-25.3 to -17.5) |
| **AGE** |  |  |  |  |  |  |
| **18-44** | 15.2  (14.4 to 15.9) | -5.8  (-6.9 to -4.6) | -27.6  (-33.2 to -22.0) | 13.2  (12.5 to 13.9) | -1.9  (-3.0 to -0.9) | -12.7  (-19.5 to -6.0) |
| **45-64** | 31.6  (30.4 to 32.8) | -11.1  (-13.0 to -9.2) | -26.0  (-30.5 to -21.5) | 24.1  (23.1 to 25.1) | -7.6  (-9.1 to -6.0) | -23.9  (-28.9 to -18.9) |
| **65-84** | 28.2  (27.2 to 29.2) | -10.6  (-12.3 to -8.9) | -27.3  (-31.7 to -22.9) | 22.3  (21.4 to 23.2) | -5.9  (-7.3 to -4.6) | -21.0  (-25.9 to -16.2) |
| **≥85** | 20.3  (19.0 to 21.5) | -8.8  (-10.8 to -6.7) | -30.2  (-37.3 to -23.0) | 14.8  (13.6 to 15.9) | -5.5  (-7.2 to -3.8) | -27.1  (-35.6 to -18.6) |
| **Sex** |  |  |  |  |  |  |
| **Male** | 23.9  (23.0 to 24.8) | -8.7  (-10.1 to -7.3) | -26.8  (-31.0 to -22.5) | 19.7  (18.9 to 20.4) | -4.3  (-5.4 to -3.1) | -17.8  (-22.6 to -12.9) |
| **Female** | 25.5  (24.7 to 26.3) | -9.3  (-10.6 to -7.9) | -26.7  (-30.6 to -22.8) | 19.3  (18.6 to 20.1) | -6.1  (-7.2 to -5.0) | -24.1  (-28.4 to -19.8) |
| **Race/ethnicity^d^** |  |  |  |  |  |  |
| **Non-Hispanic White** | 28.3  (27.5 to 29.2) | -10.3  (-11.7 to -8.9) | -26.7  (-30.2 to -23.1) | 22.0  (21.2 to 22.7) | -6.4  (-7.5 to -5.2) | -22.4  (-26.4 to -18.5) |
| **Non-Hispanic Black** | 19.7  (18.5 to 21.0) | -6.1  (-8.0 to -4.1) | -23.5  (-30.9 to -16.0) | 16.9  (15.8 to 18.0) | -2.8  (-4.4 to -1.2) | -14.2  (-22.5 to -5.9) |
| **Hispanic** | 16.1  (14.9 to 17.3) | -3.8  (-5.7 to -1.8) | -18.9  (-28.6 to -9.1) | 12.0  (11.0 to 13.0) | -4.1  (-5.7 to -2.5) | -25.4  (-35.1 to -15.6) |
| **Non-Hispanic Asian or Pacific Islander** | 12.5  (10.5 to 14.6) | -5.1  (-8.2 to -2.1) | -29.1  (-46.5 to -11.7) | 11.1  (9.2 to 12.9) | -1.5  (-4.2 to 1.3) | -11.8  (-33.8 to 10.2) |
| **Native American** | 20.9  (16.3 to 25.4) | -10.2  (-18.1 to -2.3) | -32.8  (-58.2 to -7.4) | 19.0  (14.9 to 23.1) | -1.9  (-8.0 to 4.2) | -9.1  (-38.5 to 20.2) |
| **Other** | 17.1  (14.9 to 19.3) | -5.9  (-9.6 to -2.1) | -25.6  (-42.0 to -9.2) | 16.2  (14.0 to 18.4) | -0.9  (-4.0 to 2.3) | -5.1  (-23.4 to 13.3) |
| **Zip code income quartile^e^** |  |  |  |  |  |  |
| **1 (lowest)** | 22.4  (21.4 to 23.4) | -8.0  (-9.6 to -6.4) | -26.3  (-31.7 to -21.0) | 18.0  (17.1 to 18.9) | -4.4  (-5.7 to -3.1) | -19.7  (-25.6 to -13.8) |
| **2** | 25.0  (24.0 to 26.0) | -9.7  (-11.4 to -8.1) | -28.0  (-32.8 to -23.2) | 19.3  (18.4 to 20.2) | -5.7  (-7.1 to -4.3) | -22.8  (-28.3 to -17.3) |
| **3** | 26.1  (25.0 to 27.2) | -9.6  (-11.4 to -7.9) | -27.0  (-31.9 to -22.1) | 19.9  (19.0 to 20.8) | -6.2  (-7.6 to -4.8) | -23.8  (-29.3 to -18.3) |
| **4 (highest)** | 26.9  (25.5 to 28.3) | -9.5  (-11.9 to -7.2) | -26.1  (-32.5 to -19.7) | 21.2  (19.9 to 22.4) | -5.7  (-7.6 to -3.9) | -21.3  (-28.2 to -14.4) |
| **Primary expected payer^f^** |  |  |  |  |  |  |
| **Medicare** | 29.1  (28.2 to 30.0) | -11.8  (-13.4 to -10.3) | -28.9  (-32.7 to -25.1) | 22.9  (22.0 to 23.7) | -6.2  (-7.5 to -5.0) | -21.4  (-25.8 to -17.1) |
| **Medicaid** | 20.6  (19.6 to 21.6) | -6.4  (-8.0 to -4.7) | -23.6  (-29.7 to -17.5) | 17.9  (17.0 to 18.9) | -2.7  (-4.1 to -1.3) | -12.9  (-19.7 to -6.1) |
| **Private insurance** | 20.7  (19.7 to 21.7) | -6.9  (-8.4 to -5.4) | -25.0  (-30.6 to -19.4) | 14.8  (14.0 to 15.6) | -5.9  (-7.2 to -4.6) | -28.5  (-34.7 to -22.3) |
| **Self-pay** | 21.2  (19.2 to 23.2) | -8.4  (-11.6 to -5.1) | -28.3  (-39.2 to -17.4) | 21.4  (19.3 to 23.5) | 0.2  (-2.7 to 3.1) | 0.9  (-12.8 to 14.6) |
| **No charge** | 30.1  (23.2 to 37.0) | 4.8  (-4.9 to 14.5) | 19.1  (-19.2 to 57.3) | 30.9  (21.3 to 40.5) | 0.8  (-11.0 to 12.7) | 2.7  (-36.7 to 42.1) |
| **Other** | 22.4  (19.9 to 24.8) | -9.5  (-13.7 to -5.3) | -29.8  (-42.9 to -16.8) | 17.0  (14.9 to 19.0) | -5.4  (-8.6 to -2.2) | -24.1  (-38.4 to -9.9) |
| **Patient Location^g^** |  |  |  |  |  |  |
| **"Central" counties of metro areas of >=1 million population** | 23.9  (22.6 to 25.3) | -7.1  (-9.3 to -4.9) | -22.8  (-29.9 to -15.7) | 19.3  (18.1 to 20.4) | -4.7  (-6.5 to -2.9) | -19.5  (-27.0 to -12.1) |
| **"Fringe" counties of metro areas of >=1 million population** | 27.2  (25.8 to 28.6) | -9.4  (-11.7 to -7.1) | -25.6  (-31.9 to -19.4) | 20.6  (19.5 to 21.7) | -6.6  (-8.4 to -4.8) | -24.2  (-30.8 to -17.6) |
| **Counties in metro areas of 250,000-999,999 population** | 24.7  (23.4 to 25.9) | -9.6  (-11.6 to -7.5) | -28.0  (-34.0 to -21.9) | 19.4  (18.2 to 20.6) | -5.3  (-7.0 to -3.5) | -21.3  (-28.5 to -14.2) |
| **Counties in metro areas of 50,000-249,999 population** | 24.3  (22.5 to 26.2) | -11.2  (-14.3 to -8.2) | -31.6  (-40.3 to -22.9) | 18.6  (17.0 to 20.1) | -5.7  (-8.1 to -3.3) | -23.6  (-33.4 to -13.8) |
| **Micropolitan counties** | 24.4  (22.8 to 26.1) | -10.8  (-13.5 to -8.1) | -30.6  (-38.3 to -23.0) | 19.4  (17.9 to 20.8) | -5.1  (-7.2 to -2.9) | -20.8  (-29.6 to -11.9) |
| **Not metropolitan or micropolitan counties** | 21.9  (20.2 to 23.5) | -10.1  (-12.8 to -7.4) | -31.5  (-40.0 to -23.0) | 17.5  (15.9 to 19.0) | -4.4  (-6.7 to -2.2) | -20.2  (-30.5 to -9.9) |

^a^ Cases of other opioids-related adverse event or poisoning were identified following the definition proposed by the Healthcare Cost and Utilization Project (ICD-10-CM code used: found in Table S 1).

^b^ Reflects the overall or total difference between 2016 and 2019: absolute difference (2019 – 2016) and percentage change [(2019 – 2016)/2016 × 100].

^c^ Reflects the overall or total difference during the COVID-19 pandemic in 2020 and 2021: absolute difference (2021 – 2019) and percentage change [(2021 – 2019)/2019 × 100].

^d^ Race/ethnicity variable was obtained from the Agency for Healthcare Research and Quality, Healthcare Cost and Utilization Project; nationally weighted from all records in states with reliable race/ethnicity reporting, which is collected by self-report on admission to the hospital using fixed categories.

^e^ Median household income of residents in the patient’s zip code was updated annually; 2019 range for quartile 1 was less than $48,000; quartile 2, $48,000 to $60,999; quartile 3, $61,000 to $81,999; and quartile 4, at least $82,000; 2021 range for quartile 1 was less than $52,000; quartile 2, $52,000 to $65,999; quartile 3, $66,000 to $87,999; and quartile 4, at least $88,000.

^f^ “Medicare” includes both fee-for-service and managed care Medicare patients. “Medicaid” includes both fee-for-service and managed care Medicaid patients. “Private insurance” includes Blue Cross, commercial carriers, and private HMOs and PPOs. “Other” includes Worker's Compensation, CHAMPUS (Civilian Health and Medical Program of the Uniformed Services), CHAMPVA (Civilian Health and Medical Program of the Department of Veteran's Affairs), Title V, and other government programs.

^g^ “Patient location” is a six-category urban-rural classification scheme for U.S. counties developed by the National Center for Health Statistics (NCHS) especially for use in health care research. The classification emphasizes urban distinctions and is unique in differentiating between central and fringe counties of large metropolitan areas. Smaller metropolitan counties are subdivided by population. Non-metropolitan counties are divided simply into micropolitan and non-core categories.

**Supplementary Table S28. Other Opioids-Related Adverse Event or Poisoning^a^ (2016 to 2019 and 2020 to 2021) in Nationwide Emergency Department Sample Overall and for Subtypes by Demographic Characteristics**

| **Characteristics** | **Between 2016 and 2019** | | | | **During the COVID-19 pandemic in 2020 and 2021** | | |
| --- | --- | --- | --- | --- | --- | --- | --- |
|  | **2019 Rate,**  **per 10,000** | **Absolute difference**  **from 2016-2019^b^** | **Change**  **from 2016-2019, %** | **2021 Rate,**  **per 10,000** | | **Absolute difference**  **from 2019-2021^c^** | **Change**  **from 2019-2021, %** |
| **Overall** | 8.0  (7.3 to 8.7) | -1.0  (-1.9 to -0.2) | -11.5  (-21.0 to -2.0) | 9.0  (8.4 to 9.5) | | 0.9  (0.05 to 1.8) | 11.7  (0.6 to 22.9) |
| **AGE** |  |  |  |  | |  |  |
| **18-44** | 6.1  (5.5 to 6.8) | 0.5  (-0.4 to 1.3) | 8.1  (-6.8 to 23.0) | 9.4  (8.7 to 10.2) | | 3.3  (2.3 to 4.3) | 53.7  (36.9 to 70.4) |
| **45-64** | 9.1  (7.9 to 10.3) | -2.1  (-3.5 to -0.7) | -18.9  (-31.2 to -6.6) | 9.1  (8.5 to 9.7) | | 0.02  (-1.4 to 1.4) | 0.2  (-15.1 to 15.6) |
| **65-84** | 10.4  (9.7 to 11.0) | -2.9  (-3.9 to -2.0) | -22.1  (-29.5 to -14.7) | 8.2  (7.8 to 8.7) | | -2.2  (-3.0 to -1.3) | -20.7  (-28.5 to -13.0) |
| **≥85** | 8.8  (8.0 to 9.5) | -4.2  (-5.5 to -2.9) | -32.4  (-42.1 to -22.8) | 7.2  (6.5 to 7.8) | | -1.6  (-2.6 to -0.6) | -18.0  (-29.1 to -6.9) |
| **Sex** |  |  |  |  | |  |  |
| **Male** | 9.5  (8.3 to 10.7) | -0.2  (-1.5 to 1.2) | -1.9  (-15.8 to 12.1) | 11.6  (10.8 to 12.4) | | 2.1  (0.6 to 3.5) | 21.7  (6.4 to 37.0) |
| **Female** | 6.9  (6.4 to 7.3) | -1.7  (-2.3 to -1.1) | -20.0  (-27.3 to -12.7) | 6.8  (6.4 to 7.2) | | 0  (-0.6 to 0.5) | -0.6  (-8.9 to 7.7) |
| **Race/ethnicity^e^** |  |  |  |  | |  |  |
| **Non-Hispanic White** | 9.8  (9.2 to 10.4) | NA^d^ | NA^d^ | 10.7  (10.1 to 11.4) | | 0.9  (0.1 to 1.8) | 9.6  (0.5 to 18.7) |
| **Non-Hispanic Black** | 5.9  (4.4 to 7.4) | NA^d^ | NA^d^ | 6.8  (6.1 to 7.5) | | 0.9  (-0.8 to 2.6) | 15.6  (-13.0 to 44.2) |
| **Hispanic** | 4.8  (3.8 to 5.9) | NA^d^ | NA^d^ | 5.9  (5.2 to 6.5) | | 1.0  (-0.2 to 2.3) | 21.3  (-4.9 to 47.5) |
| **Non-Hispanic Asian or Pacific Islander** | 6.3  (1.9 to 10.7) | NA^d^ | NA^d^ | 4.0  (3.4 to 4.6) | | -2.3  (-6.7 to 2.2) | -36.5  (-107.4 to 34.4) |
| **Native American** | 6.5  (5.0 to 8.1) | NA^d^ | NA^d^ | 15.2  (8.6 to 21.9) | | 8.7  (1.9 to 15.5) | 133.4  (28.8 to 237.9) |
| **Other** | 5.9  (5.0 to 6.7) | NA^d^ | NA^d^ | 7.6  (6.6 to 8.6) | | 1.7  (0.4 to 3.0) | 29.6  (7.2 to 52.0) |
| **Zip code income quartile^f^** |  |  |  |  | |  |  |
| **1 (lowest)** | 7.3  (6.3 to 8.4) | -0.4  (-1.6 to 0.8) | -5.0  (-20.9 to 10.8) | 8.9  (8.1 to 9.6) | | 1.5  (0.2 to 2.8) | 20.7  (2.7 to 38.7) |
| **2** | 7.7  (7.1 to 8.3) | -1.7  (-2.6 to -0.8) | -17.6  (-27.2 to -8.1) | 8.9  (8.2 to 9.7) | | 1.2  (0.2 to 2.2) | 15.4  (3.0 to 27.8) |
| **3** | 8.4  (7.7 to 9.1) | -1.4  (-2.3 to -0.4) | -14.0  (-23.6 to -4.3) | 8.9  (8.2 to 9.5) | | 0.5  (-0.5 to 1.4) | 5.3  (-6.0 to 16.7) |
| **4 (highest)** | 9.0  (8.1 to 9.9) | -1.3  (-2.5 to 0) | -12.4  (-24.4 to -0.3) | 8.6  (7.9 to 9.4) | | -0.4  (-1.5 to 0.8) | -3.9  (-17.1 to 9.3) |
| **Primary expected payer^g^** |  |  |  |  | |  |  |
| **Medicare** | 11.1  (10.5 to 11.8) | -3.7  (-4.6 to -2.7) | -24.7  (-31.3 to -18.1) | 9.2  (8.7 to 9.6) | | -2.0  (-2.7 to -1.2) | -17.8  (-24.7 to -10.9) |
| **Medicaid** | 8.2  (6.6 to 9.7) | 0.6  (-1.1 to 2.2) | 7.5  (-14.4 to 29.4) | 11.2  (10.2 to 12.2) | | 3.0  (1.1 to 4.8) | 36.5  (13.9 to 59.1) |
| **Private insurance** | 5.0  (4.6 to 5.5) | -1.2  (-1.8 to -0.6) | -18.9  (-28.3 to -9.6) | 5.2  (4.8 to 5.6) | | 0.1  (-0.4 to 0.7) | 2.4  (-8.9 to 13.6) |
| **Self-pay** | 7.6  (6.3 to 9.0) | 1.3  (-0.2 to 2.9) | 21.3  (-3.6 to 46.2) | 14.1  (12.5 to 15.7) | | 6.5  (4.4 to 8.6) | 84.8  (57.4 to 112.2) |
| **No charge** | 9.8  (6.4 to 13.2) | 0.1  (-7.4 to 7.7) | 1.1  (-76.7 to 78.9) | 20.9  (11.9 to 30.0) | | 11.1  (1.5 to 20.8) | 113.1  (14.8 to 211.5) |
| **Other** | 5.6  (4.5 to 6.7) | -0.8  (-2.2 to 0.6) | -12.7  (-34.4 to 8.9) | 6.0  (5.2 to 6.7) | | 0.3  (-1.0 to 1.7) | 5.9  (-17.6 to 29.4) |
| **Patient Location^h^** |  |  |  |  | |  |  |
| **"Central" counties of metro areas of >=1 million population** | 7.8  (5.8 to 9.7) | -0.4  (-2.5 to 1.7) | -4.8  (-30.9 to 21.4) | 8.5  (7.7 to 9.3) | | 0.7  (-1.4 to 2.8) | 9.2  (-17.7 to 36.2) |
| **"Fringe" counties of metro areas of >=1 million population** | 9.4  (8.3 to 10.4) | -0.7  (-2.1 to 0.7) | -6.9  (-20.8 to 7.0) | 10.6  (9.4 to 11.9) | | 1.3  (-0.4 to 2.9) | 13.3  (-4.3 to 31.0) |
| **Counties in metro areas of 250,000-999,999 population** | 7.9  (7.1 to 8.8) | -1.9  (-3.4 to -0.5) | -19.7  (-34.0 to -5.4) | 9.8  (8.4 to 11.2) | | 1.9  (0.2 to 3.5) | 23.3  (2.9 to 43.8) |
| **Counties in metro areas of 50,000-249,999 population** | 7.6  (6.7 to 8.5) | -1.1  (-2.4 to 0.2) | -12.6  (-27.5 to 2.2) | 8.3  (7.2 to 9.3) | | 0.7  (-0.7 to 2.0) | 9.1  (-8.7 to 26.8) |
| **Micropolitan counties** | 7.2  (6.1 to 8.3) | -1.9  (-3.3 to -0.4) | -20.9  (-36.9 to -4.8) | 7.1  (6.2 to 8.1) | | 0  (-1.5 to 1.4) | -0.7  (-20.7 to 19.3) |
| **Not metropolitan or micropolitan counties** | 5.9  (5.2 to 6.7) | -1.7  (-2.7 to -0.7) | -22.3  (-35.9 to -8.8) | 5.8  (5.1 to 6.4) | | -0.2  (-1.1 to 0.8) | -2.7  (-19.3 to 13.9) |

^a^ Cases of other opioids-related adverse event or poisoning were identified following the definition proposed by the Healthcare Cost and Utilization Project (ICD-10-CM code used: found in Table S 1).

^b^ Reflects the overall or total difference between 2016 and 2019: absolute difference (2019 – 2016) and percentage change [(2019 – 2016)/2016 × 100].

^c^ Reflects the overall or total difference during the COVID-19 pandemic in 2020 and 2021: absolute difference (2021 – 2019) and percentage change [(2021 – 2019)/2019 × 100].

^d^ NA, Not Available. Due to the NEDS survey design, the Race/ethnicity classification was not available until 2019 and therefore could not present estimates.

^e^ Race/ethnicity variable was obtained from the Agency for Healthcare Research and Quality, Healthcare Cost and Utilization Project; nationally weighted from all records in states with reliable race/ethnicity reporting, which is collected by self-report on admission to the hospital using fixed categories.

^f^ Median household income of residents in the patient’s zip code was updated annually; 2019 range for quartile 1 was less than $48,000; quartile 2, $48,000 to $60,999; quartile 3, $61,000 to $81,999; and quartile 4, at least $82,000; 2021 range for quartile 1 was less than $52,000; quartile 2, $52,000 to $65,999; quartile 3, $66,000 to $87,999; and quartile 4, at least $88,000.

^g^ “Medicare” includes both fee-for-service and managed care Medicare patients. “Medicaid” includes both fee-for-service and managed care Medicaid patients. “Private insurance” includes Blue Cross, commercial carriers, and private HMOs and PPOs. “Other” includes Worker's Compensation, CHAMPUS (Civilian Health and Medical Program of the Uniformed Services), CHAMPVA (Civilian Health and Medical Program of the Department of Veteran's Affairs), Title V, and other government programs.

^h^ “Patient location” is a six-category urban-rural classification scheme for U.S. counties developed by the National Center for Health Statistics (NCHS) especially for use in health care research. The classification emphasizes urban distinctions and is unique in differentiating between central and fringe counties of large metropolitan areas. Smaller metropolitan counties are subdivided by population. Non-metropolitan counties are divided simply into micropolitan and non-core categories.

**Supplementary Table S29. Nonfatal Opioid Overdose^a^ (2016 to 2019 and 2020 to 2021) in National Inpatient Sample Overall and for Subtypes by Demographic Characteristics**

| **Characteristics** | **Between 2016 and 2019** | | | **During the COVID-19 pandemic in 2020 and 2021** | | |
| --- | --- | --- | --- | --- | --- | --- |
|  | **2019 Rate,**  **per 10,000** | **Absolute difference**  **from 2016-2019^b^** | **Change**  **from 2016-2019, %** | **2021 Rate,**  **per 10,000** | **Absolute difference**  **from 2019-2021^c^** | **Change**  **from 2019-2021, %** |
| **Overall** | 27.1  (26.3 to 27.9) | -6.1  (-7.2 to -5.0) | -18.4  (-21.7 to -15.0) | 29.3  (28.3 to 30.2) | 2.2  (0.9 to 3.5) | 8.1  (3.5 to 12.7) |
| **AGE** |  |  |  |  |  |  |
| **18-44** | 41.2  (39.4 to 42.9) | -5.3  (-7.8 to -2.8) | -11.4  (-16.8 to -6.0) | 45.9  (44.0 to 47.7) | 4.7  (2.1 to 7.3) | 11.4  (5.0 to 17.8) |
| **45-64** | 37.0  (35.7 to 38.3) | -9.7  (-11.6 to -7.8) | -20.8  (-24.9 to -16.6) | 38.2  (36.7 to 39.8) | 1.2  (-0.9 to 3.3) | 3.2  (-2.4 to 8.9) |
| **65-84** | 14.1  (13.6 to 14.7) | -3.2  (-4.2 to -2.2) | -18.5  (-24.2 to -12.8) | 14.9  (14.2 to 15.5) | 0.8  (0 to 1.6) | 5.7  (-0.2 to 11.6) |
| **≥85** | 4.7  (4.1 to 5.3) | -1.0  (-1.8 to -0.2) | -17.5  (-32.1 to -3.0) | 4.5  (3.9 to 5.1) | -0.2  (-1.0 to 0.6) | -4.3  (-21.9 to 13.4) |
| **Sex** |  |  |  |  |  |  |
| **Male** | 33.4  (32.3 to 34.6) | -5.2  (-6.9 to -3.5) | -13.5  (-17.8 to -9.2) | 38.8  (37.3 to 40.2) | 5.4  (3.6 to 7.2) | 16.2  (10.8 to 21.6) |
| **Female** | 22.3  (21.6 to 23.0) | -7.0  (-8.1 to -5.9) | -23.9  (-27.7 to -20.1) | 21.8  (21.1 to 22.6) | -0.5  (-1.6 to 0.6) | -2.2  (-7.2 to 2.7) |
| **Race/ethnicity^d^** |  |  |  |  |  |  |
| **Non-Hispanic White** | 29.7  (28.8 to 30.5) | -8.7  (-10.0 to -7.4) | -22.7  (-25.9 to -19.4) | 30.1  (29.1 to 31.0) | 0.4  (-0.9 to 1.7) | 1.3  (-2.9 to 5.6) |
| **Non-Hispanic Black** | 24.4  (22.5 to 26.2) | -0.1  (-2.6 to 2.4) | -0.4  (-10.6 to 9.8) | 32.2  (29.8 to 34.7) | 7.8  (4.7 to 10.9) | 32.0  (19.3 to 44.7) |
| **Hispanic** | 20.3  (18.7 to 21.9) | -0.8  (-3.0 to 1.4) | -3.8  (-14.3 to 6.7) | 24.3  (22.5 to 26.1) | 4.0  (1.6 to 6.4) | 19.7  (8.1 to 31.3) |
| **Non-Hispanic Asian or Pacific Islander** | 8.1  (6.6 to 9.6) | -0.1  (-2.2 to 2.0) | -1.2  (-26.6 to 24.2) | 7.7  (6.2 to 9.2) | -0.4  (-2.6 to 1.8) | -4.9  (-32.3 to 22.4) |
| **Native American** | 33.6  (27.7 to 39.4) | -4.5  (-14.1 to 5.1) | -11.8  (-37.1 to 13.5) | 36.4  (28.2 to 44.7) | 2.8  (-7.3 to 12.9) | 8.3  (-21.8 to 38.4) |
| **Other** | 25.6  (22.8 to 28.4) | 5.0  (1.3 to 8.7) | 24.3  (6.1 to 42.4) | 32.1  (28.1 to 36.2) | 6.5  (1.7 to 11.3) | 25.4  (6.7 to 44.1) |
| **Zip code income quartile^e^** |  |  |  |  |  |  |
| **1 (lowest)** | 30.4  (29.2 to 31.7) | -6.1  (-8.0 to -4.2) | -16.7  (-22.0 to -11.4) | 34.7  (33.1 to 36.3) | 4.3  (2.2 to 6.4) | 14.1  (7.3 to 21.0) |
| **2** | 26.7  (25.6 to 27.7) | -7  (-8.5 to -5.5) | -20.8  (-25.3 to -16.2) | 28.9  (27.7 to 30.1) | 2.2  (0.7 to 3.7) | 8.2  (2.5 to 14) |
| **3** | 25.6  (24.5 to 26.7) | -6.7  (-8.4 to -5.0) | -20.7  (-25.9 to -15.6) | 26.3  (25.1 to 27.4) | 0.7  (-1.0 to 2.4) | 2.7  (-3.8 to 9.2) |
| **4 (highest)** | 22.3  (21.1 to 23.5) | -5.5  (-7.3 to -3.7) | -19.8  (-26.3 to -13.3) | 21.2  (20.1 to 22.3) | -1.1  (-2.8 to 0.6) | -4.9  (-12.4 to 2.5) |
| **Primary expected payer^f^** |  |  |  |  |  |  |
| **Medicare** | 19.0  (18.4 to 19.6) | -6.8  (-7.8 to -5.8) | -26.4  (-30.2 to -22.6) | 18.2  (17.6 to 18.9) | -0.8  (-1.6 to 0) | -4.2  (-8.6 to 0.2) |
| **Medicaid** | 50.1  (47.8 to 52.4) | -5.5  (-8.8 to -2.2) | -9.9  (-15.9 to -3.9) | 58.2  (55.5 to 60.8) | 8.1  (4.5 to 11.7) | 16.2  (9.0 to 23.4) |
| **Private insurance** | 17.9  (17.1 to 18.7) | -5.9  (-7.2 to -4.6) | -24.8  (-30.1 to -19.5) | 18.1  (17.2 to 19.1) | 0.2  (-1.1 to 1.5) | 1.1  (-5.9 to 8.1) |
| **Self-pay** | 71.8  (67.2 to 76.4) | -5.0  (-11.5 to 1.5) | -6.5  (-15.0 to 2.0) | 85.6  (80.2 to 90.9) | 13.8  (6.6 to 21.0) | 19.2  (9.2 to 29.3) |
| **No charge** | 95.1  (78.8 to 111.3) | 16.1  (-7.0 to 39.2) | 20.4  (-8.9 to 49.7) | 115.3  (93.1 to 137.6) | 20.2  (-7.3 to 47.7) | 21.2  (-7.7 to 50.1) |
| **Other** | 27.0  (24.4 to 29.7) | -9.0  (-13.6 to -4.4) | -25.0  (-37.8 to -12.2) | 27.6  (24.9 to 30.3) | 0.6  (-3.3 to 4.5) | 2.2  (-12.2 to 16.6) |
| **Patient Location^g^** |  |  |  |  |  |  |
| **"Central" counties of metro areas of >=1 million population** | 28.1  (26.5 to 29.8) | -3.5  (-5.9 to -1.1) | -11.1  (-18.5 to -3.6) | 32.3  (30.3 to 34.4) | 4.2  (1.5 to 6.9) | 14.9  (5.5 to 24.4) |
| **"Fringe" counties of metro areas of >=1 million population** | 27.9  (26.4 to 29.3) | -6.1  (-8.2 to -4.0) | -17.9  (-24.1 to -11.8) | 27.1  (25.6 to 28.5) | -0.8  (-2.7 to 1.1) | -2.9  (-9.8 to 4.1) |
| **Counties in metro areas of 250,000-999,999 population** | 28.4  (27 to 29.8) | -8.1  (-10.3 to -5.9) | -22.2  (-28.3 to -16.1) | 30.8  (29.2 to 32.5) | 2.4  (0.2 to 4.6) | 8.5  (0.6 to 16.3) |
| **Counties in metro areas of 50,000-249,999 population** | 25.4  (23.8 to 27.1) | -8.0  (-10.5 to -5.5) | -24.0  (-31.5 to -16.4) | 26.1  (24.3 to 27.9) | 0.7  (-1.7 to 3.1) | 2.8  (-6.5 to 12.0) |
| **Micropolitan counties** | 22.5  (21.1 to 24.0) | -9.3  (-11.5 to -7.1) | -29.2  (-36.3 to -22.2) | 23.5  (22 to 25.1) | 1.0  (-1.1 to 3.1) | 4.4  (-4.8 to 13.7) |
| **Not metropolitan or micropolitan counties** | 18.8  (17.2 to 20.3) | -8.7  (-11.2 to -6.2) | -31.6  (-40.8 to -22.5) | 20 .0  (18.4 to 21.6) | 1.2  (-1.0 to 3.4) | 6.4  (-5.4 to 18.2) |
| **Hospital region** |  |  |  |  |  |  |
| **Northeast** | 28.8  (26.8 to 30.7) | -3.8  (-6.7 to -0.9) | -11.7  (-20.6 to -2.7) | 30.6  (27.9 to 33.2) | 1.8  (-1.4 to 5.0) | 6.3  (-4.9 to 17.4) |
| **Midwest** | 26.2  (24.5 to 27.9) | -8.8  (-11.4 to -6.2) | -25.1  (-32.7 to -17.6) | 27.0  (24.9 to 29.1) | 0.8  (-2.0 to 3.6) | 3.1  (-7.6 to 13.7) |
| **South** | 27.2  (25.9 to 28.4) | -5.8  (-7.6 to -4.0) | -17.6  (-23.1 to -12.1) | 29.3  (27.9 to 30.6) | 2.1  (0.3 to 3.9) | 7.7  (1.1 to 14.4) |
| **West** | 26.4  (24.8 to 28.0) | -5.8  (-8.0 to -3.6) | -18.0  (-24.9 to -11.1) | 30.7  (28.7 to 32.7) | 4.3  (1.8 to 6.8) | 16.3  (6.8 to 25.8) |

^a^ Cases of nonfatal opioid overdose were identified following the study conducted by Barnett et al. (ICD-10-CM code used: found in Table S 3).

^b^ Reflects the overall or total difference between 2016 and 2019: absolute difference (2019 – 2016) and percentage change [(2019 – 2016)/2016 × 100].

^c^ Reflects the overall or total difference during the COVID-19 pandemic in 2020 and 2021: absolute difference (2021 – 2019) and percentage change [(2021 – 2019)/2019 × 100].

^d^ Race/ethnicity variable was obtained from the Agency for Healthcare Research and Quality, Healthcare Cost and Utilization Project; nationally weighted from all records in states with reliable race/ethnicity reporting, which is collected by self-report on admission to the hospital using fixed categories.

^e^ Median household income of residents in the patient’s zip code was updated annually; 2019 range for quartile 1 was less than $48,000; quartile 2, $48,000 to $60,999; quartile 3, $61,000 to $81,999; and quartile 4, at least $82,000; 2021 range for quartile 1 was less than $52,000; quartile 2, $52,000 to $65,999; quartile 3, $66,000 to $87,999; and quartile 4, at least $88,000.

^f^ “Medicare” includes both fee-for-service and managed care Medicare patients. “Medicaid” includes both fee-for-service and managed care Medicaid patients. “Private insurance” includes Blue Cross, commercial carriers, and private HMOs and PPOs. “Other” includes Worker's Compensation, CHAMPUS (Civilian Health and Medical Program of the Uniformed Services), CHAMPVA (Civilian Health and Medical Program of the Department of Veteran's Affairs), Title V, and other government programs.

^g^ “Patient location” is a six-category urban-rural classification scheme for U.S. counties developed by the National Center for Health Statistics (NCHS) especially for use in health care research. The classification emphasizes urban distinctions and is unique in differentiating between central and fringe counties of large metropolitan areas. Smaller metropolitan counties are subdivided by population. Non-metropolitan counties are divided simply into micropolitan and non-core categories.

**Supplementary Table S30. Injection Drug-Use Related Acute Infection^a^ (2016 to 2019 and 2020 to 2021) in National Inpatient Sample Overall and for Subtypes by Demographic Characteristics**

| **Characteristics** | **Between 2016 and 2019** | | | **During the COVID-19 pandemic in 2020 and 2021** | | |
| --- | --- | --- | --- | --- | --- | --- |
|  | **2019 Rate,**  **per 10,000** | **Absolute difference**  **from 2016-2019^b^** | **Change**  **from 2016-2019, %** | **2021 Rate,**  **per 10,000** | **Absolute difference**  **from 2019-2021^c^** | **Change**  **from 2019-2021, %** |
| **Overall** | 15.9  (15.2 to 16.6) | 2.0  (1.0 to 3.0) | 14.4  (7.3 to 21.4) | 17.2  (16.4 to 18.0) | 1.3  (0.2 to 2.4) | 8.2  (1.2 to 15.1) |
| **AGE** |  |  |  |  |  |  |
| **18-44** | 36.0  (34.1 to 37.8) | 4.9  (2.4 to 7.4) | 15.8  (7.7 to 23.8) | 36.1  (34.1 to 38.0) | 0.1  (-2.7 to 2.9) | 0.3  (-7.4 to 8.0) |
| **45-64** | 18.4  (17.4 to 19.3) | 3.1  (1.7 to 4.5) | 20.3  (11.2 to 29.3) | 21.3  (20.1 to 22.5) | 2.9  (1.4 to 4.4) | 15.8  (7.4 to 24.1) |
| **65-84** | 2.4  (2.1 to 2.6) | 0.9  (0.6 to 1.2) | 60.0  (41.5 to 78.5) | 2.9  (2.6 to 3.1) | 0.5  (0.2 to 0.8) | 20.8  (9.3 to 32.4) |
| **≥85** | 0.04  (-0.01 to 0.08) | -0.1  (-0.2 to 0.03) | -66.7  (-161.9 to 28.6) | 0.16  (0.05 to 0.27) | 0.1  (-0.01 to 0.3) | 300.0  (-28.7 to 628.7) |
| **Sex** |  |  |  |  |  |  |
| **Male** | 22.2  (21.1 to 23.2) | 2.6  (1.2 to 4.0) | 13.3  (6.2 to 20.3) | 24.4  (23.2 to 25.6) | 2.2  (0.7 to 3.7) | 9.9  (3.0 to 16.8) |
| **Female** | 11.2  (10.6 to 11.8) | 1.4  (0.6 to 2.2) | 14.3  (5.8 to 22.8) | 11.6  (11.0 to 12.2) | 0.4  (-0.4 to 1.2) | 3.6  (-3.9 to 11.0) |
| **Race/ethnicity^e^** |  |  |  |  |  |  |
| **Non-Hispanic White** | 17.6  (16.8 to 18.5) | 2.5  (1.4 to 3.6) | 16.6  (9.2 to 23.9) | 18.9  (18.0 to 19.8) | 1.3  (0.04 to 2.6) | 7.4  (0.3 to 14.5) |
| **Non-Hispanic Black** | 11.0  (9.9 to 12.1) | -0.1  (-1.6 to 1.4) | -0.9  (-14.7 to 12.9) | 13.3  (11.9 to 14.6) | 2.3  (0.6 to 4.0) | 20.9  (5.6 to 36.2) |
| **Hispanic** | 16.7  (15.2 to 18.2) | 2.0  (-0.2 to 4.2) | 13.6  (-1.5 to 28.7) | 16.8  (15.2 to 18.4) | 0.1  (-2.1 to 2.3) | 0.6  (-12.7 to 13.9) |
| **Non-Hispanic Asian or Pacific Islander** | 2.5  (1.5 to 3.5) | -0.1  (-1.5 to 1.3) | -3.8  (-57.2 to 49.5) | 3.3  (2.3 to 4.3) | 0.8  (-0.6 to 2.2) | 32.0  (-23.4 to 87.4) |
| **Native American** | 28.3  (20.1 to 36.6) | 5.6  (-4.6 to 15.8) | 24.7  (-20.4 to 69.7) | 31.1  (23.7 to 38.5) | 2.8  (-8.3 to 13.9) | 9.9  (-29.3 to 49.1) |
| **Other** | 12.6  (10.6 to 14.6) | 2.9  (0.4 to 5.4) | 29.9  (4.0 to 55.8) | 15.1  (12.7 to 17.6) | 2.5  (-0.6 to 5.6) | 19.8  (-4.5 to 44.1) |
| **Zip code income quartile^f^** |  |  |  |  |  |  |
| **1 (lowest)** | 19.4  (18.2 to 20.6) | 1.8  (0.1 to 3.5) | 10.2  (0.8 to 19.7) | 22.2  (20.7 to 23.7) | 2.8  (0.8 to 4.8) | 14.4  (4.3 to 24.5) |
| **2** | 15.1  (14.2 to 15.9) | 1.6  (0.5 to 2.7) | 11.9  (3.6 to 20.1) | 16.4  (15.4 to 17.3) | 1.3  (0.04 to 2.6) | 8.6  (0.3 to 16.9) |
| **3** | 13.3  (12.5 to 14.1) | 1.0  (-0.1 to 2.1) | 8.1  (-0.9 to 17.1) | 13.4  (12.5 to 14.3) | 0.1  (-1.0 to 1.2) | 0.8  (-7.6 to 9.1) |
| **4 (highest)** | 9.9  (9.2 to 10.7) | 0.6  (-0.5 to 1.7) | 6.5  (-5.5 to 18.4) | 10.6  (9.8 to 11.4) | 0.7  (-0.4 to 1.8) | 7.1  (-4.1 to 18.3) |
| **Primary expected payer^g^** |  |  |  |  |  |  |
| **Medicare** | 4.9  (4.6 to 5.2) | 0.7  (0.3 to 1.1) | 16.7  (6.2 to 27.1) | 5.5  (5.1 to 5.8) | 0.6  (0.05 to 1.2) | 12.2  (0.9 to 23.6) |
| **Medicaid** | 49.2  (46.6 to 51.8) | 7.3  (3.8 to 10.8) | 17.4  (9.1 to 25.7) | 51.6  (48.8 to 54.5) | 2.4  (-1.3 to 6.1) | 4.9  (-2.7 to 12.5) |
| **Private insurance** | 5.9  (5.5 to 6.4) | -0.2  (-0.8 to 0.4) | -3.3  (-12.4 to 5.8) | 6.4  (5.9 to 6.9) | 0.5  (-0.2 to 1.2) | 8.5  (-3.5 to 20.5) |
| **Self-pay** | 56.1  (51.5 to 60.7) | 4.8  (-1.4 to 11) | 9.4  (-2.8 to 21.5) | 58.9  (53.0 to 64.7) | 2.8  (-4.6 to 10.2) | 5.0  (-8.2 to 18.2) |
| **No charge** | 75.0  (58.0 to 92.0) | 19.4  (-2.0 to 40.8) | 34.9  (-3.6 to 73.4) | 59.1  (46 to 72.1) | -15.9  (-37.4 to 5.6) | -21.2  (-49.9 to 7.5) |
| **Other** | 15.3  (12.7 to 18.0) | 3.5  (0.1 to 6.9) | 29.7  (1.1 to 58.2) | 13.0  (11.1 to 15.0) | -2.3  (-5.7 to 1.1) | -15  (-37.1 to 7.0) |
| **Patient Location^h^** |  |  |  |  |  |  |
| **"Central" counties of metro areas of >=1 million population** | 17.5  (16.2 to 18.8) | 0.2  (-1.7 to 2.1) | 1.2  (-10.1 to 12.4) | 18.5  (16.9 to 20.1) | 1.0  (-1.1 to 3.1) | 5.7  (-6.2 to 17.6) |
| **"Fringe" counties of metro areas of >=1 million population** | 14.2  (13.2 to 15.3) | 0.9  (-0.6 to 2.4) | 6.8  (-4.7 to 18.3) | 14.5  (13.3 to 15.6) | 0.3  (-1.4 to 2) | 2.1  (-9.6 to 13.8) |
| **Counties in metro areas of 250,000-999,999 population** | 16.9  (15.5 to 18.4) | 2.6  (0.8 to 4.4) | 18.2  (5.5 to 30.8) | 18.6  (17 to 20.3) | 1.7  (-0.4 to 3.8) | 10.1  (-2.3 to 22.4) |
| **Counties in metro areas of 50,000-249,999 population** | 12.5  (11.1 to 13.9) | 2.5  (0.7 to 4.3) | 25.0  (6.9 to 43.1) | 15.4  (13.6 to 17.2) | 2.9  (0.7 to 5.1) | 23.2  (5.3 to 41.1) |
| **Micropolitan counties** | 12.2  (10.9 to 13.6) | 2.2  (0.4 to 4.0) | 22.0  (3.9 to 40.1) | 14.4  (13.0 to 15.9) | 2.2  (0.1 to 4.3) | 18.0  (1.0 to 35.1) |
| **Not metropolitan or micropolitan counties** | 8.7  (7.5 to 10.0) | 0.9  (-0.9 to 2.7) | 11.5  (-11.6 to 34.7) | 10.1  (8.6 to 11.6) | 1.4  (-0.6 to 3.4) | 16.1  (-6.4 to 38.6) |
| **Hospital region** |  |  |  |  |  |  |
| **Northeast** | 16.0  (14.5 to 17.5) | 0.9  (-1.3 to 3.1) | 6.0  (-8.7 to 20.6) | 18.9  (16.5 to 21.3) | 2.9  (0.1 to 5.7) | 18.1  (0.5 to 35.8) |
| **Midwest** | 12.8  (11.5 to 14.0) | 0.4  (-1.4 to 2.2) | 3.2  (-11.3 to 17.8) | 13.8  (12.6 to 15) | 1.0  (-0.7 to 2.7) | 7.8  (-5.2 to 20.8) |
| **South** | 14.5  (13.3 to 15.7) | 3.3  (1.8 to 4.8) | 29.5  (15.8 to 43.1) | 15.8  (14.6 to 17.1) | 1.3  (-0.4 to 3.0) | 9.0  (-2.5 to 20.4) |
| **West** | 22.2  (20.5 to 24.0) | 2.1  (-0.4 to 4.6) | 10.4  (-2.0 to 22.9) | 22.6  (20.8 to 24.4) | 0.4  (-2.1 to 2.9) | 1.8  (-9.4 to 13.0) |

^a^ Cases of injection drug-use related acute infection were identified following the study conducted by Barnett et al.(ICD-10-CM code used: found in Table S 3).

^b^ Reflects the overall or total difference between 2016 and 2019: absolute difference (2019 – 2016) and percentage change [(2019 – 2016)/2016 × 100].

^c^ Reflects the overall or total difference during the COVID-19 pandemic in 2020 and 2021: absolute difference (2021 – 2019) and percentage change [(2021 – 2019)/2019 × 100].

^d^ NR, Not Reportable. Suppressed to protect confidentiality, ≤10 cases.

^e^ Race/ethnicity variable was obtained from the Agency for Healthcare Research and Quality, Healthcare Cost and Utilization Project; nationally weighted from all records in states with reliable race/ethnicity reporting, which is collected by self-report on admission to the hospital using fixed categories.

^f^ Median household income of residents in the patient’s zip code was updated annually; 2019 range for quartile 1 was less than $48,000; quartile 2, $48,000 to $60,999; quartile 3, $61,000 to $81,999; and quartile 4, at least $82,000; 2021 range for quartile 1 was less than $52,000; quartile 2, $52,000 to $65,999; quartile 3, $66,000 to $87,999; and quartile 4, at least $88,000.

^g^ “Medicare” includes both fee-for-service and managed care Medicare patients. “Medicaid” includes both fee-for-service and managed care Medicaid patients. “Private insurance” includes Blue Cross, commercial carriers, and private HMOs and PPOs. “Other” includes Worker's Compensation, CHAMPUS (Civilian Health and Medical Program of the Uniformed Services), CHAMPVA (Civilian Health and Medical Program of the Department of Veteran's Affairs), Title V, and other government programs.

^h^ “Patient location” is a six-category urban-rural classification scheme for U.S. counties developed by the National Center for Health Statistics (NCHS) especially for use in health care research. The classification emphasizes urban distinctions and is unique in differentiating between central and fringe counties of large metropolitan areas. Smaller metropolitan counties are subdivided by population. Non-metropolitan counties are divided simply into micropolitan and non-core categories.

**Supplementary Table S31. Substance abuse Treatment^a^ (2016 to 2019 and 2020 to 2021) in National Inpatient Sample Overall and for Subtypes by Demographic Characteristics**

| **Characteristics** | **Between 2016 and 2019** | | | **During the COVID-19 pandemic in 2020 and 2021** | | |
| --- | --- | --- | --- | --- | --- | --- |
|  | **2019 Rate,**  **per 10,000** | **Absolute difference**  **from 2016-2019^b^** | **Change**  **from 2016-2019, %** | **2021 Rate,**  **per 10,000** | **Absolute difference**  **from 2019-2021^c^** | **Change**  **from 2019-2021, %** |
| **Overall** | 25.8  (21.2 to 30.3) | -8.6  (-15.8 to -1.5) | -25.1  (-45.9 to -4.3) | 22.8  (19.1 to 26.6) | -3.0  (-8.9 to 2.9) | -11.5  (-34.4 to 11.3) |
| **AGE** |  |  |  |  |  |  |
| **18-44** | 59.2  (49.4 to 68.9) | -19.9  (-35.9 to -4.0) | -25.2  (-45.3 to -5.1) | 51.3  (43.1 to 59.6) | -7.8  (-20.7 to 5.0) | -13.2  (-34.9 to 8.4) |
| **45-64** | 30.3  (23.3 to 37.2) | -6.7  (-16.9 to 3.4) | -18.2  (-45.7 to 9.3) | 25.6  (20.2 to 31.0) | -4.7  (-13.5 to 4.1) | -15.5  (-44.6 to 13.7) |
| **65-84** | 2.7  (2.2 to 3.3) | 0.2  (-0.6 to 0.9) | 7.0  (-22.2 to 36.3) | 2.8  (2.3 to 3.4) | 0.1  (-0.7 to 0.9) | 3.3  (-25.6 to 32.1) |
| **≥85** | NR^d^ | NR^d^ | NR^d^ | NR^d^ | NR^d^ | NR^d^ |
| **Sex** |  |  |  |  |  |  |
| **Male** | 38.9  (31.4 to 46.3) | -13.2  (-24.9 to -1.4) | -25.3  (-47.9 to -2.8) | 34.0  (27.8 to 40.2) | -4.8  (-14.5 to 4.9) | -12.5  (-37.4 to 12.5) |
| **Female** | 15.9  (13.4 to 18.4) | -5.8  (-10.0 to -1.7) | -26.8  (-45.9 to -7.7) | 14.0  (12.0 to 16.1) | -1.9  (-5.1 to 1.3) | -11.8  (-32.0 to 8.4) |
| **Race/ethnicity^e^** |  |  |  |  |  |  |
| **Non-Hispanic White** | 26.1  (22.0 to 30.1) | -8.5  (-15.3 to -1.7) | -24.6  (-44.2 to -4.9) | 24.3  (20.6 to 27.9) | -1.8  (-7.2 to 3.6) | -6.9  (-27.8 to 14.0) |
| **Non-Hispanic Black** | 26.8  (18.5 to 35.0) | -10.5  (-24.3 to 3.3) | -28.2  (-65.3 to 9.0) | 22.6  (16.4 to 28.7) | -4.2  (-14.5 to 6.1) | -15.7  (-54.3 to 22.8) |
| **Hispanic** | 25.3  (14.4 to 36.3) | -5.2  (-20.4 to 10.0) | -17  (-66.7 to 32.7) | 20.0  (11.5 to 28.4) | -5.4  (-19.2 to 8.4) | -21.3  (-75.7 to 33.2) |
| **Non-Hispanic Asian or Pacific Islander** | 5.4  (2.9 to 7.9) | -2.6  (-8.4 to 3.2) | -32.9  (-105.0 to 39.3) | 4.4  (2.7 to 6.2) | -1.0  (-4.0 to 2.1) | -17.8  (-74.6 to 39.0) |
| **Native American** | 47.2  (26.5 to 67.9) | -3.7  (-36.3 to 28.9) | -7.3  (-71.3 to 56.7) | 34.9  (17.3 to 52.5) | -12.3  (-39.5 to 14.8) | -26.1  (-83.6 to 31.4) |
| **Other** | 38.3  (22.6 to 54.0) | -8.8  (-32.5 to 14.9) | -18.7  (-69.0 to 31.7) | 26.2  (17.9 to 34.5) | -12.1  (-29.9 to 5.6) | -31.6  (-77.9 to 14.7) |
| **Zip code income quartile^f^** |  |  |  |  |  |  |
| **1 (lowest)** | 28.2  (21.5 to 35.0) | -10.0  (-20.4 to 0.4) | -26.2  (-53.4 to 1.0) | 25.5  (20.5 to 30.5) | -2.7  (-11.1 to 5.7) | -9.7  (-39.5 to 20.1) |
| **2** | 22.1  (18.1 to 26.1) | -7.3  (-13.7 to -0.9) | -24.9  (-46.6 to -3.2) | 19.6  (15.9 to 23.2) | -2.6  (-8.0 to 2.9) | -11.6  (-36.0 to 12.9) |
| **3** | 23.6  (19.6 to 27.7) | -6.9  (-13.2 to -0.5) | -22.5  (-43.4 to -1.6) | 19.3  (16.2 to 22.3) | -4.3  (-9.4 to 0.7) | -18.3  (-39.7 to 3.1) |
| **4 (highest)** | 29.0  (21.3 to 36.8) | -8.8  (-20.7 to 3.1) | -23.3  (-54.7 to 8.2) | 24.2  (17.6 to 30.8) | -4.9  (-15.1 to 5.3) | -16.8  (-51.8 to 18.2) |
| **Primary expected payer^g^** |  |  |  |  |  |  |
| **Medicare** | 7.4  (6.2 to 8.5) | -1.9  (-3.7 to 0) | -20.2  (-40.0 to -0.4) | 6.6  (5.6 to 7.6) | -0.8  (-2.3 to 0.7) | -10.4  (-30.8 to 9.9) |
| **Medicaid** | 82.7  (63.9 to 101.5) | -12.7  (-38.6 to 13.2) | -13.3  (-40.4 to 13.8) | 69.7  (55.1 to 84.2) | -13.1  (-36.8 to 10.7) | -15.8  (-44.5 to 12.9) |
| **Private insurance** | 18.1  (14.3 to 22.0) | -12.7  (-21.1 to -4.4) | -41.2  (-68.3 to -14.1) | 15.6  (12.2 to 19.0) | -2.5  (-7.6 to 2.6) | -13.9  (-42.2 to 14.4) |
| **Self-pay** | 31.9  (25.8 to 38.0) | -18.7  (-30.4 to -7.1) | -37.0  (-60.0 to -13.9) | 30.8  (24.1 to 37.5) | -1.1  (-10.2 to 7.9) | -3.5  (-31.9 to 24.8) |
| **No charge** | 111.3  (-16.4 to 239.1) | -18.3  (-230.6 to 194.0) | -14.1  (-177.9 to 149.7) | 100.4  (1.3 to 199.6) | -10.9  (-172.6 to 150.8) | -9.8  (-155.0 to 135.5) |
| **Other** | 26.9  (15.1 to 38.7) | -24.5  (-49.4 to 0.3) | -47.7  (-96.0 to 0.7) | 16.8  (10.1 to 23.5) | -10.1  (-23.7 to 3.5) | -37.6  (-88.1 to 13.0) |
| **Patient Location^h^** |  |  |  |  |  |  |
| **"Central" counties of metro areas of >=1 million population** | 37.1  (26.5 to 47.7) | -10.8  (-26.6 to 5.0) | -22.5  (-55.6 to 10.5) | 29.3  (21.7 to 37.0) | -7.7  (-20.8 to 5.3) | -20.8  (-56.0 to 14.4) |
| **"Fringe" counties of metro areas of >=1 million population** | 27.1  (19.8 to 34.4) | -8.4  (-19.8 to 3.0) | -23.6  (-55.6 to 8.3) | 25.0  (18.4 to 31.6) | -2.1  (-12.0 to 7.7) | -7.8  (-44.1 to 28.5) |
| **Counties in metro areas of 250,000-999,999 population** | 18.4  (14.2 to 22.7) | -7.0  (-14.1 to 0.2) | -27.4  (-55.5 to 0.7) | 17.2  (13.5 to 20.9) | -1.3  (-6.9 to 4.4) | -6.8  (-37.5 to 23.8) |
| **Counties in metro areas of 50,000-249,999 population** | 21.4  (12.3 to 30.4) | -6.8  (-20.7 to 7.1) | -24.1  (-73.7 to 25.4) | 19.4  (10.9 to 27.9) | -2.0  (-14.4 to 10.4) | -9.3  (-67.3 to 48.8) |
| **Micropolitan counties** | 16.7  (13.0 to 20.5) | -8.0  (-15.5 to -0.5) | -32.3  (-62.7 to -2.0) | 14.5  (11.5 to 17.5) | -2.3  (-7.0 to 2.5) | -13.5  (-42.0 to 15.0) |
| **Not metropolitan or micropolitan counties** | 12.3  (8.9 to 15.7) | -4.4  (-10.3 to 1.4) | -26.5  (-61.7 to 8.6) | 11.3  (8.4 to 14.2) | -1.0  (-5.4 to 3.5) | -7.8  (-44.4 to 28.7) |
| **Hospital region** |  |  |  |  |  |  |
| **Northeast** | 70.0  (48.2 to 91.7) | -12.7  (-44.3 to 18.9) | -15.4  (-53.6 to 22.9) | 56.2  (39.1 to 73.3) | -13.8  (-41.4 to 13.8) | -19.7  (-59.2 to 19.8) |
| **Midwest** | 19.6  (13.7 to 25.4) | -14.9  (-28.0 to -1.8) | -43.2  (-81.3 to -5.1) | 15.9  (11.4 to 20.4) | -3.7  (-11.1 to 3.7) | -18.9  (-56.7 to 18.9) |
| **South** | 15.2  (11.5 to 18.8) | -4.7  (-10.9 to 1.5) | -23.6  (-54.5 to 7.3) | 16.1  (12.2 to 19.9) | 0.9  (-4.5 to 6.3) | 5.9  (-29.7 to 41.5) |
| **West** | 12.8  (8.2 to 17.4) | -4.2  (-11.8 to 3.4) | -24.7  (-69.2 to 19.8) | 13.1  (7.9 to 18.2) | 0.3  (-6.5 to 7.1) | 2.3  (-50.8 to 55.5) |

^a^ Cases of substance abuse treatment were identified following the study conducted by Barnett et al.(ICD-10-CM code used: found in Table S 3).

^b^ Reflects the overall or total difference between 2016 and 2019: absolute difference (2019 – 2016) and percentage change [(2019 – 2016)/2016 × 100].

^c^ Reflects the overall or total difference during the COVID-19 pandemic in 2020 and 2021: absolute difference (2021 – 2019) and percentage change [(2021 – 2019)/2019 × 100].

^d^ NR, Not Reportable. Suppressed to protect confidentiality, ≤10 cases.

^e^ Race/ethnicity variable was obtained from the Agency for Healthcare Research and Quality, Healthcare Cost and Utilization Project; nationally weighted from all records in states with reliable race/ethnicity reporting, which is collected by self-report on admission to the hospital using fixed categories.

^f^ Median household income of residents in the patient’s zip code was updated annually; 2019 range for quartile 1 was less than $48,000; quartile 2, $48,000 to $60,999; quartile 3, $61,000 to $81,999; and quartile 4, at least $82,000; 2021 range for quartile 1 was less than $52,000; quartile 2, $52,000 to $65,999; quartile 3, $66,000 to $87,999; and quartile 4, at least $88,000.

^g^ “Medicare” includes both fee-for-service and managed care Medicare patients. “Medicaid” includes both fee-for-service and managed care Medicaid patients. “Private insurance” includes Blue Cross, commercial carriers, and private HMOs and PPOs. “Other” includes Worker's Compensation, CHAMPUS (Civilian Health and Medical Program of the Uniformed Services), CHAMPVA (Civilian Health and Medical Program of the Department of Veteran's Affairs), Title V, and other government programs.

^h^ “Patient location” is a six-category urban-rural classification scheme for U.S. counties developed by the National Center for Health Statistics (NCHS) especially for use in health care research. The classification emphasizes urban distinctions and is unique in differentiating between central and fringe counties of large metropolitan areas. Smaller metropolitan counties are subdivided by population. Non-metropolitan counties are divided simply into micropolitan and non-core categories.

**Supplementary Table S32. Nonfatal Opioid Overdose^a^ (2016 to 2019 and 2020 to 2021) in Nationwide Emergency Department Sample Overall and for Subtypes by Demographic Characteristics**

| **Characteristics** | **Between 2016 and 2019** | | | **During the COVID-19 pandemic in 2020 and 2021** | | |
| --- | --- | --- | --- | --- | --- | --- |
|  | **2019 Rate,**  **per 10,000** | **Absolute difference**  **from 2016-2019^b^** | **Change**  **from 2016-2019, %** | **2021 Rate,**  **per 10,000** | **Absolute difference**  **from 2019-2021^c^** | **Change**  **from 2019-2021, %** |
| **Overall** | 23.0  (20.8 to 25.1) | -1.8  (-5.0 to 1.4) | -7.3  (-20.1 to 5.6) | 28.7  (26.6 to 30.9) | 5.7  (2.7 to 8.7) | 24.8  (11.5 to 38.0) |
| **AGE** |  |  |  |  |  |  |
| **18-44** | 31.2  (28.4 to 34.0) | -1.6  (-6.4 to 3.2) | -4.9  (-19.5 to 9.7) | 40.4  (37.6 to 43.1) | 9.2  (5.3 to 13.1) | 29.5  (17.0 to 41.9) |
| **45-64** | 23.2  (19.8 to 26.5) | -0.9  (-4.7 to 2.9) | -3.7  (-19.4 to 11.9) | 28.1  (24.9 to 31.4) | 4.9  (0.3 to 9.5) | 21.1  (1.4 to 40.8) |
| **65-84** | 10.0  (9.1 to 10.8) | -0.8  (-1.8 to 0.2) | -7.4  (-16.5 to 1.7) | 11.2  (10.2 to 12.3) | 1.2  (-0.1 to 2.5) | 12.0  (-0.6 to 24.6) |
| **≥85** | 3.2  (2.9 to 3.6) | -0.9  (-1.5 to -0.3) | -22.0  (-35.5 to -8.4) | 3.8  (3.1 to 4.5) | 0.6  (-0.3 to 1.5) | 18.8  (-8.6 to 46.1) |
| **Sex** |  |  |  |  |  |  |
| **Male** | 33.2  (29.6 to 36.8) | -1.1  (-6.1 to 3.9) | -3.2  (-17.8 to 11.3) | 42.3  (39.0 to 45.7) | 9.1  (4.2 to 14.0) | 27.4  (12.8 to 42.0) |
| **Female** | 15.1  (14.0 to 16.2) | -2.5  (-4.3 to -0.7) | -14.2  (-24.5 to -3.9) | 17.6  (16.5 to 18.8) | 2.5  (0.8 to 4.2) | 16.6  (5.5 to 27.6) |
| **Race/ethnicity^e^** |  |  |  |  |  |  |
| **Non-Hispanic White** | 27.5  (25.5 to 29.6) | NA^d^ | NA^d^ | 33.0  (30.9 to 35.1) | 5.5  (2.5 to 8.5) | 20.0  (8.9 to 31.1) |
| **Non-Hispanic Black** | 17.7  (12.8 to 22.5) | NA^d^ | NA^d^ | 24.4  (19.3 to 29.4) | 6.7  (-0.4 to 13.8) | 37.9  (-2.1 to 77.8) |
| **Hispanic** | 15.0  (12.3 to 17.7) | NA^d^ | NA^d^ | 20.0  (17.4 to 22.6) | 5.0  (1.3 to 8.7) | 33.3  (8.4 to 58.3) |
| **Non-Hispanic Asian or Pacific Islander** | 12.5  (0.4 to 24.7) | NA^d^ | NA^d^ | 7.5  (6.3 to 8.7) | -5.0  (-17.2 to 7.2) | -40.0  (-137.7 to 57.7) |
| **Native American** | 22.3  (15.4 to 29.1) | NA^d^ | NA^d^ | 59.9  (34.2 to 85.6) | 37.6  (11.0 to 64.2) | 168.6  (49.4 to 287.8) |
| **Other** | 17.1  (14.7 to 19.5) | NA^d^ | NA^d^ | 29.3  (25.5 to 33.1) | 12.2  (7.8 to 16.6) | 71.3  (45.6 to 97.1) |
| **Zip code income quartile^f^** |  |  |  |  |  |  |
| **1 (lowest)** | 23.2  (20.0 to 26.3) | -0.4  (-4.8 to 4.0) | -1.7  (-20.5 to 17.1) | 31.5  (28.1 to 34.9) | 8.3  (3.7 to 12.9) | 35.8  (16.1 to 55.5) |
| **2** | 21.9  (20.0 to 23.8) | -2.1  (-5.3 to 1.1) | -8.8  (-22.1 to 4.6) | 27.5  (25.4 to 29.5) | 5.6  (2.8 to 8.4) | 25.6  (12.9 to 38.2) |
| **3** | 23.2  (21.1 to 25.3) | -2.8  (-6.0 to 0.4) | -10.8  (-23.0 to 1.5) | 27.0  (24.6 to 29.3) | 3.8  (0.6 to 7.0) | 16.4  (2.6 to 30.1) |
| **4 (highest)** | 21.9  (19.6 to 24.2) | -3.5  (-7.1 to 0.1) | -13.8  (-28.0 to 0.4) | 23.7  (21.5 to 25.9) | 1.8  (-1.4 to 5.0) | 8.2  (-6.3 to 22.8) |
| **Primary expected payer^g^** |  |  |  |  |  |  |
| **Medicare** | 13.7  (12.8 to 14.6) | -3.3  (-4.6 to -2.0) | -19.4  (-26.8 to -12.0) | 15.1  (14.1 to 16.2) | 1.4  (0.01 to 2.8) | 10.2  (0.1 to 20.3) |
| **Medicaid** | 38.2  (33.3 to 43.2) | 1.7  (-5.0 to 8.4) | 4.7  (-13.6 to 22.9) | 48.6  (44.0 to 53.1) | 10.4  (3.7 to 17.1) | 27.2  (9.8 to 44.7) |
| **Private insurance** | 12.0  (11.1 to 12.9) | -3.8  (-5.5 to -2.1) | -24.1  (-34.7 to -13.4) | 14.8  (13.0 to 16.5) | 2.8  (0.8 to 4.8) | 23.3  (6.5 to 40.1) |
| **Self-pay** | 40.1  (35.0 to 45.3) | 1.3  (-6.3 to 8.9) | 3.4  (-16.3 to 23.0) | 57.2  (51.8 to 62.6) | 17.1  (9.6 to 24.6) | 42.6  (24.0 to 61.3) |
| **No charge** | 50.3  (35.5 to 65.1) | -18.4  (-93.2 to 56.4) | -26.8  (-135.6 to 82.0) | 81.6  (48.1 to 115.1) | 31.3  (-5.3 to 67.9) | 62.2  (-10.5 to 135.0) |
| **Other** | 20.0  (10.8 to 29.2) | -1.8  (-12.6 to 9.0) | -8.3  (-57.9 to 41.4) | 20.9  (17.1 to 24.7) | 0.9  (-9.0 to 10.8) | 4.5  (-45.2 to 54.2) |
| **Patient Location^h^** |  |  |  |  |  |  |
| **"Central" counties of metro areas of >=1 million population** | 23.8  (18.4 to 29.3) | 2.5  (-3.7 to 8.7) | 11.7  (-17.5 to 41.0) | 31.0  (26.1 to 35.9) | 7.2  (-0.2 to 14.6) | 30.3  (-0.7 to 61.2) |
| **"Fringe" counties of metro areas of >=1 million population** | 27.2  (23.9 to 30.4) | -4.8  (-10.9 to 1.3) | -15.0  (-34.0 to 4.0) | 29.5  (26.3 to 32.8) | 2.3  (-2.4 to 7.0) | 8.5  (-8.9 to 25.8) |
| **Counties in metro areas of 250,000-999,999 population** | 23.5  (20.6 to 26.4) | -6.2  (-13.3 to 0.9) | -20.9  (-44.8 to 3.0) | 30.8  (27.0 to 34.7) | 7.3  (2.4 to 12.2) | 31.1  (10.2 to 51.9) |
| **Counties in metro areas of 50,000-249,999 population** | 18.6  (16.3 to 20.9) | -1.1  (-5.0 to 2.8) | -5.6  (-25.5 to 14.3) | 26.2  (22.6 to 29.8) | 7.6  (3.4 to 11.8) | 40.9  (18.1 to 63.7) |
| **Micropolitan counties** | 18.0  (15.4 to 20.6) | -2.5  (-6.2 to 1.2) | -12.2  (-30.5 to 6.1) | 22.0  (19.4 to 24.7) | 4.0  (0.4 to 7.6) | 22.2  (2.2 to 42.2) |
| **Not metropolitan or micropolitan counties** | 12.2  (11.2 to 13.2) | -2.4  (-4.1 to -0.7) | -16.4  (-28.0 to -4.9) | 16.0  (14.6 to 17.4) | 3.8  (2.1 to 5.5) | 31.1  (17.3 to 45.0) |
| **Hospital region** |  |  |  |  |  |  |
| **Northeast** | 26.6  (22.6 to 30.5) | -4.3  (-11.7 to 3.1) | -13.9  (-37.9 to 10.0) | 33.0  (28.3 to 37.7) | 6.4  (0.3 to 12.5) | 24.1  (1.0 to 47.1) |
| **Midwest** | 28.3  (21.5 to 35.1) | -1.0  (-9.5 to 7.5) | -3.4  (-32.6 to 25.8) | 30.4  (24.5 to 36.2) | 2.1  (-6.9 to 11.1) | 7.4  (-24.5 to 39.3) |
| **South** | 21.3  (18.2 to 24.4) | -0.7  (-5.6 to 4.2) | -3.2  (-25.3 to 18.9) | 27.6  (24.3 to 30.9) | 6.3  (1.7 to 10.9) | 29.6  (8.1 to 51.1) |
| **West** | 16.8  (15.1 to 18.4) | -2.2  (-5.2 to 0.8) | -11.6  (-27.3 to 4.2) | 25.2  (22.6 to 27.8) | 8.4  (5.4 to 11.4) | 50 .0  (32.2 to 67.8) |

^a^ Cases of nonfatal opioid overdose were identified following the study conducted by Barnett et al.(ICD-10-CM code used: found in Table S 3).

^b^ Reflects the overall or total difference between 2016 and 2019: absolute difference (2019 – 2016) and percentage change [(2019 – 2016)/2016 × 100].

^c^ Reflects the overall or total difference during the COVID-19 pandemic in 2020 and 2021: absolute difference (2021 – 2019) and percentage change [(2021 – 2019)/2019 × 100].

^d^ NA, Not Available. Due to the NEDS survey design, the Race/ethnicity classification was not available until 2019 and therefore could not present estimates.

^e^ Race/ethnicity variable was obtained from the Agency for Healthcare Research and Quality, Healthcare Cost and Utilization Project; nationally weighted from all records in states with reliable race/ethnicity reporting, which is collected by self-report on admission to the hospital using fixed categories.

^f^ Median household income of residents in the patient’s zip code was updated annually; 2019 range for quartile 1 was less than $48,000; quartile 2, $48,000 to $60,999; quartile 3, $61,000 to $81,999; and quartile 4, at least $82,000; 2021 range for quartile 1 was less than $52,000; quartile 2, $52,000 to $65,999; quartile 3, $66,000 to $87,999; and quartile 4, at least $88,000.

^g^ “Medicare” includes both fee-for-service and managed care Medicare patients. “Medicaid” includes both fee-for-service and managed care Medicaid patients. “Private insurance” includes Blue Cross, commercial carriers, and private HMOs and PPOs. “Other” includes Worker's Compensation, CHAMPUS (Civilian Health and Medical Program of the Uniformed Services), CHAMPVA (Civilian Health and Medical Program of the Department of Veteran's Affairs), Title V, and other government programs.

^h^ “Patient location” is a six-category urban-rural classification scheme for U.S. counties developed by the National Center for Health Statistics (NCHS) especially for use in health care research. The classification emphasizes urban distinctions and is unique in differentiating between central and fringe counties of large metropolitan areas. Smaller metropolitan counties are subdivided by population. Non-metropolitan counties are divided simply into micropolitan and non-core categories.

**Supplementary Table S33. Injection Drug-Use Related Acute Infection^a^ (2016 to 2019 and 2020 to 2021) in Nationwide Emergency Department Sample Overall and for Subtypes by Demographic Characteristics**

| **Characteristics** | **Between 2016 and 2019** | | | **During the COVID-19 pandemic in 2020 and 2021** | | |
| --- | --- | --- | --- | --- | --- | --- |
|  | **2019 Rate,**  **per 10,000** | **Absolute difference**  **from 2016-2019^b^** | **Change**  **from 2016-2019, %** | **2021 Rate,**  **per 10,000** | **Absolute difference**  **from 2019-2021^c^** | **Change**  **from 2019-2021, %** |
| **Overall** | 5.5  (5.0 to 6.0) | 0.5  (-0.3 to 1.3) | 10.0  (-6.6 to 26.6) | 5.5  (5.0 to 6.0) | 0  (-0.8 to 0.8) | 0  (-15.1 to 15.1) |
| **AGE** |  |  |  |  |  |  |
| **18-44** | 8.1  (7.3 to 8.8) | 0.7  (-0.4 to 1.8) | 9.5  (-5.5 to 24.4) | 7.7  (6.9 to 8.4) | -0.4  (-1.5 to 0.7) | -4.9  (-18.6 to 8.7) |
| **45-64** | 5.7  (5.1 to 6.2) | 1.2  (0.5 to 1.9) | 26.7  (11.0 to 42.4) | 6.0  (5.4 to 6.7) | 0.3  (-0.5 to 1.1) | 5.3  (-9.3 to 19.9) |
| **65-84** | 1.2  (1.0 to 1.4) | 0.5  (0.2 to 0.8) | 71.4  (31.8 to 111.0) | 1.4  (1.3 to 1.6) | 0.2  (-0.1 to 0.5) | 16.7  (-6.4 to 39.8) |
| **≥85** | NR^d^ | NR^d^ | NR^d^ | NR^d^ | NR^d^ | NR^d^ |
| **Sex** |  |  |  |  |  |  |
| **Male** | 7.5  (6.8 to 8.2) | 0.8  (-0.03 to 1.6) | 11.9  (-0.5 to 24.4) | 7.7  (6.9 to 8.4) | 0.2  (-0.8 to 1.2) | 2.7  (-10.4 to 15.7) |
| **Female** | 4.0  (3.6 to 4.4) | 0.3  (-0.3 to 0.9) | 8.1  (-6.9 to 23.1) | 3.7  (3.3 to 4.0) | -0.3  (-0.9 to 0.3) | -7.5  (-21.4 to 6.4) |
| **Race/ethnicity^e^** |  |  |  |  |  |  |
| **Non-Hispanic White** | 6.9  (6.2 to 7.5) | NA^i^ | NA^i^ | 6.8  (6.2 to 7.4) | -0.1  (-0.9 to 0.7) | -1.4  (-13.5 to 10.6) |
| **Non-Hispanic Black** | 2.9  (2.2 to 3.5) | NA^i^ | NA^i^ | 3.1  (2.5 to 3.8) | 0.2  (-0.6 to 1.0) | 6.9  (-21.8 to 35.6) |
| **Hispanic** | 5.0  (4.3 to 5.8) | NA^i^ | NA^i^ | 4.6  (3.8 to 5.4) | -0.4  (-1.5 to 0.7) | -8.0  (-30.2 to 14.2) |
| **Non-Hispanic Asian or Pacific Islander** | 1.1  (0.7 to 1.6) | NA^i^ | NA^i^ | 1.2  (0.7 to 1.6) | 0.1  (-0.5 to 0.7) | 9.1  (-41.3 to 59.5) |
| **Native American** | 9.2  (6.1 to 12.3) | NA^i^ | NA^i^ | 9.9  (7.2 to 12.6) | 0.7  (-3.5 to 4.9) | 7.6  (-37.7 to 52.9) |
| **Other** | 3.8  (3.2 to 4.5) | NA^i^ | NA^i^ | 4.6  (3.6 to 5.6) | 0.8  (-0.3 to 1.9) | 21.1  (-9.0 to 51.1) |
| **Zip code income quartile^f^** |  |  |  |  |  |  |
| **1 (lowest)** | 6.0  (5.2 to 6.9) | 0.8  (-0.3 to 1.9) | 15.4  (-5.9 to 36.7) | 5.9  (5.2 to 6.5) | -0.1  (-1.1 to 0.9) | -1.7  (-18.0 to 14.7) |
| **2** | 5.0  (4.4 to 5.5) | 0.4  (-0.3 to 1.1) | 8.7  (-6.7 to 24.1) | 4.9  (4.4 to 5.4) | -0.1  (-0.8 to 0.6) | -2  (-16.1 to 12.1) |
| **3** | 4.9  (4.4 to 5.4) | 0  (-0.8 to 0.8) | 0  (-17.0 to 17.0) | 5.4  (4.4 to 6.4) | 0.5  (-0.6 to 1.6) | 10.2  (-13.1 to 33.5) |
| **4 (highest)** | 4.4  (3.8 to 4.9) | -0.1  (-0.9 to 0.7) | -2.2  (-20.7 to 16.3) | 4.1  (3.5 to 4.7) | -0.3  (-1.1 to 0.5) | -6.8  (-25.7 to 12.1) |
| **Primary expected payer^g^** |  |  |  |  |  |  |
| **Medicare** | 2.4  (2.1 to 2.6) | 0.4  (0.1 to 0.7) | 20.0  (6.1 to 33.9) | 2.6  (2.3 to 2.8) | 0.2  (-0.1 to 0.5) | 8.3  (-3.2 to 19.9) |
| **Medicaid** | 12.7  (11.4 to 14.0) | 1.9  (-0.04 to 3.8) | 17.6  (-0.4 to 35.6) | 11.7  (10.6 to 12.8) | -1.0  (-2.8 to 0.8) | -7.9  (-22.1 to 6.4) |
| **Private insurance** | 1.8  (1.6 to 2.1) | -0.3  (-0.6 to -0.02) | -14.3  (-27.5 to -1.1) | 1.9  (1.6 to 2.1) | 0.1  (-0.2 to 0.4) | 5.6  (-9.8 to 21.0) |
| **Self-pay** | 7.8  (6.4 to 9.2) | 1.0  (-0.6 to 2.6) | 14.7  (-8.5 to 37.9) | 8.9  (7.0 to 10.8) | 1.1  (-1.3 to 3.5) | 14.1  (-16.6 to 44.8) |
| **No charge** | 13.4  (7.0 to 19.8) | 4.2  (-3.1 to 11.5) | 45.7  (-33.4 to 124.7) | 14.1  (7.7 to 20.5) | 0.7  (-8.4 to 9.8) | 5.2  (-63.0 to 73.5) |
| **Other** | 4.5  (3.1 to 5.9) | 0.7  (-1.0 to 2.4) | 18.4  (-25.9 to 62.8) | 3.4  (2.6 to 4.1) | -1.1  (-2.7 to 0.5) | -24.4  (-59.6 to 10.7) |
| **Patient Location^h^** |  |  |  |  |  |  |
| **"Central" counties of metro areas of >=1 million population** | 6.4  (5.4 to 7.4) | 0.2  (-1.3 to 1.7) | 3.2  (-21.5 to 27.9) | 6.2  (5 to 7.3) | -0.2  (-1.7 to 1.3) | -3.1  (-27.0 to 20.8) |
| **"Fringe" counties of metro areas of >=1 million population** | 4.8  (4.2 to 5.5) | -0.3  (-1.3 to 0.7) | -5.9  (-25.1 to 13.3) | 4.7  (3.9 to 5.5) | -0.1  (-1.1 to 0.9) | -2.1  (-22.5 to 18.3) |
| **Counties in metro areas of 250,000-999,999 population** | 6.4  (5.4 to 7.5) | 1.0  (-0.5 to 2.5) | 18.5  (-9.8 to 46.9) | 5.7  (4.9 to 6.6) | -0.7  (-2.1 to 0.7) | -10.9  (-33.0 to 11.1) |
| **Counties in metro areas of 50,000-249,999 population** | 3.7  (3.1 to 4.4) | 0.3  (-0.7 to 1.3) | 8.8  (-20.0 to 37.6) | 5.5  (4.5 to 6.5) | 1.8  (0.7 to 2.9) | 48.6  (17.8 to 79.5) |
| **Micropolitan counties** | 3.7  (3.0 to 4.4) | 0.5  (-0.3 to 1.3) | 15.6  (-10.4 to 41.6) | 4.0  (3.3 to 4.7) | 0.3  (-0.7 to 1.3) | 8.1  (-18.4 to 34.6) |
| **Not metropolitan or micropolitan counties** | 2.4  (1.8 to 3.0) | 0.6  (-0.1 to 1.3) | 33.3  (-5.9 to 72.6) | 2.8  (2.3 to 3.3) | 0.4  (-0.4 to 1.2) | 16.7  (-18.0 to 51.3) |
| **Hospital region** |  |  |  |  |  |  |
| **Northeast** | 5.9  (4.9 to 6.9) | 0.2  (-1.3 to 1.7) | 3.5  (-23.3 to 30.4) | 7.3  (5.4 to 9.2) | 1.4  (-0.8 to 3.6) | 23.7  (-13.4 to 60.9) |
| **Midwest** | 4.2  (3.3 to 5.1) | -0.1  (-1.6 to 1.4) | -2.3  (-37.9 to 33.3) | 3.6  (3 to 4.2) | -0.6  (-1.7 to 0.5) | -14.3  (-41.5 to 12.9) |
| **South** | 4.9  (4.0 to 5.8) | 1.1  (-0.04 to 2.2) | 28.9  (-1.1 to 59.0) | 4.8  (4.1 to 5.4) | -0.1  (-1.2 to 1.0) | -2.0  (-25.4 to 21.3) |
| **West** | 8.0  (6.8 to 9.3) | 0.5  (-1.3 to 2.3) | 6.7  (-17.4 to 30.8) | 7.3  (6.1 to 8.5) | -0.7  (-2.4 to 1.0) | -8.8  (-29.5 to 12.0) |

^a^ Cases of injection drug-use related acute infection were identified following the study conducted by Barnett et al. (ICD-10-CM code used: found in Table S3).

^b^ Reflects the overall or total difference between 2016 and 2019: absolute difference (2019 – 2016) and percentage change [(2019 – 2016)/2016 × 100].

^c^ Reflects the overall or total difference during the COVID-19 pandemic in 2020 and 2021: absolute difference (2021 – 2019) and percentage change [(2021 – 2019)/2019 × 100].

^d^ NR, Not Reportable. Suppressed to protect confidentiality, ≤10 cases; NA, Not Available. Due to the NEDS survey design, the Race/ethnicity classification was not available until 2019 and therefore could not present estimates.

^e^ Race/ethnicity variable was obtained from the Agency for Healthcare Research and Quality, Healthcare Cost and Utilization Project; nationally weighted from all records in states with reliable race/ethnicity reporting, which is collected by self-report on admission to the hospital using fixed categories.

^f^ Median household income of residents in the patient’s zip code was updated annually; 2019 range for quartile 1 was less than $48,000; quartile 2, $48,000 to $60,999; quartile 3, $61,000 to $81,999; and quartile 4, at least $82,000; 2021 range for quartile 1 was less than $52,000; quartile 2, $52,000 to $65,999; quartile 3, $66,000 to $87,999; and quartile 4, at least $88,000.

^g^ “Medicare” includes both fee-for-service and managed care Medicare patients. “Medicaid” includes both fee-for-service and managed care Medicaid patients. “Private insurance” includes Blue Cross, commercial carriers, and private HMOs and PPOs. “Other” includes Worker's Compensation, CHAMPUS (Civilian Health and Medical Program of the Uniformed Services), CHAMPVA (Civilian Health and Medical Program of the Department of Veteran's Affairs), Title V, and other government programs.

^h^ “Patient location” is a six-category urban-rural classification scheme for U.S. counties developed by the National Center for Health Statistics (NCHS) especially for use in health care research. The classification emphasizes urban distinctions and is unique in differentiating between central and fringe counties of large metropolitan areas. Smaller metropolitan counties are subdivided by population. Non-metropolitan counties are divided simply into micropolitan and non-core categories.

^i^ NA, Not Available. Due to the NEDS survey design, the Race/ethnicity classification was not available until 2019 and therefore could not present estimates.

**Supplementary Table S34. Substance abuse Treatment^a^ (2016 to 2019 and 2020 to 2021) in Nationwide Emergency Department Sample Overall and for Subtypes by Demographic Characteristics**

| **Characteristics** | **Between 2016 and 2019** | | | **During the COVID-19 pandemic in 2020 and 2021** | | |
| --- | --- | --- | --- | --- | --- | --- |
|  | **2019 Rate,**  **per 10,000** | **Absolute difference**  **from 2016-2019^b^** | **Change**  **from 2016-2019, %** | **2021 Rate,**  **per 10,000** | **Absolute difference**  **from 2019-2021^c^** | **Change**  **from 2019-2021, %** |
| **Overall** | 3.4  (2.2 to 4.5) | -0.7  (-2.4 to 1.0) | -17.1  (-57.6 to 23.5) | 3.6  (2.5 to 4.8) | 0.2  (-1.5 to 1.9) | 5.9  (-43.0 to 54.8) |
| **AGE** |  |  |  |  |  |  |
| **18-44** | 4.5  (3.0 to 6.0) | -1.5  (-4.2 to 1.2) | -25.0  (-69.4 to 19.4) | 5.3  (3.5 to 7.0) | 0.8  (-1.6 to 3.2) | 17.8  (-34.7 to 70.2) |
| **45-64** | 4.2  (2.6 to 5.8) | 0.1  (-1.7 to 1.9) | 2.4  (-42.7 to 47.5) | 3.9  (2.7 to 5.1) | -0.3  (-2.3 to 1.7) | -7.1  (-53.8 to 39.5) |
| **65-84** | 0.7  (0.5 to 1.0) | 0.1  (-0.2 to 0.4) | 16.7  (-29.5 to 62.9) | 0.8  (0.5 to 1.1) | 0.1  (-0.2 to 0.4) | 14.3  (-25.3 to 53.9) |
| **≥85** | NR^d^ | NR^d^ | NR^d^ | NR^d^ | NR^d^ | NR^d^ |
| **Sex** |  |  |  |  |  |  |
| **Male** | 5.0  (3.2 to 6.8) | -1.1  (-3.7 to 1.5) | -18.0  (-61.3 to 25.2) | 5.5  (3.7 to 7.2) | 0.5  (-2.0 to 3.0) | 10  (-39.9 to 59.9) |
| **Female** | 2.1  (1.5 to 2.8) | -0.6  (-1.6 to 0.4) | -22.2  (-58.5 to 14.1) | 2.1  (1.5 to 2.8) | 0  (-0.8 to 0.8) | 0  (-39.6 to 39.6) |
| **Race/ethnicity^e^** |  |  |  |  |  |  |
| **Non-Hispanic White** | 3.9  (2.8 to 5.0) | NA^i^ | NA^i^ | 4.7  (3.2 to 6.1) | 0.8  (-1.0 to 2.6) | 20.5  (-25.8 to 66.8) |
| **Non-Hispanic Black** | 2.6  (0.9 to 4.3) | NA^i^ | NA^i^ | 2.0  (1.3 to 2.8) | -0.6  (-2.5 to 1.3) | -23.1  (-97.3 to 51.2) |
| **Hispanic** | 2.2  (0.8 to 3.7) | NA^i^ | NA^i^ | 2.3  (1.1 to 3.5) | 0.1  (-1.7 to 1.9) | 4.5  (-77.6 to 86.7) |
| **Non-Hispanic Asian or Pacific Islander** | 2.0  (-0.4 to 4.4) | NA^i^ | NA^i^ | 0.5  (0.2 to 0.9) | -1.5  (-3.9 to 0.9) | -75.0  (-194.2 to 44.2) |
| **Native American** | 3.3  (1.3 to 5.3) | NA^i^ | NA^i^ | 7.7  (1.6 to 13.7) | 4.4  (-2.0 to 10.8) | 133.3  (-60.1 to 326.8) |
| **Other** | 5.0  (-0.8 to 10.9) | NA^i^ | NA^i^ | 4.8  (1.7 to 7.9) | -0.2  (-6.9 to 6.5) | -4.0  (-137.3 to 129.3) |
| **Zip code income quartile^f^** |  |  |  |  |  |  |
| **1 (lowest)** | 3.0  (1.9 to 4.2) | 0  (-1.5 to 1.5) | 0  (-51 to 51) | 3.5  (2.3 to 4.6) | 0.5  (-1.2 to 2.2) | 16.7  (-38.8 to 72.1) |
| **2** | 2.9  (1.8 to 4.0) | -0.9  (-2.9 to 1.1) | -23.7  (-76.8 to 29.4) | 3.2  (1.6 to 4.7) | 0.3  (-1.5 to 2.1) | 10.3  (-53.4 to 74.1) |
| **3** | 3.6  (2.2 to 5.1) | -0.8  (-2.9 to 1.3) | -18.2  (-65.5 to 29.2) | 3.7  (2.4 to 5.0) | 0.1  (-2.0 to 2.2) | 2.8  (-55.1 to 60.7) |
| **4 (highest)** | 4.3  (2.2 to 6.4) | -2.4  (-6.5 to 1.7) | -35.8  (-97.5 to 25.9) | 4.3  (2.4 to 6.1) | 0  (-2.8 to 2.8) | 0  (-64.8 to 64.8) |
| **Primary expected payer^g^** |  |  |  |  |  |  |
| **Medicare** | 1.7  (1.2 to 2.3) | -0.3  (-1.1 to 0.5) | -15.0  (-56.6 to 26.6) | 1.9  (1.3 to 2.4) | 0.2  (-0.6 to 1.0) | 11.8  (-37.2 to 60.7) |
| **Medicaid** | 8.5  (5.1 to 12.0) | -0.2  (-4.9 to 4.5) | -2.3  (-56.6 to 52.0) | 7.8  (5.1 to 10.6) | -0.7  (-5.2 to 3.8) | -8.2  (-60.8 to 44.3) |
| **Private insurance** | 1.9  (1.1 to 2.8) | -1.2  (-2.6 to 0.2) | -38.7  (-84.3 to 6.9) | 2.5  (1.1 to 4.0) | 0.6  (-1.0 to 2.2) | 31.6  (-51.6 to 114.7) |
| **Self-pay** | 1.6  (1.1 to 2.1) | -0.7  (-1.8 to 0.4) | -30.4  (-80.1 to 19.3) | 2.3  (1.5 to 3.2) | 0.7  (-0.3 to 1.7) | 43.8  (-17.5 to 105.0) |
| **No charge** | 2.3  (0.8 to 3.9) | -11.9  (-23.8 to 0) | -83.8  (-167.4 to -0.3) | 7.1  (1.9 to 12.3) | 4.8  (-0.5 to 10.1) | 208.7  (-23.1 to 440.5) |
| **Other** | 2.4  (0.3 to 4.4) | -0.3  (-3.0 to 2.4) | -11.1  (-109.8 to 87.6) | 1.7  (0.8 to 2.6) | -0.7  (-3.1 to 1.7) | -29.2  (-127.8 to 69.5) |
| **Patient Location^h^** |  |  |  |  |  |  |
| **"Central" counties of metro areas of >=1 million population** | 5.6  (2.7 to 8.5) | 2.6  (-0.6 to 5.8) | 86.7  (-21.5 to 194.8) | 4.5  (2.3 to 6.7) | -1.1  (-4.7 to 2.5) | -19.6  (-84.7 to 45.5) |
| **"Fringe" counties of metro areas of >=1 million population** | 3.0  (1.6 to 4.4) | -3.7  (-7.5 to 0.1) | -55.2  (-111.7 to 1.3) | 4.4  (2.4 to 6.5) | 1.4  (-1.0 to 3.8) | 46.7  (-33.1 to 126.4) |
| **Counties in metro areas of 250,000-999,999 population** | 2.5  (1.5 to 3.6) | -1.1  (-2.9 to 0.7) | -30.6  (-81.9 to 20.8) | 2.3  (1.3 to 3.4) | -0.2  (-1.6 to 1.2) | -8.0  (-63.4 to 47.4) |
| **Counties in metro areas of 50,000-249,999 population** | 1.9  (1 to 2.7) | -3.9  (-8.7 to 0.9) | -67.2  (-149.5 to 15.0) | 5.0  (0.6 to 9.3) | 3.1  (-1.3 to 7.5) | 163.2  (-67.5 to 393.8) |
| **Micropolitan counties** | 2.5  (1.1 to 3.9) | -0.6  (-3.0 to 1.8) | -19.4  (-96.5 to 57.8) | 1.7  (0.8 to 2.6) | -0.8  (-2.5 to 0.9) | -32.0  (-99.4 to 35.4) |
| **Not metropolitan or micropolitan counties** | 1.2  (0.6 to 1.7) | -0.8  (-2.3 to 0.7) | -40.0  (-114.6 to 34.6) | 1.5  (0.7 to 2.3) | 0.3  (-0.7 to 1.3) | 25.0  (-56.7 to 106.7) |
| **Hospital region** |  |  |  |  |  |  |
| **Northeast** | 8.5  (3.4 to 13.5) | -5.1  (-13.2 to 3.0) | -37.5  (-96.9 to 21.9) | 9.6  (4.8 to 14.5) | 1.1  (-6.0 to 8.2) | 12.9  (-70.2 to 96.1) |
| **Midwest** | 2.7  (1.5 to 3.9) | -0.2  (-1.9 to 1.5) | -6.9  (-64.2 to 50.5) | 2.7  (0.9 to 4.4) | 0  (-2.1 to 2.1) | 0  (-78.5 to 78.5) |
| **South** | 2.2  (0.8 to 3.6) | 0.3  (-1.3 to 1.9) | 15.8  (-67.4 to 99.0) | 2.4  (1.2 to 3.5) | 0.2  (-1.6 to 2.0) | 9.1  (-73.0 to 91.2) |
| **West** | 1.7  (0.4 to 3.1) | 0.5  (-1.1 to 2.1) | 41.7  (-90.0 to 173.4) | 1.6  (0.2 to 3.0) | -0.1  (-2.0 to 1.8) | -5.9  (-120.0 to 108.3) |

^a^ Cases of substance abuse treatment were identified following the study conducted by Barnett et al.(ICD-10-CM code used: found in Table S3).

^b^ Reflects the overall or total difference between 2016 and 2019: absolute difference (2019 – 2016) and percentage change [(2019 – 2016)/2016 × 100].

^c^ Reflects the overall or total difference during the COVID-19 pandemic in 2020 and 2021: absolute difference (2021 – 2019) and percentage change [(2021 – 2019)/2019 × 100].

^d^ NR, Not Reportable. Suppressed to protect confidentiality, ≤10 cases.

^e^ Race/ethnicity variable was obtained from the Agency for Healthcare Research and Quality, Healthcare Cost and Utilization Project; nationally weighted from all records in states with reliable race/ethnicity reporting, which is collected by self-report on admission to the hospital using fixed categories.

^f^ Median household income of residents in the patient’s zip code was updated annually; 2019 range for quartile 1 was less than $48,000; quartile 2, $48,000 to $60,999; quartile 3, $61,000 to $81,999; and quartile 4, at least $82,000; 2021 range for quartile 1 was less than $52,000; quartile 2, $52,000 to $65,999; quartile 3, $66,000 to $87,999; and quartile 4, at least $88,000.

^g^ “Medicare” includes both fee-for-service and managed care Medicare patients. “Medicaid” includes both fee-for-service and managed care Medicaid patients. “Private insurance” includes Blue Cross, commercial carriers, and private HMOs and PPOs. “Other” includes Worker's Compensation, CHAMPUS (Civilian Health and Medical Program of the Uniformed Services), CHAMPVA (Civilian Health and Medical Program of the Department of Veteran's Affairs), Title V, and other government programs.

^h^ “Patient location” is a six-category urban-rural classification scheme for U.S. counties developed by the National Center for Health Statistics (NCHS) especially for use in health care research. The classification emphasizes urban distinctions and is unique in differentiating between central and fringe counties of large metropolitan areas. Smaller metropolitan counties are subdivided by population. Non-metropolitan counties are divided simply into micropolitan and non-core categories.

^i^ NA, Not Available. Due to the NEDS survey design, the Race/ethnicity classification was not available until 2019 and therefore could not present estimates.

**Supplementary Table S35. Opioid-Related Primary Diagnoses^a^ (2016 to 2019 and 2020 to 2021) in National Inpatient Sample Overall and for Subtypes Involving Opioid Categories of Adverse Event or Poisoning^b^**

| **Characteristics** | **Between 2016 and 2019** | | | **During the COVID-19 pandemic in 2020 and 2021** | | |
| --- | --- | --- | --- | --- | --- | --- |
|  | **2019 Rate,**  **per 10,000** | **Absolute difference**  **from 2016-2019^c^** | **Change**  **from 2016-2019, %** | **2021 Rate,**  **per 10,000** | **Absolute difference**  **from 2019-2021^d^** | **Change**  **from 2019-2021, %** |
| **Overall** | 36.7  (33.6 to 40.0) | -14.5  (-20.0 to -8.9) | -28.2  (-39.1 to -17.4) | 34.8  (32.2 to 37.5) | -1.9  (-6.0 to 2.2) | -5.2  (-16.4 to 6.1) |
| **Abuse or dependence** | 20.1  (17.0 to 23.2) | -10.7  (-16.2 to -5.2) | -34.7  (-52.5 to -16.9) | 16.3  (13.8 to 18.9) | -3.8  (-7.8 to 0.2) | -18.8  (-38.6 to 1.0) |
| **Adverse event or poisoning** | 16.6  (16.1 to 17.2) | -3.8  (-4.6 to -3.0) | -18.5  (-22.5 to -14.5) | 18.5  (17.8 to 19.2) | 1.9  (1.0 to 2.8) | 11.4  (6.2 to 16.6) |
| **Synthetic opioids as**  **a proxy for fentanyl** | 1.7  (1.6 to 1.8) | 0.3  (0.09 to 0.4) | 18.1  (6.4 to 29.8) | 3.4  (3.1 to 3.7) | 1.7  (1.4 to 2.00) | 99.4  (81.4 to 117.5) |
| **Prescription natural/semisynthetic opioids as a proxy for opioid pain medications** | 6.4  (6.1 to 6.6) | -1.4  (-1.7 to -1.0) | -17.9  (-22.5 to -13.2) | 7.3  (7.0 to 7.6) | 0.9  (0.6 to 1.3) | 14.7  (8.7 to 20.6) |
| **Heroin** | 5.2  (4.9 to 5.5) | -0.7  (-1.1 to -0.3) | -11.8  (-19.4 to -4.3) | 4.5  (4.2 to 4.8) | -0.7  (-1.1 to -0.3) | -13.6  (-21.4 to -5.9) |
| **Opium** | 0.09  (0.06 to 0.1) | -0.08  (-0.1 to -0.04) | -47.1  (-72.8 to -21.3) | 0.1  (0.07 to 0.1) | 0.01  (-0.02 to 0.04) | 11.1  (-19.7 to 41.9) |
| **Methadone** | 0.8  (0.7 to 0.9) | -0.5  (-0.6 to -0.3) | -37.4  (-47.6 to -27.2) | 0.7  (0.6 to 0.8) | -0.06  (-0.2 to 0.05) | -7.8  (-22.2 to 6.6) |
| **Other opioids** | 2.5  (2.4 to 2.7) | -1.4  (-1.7 to -1.2) | -36.2  (-42.6 to -29.8) | 2.5  (2.4 to 2.7) | 0.03  (-0.2 to 0.3) | 1.2  (-7.7 to 10.1) |

^a^ The primary diagnosis for patients needs to be an opioid-related diagnosis code, which identified following the definition proposed by the Healthcare Cost and Utilization Project, including two subtypes: abuse or dependence, and adverse event or poisoning (classified by opioid category, including: synthetic opioids as a proxy for fentanyl, prescription natural/semisynthetic opioids as a proxy for opioid pain medications, heroin, opium, methadone, and other opioids).

^b^ Weights provided by the Healthcare Cost and Utilization Project, National Inpatient Sample were used to ensure that the estimates were nationally representative, and weights and design variables were included to obtain unbiased estimates and standard errors.

^c^ Reflects the overall or total difference between 2016 and 2019: absolute difference (2019 – 2016) and percentage change [(2019 – 2016)/2016 × 100].

^d^ Reflects the overall or total difference during the COVID-19 pandemic in 2020 and 2021: absolute difference (2021 – 2019) and percentage change [(2021 – 2019)/2019 × 100].

**Supplementary Table S36. Opioid-Related Primary Diagnoses^a^ (2016 to 2019 and 2020 to 2021) in Nationwide Emergency Department Sample Overall and for Subtypes Involving Opioid Categories of Adverse Event or Poisoning^b^**

| **Characteristics** | **Between 2016 and 2019** | | | **During the COVID-19 pandemic in 2020 and 2021** | | |
| --- | --- | --- | --- | --- | --- | --- |
|  | **2019 Rate,**  **per 10,000** | **Absolute difference**  **from 2016-2019^c^** | **Change**  **from 2016-2019, %** | **2021 Rate,**  **per 10,000** | **Absolute difference**  **from 2019-2021^d^** | **Change**  **from 2019-2021, %** |
| **Overall** | 36.5  (33.0 to 40.0) | -2.2  (-6.9 to 2.5) | -5.7  (-17.8 to 6.3) | 42.3  (38.7 to 45.8) | 5.8  (0.8 to 10.8) | 15.9  (2.1 to 29.6) |
| **Abuse or dependence** | 17.4  (15.3 to 19.5) | -1.1  (-3.8 to 1.6) | -5.8  (-20.5 to 8.8) | 18.4  (16.0 to 20.9) | 1.0  (-2.2 to 4.2) | 5.7  (-12.7 to 24.2) |
| **Adverse event or poisoning** | 19.1  (17.1 to 21.1) | -1.1  (-4.1 to 1.8) | -5.6  (-20.1 to 8.8) | 23.8  (22.0 to 25.7) | 4.8  (2.1 to 7.5) | 25.0  (10.8 to 39.3) |
| **Synthetic opioids as**  **a proxy for fentanyl** | 1.4  (1.2 to 1.5) | 0.4  (0.2 to 0.6) | 40.2  (21.0 to 59.4) | 3.2  (2.9 to 3.6) | 1.9  (1.5 to 2.3) | 136.8  (106.5 to 167.1) |
| **Prescription natural/semisynthetic opioids as a proxy for opioid pain medications** | 4.7  (4.1 to 5.3) | 0.3  (-0.4 to 1.0) | 7.1  (-8.0 to 22.2) | 6.7  (6.2 to 7.3) | 2.0  (1.2 to 2.9) | 43.4  (25.9 to 60.9) |
| **Heroin** | 9.5  (8.4 to 10.5) | -1.9  (-3.9 to 0.2) | -16.7  (-34.6 to 1.3) | 8.6  (7.4 to 9.7) | -0.9  (-2.5 to 0.7) | -9.6  (-26.3 to 7.1) |
| **Opium** | 0.08  (0.06 to 0.1) | -0.06  (-0.09 to -0.03) | -42.9  (-62.7 to -23.1) | 0.1  (0.08 to 0.2) | 0.03  (-0.01 to 0.07) | 37.5  (-17.3 to 92.3) |
| **Methadone** | 0.3  (0.3 to 0.4) | -0.2  (-0.2 to -0.1) | -33.3  (-47.2 to -19.5) | 0.3  (0.3 to 0.4) | -0.01  (-0.07 to 0.05) | -2.9  (-19.3 to 13.4) |
| **Other opioids** | 3.1  (2.5 to 3.8) | 0.3  (-0.4 to 1.0) | 9.4  (-14.5 to 33.4) | 4.9  (4.5 to 5.4) | 1.8  (1.0 to 2.5) | 56.6  (31.9 to 81.2) |

^a^ The primary diagnosis for patients needs to be an opioid-related diagnosis code, which identified following the definition proposed by the Healthcare Cost and Utilization Project, including two subtypes: abuse or dependence, and adverse event or poisoning (classified by opioid category, including: synthetic opioids as a proxy for fentanyl, prescription natural/semisynthetic opioids as a proxy for opioid pain medications, heroin, opium, methadone, and other opioids).

^b^ Weights provided by the Healthcare Cost and Utilization Project, Nationwide Emergency Department Sample were used to ensure that the estimates were nationally representative, and weights and design variables were included to obtain unbiased estimates and standard errors.

^c^ Reflects the overall or total difference between 2016 and 2019: absolute difference (2019 – 2016) and percentage change [(2019 – 2016)/2016 × 100].

^d^ Reflects the overall or total difference during the COVID-19 pandemic in 2020 and 2021: absolute difference (2021 – 2019) and percentage change [(2021 – 2019)/2019 × 100].

**Supplementary Table S37.** **Percentage of Synthetic Opioids in Three Specific Classifications in 2021**

| **Synthetic opioids classification** | **National Inpatient Sample** | **Nationwide Emergency Department Sample** |
| --- | --- | --- |
|  | **Unweighted No. (weighted %)** | **Unweighted No. (weighted %)** |
| **Overall** | 5205 | 13,672 |
| **Fentanyl and its analogs** | 3504 (67.3) | 10,206 (74.2) |
| **Tramadol** | 1298 (24.9) | 2255 (16.9) |
| **Other synthetic opioids** | 433 (8.3) | 1288 (9.5) |

**Supplementary Table S38.** **Percentage of Nonfatal Synthetic Opioid Overdose in Three Specific Classifications in 2021**

| **Synthetic opioids classification** | **National Inpatient Sample** | **Nationwide Emergency Department Sample** |
| --- | --- | --- |
|  | **Unweighted No. (weighted %)** | **Unweighted No. (weighted %)** |
| **Overall** | 3461 | 11,083 |
| **Fentanyl and its analogs** | 2785 (80.5) | 9299 (83.7) |
| **Tramadol** | 455 (13.1) | 986 (9.1) |
| **Other synthetic opioids** | 246 (7.1) | 869 (7.9) |

**Supplementary Table S39.** **Synthetic Opioids-Related Diagnoses (2020 Q4 to 2021 Q4) in National Inpatient Sample and Nationwide Emergency Department Sample for Subtypes**

| **Synthetic opioids classification** | **Between 2020 Q4 and 2021 Q4** | | | |
| --- | --- | --- | --- | --- |
|  | **2020 Q4 Rate, per 10,000** | **2021 Q4 Rate, per 10,000** | **Absolute difference from 2020 Q4-2021 Q4** | **Change from 2020 Q4-2021 Q4, %** |
| 1. **National Inpatient Sample** | | | | |
| **Fentanyl and its analogs** | 4.6 (4.2 to 5.1) | 6.5 (5.9 to 7.0) | 1.9 (1.2 to 2.5) | 40.0 (25.3 to 54.8) |
| **Tramadol** | 2.3 (2.0 to 2.5) | 2.2 (1.9 to 2.4) | -0.1 (-0.5 to 0.2) | -4.9 (-20.2 to 10.5) |
| **Other synthetic opioids** | 0.7 (0.5 to 0.8) | 0.7 (0.6 to 0.9) | 0.07 (-0.1 to 0.3) | 10.8 (-19.1 to 40.6) |
| 1. **Nationwide Emergency Department Sample** | | | | |
| **Fentanyl and its analogs** | 3.0 (2.6 to 3.4) | 4.4 (3.9 to 4.9) | 1.4 (0.8 to 2.1) | 47.6 (26.0 to 69.3) |
| **Tramadol** | 1.0 (0.9 to 1.1) | 0.8 (0.8 to 0.9) | -0.2 (-0.3 to -0.05) | -18.5 (-32.2 to -4.7) |
| **Other synthetic opioids** | 0.4 (0.4 to 0.5) | 0.5 (0.4 to 0.6) | 0.07 (-0.03 to 0.2) | 16.3 (-6.5 to 39.1) |

**Supplementary Table S40. Nonfatal Synthetic Opioid Overdose (2020 Q4 to 2021 Q4) in National Inpatient Sample and Nationwide Emergency Department Sample for Subtypes**

| **Synthetic opioid classification** | **Between 2020 Q4 and 2021 Q4** | | | |
| --- | --- | --- | --- | --- |
|  | **2020 Q4 Rate, per 10,000** | **2021 Q4 Rate, per 10,000** | **Absolute difference from 2020 Q4-2021 Q4** | **Change from 2020 Q4-2021 Q4, %** |
| 1. **National Inpatient Sample** | | | | |
| **Fentanyl and its analogs** | 3.3 (2.9 to 3.7) | 5.4 (4.9 to 5.9) | 2.1 (1.4 to 2.7) | 62.2 (43.0 to 81.4) |
| **Tramadol** | 0.7 (0.6 to 0.8) | 0.9 (0.7 to 1.0) | 0.2 (-0.05 to 0.4) | 23.1 (-6.5 to 52.8) |
| **Other synthetic opioids** | 0.4 (0.3 to 0.5) | 0.4 (0.3 to 0.5) | 0.03 (-0.1 to 0.2) | 6.4 (-31.6 to 44.4) |
| 1. **Nationwide Emergency Department Sample** | | | | |
| **Fentanyl and its analogs** | 2.6 (2.2 to 3.0) | 4.0 (3.5 to 4.5) | 1.4 (0.8 to 2.0) | 54.5 (30.8 to 78.3) |
| **Tramadol** | 0.4 (0.3 to 0.5) | 0.4 (0.3 to 0.4) | -0.04 (-0.1 to 0.03) | -10.8 (-29.6 to 8.0) |
| **Other synthetic opioids** | 0.3 (0.2 to 0.3) | 0.3 (0.3 to 0.4) | 0.08 (0 to 0.15) | 28.0 (-1.0 to 56.9) |

**Supplementary Figure S11 Temporal trend changes in annual percent change (APC) for opioid related diagnoses in NIS (A), opioid related adverse event or poisoning in NIS (B), opioid related diagnoses in NEDS (C), and opioid related adverse event or poisoning in NEDS (D) from 2016 to 2021 based on the Joinpoint regression model. *p < 0.05. NIS, Nationwide Inpatient Sample; NEDS, Nationwide Emergency Department Sample.**

**
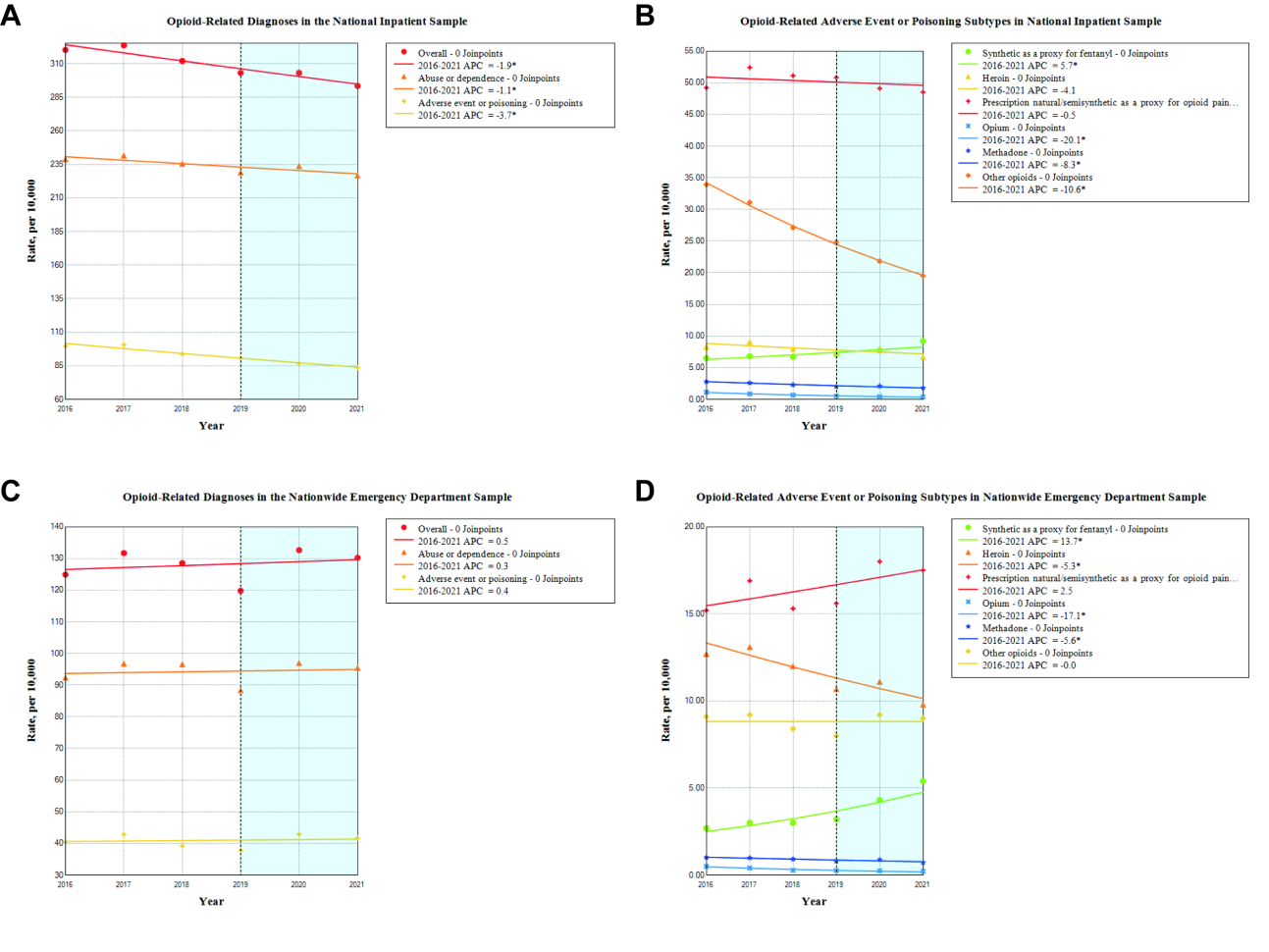
**

Note: Joinpoint methodology suggests a minimum of seven data points to allow for the presence of a joinpoint, and we calculated the annual percent change (APC) (without inflection point) using the 2016-2021 data to assess the annual trend changes.

**Supplementary Figure S12 Temporal trend changes in annual percent change (APC) for nonfatal opioid overdose and subtypes in NIS (A), injection drug-use related acute infection and subtypes in NIS (B), substance abuse treatment and subtypes in NIS (C), nonfatal opioid overdose and subtypes in NEDS (D), injection drug-use related acute infection and subtypes in NEDS (E), and substance abuse treatment and subtypes in NEDS (F) from 2016 to 2021 based on the Joinpoint regression model. *p < 0.05. NIS, Nationwide Inpatient Sample; NEDS, Nationwide Emergency Department Sample.**

**
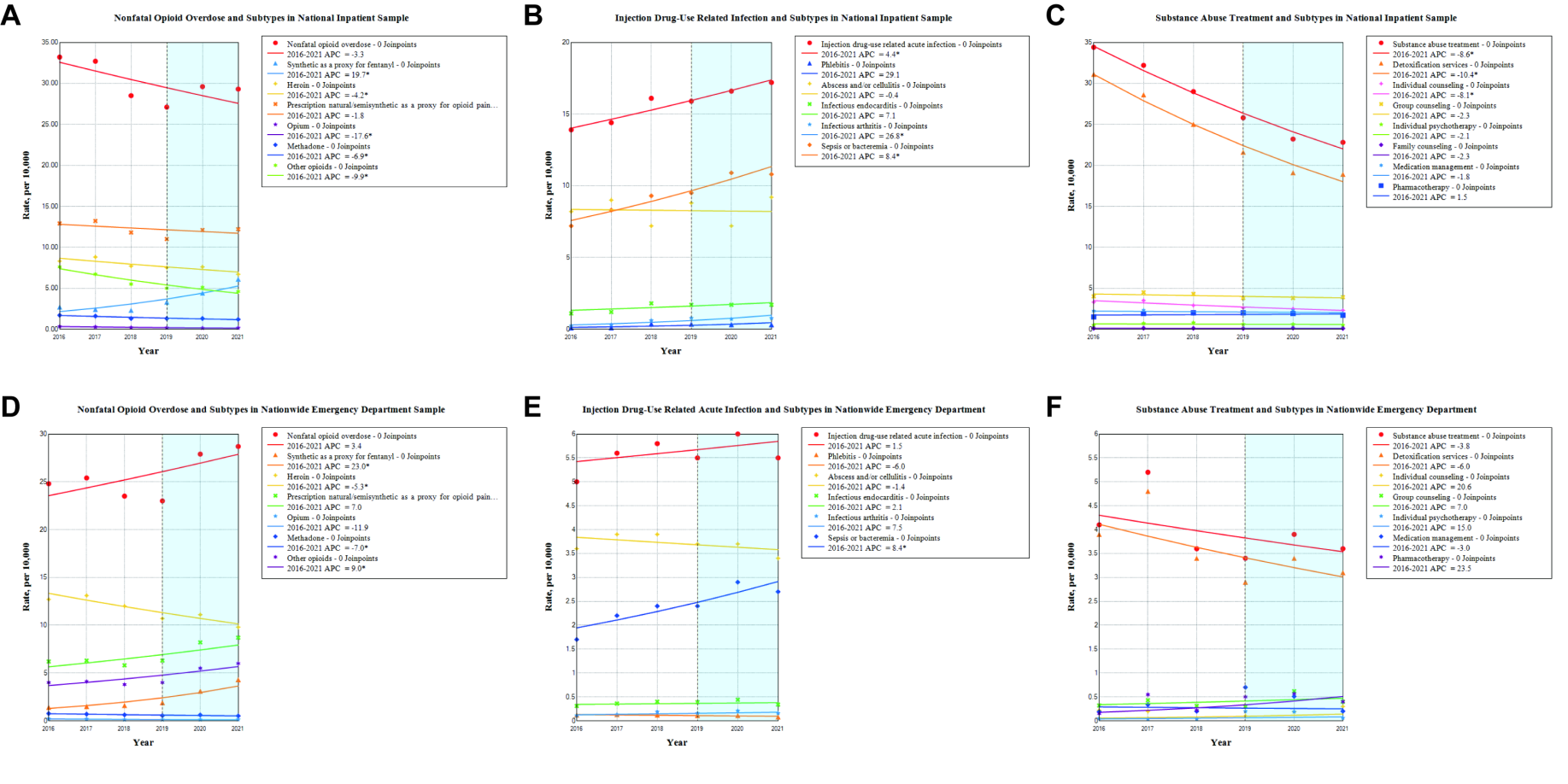
**

Note: Joinpoint methodology suggests a minimum of seven data points to allow for the presence of a joinpoint, and we calculated the annual percent change (APC) (without inflection point) using the 2016-2021 data to assess the annual trend changes.
